# Reduced evolvability of *Escherichia coli *MDS42, an IS-less cellular chassis for molecular and synthetic biology applications

**DOI:** 10.1186/1475-2859-9-38

**Published:** 2010-05-21

**Authors:** Kinga Umenhoffer, Tamás Fehér, Gabriella Balikó, Ferhan Ayaydin, János Pósfai, Frederick R Blattner, György Pósfai

**Affiliations:** 1 Institute of Biochemistry, Biological Research Center of the Hungarian Academy of Sciences, 62 Temesvari krt, H6726 Szeged, Hungary; 2 Cellular Imaging Laboratory, Biological Research Center of the Hungarian Academy of Sciences, 62 Temesvari krt, H6726 Szeged, Hungary; 3 New England Biolabs, Inc, 240 County Rd, Ipswitch, MA, 01938, USA; 4 Scarab Genomics LLC, 1202 Ann Str, Madison,WI 53713, USA; 5 Department of Genetics, University of Wisconsin, 425-G Henry Mall , Madison, WI 53706, USA

## Abstract

**Background:**

Evolvability is an intrinsic feature of all living cells. However, newly emerging, evolved features can be undesirable when genetic circuits, designed and fabricated by rational, synthetic biological approaches, are installed in the cell. Streamlined-genome *E. coli *MDS42 is free of mutation-generating IS elements, and can serve as a host with reduced evolutionary potential.

**Results:**

We analyze an extreme case of toxic plasmid clone instability, and show that random host IS element hopping, causing inactivation of the toxic cloned sequences, followed by automatic selection of the fast-growing mutants, can prevent the maintenance of a clone developed for vaccine production. Analyzing the molecular details, we identify a hydrophobic protein as the toxic byproduct of the clone, and show that IS elements spontaneously landing in the cloned fragment relieve the cell from the stress by blocking transcription of the toxic gene. Bioinformatics analysis of sequence reads from early shotgun genome sequencing projects, where clone libraries were constructed and maintained in *E. coli*, suggests that such IS-mediated inactivation of ectopic genes inhibiting the growth of the *E. coli *cloning host might happen more frequently than generally anticipated, leading to genomic instability and selection of altered clones.

**Conclusions:**

Delayed genetic adaptation of clean-genome, IS-free MDS42 host improves maintenance of unstable genetic constructs, and is suggested to be beneficial in both laboratory and industrial settings.

## Background

Molecular mechanisms generating genetic variation, coupled with selection due to the changing environment, provide the basis for the evolution of a population. While the ability to evolve is generally a necessity for survival on the long run, it might be an undesirable feature in synthetic biological (SB) applications [1]. The SB approach is ideally based on the notion that biological parts can be precisely designed and fabricated, and their interactions can be fully predicted [2]. Newly emerging, evolved features can result in undesired genotypic and phenotypic diversions of painstakingly engineered cells. Reducing the evolutionary potential of a cell, used as an SB chassis in a controlled environment, is therefore a valid goal.

In a typical bacterial cell, IS elements generate a significant share of genetic variation. Depending on the genetic and physiological context, their contribution to gene inactivation ranges from 3.9% [3] to 98% [4]. In addition to insertional inactivation, mobile elements are also capable of enhancing deletions, inversions or duplications by providing substrates for homologous recombination [5]. Under specific conditions, inhibition or stimulation of gene expression caused by genomic relocation of IS elements can help the population to overcome nutritional stress [6,7], antibiotic inhibition [8,9] or osmotic stress [10]. Moreover, in biotechnological applications, IS-mediated genomic rearrangements can cause instability of clones carrying engineered genes. Such cloning failures are by no means rare [11-17], and examples of IS-contaminated sequence artefacts obtained from shotgun sequencing projects [18-21] also indicate that the magnitude of the problem is not negligible. *E. coli *host strains in common use in research and biotechnology (e.g., MG1655, DH10B, BL21), as well as various natural isolates carry a large number of genomic ISes [22]. MG1655, the prototype K-12 strain, carries 45 ISes of 11 different kinds [23]. In contrast, MDS42, a reduced-genome derivative of MG1655, engineered to lose most of its genes irrelevant for laboratory applications, is free of active ISes [24]. It is noted, that a copy of IS*609 *is still present in MDS42. However, due to mutation resulting in a stop codon in its transposase gene (*ynck*), it displays no transposition activity (data not shown). MDS42 also lacking IS*609 *has recently become available commercially from Scarab Genomics.

In an earlier study [24], it was shown that a chimeric gene, constructed for vaccine development, and composed of a synthetic gene of the structural capsid protein VP60 of rabbit haemorrhagic disease (RHD) virus [25] fused to the gene of the B subunit of cholera toxin (CTX) was very unstable in conventional *E. coli *hosts. All recovered plasmids contained mutations in the *ctxvp60 *ORF, virtually all of which were IS insertions. In contrast, the recombinant plasmid was stable in IS-less MDS42 host; normal yields of plasmid DNA were obtained.

We analyse here the molecular details of this extreme case of instability. We identify a hydrophobic protein as the toxic byproduct of the *ctxvp60 *clone, and show that IS elements spontaneously landing in the clone relieve the cell from the stress by blocking transcription of the toxic gene. Bioinformatic analysis of raw data from various shotgun sequencing projects, where *E. coli *served as host for clone libraries, demonstrates that such IS-mediated inactivation of ectopic genes inhibiting the growth of the *E. coli *host cannot be regarded as irrelevant and rare events, but might happen in many cases, leading to genomic instability and selection of altered clones.

We show here that removal of all IS elements from the genome of a bacterial cell results in a significant increase in genomic stability and phenotypic uniformity, yielding an improved cellular chassis with reduced evolutionary potential.

## Methods

### Strains, plasmids, and media

Experiments were done using *E. coli *K-12 MG1655 [23] and various derivatives of it. MDS12, MDS30, and MDS42 are reduced-genome versions of MG1655, carrying 12, 30, and 42 genomic deletions, respectively [24,26]. T7 polymerase-expressing MG1655-T7 and MDS42-T7 strains will be described in detail elsewhere. Briefly, they carry an IPTG-inducible lac operator/T7 polymerase cassette replacing the *yahA-yaiL *genomic region of the parental strain. MDS42+IS*1 *carries a single IS*1 *in *yeaJ*. Plasmids pCTX and pCTXVP60 were originally constructed by Günther M. Keil [24] (Additional file 1). Plasmid pSG1144, carrying an F-type of replicational origin, a *trfA-oriV *replicon from plasmid R2 [27], a chloramphenicol resistance gene and a T7 promoter, was obtained from Scarab Genomics (Madison, WI, USA). Inducible variants of *orf238 *and *ctxvp60 *were constructed by cloning the genes in the multiple cloning site of pSG1144 (Additional file 1). Genes *ctxvp60*opt and *ctxvp60*dezopt were synthesized by GenScript Corporation (Piscataway, NJ, USA). Plasmids were prepared using IS-free, MDS42 host strain. Standard laboratory media LB and LB-agar plates were used during the cultivations [28]. Chloramphenicol and ampicillin were added at 25 μg/ml and 50 μg/ml final concentrations, respectively.

### PCR primers

Table 1 lists the oligonucleotides used in our experiments.

**Table 1:** Primers used in this study.

name	sequence (5'-3')	purpose
ORFstart	cgggatccatgctattgctgctatttc	cloning ORF238
ORFstop	cggaattctcataacaaattcaaaatcttcag	cloning ORF238
CTXVP2	cctcctggaactggttga	amplifying/sequencing ctxvp60
CTXVP3	ggaggatctacttctgct	amplifying/sequencing ctxvp60
CVek3	gcctggttgtacgcctgaa	amplifying/sequencing ctxvp60
T7	taatacgactcactataggg	amplifying/sequencing ctxvp60
chi3	gtgagtttcaccagttttga	amplifying/sequencing ctxvp60
vpf1	cctgggtcctattccatcagtagtagttaat	constructing ctxvp60 frame-shift
vpf2	tgatggaataggacccaggagttgttgct	constructing ctxvp60 frame-shift
IS1-1	tcgctgtcgttctca	IS*1*-specific PCR
IS1-2	aagccactggagcac	IS*1*-specific PCR
IS2-1	tcgcaggcataccatcaa	IS*2*-specific PCR
IS2-2	cagacgggttaacggca	IS*2*-specific PCR
IS3-1	agcggctggtatacgtggt	IS*3*-specific PCR
IS3-2	tcatgcgtggcgacattga	IS*3*-specific PCR
IS5-1	gacagttcggcttcgtga	IS*5*-specific PCR
IS5-2	gctcgatgacttccacca	IS*5*-specific PCR
groL1	atggcgtgggtgaagaag	amplification of control *groL*
groL2	caatctgctgacggatctga	amplification of control *groL*

### Analysis of mutations

To analyze the mutational spectrum of the *ctxvp60 *gene, a 2481-bp segment was amplified from the mutant cells using the primer pair T7/chi3. The amplified fragments were resolved on a 1% agarose gel and compared to a fragment generated from the wild-type template. Identical size indicated mutations affecting only one or a few nucleotides, a decrease in size or failure of amplification indicated a deletion, and an increase in size suggested an IS insertion. In case of insertion mutants, the identity and the localization of ISes were determined by PCR using IS- and plasmid-specific primer pairs. For 12 mutants, insertions sites were further analyzed by sequencing.

### Growth measurements and statistics

Growth characteristics were monitored in liquid medium in 100-well Honeycomb 2 plates (Oy Growth Curves Ab, Helsinki, Finland). Growth curves were taken by following the optical densities (O.D.) at 540nm in each well using the Bioscreen C Automated Microbiology Growth Analysis System (Oy Growth Curves Ab, Helsinki, Finland). Ten parallels of resuspended colonies for each plasmid-host combination were grown to saturation in ten wells at 37°C using continuous shaking in 200 μl LB medium supplemented with 25 ug/ml chloramphenicol, if required. The *orf238 *containing plasmids were grown in the presence and in the absence of IPTG. IPTG was added to induce the cultures at 0.35 O.D. in 0.6 mM final concentration. When testing the effect of pCTXVP60 on the growth of strains harboring various numbers of IS elements, the median O.D. value of the ten parallel cultures corresponding to each strain (MG1655, MDS12, MDS30 and MDS42) was calculated and plotted for every time point.

### RNA isolation from colonies and RT PCR

After transformation with pCTXVP60, small and large colonies were collected separately from the transformation plate. Cells from small and large colonies were resuspended into fresh minimal medium and the O.D. of the suspensions were adjusted to 1.0. RNA was isolated from the suspensions using the RNEasy RNA isolation kit with RNAProtect Bacteria Reagent (Qiagen, Düsseldorf, Germany), supplemented with lysozyme from Chicken Egg White (Sigma, St. Louis, MO, USA). DNA contamination was digested using DNAseI from Bovine Pancreas (Boehringer Mannheim, Germany) for 45 min at 37°C in the presence of 10 mM Tris (Sigma, St. Louis, MO, USA) and 2.25 mM MgCl_2 _(Fermentas, Vilnius, Lithuania). The reaction was stopped by adding 2.5 mM EDTA (Merck, Darmstadt, Germany) and incubation at 65°C for 10 min. cDNA was synthesized from purified- and DNase-treated RNA samples using the Transcriptor First Strand cDNA Synthesis Kit (Roche, Mannheim, Germany) with random hexanucleotide primers, according to manufacturer's instructions. The cDNA samples were used as templates in the following PCR using ORFstart and ORFstop primers, specific to the previously identified small ORF region of the *ctxvp60 *transcript. Primers groL1 and groL2 were used as positive controls.

### Cell imaging

Cells were incubated 30 mins. at 37°C with 10 μM nonyl acridine orange (NAO) and 10 μg/ml Ethidium bromide (EtBr). DNA staining was performed using 500 ng/ml 4',6-diamidino-2-phenylindole (DAPI) for 10 minutes after 70% ethanol fixation (30 mins) and PBS washes. Two percent agarose gel blocks (1 cm × 1 cm) were used to immobilize the cells on cover slips before microscopy observation. NAO, EtBr and DAPI were from Invitrogen, Carlsbad, CA, USA. Confocal laser scanning microscopy was performed using Olympus Fluoview FV1000 confocal laser scanning microscope (Olympus Life Science Europa GmbH, Hamburg, Germany) equipped with UPLSAPO 60× (oil, NA:1.35) objective. Using Olympus Fluoview software (version 1.7.2.2), DAPI images were pseudo colored red.

### Bioinformatic analysis of *E. coli *IS sequences in shotgun data

Shotgun sequencing data from early genome sequencing projects (Sanger method), where clone libraries of various genomes were constructed and maintained in *E. coli *host, provide a large dataset for checking the frequency of host IS contamination of the cloned sequences. Sequence reads were fetched from the NCBI Trace Archive. Bacterial and archaeal genomes were analyzed when individual read sequences, assembled sequence, annotations, and experimental notes were available. We built two BLAST databases for each analyzed organism, one from the read sequences, and the other from the assembled genome. A representative set of *E. coli *K12 MG1655 (U00096) IS elements was compiled, and expanded by an IS*10 *sequence from DH10B (NC_010473). Database queries were done by nucleotide level BLAST searches. To produce a conservative estimate for the number of insertion events, stringent parameter settings (expectation value 0.1, minimum score 75, minimum run length 40 nucleotides) were used. Reads from possibly non-cloning sequencing, with "finishing", "primerwalk", "454" annotations were carefully removed.

The first pass of BLAST used IS sequence baits against shotgun read databases. This round of BLAST caught reads that contain some *E. coli *IS-like sequences. With these reads as baits, a second round of BLAST mapped the genomic sequence parts of the reads onto the assembled genomes. Using IS baits, genome segments with matches to *E. coli *IS sequences were identified next. Potentially, these segments contain endogenous IS elements (and not elements acquired in the cloning process), and were excluded from further analysis. Using the remaining segments as queries, the read databases were searched, and all the reads matching to IS-acquiring segments were identified. Finally, reads collected at this pass were BLAST-ed against the assembled genomes, to find and retain only the reciprocal best hits. This back-and-forth BLAST-ing identified genome segments from which some cloning inserts acquired *E. coli *IS elements during the shotgun sequencing process. Also, the procedure yielded the reads that genuinely map to these segments, including those that contain acquired *E. coli *IS sequences. To find the functions of the genes encoded in these segments, GenBank annotations were parsed. The relationships of reads, IS elements, genome segments and gene annotations were overlaid in simple diagrams.

***Supporting online material***. Nucleotide sequences of pCTXVP60, pCTXVP60frameshift, pSG1144-*orf238*, pSG1144-*ctxvp60*, pSG1144-*ctxvp60opt*, and pSG1144-*ctxvp60dezopt*, codon use of *ctxvp60*opt and *ctxvp60*dezopt, as well as data related to the structure prediction of ORF238 can be found at http://www.brc.hu/genome, and at the end of this article (Additional files 1, 2, 3 and 4).

## Results

### Instability of pCTXVP60 clones propagated in wt *E. coli *MG1655

**Figure 1:**
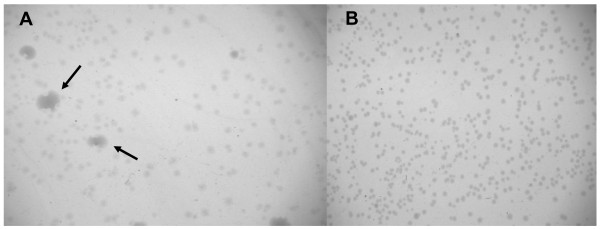
**Transformants of pCTXVP60/MG1655 (A) and pCTXVP60/MDS42 (B) on LB plates containing chloramphenicol**. Arrows indicate sectors of colonies that have arisen by outgrowth of cells harboring mutated plasmids.

**Figure 2:**
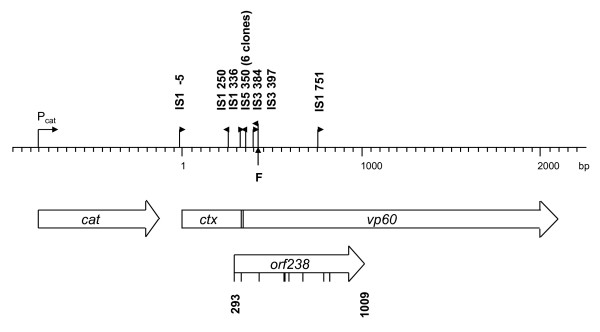
**Map of *ctxvp60*/*orf238 *with IS insertion sites**. Tick-marks on *orf238 *indicate potential translational re-initiation sites. F marks the site of a frame-shift mutation introduced in the 5' end of *vp60 *(Additional file 1).

In an earlier study [24], it was found that an expression construct of a fusion gene, coding for adjuvant CTX B subunit fused to VP60, could not be stably propagated in MG1655. Upon transformation by plasmid pCTXVP60, heterogeneous colonies of transformants were obtained on agar plates (Fig. 1A). While the majority of the colonies were small, slow-growing, and translucent, a few percent showed normal growth and appearance, as compared to control pCTX clones (not shown). Upon prolonged incubation, eventually all small colonies started to develop sectors of normal growth. A preliminary analysis of the cells from normal, healthy colonies showed that the plasmids recovered had altered restriction digestion patterns, primarily due to IS element insertions in the fusion gene [24]. An extended analysis of 100 colonies by PCR spanning the fusion gene confirmed the extreme instability of the plasmid. A vast majority, 92%, of the large colonies carried plasmids with IS insertions, while 8% displayed deletions or no alteration detectable by PCR. Insertion events were due to IS*1*, IS*3*, and IS*5 *translocations, as detected by using IS-specific PCR primers. Insertion sites were determined by sequencing 12 clones. All IS insertions occurred in the 5' third of the fusion gene, in the *ctx *part or near the 5' end of *vp60*. Results of the analysis are summarized in Fig. 2.

Similar heterogeneity of pCTXVP60 transformants was observed in other regular *E. coli *hosts, including DH5α, DH10B or C600. In contrast, transformants of IS-less MDS42 [24] displayed a nearly uniform morphology (Fig. 1B) on LB plates. Colonies generally displayed moderately retarded growth, and only < 0.1% of the colonies was larger, showing normal growth. Generally, plasmid DNA recovered from cultures using MDS42 host yielded unaltered restriction digestion pattern and nucleotide sequence (data not shown).

### Growth retardation effect of pCTXVP60 is due to an artificial byproduct, a Leu-rich ORF

**Figure 3:**
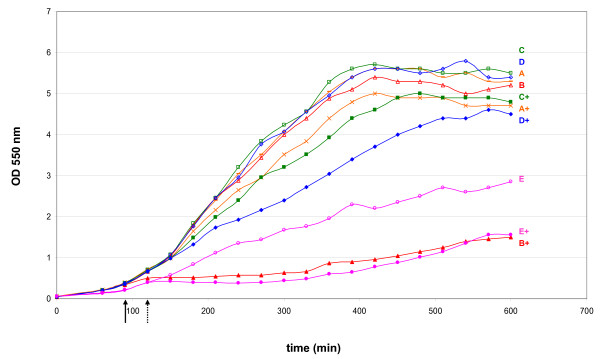
**Effect of ORF238-expression on cell growth**. MDS42-T7 cells harboring derivatives of the original *ctxvp60 *sequence inserted into the pSG1144 vector were grown in LB at 37°C. Overnight liquid cultures from colonies of freshly transformed cells were diluted into fresh medium and divided into two flasks. One of each pairs was induced by adding 1 mM of IPTG after 90 minutes growth (A+, B+, C+, D+ cultures, solid arrow), with the exception of E+, to which IPTG was added at 120 minutes (arrow with dashed line). (A, A+) pSG1144 vector (B, B+) pSG1144-*ctxvp60 *(C, C+) pSG1144-*ctxvp60opt *(D, D+) pSG1144-*ctxvp60dezopt *(E, E+) pSG1144-*orf238*.

The synthetic fusion gene *ctxvp60 *contains a large number of rare Arg ( 1 AGG, 21 AGA) codons. It was assumed that translation of *ctxvp60 *exerts a toxic effect on the cell by exhausting the rare tRNA_Arg _pool and thus rendering it unavailable for the synthesis of essential proteins. To test this hypothesis, two new versions of *ctxvp60 *were synthesized. Version *ctxvp60*opt was codon-optimized for *E. coli *and carried no rare codons, while *ctxvp60*dezopt contained the rare AGG codons for all the 22 arginines (Additional files 2 and 3). Both versions were cloned in pSG1144 (Additional file 1), using MG1655-T7 host. Surprisingly, induced expression of both constructs, optimized and deoptimized, had only a minor negative effect on the growth of the cell, comparable to that seen with the vector plasmid (Fig. 3).

On the other hand, a frame-shift mutation of *ctxvp60*, introduced in a position near the 5' end of the *vp60 *part (Fig. 2; Additional file 1), did not relieve the growth retardation effect of the plasmid (data not shown). All these results indicate that neither the CTXVP60 protein *per se*, nor the supposed rare tRNA depletion phenomenon is the cause of the toxic effect of pCTXVP60.

A thorough inspection of the original *ctxvp60 *gene revealed the presence of a 238 aa ORF (ORF238) out of the original reading frame. ORF238 spans the joint between *ctx *and *vp60 *and extends into the 5' part of *vp60 *(for coordinates, see Fig. 2). Translation of ORF238 results in an extremely Leu-rich (102 Leu residues) protein (Additional file 4). The hydrophobic nature of the protein and its four putative transmembrane domains (predicted with HMMTOP [29] and DAS-TMfilter [30,31]) strongly suggest that it integrates in the cell membranes, and might be the cause of the toxic effect (Additional file 4).

To test the hypothesis that ORF238 was the culprit of the growth defect, it was cloned as an inducible construct in plasmid pSG1144. Upon induction, expression of ORF238 resulted in a phenotype identical to that of the original *ctxvp60 *cloned in the same plasmid. Growth retardation of the culture, due to induction of ORF238, is demonstrated in Fig. 3.

**Figure 4:**
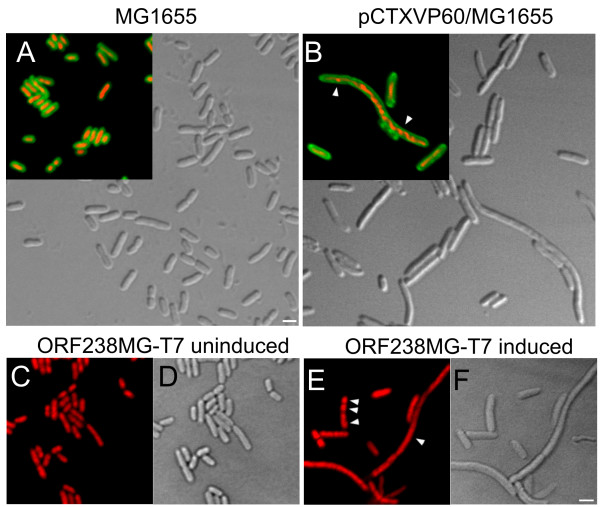
**Fluorescence microscopy images (A-F) of cells harboring control and toxic plasmids**. Plasmids and host cells are indicated for each panel. While toxic constructs pCTXVP60 (B) or induced ORF238 (E,F) give rise to abnormally long cells, untransformed MG1655 cells (A) and uninduced ORF238 (C,D) cells displayed no defects. Insets of (A) and (B) show nonyl acridine orange-stained (green) membranes superimposed onto ethidium bromide stained (red) nucleoids. DAPI stained nucleoids (pseudocolored red) are shown in (C) and (E). Arrowheads in (B) and (E) indicate abnormal, stretched or multiple nucleoids in undivided cells. Scalebars: 2 μm.

Confocal laser scanning microscopy imaging of the cells expressing either CTXVP60 or ORF238 confirmed its inhibitory role. Nucleic acid staining (Ethidium bromide or DAPI) combined with differential interference contrast (DIC) imaging and membrane staining (nonyl acridine orange) revealed that cell division is impaired, resulting in slow-growing, abnormally long cells with aberrant nucleoids (Fig. 4B, E, F). Untransformed (MG1655) or uninduced ORF238-harboring cells, on the other hand, displayed normal phenotype and size distribution (Fig 4A, C, D).

### ISes integrated in *ctxvp60 *block transcription of the Leu-rich ORF

**Figure 5:**
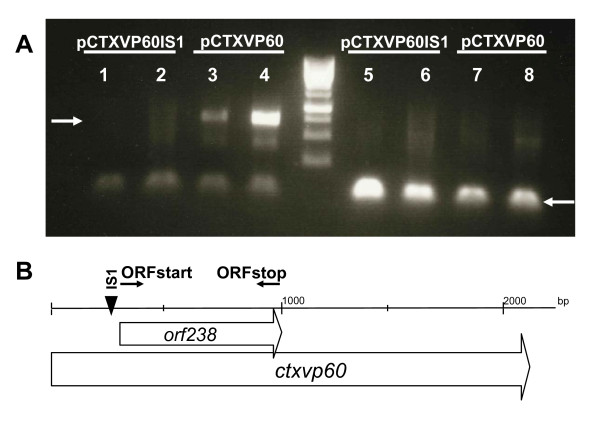
**RT PCR results confirm inhibition of transcription by IS*1 *insertion**. (A) Gel electrophoresis of RT-PCR products made from RNA prepared from cells harboring pCTXVP60IS*1 *or pCTXVP60, as indicated. Two parallel experiments were performed with each sample. Primers ORFstart and ORFstop were used in lanes 1-4, and control primers groL1 and groL2 in lanes 5-8. White arrows mark the expected sizes of the respective PCR products. (B) Map indicating the position of IS*1*, *orf238 *and PCR primers relative to the *ctxvp60 *gene.

All IS integrations, found in pCTXVP60, occurred either upstream or in the 5' end region of ORF238. It was assumed that ISes landing in this region relieve the cell from stress by blocking transcription of ORF238. To prove this, we showed that RNA prepared from cells harboring pCTXVP60 contained transcripts of the toxic gene detectable by RT-PCR. However, when picking a mutant with an IS*1 *or IS*5 *inserted into the 5' end of *ctxvp60*, transcripts of downstream sequences could not be detected (Fig. 5).

### Growth dynamics and evolution of strains harboring pCTXVP60 is related to the number of genomic ISes

**Figure 6:**
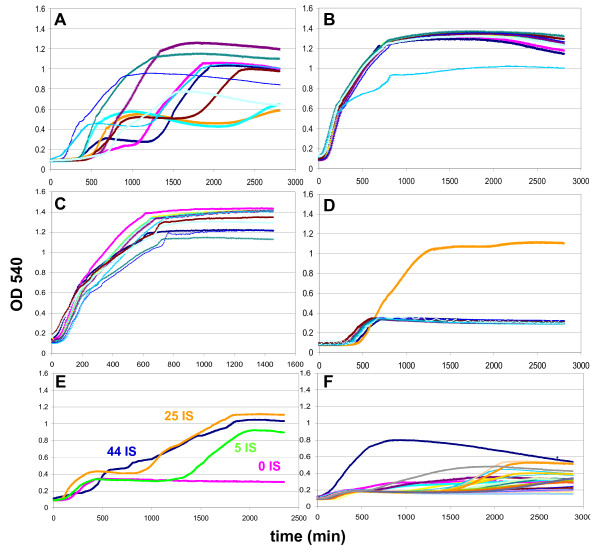
**Effect of *ctxvp60 *on cell growth**. The graphs display growth curves of individual cultures, with the exception of panel E, where each line represents the median growth curve of 10 independent cultures. Cells were grown in LB at 37°C by inoculating colonies of freshly transformed cells grown on agar+Cam plates, with the exception of panel C, which displays cultures from panel A, re-inoculated into fresh medium at 1:100 volume. (A) pCTXVP60/MG1655, (B) pCTX/MG1655 (non-toxic control), (C) Re-inoculated pCTXVP60/MG1655 cultures from panel A, (D) pCTXVP60/MDS42, (E) pCTXVP60 in MDS cells with different number of resident ISes, indicated by the numbers, (F) pCTXVP60/MDS42+IS.

Growth characteristics of transformants of toxic and mutant plasmids were monitored in liquid medium. Ten parallels originating from 10 colonies were grown for each plasmid-host combination, and automatic O.D.-readings were taken in a Bioscreen C instrument (Fig. 6).

Initial growth of MG1655/pCTXVP60 stopped at ~O.D. = 0.2-0.5. However, after prolonged incubation, individual cultures resumed growth at various time points, and reached a final density comparable to the MG1655/pCTX control (Fig. 6A, B). Plasmids isolated from these outgrown cultures almost invariably contained ISes inserted in pCTXVP60. Reinoculation of the outgrown cultures into fresh medium resulted in uninterrupted, normal growth (Fig. 6C). The results were consistent with the observed growth characteristics of transformants on agar plates: i) pCTXVP60 causes growth retardation, ii) in a stochastic manner, a fraction of the cells picks up an IS insertion in *ctxvp60*, due to the mobility of genomic ISes, iii) cells harboring an IS-inactivated ORF238 resume normal growth and quickly become dominant in the culture.

In contrast, growth of MDS42/pCTXVP60 displayed a nearly uniform pattern. Initial growth of the cultures stopped at ~O.D. = 0.3, and only one culture of 10 parallels grew eventually to high density (Fig. 6D). Plasmid isolated from this dense culture revealed a deletion in *ctxvp60*. Results indicate that the primary route to inactivation of the toxic gene is via IS translocation.

Next, strains representing the various stages of the genome reduction process (MG1655, MDS12, MDS30, MDS42), and thus harboring various numbers of ISes (44, 25, 5, and 0, respectively) were transformed with pCTXVP60. The median growth curves observed for the transformants seemed to support the notion that the time needed for evolution of a non-toxic plasmid variant in the culture correlated with the number of ISes in the host genome (Fig. 6E). Even a single IS*1*, present in the host genome, had a marked effect on growth dynamics, and accelerated the evolution of fast-growing variants (Fig. 6F), as compared to the IS-less, essentially isogenic host (MDS42) (Fig. 6D).

### Bioinformatic analysis of *E. coli *IS sequences in shotgun sequencing data

To test whether IS-mediated instability of cloned sequences could be more wide-spread than anticipated, a bioinformatic analysis of raw data from early genome sequencing projects was performed. Protocols for shotgun genome sequencing by the Sanger method started by the insertion of fragments from a target genome into a plasmid vector, followed by transformation into *E. coli *cells. Next, transformed cells were grown first on solid medium, then in liquid cultures, prior to processing. Enriched target fragments were then sequenced, and overlapping sequence reads were assembled into contigs. The middle, cell growing steps can be viewed upon as some biological test of *E. coli *'s tolerance to the introduction of foreign DNA, consequently, read data from the thousands of clones can be mined for signs of the host's reaction to foreign DNA segments [32]. In this study we searched the read sets for the appearance of *E. coli *IS elements in raw (prior to their assembly into target genome sequences) sequence reads.

A typical bacterial shotgun library for Sanger sequencing would comprise 2-4 × 10^4 ^clones, which are grown for about 20 generations on solid medium, followed by another 10 generations of growth in liquid cultures, prior to processing. Considering the typical spontaneous, combined transposition rate of 10^-8^/gene/generation of resident ISes [9], the chances of sequencing a spontaneously arisen insertion mutant is about 1.2 × 10^-2 ^(=30 × 4 × 10^4 ^× 10^-8^). This means that approximately one in 100 sequencing projects would yield a gene interrupted by an IS of the cloning host. Higher incidence of host ISes in the sequence reads would likely indicate selection events preferring the IS-inserted clones during cultivation.

**Figure 7:**
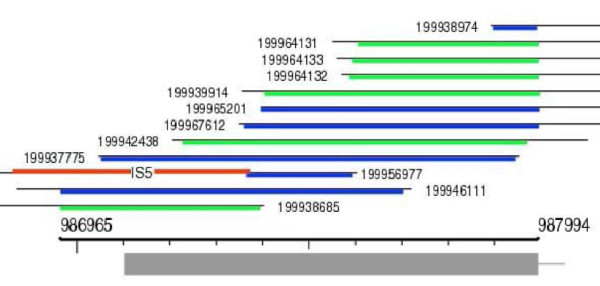
**Example of a typical IS insertion in shotgun sequence data found in sequencing reads**. Overlapping reads of *Streptococcus agalactiae *cover genome segment 986965..987994. Green (blue) lines show the extent of read alignment to the top (bottom) strand. Red line on read 199937775 shows the acquired IS*5*. Gray shaded bar below the axis represents the gene for an ABC transporter.

Data from 295 shotgun genome sequencing projects were downloaded and analyzed. The sets typically contain 50-70 thousand reads. Of the ~18 million reads, a total of 22 thousand match some *E. coli *IS sequence within the strict expectation, score, and run length thresholds. While 166 sets contain one or more reads with IS sequences, 129 sets are free of such reads. Of the 295 analyzed organisms 62 have complete (assembled) genome sequence. The number of organisms possessing both IS- containing reads and an assembled genome (intersection of the 166 set and the 62 set) is 30. In some IS-containing reads chromosomal sequence was not detectable, while in others IS elements seemed to have cloning vector origins (e.g., are spliced with *E. coli lacZ*), these have been weeded out. To avoid the difficulties of distinguishing true cloning related *E. coli *IS insertions from genomic self-rearrangements, organisms which have their own IS elements were excluded. After these filtering steps 14 shotgun sequencing sets remain, which possess a total of 109 IS-containing reads. IS*10 *and IS*1 *are the dominating invaders (they show up 68 and 30 times, respectively), but six other IS types were also found (IS*2*, IS*3*, IS*4*, IS*5*, IS*150*, IS*186*). Examples of IS elements appearing in sequence reads are shown in Fig. 7 and Additional file 5.

## Discussion

Synthetic biology is envisioned as the art of predictable assembly of off-the-shelf biological parts and devices with standard connections in a robust biological chassis (such as *E. coli*), using engineering principles [33,34]. To achieve such a goal, a major challenge to overcome is the intrinsic genetic instability of the living systems. Cells engineered to generate useful biological products almost invariably suffer a fitness loss, compared to wt. This can lead to selected accumulation of mutations, produced by the cell's mutation-generating mechanisms, inactivating the disabling, engineered parts, and compromising productivity. A possible solution to the problem is rendering certain metabolic pathways essential both for the desired product and for survival by interrupting all pathways that would allow the cellular biochemical network to operate with decreased production yields [35,36]. A complementing approach can be the reduction of the cell's evolutionary potential by genetic disabling of the mutation-generating mechanisms.

In *E. coli*, a significant part of the mutation spectrum is due to genomic rearrangements, caused by ISes. In an earlier study, we have shown that in unstressed cells, 20-25% of all mutations in an unselected gene (*cycA*) are due to IS hopping [24]. In stressed cells, the share of ISes in the total mutational load can even be higher. It was found that overexpression of CAT resulted in increased IS translocation frequency, manifested in a 4-fold elevated level of IS-mediated inactivation of *cycA*. The share of IS-generated mutations in adaptation can be critical in some settings. It was shown that genetic adaptation to salicin carbon source proceeded primarily via IS mutagenesis [4,6], and was significantly reduced in an IS-less host [24].

Developed for animal vaccine production, fusion gene *ctxvp60 *was inserted downstream of the vector's cloramphenicol resistance gene and transcribed from the *cam *promoter. This proved to be extremely unstable in ordinary IS-containing *E. coli *hosts. Almost invariably, clones with ISes in the 5' end of *ctxvp60 *were obtained from cells transformed with the plasmid. Analysis of the clones revealed that the toxic component of the clone was a Leu-rich peptide, the translated product of a secondary reading frame. Interestingly, *orf238 *coding for the Leu-rich peptide is an artificial gene, obtained unintentionally by designing and synthesizing a plant host-optimized variant of *ctxvp60*. The cause of instability lies in the dynamic process of stress, mutagenesis and selection. The translated product of *orf238 *interferes with host cell division and caused severe growth retardation. In this slow-growing population, random IS hopping, normally at frequencies ~10^-8^/gene/generation [9] but potentially elevated several-fold during stress [24], can produce clones with ISes interrupting sequences upstream of *orf238*. IS-inserted clones that stopped expressing ORF238, resumed normal growth, and rapidly became dominant in the culture. Insertions of IS*1 *and IS*3 *were found at multiple locations in both orientations, while IS5 was only seen at a single locus, corresponding to its integration hotspot "CWAR" [37], in opposite orientation only. Why is IS insertion the favored mode of inactivation of ORF238? We propose that ISes, due to their relatively large size and complex composition, are particularly effective at disrupting genes, either directly or indirectly. In a fast-growing pCTXVP60 mutant, we showed that IS*1 *acted by blocking transcription of *orf238*. Although in some cases insertion of ISes can generate outward-pointing transcription, expressing downstream genes [38], in this instance, apparently, it is the transcription-disrupting role that comes into play.

In principle, point-mutations (such as promoter mutations, active-site mutations, or frame-shifts) can also inactivate a gene. In fact, being generally more frequent than IS translocations, they might eventually be dominantly selected. In this particular case, however, point-mutations are rarely selected, because i) the promoter must be preserved to activate the selection marker, *cam*, and ii) single point-mutations are unlikely to eliminate the membrane-disrupting effect of the relatively large hydrophobic Leu-rich protein, iii) frame-shift mutations are presumably relatively ineffective, due to the presence of several potential in-frame re-initiation sites with AUG codons and ribosome-binding sites in ORF238 (Fig. 2). Deletions, another type of potentially effective, disruption mutation, are also rarely selected, presumably due to the absence of deletion-stimulating repeat sequences in pCTXVP60.

Sequence reads from shotgun sequencing libraries provide a convenient, large database for checking how widespread the emergence of ISes in cloned sequences is. We have calculated that, considering typical shotgun library construction/processing steps and background transposition rates of resident ISes of the cloning host, one would expect practically no host ISes to emerge in gene-sized shotgun sequence reads. However, the survey of a large set of shotgun sequence reads revealed quite a number of host ISes in the cloned segments. This elevated rate could be explained by processes analogous to those seen when cloning *ctxvp60*. In cases the cloned segment imposes stress on the host, individual clones grown for DNA preparation for sequencing can become dominated by IS-inserted variants, due to selection of the faster-growing mutants. This in turn could allow the IS elements of the cloning host to appear in the sequencing reads.

The very stringent criteria, by which we identify host ISes in shotgun sequence reads, probably underestimates the real number of IS transpositions. Furthermore, in addition to insertions, other sequence alterations (deletions and rearrangements) can also be induced by ISes. The occurrence and identification of IS-positive segments may depend on other factors as well, that are related to experimental procedures and to data collection protocols. Genes from taxonomically distant organisms may not get expressed in an *E. coli *host and would be less likely to cause growth retardation, thus, their IS-inserted mutants will not be subjects of selection. Information regarding the handling of clones and the construction of databases is sometimes unavailable, in other times incongruous (annotation is not uniform, same data types may refer to different content). It seems likely, that most datasets have probably undergone some computational pre-filtering, thereby IS hopping is masked in a fraction of the datasets. These considerations suggest, that *E. coli *IS-mediated events are not rare in cloning experiments, and underscore the significance of using IS-free MDS host in large-scale cloning projects.

The contribution of various IS elements to the detected transposition events do not necessarily reflect their relative transposition-activities. For example, despite the fact that IS*150 *was shown to be responsible for the majority of rearrangements over a 10,000-generation evolutionary experiment [39], as well as during repeated freeze-thaw cycles [40], its highly specific target site most probably does not allow its frequent occurrence in mutant-selection mechanisms described above. On the other hand, the promiscuous target selection of IS*1*, in addition to its high transposition-activity explains its high ratio among the insertion-mutants of *orf238*, *ebgR *[7], *bglR *[4], *cycA *[9], *tonB *[8] in evolutionary experiments [39,40] as well as in the output of our bioinformatic analysis. The frequent occurrence of IS*10 *could seem surprising, since it is not native to most K-12 strains. It was introduced accidentally in the 1970's through the use of Tn10 as a genetic transfer agent [41]. Considering our findings, together with its pronounced transposition activity described earlier [8,42-44], one might call it an invasive species of IS elements.

It is tempting to speculate that ISes, besides their parasitic and occasionally beneficial roles, could have a protective effect in natural settings (e.g., alleviating the negative consequences of horizontal gene transfer, HGT, on the host). However, this is an unlikely scenario: it does not seem economical to maintain a general, IS-based system (potentially deleterious itself) to alleviate harmful gene transfer events. Cells suffering a HGT event with negative consequences may simply become extinct, while the rest of the population would survive.

## Conclusions

Engineered genetic constructs, toxic for the *E. coli *K-12 host cell, may accumulate neutralizing, IS-mediated mutations, and the altered clones can be rapidly selected by their faster growth, leading to undesired genotypic and phenotypic changes. We show here that reducing the evolutionary potential of the host cell by removal of all genomic IS elements can result in significant stabilization of otherwise unstable clones. It is interesting to note, moreover, that reduced-genome MDS42 appears to possess a generally lowered propensity for recombination [45], further reducing the evolutionary potential of the strain.

Delaying the genetic adaptation in clean-genome MDS42, serving as host in SB applications, is suggested to be beneficial in both laboratory and industrial settings.

## Competing interests

FRB has a financial interest in Scarab Genomics LLC.

## Authors' contributions

KU carried out most cloning, transformation and overexpression experiments, as well as growth measurements. TF participated in cloning, strain development, database and literature searches, acquisition of funding and completion of the manuscript. GB performed DNA and protein sequence analysis of ctxvp60, database searches and growth rate and protein overexpression studies. FA carried out all the experiments concerning imaging. JP was responsible for the bioinformatic analysis of raw sequencing data. FRB provided intellectual help, as well as critical reading and correction of the manuscript. GP conceived the research, obtained funding, coordinated the experiments and drafted the manuscript. All authors have read and approved the final version of the manuscript.

## Supplementary Material

Additional file 1 Map and sequences of pCTXVP60, pCTXVP60frameshift, pSG1144-*orf238*, pSG1144-*ctxvp60*, pSG1144-*ctxvp60opt*, pSG1144-*ctxvp60dezopt*. Click here for file

Additional file 2 Translation and codon usage of *ctxvp60opt *gene. Click here for file

Additional file 3 Translation and codon usage of *ctxvp60dezopt *gene. Click here for file

Additional file 4 Structure predicitions for ORF238. Click here for file

Additional file 5 Relationship of reads, IS elements, genome segments and gene annotations. Click here for file
