# Supplementary material for: Reduced evolvability of Escherichia coli MDS42, an IS-less cellular chassis for molecular and synthetic biology applications
Source: Microb Cell Fact. 2010 May 21;9:38. doi: 10.1186/1475-2859-9-38 (PMC2891674; doi:10.1186/1475-2859-9-38)
Supplement: Additional file 1 — Map and sequences of pCTXVP60, pCTXVP60frameshift, pSG1144-orf238, pSG1144-ctxvp60, pSG1144-ctxvp60opt, pSG1144-ctxvp60dezopt. [file 1475-2859-9-38-S1.PDF]

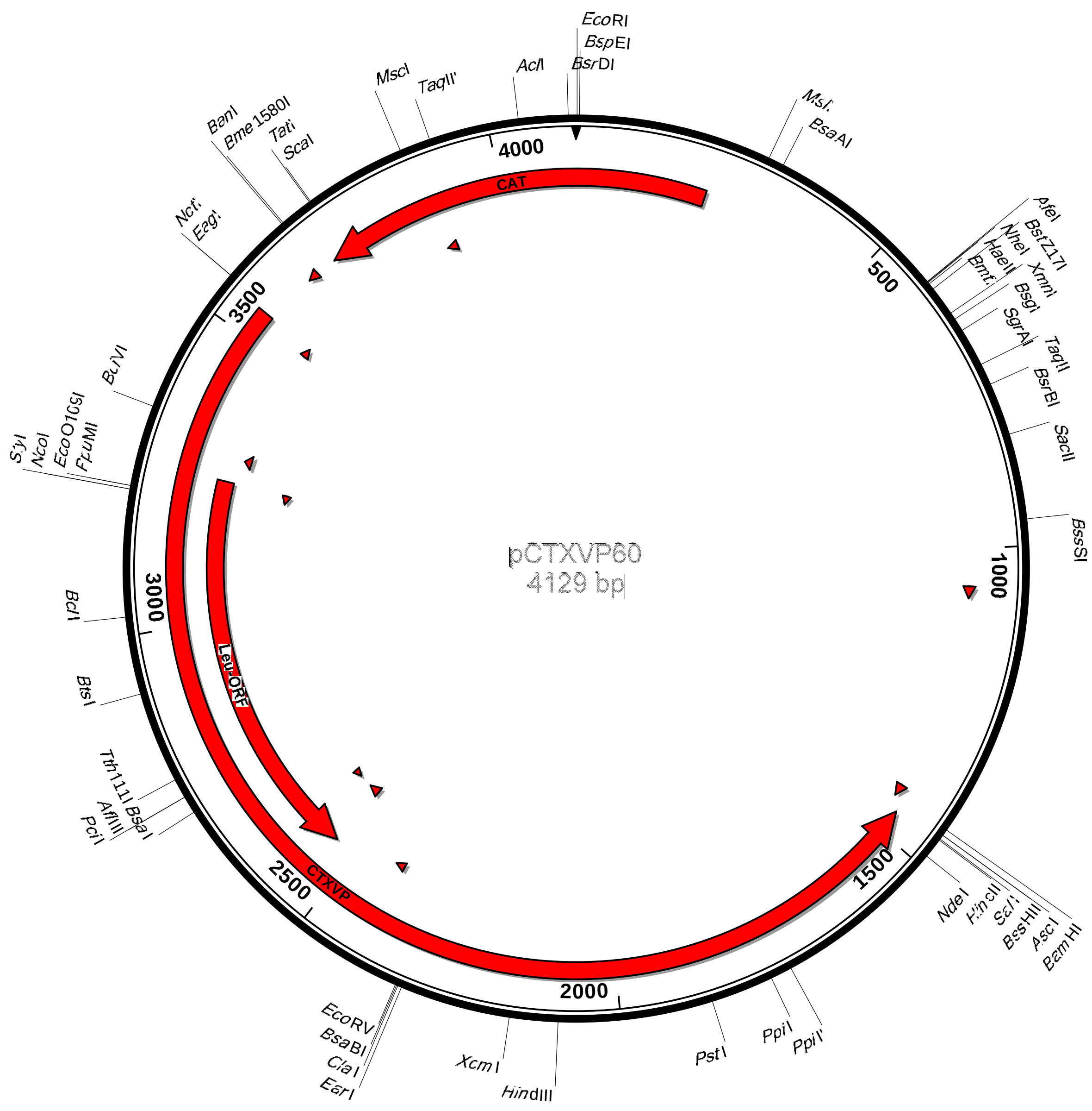

pCTXVP60:

ctxvp60: complement 1459-3549

orf238: complement 2541-3257

Cm^R: complement 3692-219 (wrapping)

GAATTCCGGATGAGCATTCATCAGGCGGGCAAGAATGTGAATAAAGGCCGGATAAA
ACTTGTGCTTATTTTTCTTTACGGTCTTTAAAAAGGCCGTAATATCCAGCTGAACGGT
CTGGTTATAGGTACATTGAGCAACTGACTGAAATGCCTCAAAATGTTCTTTACGATG
CCATTGGGATATATCAACGGTGGTATATCCAGTGATTTTTTTCTCCATTTTAGCTTCCT
TAGCTCCTGAAAATCTCGATAACTCAAAAAATACGCCCGGTAGTGATCTTATTTCATT
ATGGTGAAAGTTGGAACCTCTTACGTGCCGATCAACGTCTCATTTTCGCCAAAAGTT
GGCCCAGGGCTTCCCGGTATCAACAGGGACACCAGGATTTATTTATTCTGCGAAGTG
ATCTTCCGTCACAGGTATTTATTCGGCGCAAAGTGCGTCGGGTGATGCTGCCAACTT
ACTGATTTAGTGTATGATGGTGTTTTTGAGGTGCTCCAGTGGCTTCTGTTTCTATCAG
CTGTCCCTCCTGTTCAGCTACTGACGGGGTGGTGCGTAACGGCAAAAGCACCGCCGG
ACATCAGCGCTAGCGGAGTGTATACTGGCTTACTATGTTGGCACTGATGAGGGTGTC
AGTGAAGTGCTTCATGTGGCAGGAGAAAAAAGGCTGCACCGGTGCGTCAGCAGAAT
ATGTGATACAGGATATATTCCGCTTCCTCGCTCACTGACTCGCTACGCTCGGTCGTTC
GACTGCGGCGAGCGGAAATGGCTTACGAACGGGGCGGAGATTTCCTGGAAGATGCC
AGGAAGATACTTAACAGGGAAGTGAGAGGGCCGCGGCAAAGCCGTTTTTCCATAGG
CTCCGCCCCCCTGACAAGCATCACGAAATCTGACGCTCAAATCAGTGGTGGCGAAAC
CCGACAGGACTATAAAGATACCAGGCGTTTCCCCCTGGCGGCTCCCTCGTGCGCTCT
CCTGTTCCTGCCTTTCGGTTTACCGGTGTCATTCCGCTGTTATGGCCGCGTTTGTCTCA
TTCCACGCCTGACACTCAGTTCCGGGTAGGCAGTTCGCTCCAAGCTGGACTGTATGC
ACGAACCCCCCGTTCAGTCCGACCGCTGCGCCTTATCCGGTAACTATCGTCTTGAGTC
CAACCCGGAAAGACATGCAAAAGCACCACTGGCAGCAGCCACTGGTAATTGATTTA
GAGGAGTTAGTCTTGAAGTCATGCGCCGGTTAAGGCTAAACTGAAAGGACAAGTTTT
GGTGACTGCGCTCCTCCAAGCCAGTTACCTCGGTTCAAAGAGTTGGTAGCTCAGAGA
ACCTTCGAAAAACCGCCCTGCAAGGCGGTTTTTTCGTTTTCAGAGCAAGAGATTACG
CGCAGACCAAAACGATCTCAAGAAGATCATCTTATTAGGATCTATTAATACGACTCA
CTATAGGGATCCCGGGCGCGCCGTCGACTTTAAAGTTCATCCTTTTCAGAAACATAT
GAAAATCCATTAGCAGTTCCTCCAAGATTAAAAACAAGAGTAGACTTAGATGGTCTT
GGTCCAACTGGTCTAACATCAATAAGTTCAGTAAGATCAATAAGAGTAGTAGAAGCT
CCAGTTCCAGCATAAAAATATCCATCAACAGAAAGTCCAATTTCCATGAATCCAGAA
GCAAAAGTAAGTTGCCAAACAAAAAATTGTCCTGGCATAAGAGCAGAAGAATAATT
ATTAAGAGAAAGTCCAATAGTAACTGGAAGTGGTTGAGATCCAGTTCCATATTGAGT
TCCATTAGCAGATCCAGCAGTAGCATTAACATCTCCAGTTCTTCTAACAACAGAAGC
AAACATAATTGGAGTATTCTTTCCAACTGGAGCTGCAGCTGGAGTTCCTGGAGTAGT
AACAATTCTATCTGGTTGTGGAGTATAAGTAATAGCATTAGCAGATGGAGTAGAAAT
AACTCCAGAAGCCATAACAAAAAGTCCAGCTGGATTTTGAGCAGTTCCAGTAACAAC
AGCATAGATAGACTTAGCAACAGTTTGAGATCCAGAAGTATTAGTAGTTGGTTGAAG
ATTTCCTGGAGCTCCAGTAGCAAATCCAAGTTCATAAGCTTGAACAGTAGTAACATT
TGGAGCTCCAGAATTAGAGTTCCAAATAGCTCCAAATCCAACCCATCCAGCAGCTGG
AATACCTGGTCCATTGAATGGAACAAAAGACATATCTGGAAATCCATCTGGAGCAAC
TTGAGAAATTGGATTATCAATAGCAGATCCAGCATTAGCATACCAAAATTGAAGAAC
ATTAGTAGCATTAGATCCTGGATAAGAAGCAGATCCTCTTCTATGATCGATATCAGC
AAATCTTGGAGAAGACCATCCATAAGTAGATCCATTAAGATTCCAATGTCTATTACA
AGTAGAAAATCCTCCTGGAACTGGTTGAAGTCCAACAATTTGTCCATTCCATCTATTA
TCATTTCCAACTCCAGTAAGAACTGGAGTAGTAAGAAGTCCAGCTGGATAGATAGAA
TCAACAGTCTTAGAAGATGGAGTTCTAATCATAACAAATTCAAAATCTTCAGATGGT
CTAGTTTCAACAGTAACTTGAATAGCAGAAGTAGATCCTCCAAATGGATTAATAAGA
TTATTATAAACAGAAAGAACAAGAGTTGGAACAAGTCCTGGATCTCCAGTTGGATGA

TACATATTTGGTCTAAGGTCTGGCATAGTAATAGTAACTGGTTCAAGAGACCTAGCA
TCAATAACAACATGTGGAAATTGTCTAACTTCAAGACCTGGTCCAATTTCAATTCCTG
GTGGAATAACAGCAGCAACAAGTCTTCCTCCAAAAACTCCAGATCCAGCAACAATG
AATCTGAATTGCATTCCTCCAGCCCATCCAGCATACATTTGAGAAAGAACAGCAGTG
AATGGATTATTTTGTGGAGAATGTTGAACAGTATAAAGAATAGATCCTGGAGCATCA
GCAACAGACCAAGTAAAAACATCATTATAATAAAAATTAGTTCTCCAAGTTTCTTGT
TGATCAACTTGTTGTGGTGGTCCTCCAATTCCAGCAGTAGCAATAGAAGCAGAAGAA
TTTTCAGCAGTAACAACAGAAGTAGTAGCAACAACTCCTGGGTCCATTCCATCAGTA
GTAGTTCCTGGAACAGAAGCAGTAGTAGCAGTTCCAGCAGCTCCAGCTTGTGGAGCA
GTTCTAGCCTTTCCTTCCATGGGTCCTGGTCCATTAGCCATAGAAATAGCAGCAATAG
CATGTGGAGTCTTATTATTCCAAACACACAACTTTTCAACCTTAGCTTCAGTAAGATA
AGCAATTCTAAGAGTATCCTTCATTCTTTCAATAGCCTTCTTTTGAGAATCAATATGT
TGAGATCCTGGAACTTCAACTTGAAAAGTAGCTCCATTCTTAAAAGTAATAATAGCC
ATTTCTCTCTTTCCAGCAAGAGATTCAGTATAAGAAAAAATCTTATCATTAAGAGTAT
GAATTTGAGTATTATGATATTCAGCACAAAGATCAGTAATATTTTGTGGAGTGGAGG
AAGCCATTGTCCCGGGCGGCCGCTTCTATAGTGTCACCTAAATCGGGTCGAATTTGC
TTTCGAATTTCTGCCATTCATCCGCTTATTATCACTTATTCAGGCGTACAACCAGGCG
TTTAAGGGCACCAATAACTGCCTTAAAAAAATTACGCCCCGCCCTGCCACTCATCGC
AGTACTGTTGTAATTCATTAAGCATTCTGCCGACATGGAAGCCATCACAAACGGCAT
GATGAACCTGAATCGCCAGCGGCATCAGCACCTTGTCGCCTTGCGTATAATATTTGC
CCATAGTGAAAACGGGGGCGAAGAAGTTGTCCATATTGGCCACGTTTAAATCAAAA
CTGGTGAAACTCACCCAGGGATTGGCTGAGACGAAAAACATATTCTCAATAAACCCT
TTAGGGAAATAGGCCAGGTTTTCACCGTAACACGCCACATCTTGCGAATATATGTGT
AGAAACTGCCGGAAATCGTCGTGGTATTCACTCCAGAGCGATGAAAACGTTTCAGTT
TGCTCATGGAAAACGGTGTAACAAGGGTGAACACTATCCCATATCACCAGCTCACCG
TCTTTCATTGCCATACG

pCTXVP60frameshift:
extra T at 3128
ctxvp60truncated: complement 3086-3550
orf238truncated: complement 2541-3134
Cm^{R}: complement 3693-219 (wrapping)
GAATTCCGGATGAGCATTCATCAGGCGGGCAAGAATGTGAATAAAGGCCGGATAAA
ACTTGTGCTTATTTTTCTTTACGGTCTTTAAAAAGGCCGTAATATCCAGCTGAACGGT
CTGGTTATAGGTACATTGAGCAACTGACTGAAATGCCTCAAAATGTTCTTTACGATG
CCATTGGGATATATCAACGGTGGTATATCCAGTGATTTTTTTCTCCATTTTAGCTTCCT
TAGCTCCTGAAAATCTCGATAACTCAAAAAATACGCCCGGTAGTGATCTTATTTCATT
ATGGTGAAAGTTGGAACCTCTTACGTGCCGATCAACGTCTCATTTTCGCCAAAAGTT
GGCCCAGGGCTTCCCGGTATCAACAGGGACACCAGGATTTATTTATTCTGCGAAGTG
ATCTTCCGTCACAGGTATTTATTCGGCGCAAAGTGCGTCGGGTGATGCTGCCAACTT
ACTGATTTAGTGTATGATGGTGTTTTTGAGGTGCTCCAGTGGCTTCTGTTTCTATCAG
CTGTCCCTCCTGTTCAGCTACTGACGGGGTGGTGCGTAACGGCAAAAGCACCGCCGG
ACATCAGCGCTAGCGGAGTGTATACTGGCTTACTATGTTGGCACTGATGAGGGTGTC
AGTGAAGTGCTTCATGTGGCAGGAGAAAAAAGGCTGCACCGGTGCGTCAGCAGAAT
ATGTGATACAGGATATATTCCGCTTCCTCGCTCACTGACTCGCTACGCTCGGTCGTTC

GACTGCGGCGAGCGGAAATGGCTTACGAACGGGGCGGAGATTTCCTGGAAGATGCC
AGGAAGATACTTAACAGGGAAGTGAGAGGGCCGCGGCAAAGCCGTTTTTCCATAGG
CTCCGCCCCCCTGACAAGCATCACGAAATCTGACGCTCAAATCAGTGGTGGCGAAAC
CCGACAGGACTATAAAGATACCAGGCGTTTCCCCCTGGCGGCTCCCTCGTGCGCTCT
CCTGTTCCTGCCTTTCGGTTTACCGGTGTCATTCCGCTGTTATGGCCGCGTTTGTCTCA
TTCCACGCCTGACACTCAGTTCCGGGTAGGCAGTTCGCTCCAAGCTGGACTGTATGC
ACGAACCCCCCGTTCAGTCCGACCGCTGCGCCTTATCCGGTAACTATCGTCTTGAGTC
CAACCCGGAAAGACATGCAAAAGCACCACTGGCAGCAGCCACTGGTAATTGATTTA
GAGGAGTTAGTCTTGAAGTCATGCGCCGGTTAAGGCTAAACTGAAAGGACAAGTTTT
GGTGACTGCGCTCCTCCAAGCCAGTTACCTCGGTTCAAAGAGTTGGTAGCTCAGAGA
ACCTTCGAAAAACCGCCCTGCAAGGCGGTTTTTTCGTTTTCAGAGCAAGAGATTACG
CGCAGACCAAAACGATCTCAAGAAGATCATCTTATTAGGATCTATTAATACGACTCA
CTATAGGGATCCCGGGCGCGCCGTCGACTTTAAAGTTCATCCTTTTCAGAAACATAT
GAAAATCCATTAGCAGTTCCTCCAAGATTAAAAACAAGAGTAGACTTAGATGGTCTT
GGTCCAACTGGTCTAACATCAATAAGTTCAGTAAGATCAATAAGAGTAGTAGAAGCT
CCAGTTCCAGCATAAAAATATCCATCAACAGAAAGTCCAATTTCCATGAATCCAGAA
GCAAAAGTAAGTTGCCAAACAAAAAATTGTCCTGGCATAAGAGCAGAAGAATAATT
ATTAAGAGAAAGTCCAATAGTAACTGGAAGTGGTTGAGATCCAGTTCCATATTGAGT
TCCATTAGCAGATCCAGCAGTAGCATTAACATCTCCAGTTCTTCTAACAACAGAAGC
AAACATAATTGGAGTATTCTTTCCAACTGGAGCTGCAGCTGGAGTTCCTGGAGTAGT
AACAATTCTATCTGGTTGTGGAGTATAAGTAATAGCATTAGCAGATGGAGTAGAAAT
AACTCCAGAAGCCATAACAAAAAGTCCAGCTGGATTTTGAGCAGTTCCAGTAACAAC
AGCATAGATAGACTTAGCAACAGTTTGAGATCCAGAAGTATTAGTAGTTGGTTGAAG
ATTTCCTGGAGCTCCAGTAGCAAATCCAAGTTCATAAGCTTGAACAGTAGTAACATT
TGGAGCTCCAGAATTAGAGTTCCAAATAGCTCCAAATCCAACCCATCCAGCAGCTGG
AATACCTGGTCCATTGAATGGAACAAAAGACATATCTGGAAATCCATCTGGAGCAAC
TTGAGAAATTGGATTATCAATAGCAGATCCAGCATTAGCATACCAAAATTGAAGAAC
ATTAGTAGCATTAGATCCTGGATAAGAAGCAGATCCTCTTCTATGATCGATATCAGC
AAATCTTGGAGAAGACCATCCATAAGTAGATCCATTAAGATTCCAATGTCTATTACA
AGTAGAAAATCCTCCTGGAACTGGTTGAAGTCCAACAATTTGTCCATTCCATCTATTA
TCATTTCCAACTCCAGTAAGAACTGGAGTAGTAAGAAGTCCAGCTGGATAGATAGAA
TCAACAGTCTTAGAAGATGGAGTTCTAATCATAACAAATTCAAAATCTTCAGATGGT
CTAGTTTCAACAGTAACTTGAATAGCAGAAGTAGATCCTCCAAATGGATTAATAAGA
TTATTATAAACAGAAAGAACAAGAGTTGGAACAAGTCCTGGATCTCCAGTTGGATGA
TACATATTTGGTCTAAGGTCTGGCATAGTAATAGTAACTGGTTCAAGAGACCTAGCA
TCAATAACAACATGTGGAAATTGTCTAACTTCAAGACCTGGTCCAATTTCAATTCCTG
GTGGAATAACAGCAGCAACAAGTCTTCCTCCAAAAACTCCAGATCCAGCAACAATG
AATCTGAATTGCATTCCTCCAGCCCATCCAGCATACATTTGAGAAAGAACAGCAGTG
AATGGATTATTTTGTGGAGAATGTTGAACAGTATAAAGAATAGATCCTGGAGCATCA
GCAACAGACCAAGTAAAAACATCATTATAATAAAAATTAGTTCTCCAAGTTTCTTGT
TGATCAACTTGTTGTGGTGGTCCTCCAATTCCAGCAGTAGCAATAGAAGCAGAAGAA
TTTTCAGCAGTAACAACAGAAGTAGTAGCAACAACTCCTGGGTCCtATTCCATCAGTA
GTAGTTCCTGGAACAGAAGCAGTAGTAGCAGTTCCAGCAGCTCCAGCTTGTGGAGCA
GTTCTAGCCTTTCCTTCCATGGGTCCTGGTCCATTAGCCATAGAAATAGCAGCAATAG
CATGTGGAGTCTTATTATTCCAAACACACAACTTTTCAACCTTAGCTTCAGTAAGATA
AGCAATTCTAAGAGTATCCTTCATTCTTTCAATAGCCTTCTTTTGAGAATCAATATGT
TGAGATCCTGGAACTTCAACTTGAAAAGTAGCTCCATTCTTAAAAGTAATAATAGCC

ATTTCTCTCTTTCCAGCAAGAGATTCAGTATAAGAAAAAATCTTATCATTAAGAGTAT
GAATTTGAGTATTATGATATTCAGCACAAAGATCAGTAATATTTTGTGGAGTGGAGG
AAGCCATTGTCCCGGGCGGCCGCTTCTATAGTGTCACCTAAATCGGGTCGAATTTGC
TTTCGAATTTCTGCCATTCATCCGCTTATTATCACTTATTCAGGCGTACAACCAGGCG
TTTAAGGGCACCAATAACTGCCTTAAAAAAATTACGCCCCGCCCTGCCACTCATCGC
AGTACTGTTGTAATTCATTAAGCATTCTGCCGACATGGAAGCCATCACAAACGGCAT
GATGAACCTGAATCGCCAGCGGCATCAGCACCTTGTCGCCTTGCGTATAATATTTGC
CCATAGTGAAAACGGGGGCGAAGAAGTTGTCCATATTGGCCACGTTTAAATCAAAA
CTGGTGAAACTCACCCAGGGATTGGCTGAGACGAAAAACATATTCTCAATAAACCCT
TTAGGGAAATAGGCCAGGTTTTCACCGTAACACGCCACATCTTGCGAATATATGTGT
AGAAACTGCCGGAAATCGTCGTGGTATTCACTCCAGAGCGATGAAAACGTTTCAGTT
TGCTCATGGAAAACGGTGTAACAAGGGTGAACACTATCCCATATCACCAGCTCACCG
TCTTTCATTGCCATACG

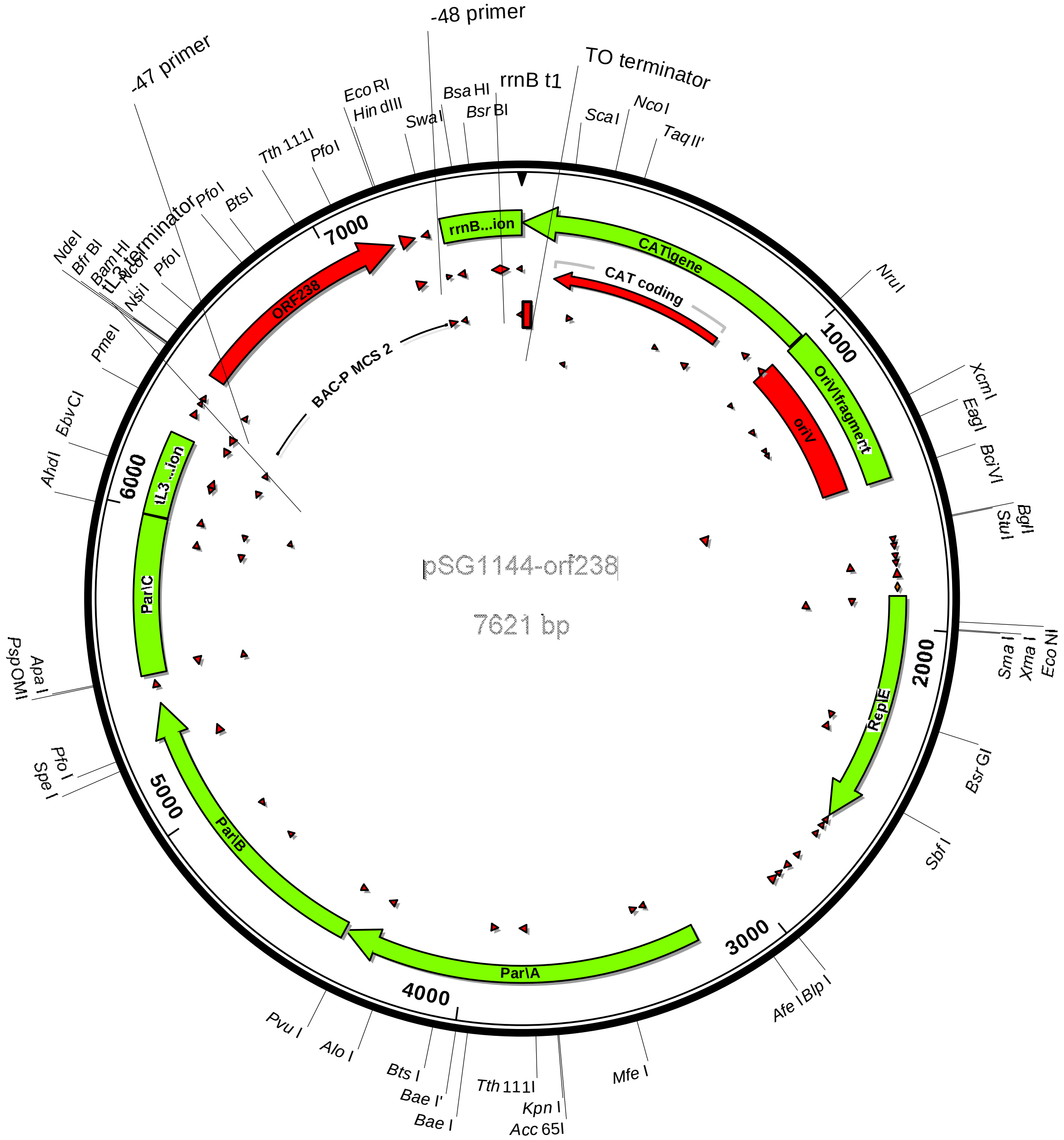

pSG1144-*orf238*

orf238 gene: 6470-7198

Cm^{R}: complement 2-990

GGTACAATAAAAAACGCCCGGCGGCAACCGAGCGTTCATTTCTGCCATTCATCCGCT
TATTATCACTTATTCAGGCGTAGCAACCAGGCGTTTAAGGGCACCAATAACTGCCTT
AAAAAAATTACGCCCCGCCCTGCCACTCATCGCAGTACTGTTGTAATTCATTAAGCA

TTCTGCCGACATGGAAGCCATCACAAACGGCATGATGAACCTGAATCGCCAGCGGC
ATCAGCACCTTGTCGCCTTGCGTATAATATTTGCCCATGGTGAAAACGGGGGCGAAG
AAGTTGTCCATATTGGCCACGTTTAAATCAAAACTGGTGAAACTCACCCAGGGATTG
GCTGAGACGAAAAACATATTCTCAATAAACCCTTTAGGGAAATAGGCCAGGTTTTCA
CCGTAACACGCCACATCTTGCGAATATATGTGTAGAAACTGCCGGAAATCGTCGTGG
TATTCACTCCAGAGCGATGAAAACGTTTCAGTTTGCTCATGGAAAACGGTGTAACAA
GGGTGAACACTATCCCATATCACCAGCTCACCGTCTTTCATTGCCATACGTAATTCCG
GATGAGCATTCATCAGGCGGGCAAGAATGTGAATAAAGGCCGGATAAAACTTGTGC
TTATTTTTCTTTACGGTCTTTAAAAAGGCCGTAATATCCAGTTGAACGGTCTGGTTAT
AGGTACATTGAGCAACTGACTGAAATGCCTCAAAATGTTCTTTACGATGCCATTGGG
ATATATCAACGGTGGTATATCCAGTGATTTTTTTCTCCATTTTAGCTTCCTTAGCTCCT
GAAAATCTCGATAACTCAAAAAATACGCCCGGTAGTGATCTTATTTCATTATGGTGA
AAGTTGGAACCTCTTACGTGCCGATCAACGTCTCATTTTCGCCAAAAGTTGGCCCAG
GGCTTCCCGGTATCAACAGGGACACCAGGATTTATTTATTCTGCGAAGTGATCTTCC
GTCACAGGTATTTATTCGCGATACAGGGTTCGAGAAGGGGGGGCACCCCCCTTCGGC
GTGCGCGGTCACGCGCACAGGGCGCAGCCCTGGTTAAAAACAAGGTTTATAAATATT
GGTTTAAAAGCAGGTTAAAAGACAGGTTAGCGGTGGCCGAAAAACGGGCGGAAACC
CTTGCAAATGCTGGATTTTCTGCCTGTGGACAGCCCCTCAAATGTCAATAGGTGCGC
CCCTCATCTGTCAGCACTCTGCCCCTCAAGTGTCAAGGATCGCGCCCCTCATCTGTCA
GTAGTCGCGCCCCTCAAGTGTCAATACCGCAGGGCACTTATCCCCAGGCTTGTCCAC
ATCATCTGTGGGAAACTCGCGTAAAATCAGGCGTTTTCGCCGATTTGCGAGGCTGGC
CAGCTCCACGTCGCCGGCCGAAATCGAGCCTGCCCCTCATCTGTAAACGCCGCGCCG
GGTGAGTCGGCCCCTCAAGTGTCAACGTCCGCCCCTCATCTGTCAGTGAGGGCCAAG
TTTTCCGCGAGGTATCCACAACGCCGGCGGTCGAGTGAGCGAGGAAGCACCAGGGA
ACAGCACTTATATATTCTGCTTACACACGATGCCTGAAAAAACTTCCCTTGGGGTTAT
CCACTTATCCACGGGGATATTTTTATAATTATTTTTTTTATAGTTTTTAGATCTTCTTT
TTTAGAGCGCCTTGTAGGCCTTTATCCATGCTGGTTCTAGAGAAGGTGTTGTGACAA
ATTGCCCTTTCAGTGTGACAAATCACCCTCAAATGACAGTCCTGTCTGTGACAAATTG
CCCTTAACCCTGTGACAAATTGCCCTCAGAAGAAGCTGTTTTTTCACAAAGTTATCCC
TGCTTATTGACTCTTTTTTATTTAGTGTGACAATCTAAAAACTTGTCACACTTCACAT
GGATCTGTCATGGCGGAAACAGCGGTTATCAATCACAAGAAACGTAAAAATAGCCC
GCGAATCGTCCAGTCAAACGACCTCACTGAGGCGGCATATAGTCTCTCCCGGGATCA
AAAACGTATGCTGTATCTGTTCGTTGACCAGATCAGAAAATCTGATGGCACCCTACA
GGAACATGACGGTATCTGCGAGATCCATGTTGCTAAATATGCTGAAATATTCGGATT
GACCTCTGCGGAAGCCAGTAAGGATATACGGCAGGCATTGAAGAGTTTCGCGGGGA
AGGAAGTGGTTTTTTATCGCCCTGAAGAGGATGCCGGCGATGAAAAAGGCTATGAAT
CTTTTCCTTGGTTTATCAAACGTGCGCACAGTCCATCCAGAGGGCTTTACAGTGTACA
TATCAACCCATATCTCATTCCCTTCTTTATCGGGTTACAGAACCGGTTTACGCAGTTT
CGGCTTAGTGAAACAAAAGAAATCACCAATCCGTATGCCATGCGTTTATACGAATCC
CTGTGTCAGTATCGTAAGCCGGATGGCTCAGGCATCGTCTCTCTGAAAATCGACTGG
ATCATAGAGCGTTACCAACTGCCTCAAAGTTACCAGCGTATGCCTGACTTCCGCCGC
CGCTTCCTGCAGGTCTGTGTTAATGAGATCAACAGCAGAACTCCAATGCGCCTCTCA
TACATTGAGAAAAAGAAAGGCCGCCAGACGACTCATATCGTATTTTCCTTCCGCGAT
ATCACTTCCATGACGACAGGATAGTCTGAGGGTTATCTGTCACAGATTTGAGGGTGG
TTCGTCACATTTGTTCTGACCTACTGAGGGTAATTTGTCACAGTTTTGCTGTTTCCTTC
AGCCTGCATGGATTTTCTCATACTTTTTGAACTGTAATTTTTAAGGAAGCCAAATTTG
AGGGCAGTTTGTCACAGTTGATTTCCTTCTCTTTCCCTTCGTCATGTGACCTGATATC

GGGGGTTAGTTCGTCATCATTGATGAGGGTTGATTATCACAGTTTATTACTCTGAATT
GGCTATCCGCGTGTGTACCTCTACCTGGAGTTTTTCCCACGGTGGATATTTCTTCTTG
CGCTGAGCGTAAGAGCTATCTGACAGAACAGTTCTTCTTTGCTTCCTCGCCAGTTCGC
TCGCTATGCTCGGTTACACGGCTGCGGCGAGCGCTAGTGATAATAAGTGACTGAGGT
ATGTGCTCTTCTTATCTCCTTTTGTAGTGTTGCTCTTATTTTAAACAACTTTGCGGTTT
TTTGATGACTTTGCGATTTTGTTGTTGCTTTGCAGTAAATTGCAAGATTTAATAAAAA
AACGCAAAGCAATGATTAAAGGATGTTCAGAATGAAACTCATGGAAACACTTAACC
AGTGCATAAACGCTGGTCATGAAATGACGAAGGCTATCGCCATTGCACAGTTTAATG
ATGACAGCCCGGAAGCGAGGAAAATAACCCGGCGCTGGAGAATAGGTGAAGCAGC
GGATTTAGTTGGGGTTTCTTCTCAGGCTATCAGAGATGCCGAGAAAGCAGGGCGACT
ACCGCACCCGGATATGGAAATTCGAGGACGGGTTGAGCAACGTGTTGGTTATACAAT
TGAACAAATTAATCACATGCGTGATGTGTTTGGTACGCGATTGCGACGTGCTGAAGA
CGTATTTCCACCGGTGATCGGGGTTGCTGCGCATAAAGGCGGCGTTTACAAAACCTC
AGTTTCTGTTCATCTTGCTCAGGATCTGGCTCTGAAGGGGCTACGTGTTTTGCTCGTG
GAAGGTAACGACCCCCAGGGAACAGCCTCAATGTATCACGGATGGGTACCAGATCT
TCATATTCATGCAGAAGACACTCTCCTGCCTTTCTATCTTGGGGAAAAGGACGATGT
CACTTATGCAATAAAGCCCACTTGCTGGCCGGGGCTTGACATTATTCCTTCCTGTCTG
GCTCTGCACCGTATTGAAACTGAGTTAATGGGCAAATTTGATGAAGGTAAACTGCCC
ACCGATCCACACCTGATGCTCCGACTGGCCATTGAAACTGTTGCTCATGACTATGAT
GTCATAGTTATTGACAGCGCGCCTAACCTGGGTATCGGCACGATTAATGTCGTATGT
GCTGCTGATGTGCTGATTGTTCCCACGCCTGCTGAGTTGTTTGACTACACCTCCGCAC
TGCAGTTTTTCGATATGCTTCGTGATCTGCTCAAGAACGTTGATCTTAAAGGGTTCGA
GCCTGATGTACGTATTTTGCTTACCAAATACAGCAATAGTAATGGCTCTCAGTCCCCG
TGGATGGAGGAGCAAATTCGGGATGCCTGGGGAAGCATGGTTCTAAAAAATGTTGT
ACGTGAAACGGATGAAGTTGGTAAAGGTCAGATCCGGATGAGAACTGTTTTTGAAC
AGGCCATTGATCAACGCTCTTCAACTGGTGCCTGGAGAAATGCTCTTTCTATTTGGGA
ACCTGTCTGCAATGAAATTTTCGATCGTCTGATTAAACCACGCTGGGAGATTAGATA
ATGAAGCGTGCGCCTGTTATTCCAAAACATACGCTCAATACTCAACCGGTTGAAGAT
ACTTCGTTATCGACCCCTGCTGCCCCGATGGTGGATTCGTTAATTGCGCGCGTAGGA
GTAATGGCTCGCGGTAATGCCATTACTTTGCCTGTATGTGGTCGGGATGTGAAGTTTA
CTCTTGAAGTGCTCCGGGGTGATAGTGTTGAGAAGACCTCTCGGGTATGGTCAGGTA
ATGAACGTGACCAGGAGCTGCTTACTGAGGACGCACTGGATGATCTCATCCCTTCTT
TTCTACTGACTGGTCAACAGACACCGGCGTTCGGTCGAAGAGTATCTGGTGTCATAG
AAATTGCCGATGGGAGTCGCCGTCGTAAAGCTGCTGCACTTACCGAAAGTGATTATC
GTGTTCTGGTTGGCGAGCTGGATGATGAGCAGATGGCTGCATTATCCAGATTGGGTA
ACGATTATCGCCCAACAAGTGCTTATGAACGTGGTCAGCGTTATGCAAACCGATTGC
AGAATGAATTTGCTGGAAATATTTCTGCGCTGGCTGATGCGGAAAATATTTCACGTA
AGATTATTACCCGCTGTATCAACACCGCCAAATTGCCTAAATCAGTTGTTGCTCTTTT
TTCTCACCCCGGTGAACTATCTGCCCGGTCAGGTGATGCACTTCAAAAAGCCTTTAC
AGATAAAGAGGAATTACTTAAGCAGCAGGCATCTAACCTTCATGAGCAGAAAAAAG
CTGGGGTGATATTTGAAGCTGAAGAAGTTATCACTCTTTTAACTTCTGTGCTTAAAAC
GTCATCTGCATCAAGAACTAGTTTAAGCTCACGACATCAGTTTGCTCCTGGAGCGAC
AGTATTGTATAAGGGCGATAAAATGGTGCTTAACCTGGACAGGTCTCGTGTTCCAAC
TGAGTGTATAGAGAAAATTGAGGCCATTCTTAAGGAACTTGAAAAGCCAGCACCCTG
ATGCGACCACGTTTTAGTCTACGTTTATCTGTCTTTACTTAATGTCCTTTGTTACAGGC
CAGAAAGCATAACTGGCCTGAATATTCTCTCTGGGCCCACTGTTCCACTTGTATCGTC
GGTCTGATTATTAGTCTGGGACCAAGGTCCCACTCGTATCGTCGGTCTGATTATTAGT

CTGGGACCACGGTCCCACTCGTATCGTCGGTCTGATTATTAGTCTGGGACCACGGTC
CCACTCGTATCGTCGGTCTGATAATCAGACTGGGACCACGGTCCCACTCGTATCGTC
GGTCTGATTATTAGTCTGGGACCAAGGTCCCACTCGTATCGTCGGTCTGATTATTAGT
CTGGGACCACGGTCCCACTCGTATCGTCGGTCTGATTATTAGTCTGGGACCAAGGTC
CCACTCGTATCGTCGGTCTGATTATTAGTCTGGGACCACGGTCCCACTCGTATCGTCG
GTCTGATTATTAGTCTGGGACCACGATCCCACTCGTGTTGTCGGTCTGATTATCGGTC
TGGGACCACGGTCCCACTTGTATTGTCGATCAGACTATCAGCGTGAGACTACGATTC
CATCAATGCCTGTCAAGGGCAAGTATTGACACTTCGTCTGTTTCTACTGGTATTGGCA
CAAACCTGATTCCAATTTGAGCAAGGCTATGTGCCATCTCGATACTCGTTCTTAACTC
AACAGAAGATGCTTTGTGCATACAGCCCCTCGTTTATTATTTATCTCCTCAGCCAGCC
GCTGTGCTTTCAGTGGATTTCGGATAACAGAAAGGCCGGGAAATACCCAGCCTCGCT
TTGTAACGGAGTAGAGACGAAAGTGATTGCGCCTACCCGGATATTATCGTGAGGATG
CGTCATCGCCATTAATTCACTGATCAGTGATAAGCTGTCATCTATGTCGGGTGCGGA
GAAAGAGGTAATGAAATGGCAGTTTAAACCGCCAGGGTTTTCCCAGTCACGACCCTA
GAGTCGAGCCAGGCGCGGGGTTCGCCCCGCAATTAATACGACTCACTATAGGGAGA
CCACAACGGTTTCCCTCTAGAAATAATTTTGTTTAACTTTAAGAAGGAGATATACAT
ATGCATGGATCCATGCTATTGCTGCTATTTCTATGGCTAATGGACCAGGACCCATGG
AAGGAAAGGCTAGAACTGCTCCACAAGCTGGAGCTGCTGGAACTGCTACTACTGCTT
CTGTTCCAGGAACTACTACTGATGGAATGGACCCAGGAGTTGTTGCTACTACTTCTGT
TGTTACTGCTGAAAATTCTTCTGCTTCTATTGCTACTGCTGGAATTGGAGGACCACCA
CAACAAGTTGATCAACAAGAAACTTGGAGAACTAATTTTTATTATAATGATGTTTTT
ACTTGGTCTGTTGCTGATGCTCCAGGATCTATTCTTTATACTGTTCAACATTCTCCAC
AAAATAATCCATTCACTGCTGTTCTTTCTCAAATGTATGCTGGATGGGCTGGAGGAA
TGCAATTCAGATTCATTGTTGCTGGATCTGGAGTTTTTGGAGGAAGACTTGTTGCTGC
TGTTATTCCACCAGGAATTGAAATTGGACCAGGTCTTGAAGTTAGACAATTTCCACA
TGTTGTTATTGATGCTAGGTCTCTTGAACCAGTTACTATTACTATGCCAGACCTTAGA
CCAAATATGTATCATCCAACTGGAGATCCAGGACTTGTTCCAACTCTTGTTCTTTCTG
TTTATAATAATCTTATTAATCCATTTGGAGGATCTACTTCTGCTATTCAAGTTACTGTT
GAAACTAGACCATCTGAAGATTTTGAATTTGTTATGAGAATTCAAGCTTAATTAGCT
GATAACTAGCATAACCCCTTGGGGCCTCTAAACGGGTCTTGAGGGGTTTTTTGCTGA
AAGGAGCTCGACCTGCAATCCTGTGTGAAATTGTTATCCGCTATTTAAATATTACCCT
GTTATCCCTACAGCTTGGCACTGGCCACGCAAAAAGGCCATCCGTCAGGATGGCCTT
CTGCTTAATTTGATGCCTGGCAGTTTATGGCGGGCGTCCTGCCCGCCACCCTCCGGGC
CGTTGCTTCGCAACGTTCAAATCCGCTCCCGGCGGATTTGTCCTACTCAGGAGAGCG
TTCACCGACAAACAACAGATAAAACGAAAGGCCCAGTCTTTCGACTGAGCCTTTCGT
TTTATTTGATGCCTGGCAGTTCCCTACTCTCGCATGGGGAGACCCCACACTACCATCG
G

pSG1144-*ctxvp60*
ctxvp60 gene: 6484-8574
Cm^R: complement 2-990
GGTACAATAAAAAACGCCCGGCGGCAACCGAGCGTTCATTTCTGCCATTCATCCGCT
TATTATCACTTATTCAGGCGTAGCAACCAGGCGTTTAAGGGCACCAATAACTGCCTT
AAAAAAATTACGCCCCGCCCTGCCACTCATCGCAGTACTGTTGTAATTCATTAAGCA
TTCTGCCGACATGGAAGCCATCACAAACGGCATGATGAACCTGAATCGCCAGCGGC
ATCAGCACCTTGTCGCCTTGCGTATAATATTTGCCCATGGTGAAAACGGGGGCGAAG

AAGTTGTCCATATTGGCCACGTTTAAATCAAAACTGGTGAAACTCACCCAGGGATTG
GCTGAGACGAAAAACATATTCTCAATAAACCCTTTAGGGAAATAGGCCAGGTTTTCA
CCGTAACACGCCACATCTTGCGAATATATGTGTAGAAACTGCCGGAAATCGTCGTGG
TATTCACTCCAGAGCGATGAAAACGTTTCAGTTTGCTCATGGAAAACGGTGTAACAA
GGGTGAACACTATCCCATATCACCAGCTCACCGTCTTTCATTGCCATACGTAATTCCG
GATGAGCATTCATCAGGCGGGCAAGAATGTGAATAAAGGCCGGATAAAACTTGTGC
TTATTTTTCTTTACGGTCTTTAAAAAGGCCGTAATATCCAGTTGAACGGTCTGGTTAT
AGGTACATTGAGCAACTGACTGAAATGCCTCAAAATGTTCTTTACGATGCCATTGGG
ATATATCAACGGTGGTATATCCAGTGATTTTTTTCTCCATTTTAGCTTCCTTAGCTCCT
GAAAATCTCGATAACTCAAAAAATACGCCCGGTAGTGATCTTATTTCATTATGGTGA
AAGTTGGAACCTCTTACGTGCCGATCAACGTCTCATTTTCGCCAAAAGTTGGCCCAG
GGCTTCCCGGTATCAACAGGGACACCAGGATTTATTTATTCTGCGAAGTGATCTTCC
GTCACAGGTATTTATTCGCGATACAGGGTTCGAGAAGGGGGGCACCCCCCTTCGGC
GTGCGCGGTCACGCGCACAGGGCGCAGCCCTGGTTAAAAACAAGGTTTATAAATATT
GGTTTAAAAGCAGGTTAAAAGACAGGTTAGCGGTGGCCGAAAAACGGGCGGAAACC
CTTGCAAATGCTGGATTTTCTGCCTGTGGACAGCCCCTCAAATGTCAATAGGTGCGC
CCCTCATCTGTCAGCACTCTGCCCCTCAAGTGTCAAGGATCGCGCCCCTCATCTGTCA
GTAGTCGCGCCCCTCAAGTGTCAATACCGCAGGGCACTTATCCCCAGGCTTGTCCAC
ATCATCTGTGGGAAACTCGCGTAAAATCAGGCGTTTTCGCCGATTTGCGAGGCTGGC
CAGCTCCACGTCGCCGGCCGAAATCGAGCCTGCCCCTCATCTGTAAACGCCGCGCCG
GGTGAGTCGGCCCCTCAAGTGTCAACGTCCGCCCCTCATCTGTCAGTGAGGGCCAAG
TTTTCCGCGAGGTATCCACAACGCCGGCGGTCGAGTGAGCGAGGAAGCACCAGGGA
ACAGCACTTATATATTCTGCTTACACACGATGCCTGAAAAAACTTCCCTTGGGGTTAT
CCACTTATCCACGGGGATATTTTTATAATTATTTTTTTTATAGTTTTTAGATCTTCTTT
TTTAGAGCGCCTTGTAGGCCTTTATCCATGCTGGTTCTAGAGAAGGTGTTGTGACAA
ATTGCCCTTTCAGTGTGACAAATCACCCTCAAATGACAGTCCTGTCTGTGACAAATTG
CCCTTAACCCTGTGACAAATTGCCCTCAGAAGAAGCTGTTTTTTCACAAAGTTATCCC
TGCTTATTGACTCTTTTTTATTTAGTGTGACAATCTAAAAACTTGTCACACTTCACAT
GGATCTGTCATGGCGGAAACAGCGGTTATCAATCACAAGAAACGTAAAAATAGCCC
GCGAATCGTCCAGTCAAACGACCTCACTGAGGCGGCATATAGTCTCTCCCGGGATCA
AAAACGTATGCTGTATCTGTTCGTTGACCAGATCAGAAAATCTGATGGCACCCTACA
GGAACATGACGGTATCTGCGAGATCCATGTTGCTAAATATGCTGAAATATTCGGATT
GACCTCTGCGGAAGCCAGTAAGGATATACGGCAGGCATTGAAGAGTTTCGCGGGGA
AGGAAGTGGTTTTTTATCGCCCTGAAGAGGATGCCGGCGATGAAAAAGGCTATGAAT
CTTTTCCTTGGTTTATCAAACGTGCGCACAGTCCATCCAGAGGGCTTTACAGTGTACA
TATCAACCCATATCTCATTCCCTTCTTTATCGGGTTACAGAACCGGTTTACGCAGTTT
CGGCTTAGTGAAACAAAAGAAATCACCAATCCGTATGCCATGCGTTTATACGAATCC
CTGTGTCAGTATCGTAAGCCGGATGGCTCAGGCATCGTCTCTCTGAAAATCGACTGG
ATCATAGAGCGTTACCAACTGCCTCAAAGTTACCAGCGTATGCCTGACTTCCGCCGC
CGCTTCCTGCAGGTCTGTGTTAATGAGATCAACAGCAGAACTCCAATGCGCCTCTCA
TACATTGAGAAAAAGAAAGGCCGCCAGACGACTCATATCGTATTTTCCTTCCGCGAT
ATCACTTCCATGACGACAGGATAGTCTGAGGGTTATCTGTCACAGATTTGAGGGTGG
TTCGTCACATTTGTTCTGACCTACTGAGGGTAATTTGTCACAGTTTTGCTGTTTCCTTC
AGCCTGCATGGATTTTCTCATACTTTTTGAACTGTAATTTTTAAGGAAGCCAAATTTG
AGGGCAGTTTGTCACAGTTGATTTCCTTCTCTTTCCCTTCGTCATGTGACCTGATATC
GGGGGTTAGTTCGTCATCATTGATGAGGGTTGATTATCACAGTTTATTACTCTGAATT
GGCTATCCGCGTGTGTACCTCTACCTGGAGTTTTTCCCACGGTGGATATTTCTTCTTG

CGCTGAGCGTAAGAGCTATCTGACAGAACAGTTCTTCTTTGCTTCCTCGCCAGTTCGC
TCGCTATGCTCGGTTACACGGCTGCGGCGAGCGCTAGTGATAATAAGTGACTGAGGT
ATGTGCTCTTCTTATCTCCTTTTGTAGTGTTGCTCTTATTTTAAACAACTTTGCGGTTT
TTTGATGACTTTGCGATTTTGTTGTTGCTTTGCAGTAAATTGCAAGATTTAATAAAAA
AACGCAAAGCAATGATTAAAGGATGTTCAGAATGAAACTCATGGAAACACTTAACC
AGTGCATAAACGCTGGTCATGAAATGACGAAGGCTATCGCCATTGCACAGTTTAATG
ATGACAGCCCGGAAGCGAGGAAAATAACCCGGCGCTGGAGAATAGGTGAAGCAGC
GGATTTAGTTGGGGTTTCTTCTCAGGCTATCAGAGATGCCGAGAAAGCAGGGCGACT
ACCGCACCCGGATATGGAAATTCGAGGACGGGTTGAGCAACGTGTTGGTTATACAAT
TGAACAAATTAATCACATGCGTGATGTGTTTGGTACGCGATTGCGACGTGCTGAAGA
CGTATTTCCACCGGTGATCGGGGTTGCTGCGCATAAAGGCGGCGTTTACAAAACCTC
AGTTTCTGTTCATCTTGCTCAGGATCTGGCTCTGAAGGGGCTACGTGTTTTGCTCGTG
GAAGGTAACGACCCCCAGGGAACAGCCTCAATGTATCACGGATGGGTACCAGATCT
TCATATTCATGCAGAAGACACTCTCCTGCCTTTCTATCTTGGGGAAAAGGACGATGT
CACTTATGCAATAAAGCCCACTTGCTGGCCGGGGCTTGACATTATTCCTTCCTGTCTG
GCTCTGCACCGTATTGAAACTGAGTTAATGGGCAAATTTGATGAAGGTAAACTGCCC
ACCGATCCACACCTGATGCTCCGACTGGCCATTGAAACTGTTGCTCATGACTATGAT
GTCATAGTTATTGACAGCGCGCCTAACCTGGGTATCGGCACGATTAATGTCGTATGT
GCTGCTGATGTGCTGATTGTTCCCACGCCTGCTGAGTTGTTTGACTACACCTCCGCAC
TGCAGTTTTTCGATATGCTTCGTGATCTGCTCAAGAACGTTGATCTTAAAGGGTTCGA
GCCTGATGTACGTATTTTGCTTACCAAATACAGCAATAGTAATGGCTCTCAGTCCCCG
TGGATGGAGGAGCAAATTCGGGATGCCTGGGGAAGCATGGTTCTAAAAAATGTTGT
ACGTGAAACGGATGAAGTTGGTAAAGGTCAGATCCGGATGAGAACTGTTTTTGAAC
AGGCCATTGATCAACGCTCTTCAACTGGTGCCTGGAGAAATGCTCTTTCTATTTGGGA
ACCTGTCTGCAATGAAATTTTCGATCGTCTGATTAAACCACGCTGGGAGATTAGATA
ATGAAGCGTGCGCCTGTTATTCCAAAACATACGCTCAATACTCAACCGGTTGAAGAT
ACTTCGTTATCGACCCCTGCTGCCCCGATGGTGGATTCGTTAATTGCGCGCGTAGGA
GTAATGGCTCGCGGTAATGCCATTACTTTGCCTGTATGTGGTCGGGATGTGAAGTTTA
CTCTTGAAGTGCTCCGGGGTGATAGTGTTGAGAAGACCTCTCGGGTATGGTCAGGTA
ATGAACGTGACCAGGAGCTGCTTACTGAGGACGCACTGGATGATCTCATCCCTTCTT
TTCTACTGACTGGTCAACAGACACCGGCGTTCGGTCGAAGAGTATCTGGTGTCATAG
AAATTGCCGATGGGAGTCGCCGTCGTAAAGCTGCTGCACTTACCGAAAGTGATTATC
GTGTTCTGGTTGGCGAGCTGGATGATGAGCAGATGGCTGCATTATCCAGATTGGGTA
ACGATTATCGCCCAACAAGTGCTTATGAACGTGGTCAGCGTTATGCAAACCGATTGC
AGAATGAATTTGCTGGAAATATTTCTGCGCTGGCTGATGCGGAAAATATTTCACGTA
AGATTATTACCCGCTGTATCAACACCGCCAAATTGCCTAAATCAGTTGTTGCTCTTTT
TTCTCACCCCGGTGAACTATCTGCCCGGTCAGGTGATGCACTTCAAAAAGCCTTTAC
AGATAAAGAGGAATTACTTAAGCAGCAGGCATCTAACCTTCATGAGCAGAAAAAAG
CTGGGGTGATATTTGAAGCTGAAGAAGTTATCACTCTTTTAACTTCTGTGCTTAAAAC
GTCATCTGCATCAAGAACTAGTTTAAGCTCACGACATCAGTTTGCTCCTGGAGCGAC
AGTATTGTATAAGGGCGATAAAATGGTGCTTAACCTGGACAGGTCTCGTGTTCCAAC
TGAGTGTATAGAGAAAATTGAGGCCATTCTTAAGGAACTTGAAAAGCCAGCACCCTG
ATGCGACCACGTTTTAGTCTACGTTTATCTGTCTTTACTTAATGTCCTTTGTTACAGGC
CAGAAAGCATAACTGGCCTGAATATTCTCTCTGGGCCCACTGTTCCACTTGTATCGTC
GGTCTGATTATTAGTCTGGGACCAAGGTCCCACTCGTATCGTCGGTCTGATTATTAGT
CTGGGACCACGGTCCCACTCGTATCGTCGGTCTGATTATTAGTCTGGGACCACGGTC
CCACTCGTATCGTCGGTCTGATAATCAGACTGGGACCACGGTCCCACTCGTATCGTC

GGTCTGATTATTAGTCTGGGACCAAGGTCCCACTCGTATCGTCGGTCTGATTATTAGT
CTGGGACCACGGTCCCACTCGTATCGTCGGTCTGATTATTAGTCTGGGACCAAGGTC
CCACTCGTATCGTCGGTCTGATTATTAGTCTGGGACCACGGTCCCACTCGTATCGTCG
GTCTGATTATTAGTCTGGGACCACGATCCCACTCGTGTTGTCGGTCTGATTATCGGTC
TGGGACCACGGTCCCACTTGTATTGTCGATCAGACTATCAGCGTGAGACTACGATTC
CATCAATGCCTGTCAAGGGCAAGTATTGACACTTCGTCTGTTTCTACTGGTATTGGCA
CAAACCTGATTCCAATTTGAGCAAGGCTATGTGCCATCTCGATACTCGTTCTTAACTC
AACAGAAGATGCTTTGTGCATACAGCCCCTCGTTTATTATTTATCTCCTCAGCCAGCC
GCTGTGCTTTCAGTGGATTTCGGATAACAGAAAGGCCGGGAAATACCCAGCCTCGCT
TTGTAACGGAGTAGAGACGAAAGTGATTGCGCCTACCCGGATATTATCGTGAGGATG
CGTCATCGCCATTAATTCACTGATCAGTGATAAGCTGTCATCTATGTCGGGTGCGGA
GAAAGAGGTAATGAAATGGCAGTTTAAACCGCCAGGGTTTTCCCAGTCACGACCCTA
GAGTCGAGCCAGGCGCGGGGTTCGCCCCGCAATTAATACGACTCACTATAGGGAGA
CCACAACGGTTTCCCTCTAGAAATAATTTTGTTTAACTTTAAGAAGGAGATATACAT
ATGCATGGATCCCAATGGCTTCCTCCACTCCACAAAATATTACTGATCTTTGTGCTGA
ATATCATAATACTCAAATTCATACTCTTAATGATAAGATTTTTTCTTATACTGAATCT
CTTGCTGGAAAGAGAGAAATGGCTATTATTACTTTTAAGAATGGAGCTACTTTTCAA
GTTGAAGTTCCAGGATCTCAACATATTGATTCTCAAAAGAAGGCTATTGAAAGAATG
AAGGATACTCTTAGAATTGCTTATCTTACTGAAGCTAAGGTTGAAAAGTTGTGTGTTT
GGAATAATAAGACTCCACATGCTATTGCTGCTATTTCTATGGCTAATGGACCAGGAC
CCATGGAAGGAAAGGCTAGAACTGCTCCACAAGCTGGAGCTGCTGGAACTGCTACT
ACTGCTTCTGTTCCAGGAACTACTACTGATGGAATGGACCCAGGAGTTGTTGCTACT
ACTTCTGTTGTTACTGCTGAAAATTCTTCTGCTTCTATTGCTACTGCTGGAATTGGAG
GACCACCACAACAAGTTGATCAACAAGAAACTTGGAGAACTAATTTTTATTATAATG
ATGTTTTTACTTGGTCTGTTGCTGATGCTCCAGGATCTATTCTTTATACTGTTCAACAT
TCTCCACAAAATAATCCATTCACTGCTGTTCTTTCTCAAATGTATGCTGGATGGGCTG
GAGGAATGCAATTCAGATTCATTGTTGCTGGATCTGGAGTTTTTGGAGGAAGACTTG
TTGCTGCTGTTATTCCACCAGGAATTGAAATTGGACCAGGTCTTGAAGTTAGACAAT
TTCCACATGTTGTTATTGATGCTAGGTCTCTTGAACCAGTTACTATTACTATGCCAGA
CCTTAGACCAAATATGTATCATCCAACTGGAGATCCAGGACTTGTTCCAACTCTTGTT
CTTTCTGTTTATAATAATCTTATTAATCCATTTGGAGGATCTACTTCTGCTATTCAAGT
TACTGTTGAAACTAGACCATCTGAAGATTTTGAATTTGTTATGATTAGAACTCCATCT
TCTAAGACTGTTGATTCTATCTATCCAGCTGGACTTCTTACTACTCCAGTTCTTACTG
GAGTTGGAAATGATAATAGATGGAATGGACAAATTGTTGGACTTCAACCAGTTCCAG
GAGGATTTTCTACTTGTAATAGACATTGGAATCTTAATGGATCTACTTATGGATGGTC
TTCTCCAAGATTTGCTGATATCGATCATAGAAGAGGATCTGCTTCTTATCCAGGATCT
AATGCTACTAATGTTCTTCAATTTTGGTATGCTAATGCTGGATCTGCTATTGATAATC
CAATTTCTCAAGTTGCTCCAGATGGATTTCCAGATATGTCTTTTGTTCCATTCAATGG
ACCAGGTATTCCAGCTGCTGGATGGGTTGGATTTGGAGCTATTTGGAACTCTAATTCT
GGAGCTCCAAATGTTACTACTGTTCAAGCTTATGAACTTGGATTTGCTACTGGAGCTC
CAGGAAATCTTCAACCAACTACTAATACTTCTGGATCTCAAACTGTTGCTAAGTCTAT
CTATGCTGTTGTTACTGGAACTGCTCAAAATCCAGCTGGACTTTTTGTTATGGCTTCT
GGAGTTATTTCTACTCCATCTGCTAATGCTATTACTTATACTCCACAACCAGATAGAA
TTGTTACTACTCCAGGAACTCCAGCTGCAGCTCCAGTTGGAAAGAATACTCCAATTA
TGTTTGCTTCTGTTGTTAGAAGAACTGGAGATGTTAATGCTACTGCTGGATCTGCTAA
TGGAACTCAATATGGAACTGGATCTCAACCACTTCCAGTTACTATTGGACTTTCTCTT
AATAATTATTCTTCTGCTCTTATGCCAGGACAATTTTTTGTTTGGCAACTTACTTTTGC

TTCTGGATTCATGGAAATTGGACTTTCTGTTGATGGATATTTTTATGCTGGAACTGGA
GCTTCTACTACTCTTATTGATCTTACTGAACTTATTGATGTTAGACCAGTTGGACCAA
GACCATCTAAGTCTACTCTTGTTTTTAATCTTGGAGGAACTGCTAATGGATTTTCATA
TGTTTCTGAAAAGGATGAACTTTAAAGTCGACGGAATTCAAGCTTAATTAGCTGATA
ACTAGCATAACCCCTTGGGGCCTCTAAACGGGTCTTGAGGGGTTTTTTGCTGAAAGG
AGCTCGACCTGCAATCCTGTGTGAAATTGTTATCCGCTATTTAAATATTACCCTGTTA
TCCCTACAGCTTGGCACTGGCCACGCAAAAAGGCCATCCGTCAGGATGGCCTTCTGC
TTAATTTGATGCCTGGCAGTTTATGGCGGGCGTCCTGCCCGCCACCCTCCGGGCCGTT
GCTTCGCAACGTTCAAATCCGCTCCCGGCGGATTTGTCCTACTCAGGAGAGCGTTCA
CCGACAAACAACAGATAAAACGAAAGGCCCAGTCTTTCGACTGAGCCTTTCGTTTTA
TTTGATGCCTGGCAGTTCCCTACTCTCGCATGGGGAGACCCCACACTACCATCGG

pSG1144-*ctxvp60opt*
ctxvp60 gene: 6470-8560
Cm^R: complement 2-990
GGTACAATAAAAAACGCCCGGCGGCAACCGAGCGTTCATTTCTGCCATTCATCCGCT
TATTATCACTTATTCAGGCGTAGCAACCAGGCGTTTAAGGGCACCAATAACTGCCTT
AAAAAAATTACGCCCCGCCCTGCCACTCATCGCAGTACTGTTGTAATTCATTAAGCA
TTCTGCCGACATGGAAGCCATCACAAACGGCATGATGAACCTGAATCGCCAGCGGC
ATCAGCACCTTGTCGCCTTGCGTATAATATTTGCCCATGGTGAAAACGGGGGCGAAG
AAGTTGTCCATATTGGCCACGTTTAAATCAAAACTGGTGAAACTCACCCAGGGATTG
GCTGAGACGAAAAACATATTCTCAATAAACCCTTTAGGGAAATAGGCCAGGTTTTCA
CCGTAACACGCCACATCTTGCGAATATATGTGTAGAAACTGCCGGAAATCGTCGTGG
TATTCACTCCAGAGCGATGAAAACGTTTCAGTTTGCTCATGGAAAACGGTGTAACAA
GGGTGAACACTATCCCATATCACCAGCTCACCGTCTTTCATTGCCATACGTAATTCCG
GATGAGCATTCATCAGGCGGGCAAGAATGTGAATAAAGGCCGGATAAAACTTGTGC
TTATTTTTCTTTACGGTCTTTAAAAAGGCCGTAATATCCAGTTGAACGGTCTGGTTAT
AGGTACATTGAGCAACTGACTGAAATGCCTCAAAATGTTCTTTACGATGCCATTGGG
ATATATCAACGGTGGTATATCCAGTGATTTTTTTCTCCATTTTAGCTTCCTTAGCTCCT
GAAAATCTCGATAACTCAAAAAATACGCCCGGTAGTGATCTTATTTCATTATGGTGA
AAGTTGGAACCTCTTACGTGCCGATCAACGTCTCATTTTCGCCAAAAGTTGGCCCAG
GGCTTCCCGGTATCAACAGGGACACCAGGATTTATTTATTCTGCGAAGTGATCTTCC
GTCACAGGTATTTATTCGCGATACAGGGTTCGAGAAGGGGGGGCACCCCCCTTCGGC
GTGCGCGGTCACGCGCACAGGGCGCAGCCCTGGTTAAAAACAAGGTTTATAAATATT
GGTTTAAAAGCAGGTTAAAAGACAGGTTAGCGGTGGCCGAAAAACGGGCGGAAACC
CTTGCAAATGCTGGATTTTCTGCCTGTGGACAGCCCCTCAAATGTCAATAGGTGCGC
CCCTCATCTGTCAGCACTCTGCCCCTCAAGTGTCAAGGATCGCGCCCCTCATCTGTCA
GTAGTCGCGCCCCTCAAGTGTCAATACCGCAGGGCACTTATCCCCAGGCTTGTCCAC
ATCATCTGTGGGAAACTCGCGTAAAATCAGGCGTTTTCGCCGATTTGCGAGGCTGGC
CAGCTCCACGTCGCCGGCCGAAATCGAGCCTGCCCCTCATCTGTAAACGCCGCGCCG
GGTGAGTCGGCCCCTCAAGTGTCAACGTCCGCCCCTCATCTGTCAGTGAGGGCCAAG
TTTTCCGCGAGGTATCCACAACGCCGGCGGTCGAGTGAGCGAGGAAGCACCAGGGA
ACAGCACTTATATATTCTGCTTACACACGATGCCTGAAAAAACTTCCCTTGGGGTTAT
CCACTTATCCACGGGGATATTTTTATAATTATTTTTTTTATAGTTTTTAGATCTTCTTT
TTTAGAGCGCCTTGTAGGCCTTTATCCATGCTGGTTCTAGAGAAGGTGTTGTGACAA
ATTGCCCTTTCAGTGTGACAAATCACCCTCAAATGACAGTCCTGTCTGTGACAAATTG
CCCTTAACCCTGTGACAAATTGCCCTCAGAAGAAGCTGTTTTTTCACAAAGTTATCCC

TGCTTATTGACTCTTTTTTATTTAGTGTGACAATCTAAAAACTTGTCACACTTCACAT
GGATCTGTCATGGCGGAAACAGCGGTTATCAATCACAAGAAACGTAAAAATAGCCC
GCGAATCGTCCAGTCAAACGACCTCACTGAGGCGGCATATAGTCTCTCCCGGGATCA
AAAACGTATGCTGTATCTGTTCGTTGACCAGATCAGAAAATCTGATGGCACCCTACA
GGAACATGACGGTATCTGCGAGATCCATGTTGCTAAATATGCTGAAATATTCGGATT
GACCTCTGCGGAAGCCAGTAAGGATATACGGCAGGCATTGAAGAGTTTCGCGGGGA
AGGAAGTGGTTTTTTATCGCCCTGAAGAGGATGCCGGCGATGAAAAAGGCTATGAAT
CTTTTCCTTGGTTTATCAAACGTGCGCACAGTCCATCCAGAGGGCTTTACAGTGTACA
TATCAACCCATATCTCATTCCCTTCTTTATCGGGTTACAGAACCGGTTTACGCAGTTT
CGGCTTAGTGAAACAAAAGAAATCACCAATCCGTATGCCATGCGTTTATACGAATCC
CTGTGTCAGTATCGTAAGCCGGATGGCTCAGGCATCGTCTCTCTGAAAATCGACTGG
ATCATAGAGCGTTACCAACTGCCTCAAAGTTACCAGCGTATGCCTGACTTCCGCCGC
CGCTTCCTGCAGGTCTGTGTTAATGAGATCAACAGCAGAACTCCAATGCGCCTCTCA
TACATTGAGAAAAAGAAAGGCCGCCAGACGACTCATATCGTATTTTCCTTCCGCGAT
ATCACTTCCATGACGACAGGATAGTCTGAGGGTTATCTGTCACAGATTTGAGGGTGG
TTCGTCACATTTGTTCTGACCTACTGAGGGTAATTTGTCACAGTTTTGCTGTTTCCTTC
AGCCTGCATGGATTTTCTCATACTTTTTGAACTGTAATTTTTAAGGAAGCCAAATTTG
AGGGCAGTTTGTCACAGTTGATTTCCTTCTCTTTCCCTTCGTCATGTGACCTGATATC
GGGGGTTAGTTCGTCATCATTGATGAGGGTTGATTATCACAGTTTATTACTCTGAATT
GGCTATCCGCGTGTGTACCTCTACCTGGAGTTTTTCCCACGGTGGATATTTCTTCTTG
CGCTGAGCGTAAGAGCTATCTGACAGAACAGTTCTTCTTTGCTTCCTCGCCAGTTCGC
TCGCTATGCTCGGTTACACGGCTGCGGCGAGCGCTAGTGATAATAAGTGACTGAGGT
ATGTGCTCTTCTTATCTCCTTTTGTAGTGTTGCTCTTATTTTAAACAACTTTGCGGTTT
TTTGATGACTTTGCGATTTTGTTGTTGCTTTGCAGTAAATTGCAAGATTTAATAAAAA
AACGCAAAGCAATGATTAAAGGATGTTCAGAATGAAACTCATGGAAACACTTAACC
AGTGCATAAACGCTGGTCATGAAATGACGAAGGCTATCGCCATTGCACAGTTTAATG
ATGACAGCCCGGAAGCGAGGAAAATAACCCGGCGCTGGAGAATAGGTGAAGCAGC
GGATTTAGTTGGGGTTTCTTCTCAGGCTATCAGAGATGCCGAGAAAGCAGGGCGACT
ACCGCACCCGGATATGGAAATTCGAGGACGGGTTGAGCAACGTGTTGGTTATACAAT
TGAACAAATTAATCACATGCGTGATGTGTTTGGTACGCGATTGCGACGTGCTGAAGA
CGTATTTCCACCGGTGATCGGGGTTGCTGCGCATAAAGGCGGCGTTTACAAAACCTC
AGTTTCTGTTCATCTTGCTCAGGATCTGGCTCTGAAGGGGCTACGTGTTTTGCTCGTG
GAAGGTAACGACCCCCAGGGAACAGCCTCAATGTATCACGGATGGGTACCAGATCT
TCATATTCATGCAGAAGACACTCTCCTGCCTTTCTATCTTGGGGAAAAGGACGATGT
CACTTATGCAATAAAGCCCACTTGCTGGCCGGGGCTTGACATTATTCCTTCCTGTCTG
GCTCTGCACCGTATTGAAACTGAGTTAATGGGCAAATTTGATGAAGGTAAACTGCCC
ACCGATCCACACCTGATGCTCCGACTGGCCATTGAAACTGTTGCTCATGACTATGAT
GTCATAGTTATTGACAGCGCGCCTAACCTGGGTATCGGCACGATTAATGTCGTATGT
GCTGCTGATGTGCTGATTGTTCCCACGCCTGCTGAGTTGTTTGACTACACCTCCGCAC
TGCAGTTTTTCGATATGCTTCGTGATCTGCTCAAGAACGTTGATCTTAAAGGGTTCGA
GCCTGATGTACGTATTTTGCTTACCAAATACAGCAATAGTAATGGCTCTCAGTCCCCG
TGGATGGAGGAGCAAATTCGGGATGCCTGGGGAAGCATGGTTCTAAAAAATGTTGT
ACGTGAAACGGATGAAGTTGGTAAAGGTCAGATCCGGATGAGAACTGTTTTTGAAC
AGGCCATTGATCAACGCTCTTCAACTGGTGCCTGGAGAAATGCTCTTTCTATTTGGGA
ACCTGTCTGCAATGAAATTTTCGATCGTCTGATTAAACCACGCTGGGAGATTAGATA
ATGAAGCGTGCGCCTGTTATTCCAAAACATACGCTCAATACTCAACCGGTTGAAGAT
ACTTCGTTATCGACCCCTGCTGCCCCGATGGTGGATTCGTTAATTGCGCGCGTAGGA

GTAATGGCTCGCGGTAATGCCATTACTTTGCCTGTATGTGGTCGGGATGTGAAGTTTA
CTCTTGAAGTGCTCCGGGGTGATAGTGTTGAGAAGACCTCTCGGGTATGGTCAGGTA
ATGAACGTGACCAGGAGCTGCTTACTGAGGACGCACTGGATGATCTCATCCCTTCTT
TTCTACTGACTGGTCAACAGACACCGGCGTTCGGTCGAAGAGTATCTGGTGTCATAG
AAATTGCCGATGGGAGTCGCCGTCGTAAAGCTGCTGCACTTACCGAAAGTGATTATC
GTGTTCTGGTTGGCGAGCTGGATGATGAGCAGATGGCTGCATTATCCAGATTGGGTA
ACGATTATCGCCCAACAAGTGCTTATGAACGTGGTCAGCGTTATGCAAACCGATTGC
AGAATGAATTTGCTGGAAATATTTCTGCGCTGGCTGATGCGGAAAATATTTCACGTA
AGATTATTACCCGCTGTATCAACACCGCCAAATTGCCTAAATCAGTTGTTGCTCTTTT
TTCTCACCCCGGTGAACTATCTGCCCGGTCAGGTGATGCACTTCAAAAAGCCTTTAC
AGATAAAGAGGAATTACTTAAGCAGCAGGCATCTAACCTTCATGAGCAGAAAAAAG
CTGGGGTGATATTTGAAGCTGAAGAAGTTATCACTCTTTTAACTTCTGTGCTTAAAAC
GTCATCTGCATCAAGAACTAGTTTAAGCTCACGACATCAGTTTGCTCCTGGAGCGAC
AGTATTGTATAAGGGCGATAAAATGGTGCTTAACCTGGACAGGTCTCGTGTTCCAAC
TGAGTGTATAGAGAAAATTGAGGCCATTCTTAAGGAACTTGAAAAGCCAGCACCCTG
ATGCGACCACGTTTTAGTCTACGTTTATCTGTCTTTACTTAATGTCCTTTGTTACAGGC
CAGAAAGCATAACTGGCCTGAATATTCTCTCTGGGCCCACTGTTCCACTTGTATCGTC
GGTCTGATTATTAGTCTGGGACCAAGGTCCCACTCGTATCGTCGGTCTGATTATTAGT
CTGGGACCACGGTCCCACTCGTATCGTCGGTCTGATTATTAGTCTGGGACCACGGTC
CCACTCGTATCGTCGGTCTGATAATCAGACTGGGACCACGGTCCCACTCGTATCGTC
GGTCTGATTATTAGTCTGGGACCAAGGTCCCACTCGTATCGTCGGTCTGATTATTAGT
CTGGGACCACGGTCCCACTCGTATCGTCGGTCTGATTATTAGTCTGGGACCAAGGTC
CCACTCGTATCGTCGGTCTGATTATTAGTCTGGGACCACGGTCCCACTCGTATCGTCG
GTCTGATTATTAGTCTGGGACCACGATCCCACTCGTGTTGTCGGTCTGATTATCGGTC
TGGGACCACGGTCCCACTTGTATTGTCGATCAGACTATCAGCGTGAGACTACGATTC
CATCAATGCCTGTCAAGGGCAAGTATTGACACTTCGTCTGTTTCTACTGGTATTGGCA
CAAACCTGATTCCAATTTGAGCAAGGCTATGTGCCATCTCGATACTCGTTCTTAACTC
AACAGAAGATGCTTTGTGCATACAGCCCCTCGTTTATTATTTATCTCCTCAGCCAGCC
GCTGTGCTTTCAGTGGATTTCGGATAACAGAAAGGCCGGGAAATACCCAGCCTCGCT
TTGTAACGGAGTAGAGACGAAAGTGATTGCGCCTACCCGGATATTATCGTGAGGATG
CGTCATCGCCATTAATTCACTGATCAGTGATAAGCTGTCATCTATGTCGGGTGCGGA
GAAAGAGGTAATGAAATGGCAGTTTAAACCGCCAGGGTTTTCCCAGTCACGACCCTA
GAGTCGAGCCAGGCGCGGGGTTCGCCCCGCAATTAATACGACTCACTATAGGGAGA
CCACAACGGTTTCCCTCTAGAAATAATTTTGTTTAACTTTAAGAAGGAGATATACAT
ATGGCCAGCAGCACCCCGCAGAACATTACCGATCTGTGCGCGGAATATCATAATACC
CAGATTCATACCCTGAATGATAAAATCTTCAGCTATACCGAAAGCCTGGCGGGTAAA
CGCGAAATGGCCATTATTACCTTTAAAAACGGTGCGACCTTTCAGGTTGAAGTGCCG
GGTAGCCAGCACATCGATAGCCAGAAAAAAGCGATTGAACGCATGAAAGATACCCT
GCGTATTGCGTATCTGACCGAAGCGAAAGTGGAAAAACTGTGCGTGTGGAACAATA
AAACCCCGCACGCGATCGCGGCGATCAGCATGGCCAACGGCCCGGGTCCGATGGAA
GGTAAAGCCCGTACCGCGCCGCAGGCCGGTGCGGCGGGTACCGCGACCACCGCGAG
CGTTCCGGGTACCACCACCGATGGCATGGATCCGGGCGTTGTGGCGACCACCAGCGT
GGTGACCGCCGAAAATAGCAGCGCCAGCATCGCGACCGCCGGCATTGGTGGCCCGC
CGCAGCAGGTGGATCAGCAGGAAACCTGGCGCACCAATTTCTATTATAATGATGTGT
TCACCTGGAGCGTTGCCGATGCCCCGGGTAGCATTCTGTATACCGTGCAGCATAGCC
CGCAGAACAATCCGTTTACCGCCGTTCTGAGCCAGATGTATGCGGGCTGGGCCGGTG
GCATGCAGTTCCGTTTCATTGTGGCGGGTAGCGGCGTTTTCGGCGGTCGCCTGGTGG

CCGCCGTGATTCCGCCGGGCATTGAAATTGGTCCGGGCCTGGAAGTGCGCCAGTTCC
CGCATGTGGTGATCGATGCGCGCAGCCTGGAACCGGTGACCATCACCATGCCGGATC
TGCGTCCGAACATGTATCATCCGACCGGTGATCCGGGCCTGGTGCCGACCCTGGTGC
TGAGCGTTTATAACAATCTGATCAACCCGTTTGGTGGCAGCACCAGCGCCATTCAGG
TTACCGTTGAAACCCGCCCGAGCGAAGATTTTGAATTTGTGATGATTCGCACCCCGA
GCAGCAAAACCGTGGATAGCATTTATCCGGCGGGCCTGCTGACCACCCCGGTGCTGA
CCGGTGTGGGCAACGATAACCGTTGGAATGGTCAGATCGTGGGCCTGCAGCCGGTTC
CGGGTGGTTTTAGCACCTGCAATCGTCATTGGAACCTGAACGGTAGCACCTATGGTT
GGAGCAGCCCGCGCTTTGCGGATATTGATCATCGCCGCGGCAGCGCGAGCTATCCGG
GTAGCAACGCGACCAATGTTCTGCAGTTTTGGTATGCCAATGCGGGCAGCGCGATTG
ATAACCCGATTAGCCAGGTGGCCCCGGATGGTTTTCCGGATATGAGCTTTGTGCCGT
TTAATGGCCCGGGCATCCCGGCGGCCGGTTGGGTGGGCTTTGGTGCCATTTGGAACA
GCAATAGCGGTGCGCCGAACGTGACCACCGTTCAGGCGTATGAACTGGGCTTTGCGA
CCGGTGCGCCGGGCAATCTGCAGCCGACCACCAACACCAGCGGCAGCCAGACCGTG
GCGAAAAGCATTTATGCCGTTGTTACCGGTACCGCGCAGAATCCGGCGGGCCTGTTT
GTTATGGCGAGCGGCGTGATTAGCACCCCGAGCGCCAATGCCATCACCTATACCCCG
CAGCCGGATCGTATCGTGACCACCCCGGGCACCCCGGCGGCGGCGCCGGTTGGCAA
AAATACCCCGATTATGTTCGCCAGCGTTGTTCGCCGTACCGGTGATGTTAACGCGAC
CGCGGGCAGCGCGAATGGCACCCAGTATGGCACCGGCAGCCAGCCGCTGCCGGTGA
CCATCGGTCTGAGCCTGAACAATTATAGCAGCGCGCTGATGCCGGGTCAGTTCTTCG
TGTGGCAGCTGACCTTTGCCAGCGGTTTTATGGAAATTGGCCTGAGCGTGGATGGCT
ATTTTTATGCGGGCACCGGTGCGAGCACCACCCTGATTGATCTGACCGAACTGATTG
ATGTTCGTCCGGTTGGTCCGCGCCCGAGCAAAAGCACCCTGGTTTTTAACCTGGGCG
GTACCGCGAACGGTTTTAGCTATGTGAGCGAAAAAGATGAACTGTAAAAGCTTAATT
AGCTGATAACTAGCATAACCCCTTGGGGCCTCTAAACGGGTCTTGAGGGGTTTTTTG
CTGAAAGGAGCTCGACCTGCAATCCTGTGTGAAATTGTTATCCGCTATTTAAATATTA
CCCTGTTATCCCTACAGCTTGGCACTGGCCACGCAAAAAGGCCATCCGTCAGGATGG
CCTTCTGCTTAATTTGATGCCTGGCAGTTTATGGCGGGCGTCCTGCCCGCCACCCTCC
GGGCCGTTGCTTCGCAACGTTCAAATCCGCTCCCGGCGGATTTGTCCTACTCAGGAG
AGCGTTCACCGACAAACAACAGATAAAACGAAAGGCCCAGTCTTTCGACTGAGCCTT
TCGTTTTATTTGATGCCTGGCAGTTCCCTACTCTCGCATGGGGAGACCCCACACTACC
ATCGG

pSG1144-*ctxvp60dezopt*
ctxvp60 gene: 6470-8560
Cm^R: complement 2-990
GGTACAATAAAAAACGCCCGGCGGCAACCGAGCGTTCATTTCTGCCATTCATCCGCT
TATTATCACTTATTCAGGCGTAGCAACCAGGCGTTTAAGGGCACCAATAACTGCCTT
AAAAAAATTACGCCCCGCCCTGCCACTCATCGCAGTACTGTTGTAATTCATTAAGCA
TTCTGCCGACATGGAAGCCATCACAAACGGCATGATGAACCTGAATCGCCAGCGGC
ATCAGCACCTTGTCGCCTTGCGTATAATATTTGCCCATGGTGAAAACGGGGGCGAAG
AAGTTGTCCATATTGGCCACGTTTAAATCAAAACTGGTGAAACTCACCCAGGGATTG
GCTGAGACGAAAAACATATTCTCAATAAACCCTTTAGGGAAATAGGCCAGGTTTTCA
CCGTAACACGCCACATCTTGCGAATATATGTGTAGAAACTGCCGGAAATCGTCGTGG
TATTCACTCCAGAGCGATGAAAACGTTTCAGTTTGCTCATGGAAAACGGTGTAACAA
GGGTGAACACTATCCCATATCACCAGCTCACCGTCTTTCATTGCCATACGTAATTCCG

GATGAGCATTCATCAGGCGGGCAAGAATGTGAATAAAGGCCGGATAAAACTTGTGC
TTATTTTTCTTTACGGTCTTTAAAAAGGCCGTAATATCCAGTTGAACGGTCTGGTTAT
AGGTACATTGAGCAACTGACTGAAATGCCTCAAAATGTTCTTTACGATGCCATTGGG
ATATATCAACGGTGGTATATCCAGTGATTTTTTTCTCCATTTTAGCTTCCTTAGCTCCT
GAAAATCTCGATAACTCAAAAAATACGCCCGGTAGTGATCTTATTTCATTATGGTGA
AAGTTGGAACCTCTTACGTGCCGATCAACGTCTCATTTTCGCCAAAAGTTGGCCCAG
GGCTTCCCGGTATCAACAGGGACACCAGGATTTATTTATTCTGCGAAGTGATCTTCC
GTCACAGGTATTTATTCGCGATACAGGGTTCGAGAAGGGGGGCACCCCCCTTCGGC
GTGCGCGGTCACGCGCACAGGGCGCAGCCCTGGTTAAAAACAAGGTTTATAAATATT
GGTTTAAAAGCAGGTTAAAAGACAGGTTAGCGGTGGCCGAAAAACGGGCGGAAACC
CTTGCAAATGCTGGATTTTCTGCCTGTGGACAGCCCCTCAAATGTCAATAGGTGCGC
CCCTCATCTGTCAGCACTCTGCCCCTCAAGTGTCAAGGATCGCGCCCCTCATCTGTCA
GTAGTCGCGCCCCTCAAGTGTCAATACCGCAGGGCACTTATCCCCAGGCTTGTCCAC
ATCATCTGTGGGAAACTCGCGTAAAATCAGGCGTTTTCGCCGATTTGCGAGGCTGGC
CAGCTCCACGTCGCCGGCCGAAATCGAGCCTGCCCCTCATCTGTAAACGCCGCGCCG
GGTGAGTCGGCCCCTCAAGTGTCAACGTCCGCCCCTCATCTGTCAGTGAGGGCCAAG
TTTTCCGCGAGGTATCCACAACGCCGGCGGTCGAGTGAGCGAGGAAGCACCAGGGA
ACAGCACTTATATATTCTGCTTACACACGATGCCTGAAAAAACTTCCCTTGGGGTTAT
CCACTTATCCACGGGGATATTTTTATAATTATTTTTTTTATAGTTTTTAGATCTTCTTT
TTTAGAGCGCCTTGTAGGCCTTTATCCATGCTGGTTCTAGAGAAGGTGTTGTGACAA
ATTGCCCTTTCAGTGTGACAAATCACCCTCAAATGACAGTCCTGTCTGTGACAAATTG
CCCTTAACCCTGTGACAAATTGCCCTCAGAAGAAGCTGTTTTTTCACAAAGTTATCCC
TGCTTATTGACTCTTTTTTATTTAGTGTGACAATCTAAAAACTTGTCACACTTCACAT
GGATCTGTCATGGCGGAAACAGCGGTTATCAATCACAAGAAACGTAAAAATAGCCC
GCGAATCGTCCAGTCAAACGACCTCACTGAGGCGGCATATAGTCTCTCCCGGGATCA
AAAACGTATGCTGTATCTGTTCGTTGACCAGATCAGAAAATCTGATGGCACCCTACA
GGAACATGACGGTATCTGCGAGATCCATGTTGCTAAATATGCTGAAATATTCGGATT
GACCTCTGCGGAAGCCAGTAAGGATATACGGCAGGCATTGAAGAGTTTCGCGGGGA
AGGAAGTGGTTTTTTATCGCCCTGAAGAGGATGCCGGCGATGAAAAAGGCTATGAAT
CTTTTCCTTGGTTTATCAAACGTGCGCACAGTCCATCCAGAGGGCTTTACAGTGTACA
TATCAACCCATATCTCATTCCCTTCTTTATCGGGTTACAGAACCGGTTTACGCAGTTT
CGGCTTAGTGAAACAAAAGAAATCACCAATCCGTATGCCATGCGTTTATACGAATCC
CTGTGTCAGTATCGTAAGCCGGATGGCTCAGGCATCGTCTCTCTGAAAATCGACTGG
ATCATAGAGCGTTACCAACTGCCTCAAAGTTACCAGCGTATGCCTGACTTCCGCCGC
CGCTTCCTGCAGGTCTGTGTTAATGAGATCAACAGCAGAACTCCAATGCGCCTCTCA
TACATTGAGAAAAAGAAAGGCCGCCAGACGACTCATATCGTATTTTCCTTCCGCGAT
ATCACTTCCATGACGACAGGATAGTCTGAGGGTTATCTGTCACAGATTTGAGGGTGG
TTCGTCACATTTGTTCTGACCTACTGAGGGTAATTTGTCACAGTTTTGCTGTTTCCTTC
AGCCTGCATGGATTTTCTCATACTTTTTGAACTGTAATTTTTAAGGAAGCCAAATTTG
AGGGCAGTTTGTCACAGTTGATTTCCTTCTCTTTCCCTTCGTCATGTGACCTGATATC
GGGGGTTAGTTCGTCATCATTGATGAGGGTTGATTATCACAGTTTATTACTCTGAATT
GGCTATCCGCGTGTGTACCTCTACCTGGAGTTTTTCCCACGGTGGATATTTCTTCTTG
CGCTGAGCGTAAGAGCTATCTGACAGAACAGTTCTTCTTTGCTTCCTCGCCAGTTCGC
TCGCTATGCTCGGTTACACGGCTGCGGCGAGCGCTAGTGATAATAAGTGACTGAGGT
ATGTGCTCTTCTTATCTCCTTTTGTAGTGTTGCTCTTATTTTAAACAACTTTGCGGTTT
TTTGATGACTTTGCGATTTTGTTGTTGCTTTGCAGTAAATTGCAAGATTTAATAAAAA
AACGCAAAGCAATGATTAAAGGATGTTCAGAATGAAACTCATGGAAACACTTAACC

AGTGCATAAACGCTGGTCATGAAATGACGAAGGCTATCGCCATTGCACAGTTTAATG
ATGACAGCCCGGAAGCGAGGAAAATAACCCGGCGCTGGAGAATAGGTGAAGCAGC
GGATTTAGTTGGGGTTTCTTCTCAGGCTATCAGAGATGCCGAGAAAGCAGGGCGACT
ACCGCACCCGGATATGGAAATTCGAGGACGGGTTGAGCAACGTGTTGGTTATACAAT
TGAACAAATTAATCACATGCGTGATGTGTTTGGTACGCGATTGCGACGTGCTGAAGA
CGTATTTCCACCGGTGATCGGGGTTGCTGCGCATAAAGGCGGCGTTTACAAAACCTC
AGTTTCTGTTCATCTTGCTCAGGATCTGGCTCTGAAGGGGCTACGTGTTTTGCTCGTG
GAAGGTAACGACCCCCAGGGAACAGCCTCAATGTATCACGGATGGGTACCAGATCT
TCATATTCATGCAGAAGACACTCTCCTGCCTTTCTATCTTGGGGAAAAGGACGATGT
CACTTATGCAATAAAGCCCACTTGCTGGCCGGGGCTTGACATTATTCCTTCCTGTCTG
GCTCTGCACCGTATTGAAACTGAGTTAATGGGCAAATTTGATGAAGGTAAACTGCCC
ACCGATCCACACCTGATGCTCCGACTGGCCATTGAAACTGTTGCTCATGACTATGAT
GTCATAGTTATTGACAGCGCGCCTAACCTGGGTATCGGCACGATTAATGTCGTATGT
GCTGCTGATGTGCTGATTGTTCCCACGCCTGCTGAGTTGTTTGACTACACCTCCGCAC
TGCAGTTTTTCGATATGCTTCGTGATCTGCTCAAGAACGTTGATCTTAAAGGGTTCGA
GCCTGATGTACGTATTTTGCTTACCAAATACAGCAATAGTAATGGCTCTCAGTCCCCG
TGGATGGAGGAGCAAATTCGGGATGCCTGGGGAAGCATGGTTCTAAAAAATGTTGT
ACGTGAAACGGATGAAGTTGGTAAAGGTCAGATCCGGATGAGAACTGTTTTTGAAC
AGGCCATTGATCAACGCTCTTCAACTGGTGCCTGGAGAAATGCTCTTTCTATTTGGGA
ACCTGTCTGCAATGAAATTTTCGATCGTCTGATTAAACCACGCTGGGAGATTAGATA
ATGAAGCGTGCGCCTGTTATTCCAAAACATACGCTCAATACTCAACCGGTTGAAGAT
ACTTCGTTATCGACCCCTGCTGCCCCGATGGTGGATTCGTTAATTGCGCGCGTAGGA
GTAATGGCTCGCGGTAATGCCATTACTTTGCCTGTATGTGGTCGGGATGTGAAGTTTA
CTCTTGAAGTGCTCCGGGGTGATAGTGTTGAGAAGACCTCTCGGGTATGGTCAGGTA
ATGAACGTGACCAGGAGCTGCTTACTGAGGACGCACTGGATGATCTCATCCCTTCTT
TTCTACTGACTGGTCAACAGACACCGGCGTTCGGTCGAAGAGTATCTGGTGTCATAG
AAATTGCCGATGGGAGTCGCCGTCGTAAAGCTGCTGCACTTACCGAAAGTGATTATC
GTGTTCTGGTTGGCGAGCTGGATGATGAGCAGATGGCTGCATTATCCAGATTGGGTA
ACGATTATCGCCCAACAAGTGCTTATGAACGTGGTCAGCGTTATGCAAACCGATTGC
AGAATGAATTTGCTGGAAATATTTCTGCGCTGGCTGATGCGGAAAATATTTCACGTA
AGATTATTACCCGCTGTATCAACACCGCCAAATTGCCTAAATCAGTTGTTGCTCTTTT
TTCTCACCCCGGTGAACTATCTGCCCGGTCAGGTGATGCACTTCAAAAAGCCTTTAC
AGATAAAGAGGAATTACTTAAGCAGCAGGCATCTAACCTTCATGAGCAGAAAAAAG
CTGGGGTGATATTTGAAGCTGAAGAAGTTATCACTCTTTTAACTTCTGTGCTTAAAAC
GTCATCTGCATCAAGAACTAGTTTAAGCTCACGACATCAGTTTGCTCCTGGAGCGAC
AGTATTGTATAAGGGCGATAAAATGGTGCTTAACCTGGACAGGTCTCGTGTTCCAAC
TGAGTGTATAGAGAAAATTGAGGCCATTCTTAAGGAACTTGAAAAGCCAGCACCCTG
ATGCGACCACGTTTTAGTCTACGTTTATCTGTCTTTACTTAATGTCCTTTGTTACAGGC
CAGAAAGCATAACTGGCCTGAATATTCTCTCTGGGCCCACTGTTCCACTTGTATCGTC
GGTCTGATTATTAGTCTGGGACCAAGGTCCCACTCGTATCGTCGGTCTGATTATTAGT
CTGGGACCACGGTCCCACTCGTATCGTCGGTCTGATTATTAGTCTGGGACCACGGTC
CCACTCGTATCGTCGGTCTGATAATCAGACTGGGACCACGGTCCCACTCGTATCGTC
GGTCTGATTATTAGTCTGGGACCAAGGTCCCACTCGTATCGTCGGTCTGATTATTAGT
CTGGGACCACGGTCCCACTCGTATCGTCGGTCTGATTATTAGTCTGGGACCAAGGTC
CCACTCGTATCGTCGGTCTGATTATTAGTCTGGGACCACGGTCCCACTCGTATCGTCG
GTCTGATTATTAGTCTGGGACCACGATCCCACTCGTGTTGTCGGTCTGATTATCGGTC
TGGGACCACGGTCCCACTTGTATTGTCGATCAGACTATCAGCGTGAGACTACGATTC

CATCAATGCCTGTCAAGGGCAAGTATTGACACTTCGTCTGTTTCTACTGGTATTGGCA
CAAACCTGATTCCAATTTGAGCAAGGCTATGTGCCATCTCGATACTCGTTCTTAACTC
AACAGAAGATGCTTTGTGCATACAGCCCCTCGTTTATTATTTATCTCCTCAGCCAGCC
GCTGTGCTTTCAGTGGATTTCGGATAACAGAAAGGCCGGGAAATACCCAGCCTCGCT
TTGTAACGGAGTAGAGACGAAAGTGATTGCGCCTACCCGGATATTATCGTGAGGATG
CGTCATCGCCATTAATTCACTGATCAGTGATAAGCTGTCATCTATGTCGGGTGCGGA
GAAAGAGGTAATGAAATGGCAGTTTAAACCGCCAGGGTTTTCCCAGTCACGACCCTA
GAGTCGAGCCAGGCGCGGGGTTCGCCCCGCAATTAATACGACTCACTATAGGGAGA
CCACAACGGTTTCCCTCTAGAAATAATTTTGTTTAACTTTAAGAAGGAGATATACAT
ATGGCCAGCAGCACCCCGCAGAACATTACCGATCTGTGCGCGGAATATCATAATACC
CAGATTCATACCCTGAATGATAAAATCTTCAGCTATACCGAAAGCCTGGCGGGTAAA
AGGGAAATGGCCATTATTACCTTTAAAAACGGTGCGACCTTTCAGGTTGAAGTGCCG
GGTAGCCAGCACATCGATAGCCAGAAAAAAGCGATTGAAAGGATGAAAGATACCCT
GAGGATTGCGTATCTGACCGAAGCGAAAGTGGAAAAACTGTGCGTGTGGAACAATA
AAACCCCGCACGCGATCGCGGCGATCAGCATGGCCAACGGCCCGGGTCCGATGGAA
GGTAAAGCCAGGACCGCGCCGCAGGCCGGTGCGGCGGGTACCGCGACCACCGCGAG
CGTTCCGGGTACCACCACCGATGGCATGGATCCGGGCGTTGTGGCGACCACCAGCGT
GGTGACCGCCGAAAATAGCAGCGCCAGCATCGCGACCGCCGGCATTGGTGGCCCGC
CGCAGCAGGTGGATCAGCAGGAAACCTGGAGGACCAATTTCTATTATAATGATGTGT
TCACCTGGAGCGTTGCCGATGCCCCGGGTAGCATTCTGTATACCGTGCAGCATAGCC
CGCAGAACAATCCGTTTACCGCCGTTCTGAGCCAGATGTATGCGGGCTGGGCCGGTG
GCATGCAGTTCAGGTTCATTGTGGCGGGTAGCGGCGTTTTCGGCGGTAGGCTGGTGG
CCGCCGTGATTCCGCCGGGCATTGAAATTGGTCCGGGCCTGGAAGTGAGGCAGTTCC
CGCATGTGGTGATCGATGCGAGGAGCCTGGAACCGGTGACCATCACCATGCCGGATC
TGAGGCCGAACATGTATCATCCGACCGGTGATCCGGGCCTGGTGCCGACCCTGGTGC
TGAGCGTTTATAACAATCTGATCAACCCGTTTGGTGGCAGCACCAGCGCCATTCAGG
TTACCGTTGAAACCAGGCCGAGCGAAGATTTTGAATTTGTGATGATTAGGACCCCGA
GCAGCAAAACCGTGGATAGCATTTATCCGGCGGGCCTGCTGACCACCCCGGTGCTGA
CCGGTGTGGGCAACGATAACAGGTGGAATGGTCAGATCGTGGGCCTGCAGCCGGTT
CCGGGTGGTTTTAGCACCTGCAATAGGCATTGGAACCTGAACGGTAGCACCTATGGT
TGGAGCAGCCCGAGGTTTGCGGATATTGATCATAGGAGGGGCAGCGCGAGCTATCC
GGGTAGCAACGCGACCAATGTTCTGCAGTTTTGGTATGCCAATGCGGGCAGCGCGAT
TGATAACCCGATTAGCCAGGTGGCCCCGGATGGTTTTCCGGATATGAGCTTTGTGCC
GTTTAATGGCCCGGGCATCCCGGCGGCCGGTTGGGTGGGCTTTGGTGCCATTTGGAA
CAGCAATAGCGGTGCGCCGAACGTGACCACCGTTCAGGCGTATGAACTGGGCTTTGC
GACCGGTGCGCCGGGCAATCTGCAGCCGACCACCAACACCAGCGGCAGCCAGACCG
TGGCGAAAAGCATTTATGCCGTTGTTACCGGTACCGCGCAGAATCCGGCGGGCCTGT
TTGTTATGGCGAGCGGCGTGATTAGCACCCCGAGCGCCAATGCCATCACCTATACCC
CGCAGCCGGATAGGATCGTGACCACCCCGGGCACCCCGGCGGCGGCGCCGGTTGGC
AAAAATACCCCGATTATGTTCGCCAGCGTTGTTAGGAGGACCGGTGATGTTAACGCG
ACCGCGGGCAGCGCGAATGGCACCCAGTATGGCACCGGCAGCCAGCCGCTGCCGGT
GACCATCGGTCTGAGCCTGAACAATTATAGCAGCGCGCTGATGCCGGGTCAGTTCTT
CGTGTGGCAGCTGACCTTTGCCAGCGGTTTTATGGAAATTGGCCTGAGCGTGGATGG
CTATTTTTATGCGGGCACCGGTGCGAGCACCACCCTGATTGATCTGACCGAACTGAT
TGATGTTAGGCCGGTTGGTCCGAGGCCGAGCAAAAGCACCCTGGTTTTTAACCTGGG
CGGTACCGCGAACGGTTTTAGCTATGTGAGCGAAAAAGATGAACTGTAAAAGCTTA
ATTAGCTGATAACTAGCATAACCCCTTGGGGCCTCTAAACGGGTCTTGAGGGGTTTT

TTGCTGAAAGGAGCTCGACCTGCAATCCTGTGTGAAATTGTTATCCGCTATTTAAATA
TTACCCTGTTATCCCTACAGCTTGGCACTGGCCACGCAAAAAGGCCATCCGTCAGGA
TGGCCTTCTGCTTAATTTGATGCCTGGCAGTTTATGGCGGGCGTCCTGCCCGCCACCC
TCCGGGCCGTTGCTTCGCAACGTTCAAATCCGCTCCCGGCGGATTTGTCCTACTCAGG
AGAGCGTTCACCGACAAACAACAGATAAAACGAAAGGCCCAGTCTTTCGACTGAGC
CTTTCGTTTTATTTGATGCCTGGCAGTTCCCTACTCTCGCATGGGGAGACCCCACACT
ACCATCGG
