# Supplementary material for: Reduced evolvability of Escherichia coli MDS42, an IS-less cellular chassis for molecular and synthetic biology applications
Source: Microb Cell Fact. 2010 May 21;9:38. doi: 10.1186/1475-2859-9-38 (PMC2891674; doi:10.1186/1475-2859-9-38)
Supplement: Additional file 2 — Translation and codon usage of ctxvp60opt gene. [file 1475-2859-9-38-S2.PDF]

CTXVP60opt_gene

```
5' ATG GCC AGC AGC ACC CCG CAG AAC ATT ACC GAT CTG TGC GCG GAA
o                                                              45
3' TAC CGG TCG TCG TGG GGC GTC TTG TAA TGG CTA GAC ACG CGC CTT
1  Met Ala Ser Ser Thr Pro Gln Asn Ile Thr Asp Leu Cys Ala Glu
o
5' TAT CAT AAT ACC CAG ATT CAT ACC CTG AAT GAT AAA ATC TTC AGC
o                                                              90
3' ATA GTA TTA TGG GTC TAA GTA TGG GAC TTA CTA TTT TAG AAG TCG
1  Tyr His Asn Thr Gln Ile His Thr Leu Asn Asp Lys Ile Phe Ser
o
5' TAT ACC GAA AGC CTG GCG GGT AAA CGC GAA ATG GCC ATT ATT ACC
o                                                              135
3' ATA TGG CTT TCG GAC CGC CCA TTT GCG CTT TAC CGG TAA TAA TGG
1  Tyr Thr Glu Ser Leu Ala Gly Lys Arg Glu Met Ala Ile Ile Thr
o
5' TTT AAA AAC GGT GCG ACC TTT CAG GTT GAA GTG CCG GGT AGC CAG
o                                                              180
3' AAA TTT TTG CCA CGC TGG AAA GTC CAA CTT CAC GGC CCA TCG GTC
1  Phe Lys Asn Gly Ala Thr Phe Gln Val Glu Val Pro Gly Ser Gln
o
5' CAC ATC GAT AGC CAG AAA AAA GCG ATT GAA CGC ATG AAA GAT ACC
o                                                              225
3' GTG TAG CTA TCG GTC TTT TTT CGC TAA CTT GCG TAC TTT CTA TGG
1  His Ile Asp Ser Gln Lys Lys Ala Ile Glu Arg Met Lys Asp Thr
o
5' CTG CGT ATT GCG TAT CTG ACC GAA GCG AAA GTG GAA AAA CTG TGC
o                                                              270
3' GAC GCA TAA CGC ATA GAC TGG CTT CGC TTT CAC CTT TTT GAC ACG
1  Leu Arg Ile Ala Tyr Leu Thr Glu Ala Lys Val Glu Lys Leu Cys
o
5' GTG TGG AAC AAT AAA ACC CCG CAC GCG ATC GCG GCG ATC AGC ATG
o                                                              315
3' CAC ACC TTG TTA TTT TGG GGC GTG CGC TAG CGC CGC TAG TCG TAC
1  Val Trp Asn Asn Lys Thr Pro His Ala Ile Ala Ala Ile Ser Met
o
5' GCC AAC GGC CCG GGT CCG ATG GAA GGT AAA GCC CGT ACC GCG CCG
o                                                              360
3' CGG TTG CCG GGC CCA GGC TAC CTT CCA TTT CGG GCA TGG CGC GGC
1  Ala Asn Gly Pro Gly Pro Met Glu Gly Lys Ala Arg Thr Ala Pro
o
5' CAG GCC GGT GCG GCG GGT ACC GCG ACC ACC GCG AGC GTT CCG GGT
o                                                              405
3' GTC CGG CCA CGC CGC CCA TGG CGC TGG TGG CGC TCG CAA GGC CCA
1  Gln Ala Gly Ala Ala Gly Thr Ala Thr Thr Ala Ser Val Pro Gly
o
5' ACC ACC ACC GAT GGC ATG GAT CCG GGC GTT GTG GCG ACC ACC AGC
o                                                              450
3' TGG TGG TGG CTA CCG TAC CTA GGC CCG CAA CAC CGC TGG TGG TCG
1  Thr Thr Thr Asp Gly Met Asp Pro Gly Val Val Ala Thr Thr Ser
o
5' GTG GTG ACC GCC GAA AAT AGC AGC GCC AGC ATC GCG ACC GCC GGC
o                                                              495
3' CAC CAC TGG CGG CTT TTA TCG TCG CGG TCG TAG CGC TGG CGG CCG
1  Val Val Thr Ala Glu Asn Ser Ser Ala Ser Ile Ala Thr Ala Gly
o
```

5' ACC AAT TTC TAT TAT AAT GAT GTG TTC ACC TGG AGC GTT GCC GAT
o 585
3' TGG TTA AAG ATA ATA TTA CTA CAC AAG TGG ACC TCG CAA CGG CTA
1 Thr Asn Phe Tyr Tyr Asn Asp Val Phe Thr Trp Ser Val Ala Asp
o

5' GCC CCG GGT AGC ATT CTG TAT ACC GTG CAG CAT AGC CCG CAG AAC
o 630
3' CGG GGC CCA TCG TAA GAC ATA TGG CAC GTC GTA TCG GGC GTC TTG
1 Ala Pro Gly Ser Ile Leu Tyr Thr Val Gln His Ser Pro Gln Asn
o

5' AAT CCG TTT ACC GCC GTT CTG AGC CAG ATG TAT GCG GGC TGG GCC
o 675
3' TTA GGC AAA TGG CGG CAA GAC TCG GTC TAC ATA CGC CCG ACC CGG
1 Asn Pro Phe Thr Ala Val Leu Ser Gln Met Tyr Ala Gly Trp Ala
o

5' GGT GGC ATG CAG TTC CGT TTC ATT GTG GCG GGT AGC GGC GTT TTC
o 720
3' CCA CCG TAC GTC AAG GCA AAG TAA CAC CGC CCA TCG CCG CAA AAG
1 Gly Gly Met Gln Phe Arg Phe Ile Val Ala Gly Ser Gly Val Phe
o

5' GGC GGT CGC CTG GTG GCC GCC GTG ATT CCG CCG GGC ATT GAA ATT
o 765
3' CCG CCA GCG GAC CAC CGG CGG CAC TAA GGC GGC CCG TAA CTT TAA
1 Gly Gly Arg Leu Val Ala Ala Val Ile Pro Pro Gly Ile Glu Ile
o

5' GGT CCG GGC CTG GAA GTG CGC CAG TTC CCG CAT GTG GTG ATC GAT
o 810
3' CCA GGC CCG GAC CTT CAC GCG GTC AAG GGC GTA CAC CAC TAG CTA
1 Gly Pro Gly Leu Glu Val Arg Gln Phe Pro His Val Val Ile Asp
o

5' GCG CGC AGC CTG GAA CCG GTG ACC ATC ACC ATG CCG GAT CTG CGT
o 855
3' CGC GCG TCG GAC CTT GGC CAC TGG TAG TGG TAC GGC CTA GAC GCA
1 Ala Arg Ser Leu Glu Pro Val Thr Ile Thr Met Pro Asp Leu Arg
o

5' CCG AAC ATG TAT CAT CCG ACC GGT GAT CCG GGC CTG GTG CCG ACC
o 900
3' GGC TTG TAC ATA GTA GGC TGG CCA CTA GGC CCG GAC CAC GGC TGG
1 Pro Asn Met Tyr His Pro Thr Gly Asp Pro Gly Leu Val Pro Thr
o

5' CTG GTG CTG AGC GTT TAT AAC AAT CTG ATC AAC CCG TTT GGT GGC
o 945
3' GAC CAC GAC TCG CAA ATA TTG TTA GAC TAG TTG GGC AAA CCA CCG
1 Leu Val Leu Ser Val Tyr Asn Asn Leu Ile Asn Pro Phe Gly Gly
o

5' AGC ACC AGC GCC ATT CAG GTT ACC GTT GAA ACC CGC CCG AGC GAA
o 990
3' TCG TGG TCG CGG TAA GTC CAA TGG CAA CTT TGG GCG GGC TCG CTT
1 Ser Thr Ser Ala Ile Gln Val Thr Val Glu Thr Arg Pro Ser Glu
o

5' GAT TTT GAA TTT GTG ATG ATT CGC ACC CCG AGC AGC AAA ACC GTG
o 1035
3' CTA AAA CTT AAA CAC TAC TAA GCG TGG GGC TCG TCG TTT TGG CAC
1 Asp Phe Glu Phe Val Met Ile Arg Thr Pro Ser Ser Lys Thr Val
o

```
5'  GGT GTG GGC AAC GAT AAC CGT TGG AAT GGT CAG ATC GTG GGC CTG
o                                                                 1125
3'  CCA CAC CCG TTG CTA TTG GCA ACC TTA CCA GTC TAG CAC CCG GAC
1   Gly Val Gly Asn Asp Asn Arg Trp Asn Gly Gln Ile Val Gly Leu
o
5'  CAG CCG GTT CCG GGT GGT TTT AGC ACC TGC AAT CGT CAT TGG AAC
o                                                                 1170
3'  GTC GGC CAA GGC CCA CCA AAA TCG TGG ACG TTA GCA GTA ACC TTG
1   Gln Pro Val Pro Gly Gly Phe Ser Thr Cys Asn Arg His Trp Asn
o
5'  CTG AAC GGT AGC ACC TAT GGT TGG AGC AGC CCG CGC TTT GCG GAT
o                                                                 1215
3'  GAC TTG CCA TCG TGG ATA CCA ACC TCG TCG GGC GCG AAA CGC CTA
1   Leu Asn Gly Ser Thr Tyr Gly Trp Ser Ser Pro Arg Phe Ala Asp
o
5'  ATT GAT CAT CGC CGC GGC AGC GCG AGC TAT CCG GGT AGC AAC GCG
o                                                                 1260
3'  TAA CTA GTA GCG GCG CCG TCG CGC TCG ATA GGC CCA TCG TTG CGC
1   Ile Asp His Arg Arg Gly Ser Ala Ser Tyr Pro Gly Ser Asn Ala
o
5'  ACC AAT GTT CTG CAG TTT TGG TAT GCC AAT GCG GGC AGC GCG ATT
o                                                                 1305
3'  TGG TTA CAA GAC GTC AAA ACC ATA CGG TTA CGC CCG TCG CGC TAA
1   Thr Asn Val Leu Gln Phe Trp Tyr Ala Asn Ala Gly Ser Ala Ile
o
5'  GAT AAC CCG ATT AGC CAG GTG GCC CCG GAT GGT TTT CCG GAT ATG
o                                                                 1350
3'  CTA TTG GGC TAA TCG GTC CAC CGG GGC CTA CCA AAA GGC CTA TAC
1   Asp Asn Pro Ile Ser Gln Val Ala Pro Asp Gly Phe Pro Asp Met
o
5'  AGC TTT GTG CCG TTT AAT GGC CCG GGC ATC CCG GCG GCC GGT TGG
o                                                                 1395
3'  TCG AAA CAC GGC AAA TTA CCG GGC CCG TAG GGC CGC CGG CCA ACC
1   Ser Phe Val Pro Phe Asn Gly Pro Gly Ile Pro Ala Ala Gly Trp
o
5'  GTG GGC TTT GGT GCC ATT TGG AAC AGC AAT AGC GGT GCG CCG AAC
o                                                                 1440
3'  CAC CCG AAA CCA CGG TAA ACC TTG TCG TTA TCG CCA CGC GGC TTG
1   Val Gly Phe Gly Ala Ile Trp Asn Ser Asn Ser Gly Ala Pro Asn
o
5'  GTG ACC ACC GTT CAG GCG TAT GAA CTG GGC TTT GCG ACC GGT GCG
o                                                                 1485
3'  CAC TGG TGG CAA GTC CGC ATA CTT GAC CCG AAA CGC TGG CCA CGC
1   Val Thr Thr Val Gln Ala Tyr Glu Leu Gly Phe Ala Thr Gly Ala
o
5'  CCG GGC AAT CTG CAG CCG ACC ACC AAC ACC AGC GGC AGC CAG ACC
o                                                                 1530
3'  GGC CCG TTA GAC GTC GGC TGG TGG TTG TGG TCG CCG TCG GTC TGG
1   Pro Gly Asn Leu Gln Pro Thr Thr Asn Thr Ser Gly Ser Gln Thr
o
5'  GTG GCG AAA AGC ATT TAT GCC GTT GTT ACC GGT ACC GCG CAG AAT
o                                                                 1575
3'  CAC CGC TTT TCG TAA ATA CGG CAA CAA TGG CCA TGG CGC GTC TTA
1   Val Ala Lys Ser Ile Tyr Ala Val Val Thr Gly Thr Ala Gln Asn
o
```

CTXVP60opt_gene

```
5'  AGC GCC AAT GCC ATC ACC TAT ACC CCG CAG CCG GAT CGT ATC GTG
o                                                                1665
3'  TCG CGG TTA CGG TAG TGG ATA TGG GGC GTC GGC CTA GCA TAG CAC
1   Ser Ala Asn Ala Ile Thr Tyr Thr Pro Gln Pro Asp Arg Ile Val
o
5'  ACC ACC CCG GGC ACC CCG GCG GCG GCG CCG GTT GGC AAA AAT ACC
o                                                                1710
3'  TGG TGG GGC CCG TGG GGC CGC CGC CGC GGC CAA CCG TTT TTA TGG
1   Thr Thr Pro Gly Thr Pro Ala Ala Ala Pro Val Gly Lys Asn Thr
o
5'  CCG ATT ATG TTC GCC AGC GTT GTT CGC CGT ACC GGT GAT GTT AAC
o                                                                1755
3'  GGC TAA TAC AAG CGG TCG CAA CAA GCG GCA TGG CCA CTA CAA TTG
1   Pro Ile Met Phe Ala Ser Val Val Arg Arg Thr Gly Asp Val Asn
o
5'  GCG ACC GCG GGC AGC GCG AAT GGC ACC CAG TAT GGC ACC GGC AGC
o                                                                1800
3'  CGC TGG CGC CCG TCG CGC TTA CCG TGG GTC ATA CCG TGG CCG TCG
1   Ala Thr Ala Gly Ser Ala Asn Gly Thr Gln Tyr Gly Thr Gly Ser
o
5'  CAG CCG CTG CCG GTG ACC ATC GGT CTG AGC CTG AAC AAT TAT AGC
o                                                                1845
3'  GTC GGC GAC GGC CAC TGG TAG CCA GAC TCG GAC TTG TTA ATA TCG
1   Gln Pro Leu Pro Val Thr Ile Gly Leu Ser Leu Asn Asn Tyr Ser
o
5'  AGC GCG CTG ATG CCG GGT CAG TTC TTC GTG TGG CAG CTG ACC TTT
o                                                                1890
3'  TCG CGC GAC TAC GGC CCA GTC AAG AAG CAC ACC GTC GAC TGG AAA
1   Ser Ala Leu Met Pro Gly Gln Phe Phe Val Trp Gln Leu Thr Phe
o
5'  GCC AGC GGT TTT ATG GAA ATT GGC CTG AGC GTG GAT GGC TAT TTT
o                                                                1935
3'  CGG TCG CCA AAA TAC CTT TAA CCG GAC TCG CAC CTA CCG ATA AAA
1   Ala Ser Gly Phe Met Glu Ile Gly Leu Ser Val Asp Gly Tyr Phe
o
5'  TAT GCG GGC ACC GGT GCG AGC ACC ACC CTG ATT GAT CTG ACC GAA
o                                                                1980
3'  ATA CGC CCG TGG CCA CGC TCG TGG TGG GAC TAA CTA GAC TGG CTT
1   Tyr Ala Gly Thr Gly Ala Ser Thr Thr Leu Ile Asp Leu Thr Glu
o
5'  CTG ATT GAT GTT CGT CCG GTT GGT CCG CGC CCG AGC AAA AGC ACC
o                                                                2025
3'  GAC TAA CTA CAA GCA GGC CAA CCA GGC GCG GGC TCG TTT TCG TGG
1   Leu Ile Asp Val Arg Pro Val Gly Pro Arg Pro Ser Lys Ser Thr
o
5'  CTG GTT TTT AAC CTG GGC GGT ACC GCG AAC GGT TTT AGC TAT GTG
o                                                                2070
3'  GAC CAA AAA TTG GAC CCG CCA TGG CGC TTG CCA AAA TCG ATA CAC
1   Leu Val Phe Asn Leu Gly Gly Thr Ala Asn Gly Phe Ser Tyr Val
o
5'  AGC GAA AAA GAT GAA CTG TAA
o                                                                2091
3'  TCG CTT TTT CTA CTT GAC ATT
1   Ser Glu Lys Asp Glu Leu  .
o
```
