# Supplementary material for: Reduced evolvability of Escherichia coli MDS42, an IS-less cellular chassis for molecular and synthetic biology applications
Source: Microb Cell Fact. 2010 May 21;9:38. doi: 10.1186/1475-2859-9-38 (PMC2891674; doi:10.1186/1475-2859-9-38)
Supplement: Additional file 3 — Translation and codon usage of ctxvp60dezopt gene. [file 1475-2859-9-38-S3.PDF]

CTXVP60dezopt_gene

5' ATG GCC AGC AGC ACC CCG CAG AAC ATT ACC GAT CTG TGC GCG GAA 45
1 Met Ala Ser Ser Thr Pro Gln Asn Ile Thr Asp Leu Cys Ala Glu

5' TAT CAT AAT ACC CAG ATT CAT ACC CTG AAT GAT AAA ATC TTC AGC 90
1 Tyr His Asn Thr Gln Ile His Thr Leu Asn Asp Lys Ile Phe Ser

5' TAT ACC GAA AGC CTG GCG GGT AAA AGG GAA ATG GCC ATT ATT ACC 135
1 Tyr Thr Glu Ser Leu Ala Gly Lys Arg Glu Met Ala Ile Ile Thr

5' TTT AAA AAC GGT GCG ACC TTT CAG GTT GAA GTG CCG GGT AGC CAG 180
1 Phe Lys Asn Gly Ala Thr Phe Gln Val Glu Val Pro Gly Ser Gln

5' CAC ATC GAT AGC CAG AAA AAA GCG ATT GAA AGG ATG AAA GAT ACC 225
1 His Ile Asp Ser Gln Lys Lys Ala Ile Glu Arg Met Lys Asp Thr

5' CTG AGG ATT GCG TAT CTG ACC GAA GCG AAA GTG GAA AAA CTG TGC 270
1 Leu Arg Ile Ala Tyr Leu Thr Glu Ala Lys Val Glu Lys Leu Cys

5' GTG TGG AAC AAT AAA ACC CCG CAC GCG ATC GCG GCG ATC AGC ATG 315
1 Val Trp Asn Asn Lys Thr Pro His Ala Ile Ala Ala Ile Ser Met

5' GCC AAC GGC CCG GGT CCG ATG GAA GGT AAA GCC AGG ACC GCG CCG 360
1 Ala Asn Gly Pro Gly Pro Met Glu Gly Lys Ala Arg Thr Ala Pro

5' CAG GCC GGT GCG GCG GGT ACC GCG ACC ACC GCG AGC GTT CCG GGT 405
1 Gln Ala Gly Ala Ala Gly Thr Ala Thr Thr Ala Ser Val Pro Gly

5' ACC ACC ACC GAT GGC ATG GAT CCG GGC GTT GTG GCG ACC ACC AGC 450
1 Thr Thr Thr Asp Gly Met Asp Pro Gly Val Val Ala Thr Thr Ser

5' GTG GTG ACC GCC GAA AAT AGC AGC GCC AGC ATC GCG ACC GCC GGC 495
1 Val Val Thr Ala Glu Asn Ser Ser Ala Ser Ile Ala Thr Ala Gly

5' ATT GGT GGC CCG CCG CAG CAG GTG GAT CAG CAG GAA ACC TGG AGG 540
1 Ile Gly Gly Pro Pro Gln Gln Val Asp Gln Gln Glu Thr Trp Arg

5' ACC AAT TTC TAT TAT AAT GAT GTG TTC ACC TGG AGC GTT GCC GAT 585
1 Thr Asn Phe Tyr Tyr Asn Asp Val Phe Thr Trp Ser Val Ala Asp

5' GCC CCG GGT AGC ATT CTG TAT ACC GTG CAG CAT AGC CCG CAG AAC 630
1 Ala Pro Gly Ser Ile Leu Tyr Thr Val Gln His Ser Pro Gln Asn

5' AAT CCG TTT ACC GCC GTT CTG AGC CAG ATG TAT GCG GGC TGG GCC 675
1 Asn Pro Phe Thr Ala Val Leu Ser Gln Met Tyr Ala Gly Trp Ala

```
5' GGT GGC ATG CAG TTC AGG TTC ATT GTG GCG GGT AGC GGC GTT TTC
o                                                               720
1  Gly Gly Met Gln Phe Arg Phe Ile Val Ala Gly Ser Gly Val Phe
o
5' GGC GGT AGG CTG GTG GCC GCC GTG ATT CCG CCG GGC ATT GAA ATT
o                                                               765
1  Gly Gly Arg Leu Val Ala Ala Val Ile Pro Pro Gly Ile Glu Ile
o
5' GGT CCG GGC CTG GAA GTG AGG CAG TTC CCG CAT GTG GTG ATC GAT
o                                                               810
1  Gly Pro Gly Leu Glu Val Arg Gln Phe Pro His Val Val Ile Asp
o
5' GCG AGG AGC CTG GAA CCG GTG ACC ATC ACC ATG CCG GAT CTG AGG
o                                                               855
1  Ala Arg Ser Leu Glu Pro Val Thr Ile Thr Met Pro Asp Leu Arg
o
5' CCG AAC ATG TAT CAT CCG ACC GGT GAT CCG GGC CTG GTG CCG ACC
o                                                               900
1  Pro Asn Met Tyr His Pro Thr Gly Asp Pro Gly Leu Val Pro Thr
o
5' CTG GTG CTG AGC GTT TAT AAC AAT CTG ATC AAC CCG TTT GGT GGC
o                                                               945
1  Leu Val Leu Ser Val Tyr Asn Asn Leu Ile Asn Pro Phe Gly Gly
o
5' AGC ACC AGC GCC ATT CAG GTT ACC GTT GAA ACC AGG CCG AGC GAA
o                                                               990
1  Ser Thr Ser Ala Ile Gln Val Thr Val Glu Thr Arg Pro Ser Glu
o
5' GAT TTT GAA TTT GTG ATG ATT AGG ACC CCG AGC AGC AAA ACC GTG
o                                                               1035
1  Asp Phe Glu Phe Val Met Ile Arg Thr Pro Ser Ser Lys Thr Val
o
5' GAT AGC ATT TAT CCG GCG GGC CTG CTG ACC ACC CCG GTG CTG ACC
o                                                               1080
1  Asp Ser Ile Tyr Pro Ala Gly Leu Leu Thr Thr Pro Val Leu Thr
o
5' GGT GTG GGC AAC GAT AAC AGG TGG AAT GGT CAG ATC GTG GGC CTG
o                                                               1125
1  Gly Val Gly Asn Asp Asn Arg Trp Asn Gly Gln Ile Val Gly Leu
o
5' CAG CCG GTT CCG GGT GGT TTT AGC ACC TGC AAT AGG CAT TGG AAC
o                                                               1170
1  Gln Pro Val Pro Gly Gly Phe Ser Thr Cys Asn Arg His Trp Asn
o
5' CTG AAC GGT AGC ACC TAT GGT TGG AGC AGC CCG AGG TTT GCG GAT
o                                                               1215
1  Leu Asn Gly Ser Thr Tyr Gly Trp Ser Ser Pro Arg Phe Ala Asp
o
5' ATT GAT CAT AGG AGG GGC AGC GCG AGC TAT CCG GGT AGC AAC GCG
o                                                               1260
1  Ile Asp His Arg Arg Gly Ser Ala Ser Tyr Pro Gly Ser Asn Ala
o
5' ACC AAT GTT CTG CAG TTT TGG TAT GCC AAT GCG GGC AGC GCG ATT
o                                                               1305
1  Thr Asn Val Leu Gln Phe Trp Tyr Ala Asn Ala Gly Ser Ala Ile
o
5' GAT AAC CCG ATT AGC CAG GTG GCC CCG GAT GGT TTT CCG GAT ATG
o                                                               1350
1  Asp Asn Pro Ile Ser Gln Val Ala Pro Asp Gly Phe Pro Asp Met
o
```

5' AGC TTT GTG CCG TTT AAT GGC CCG GGC ATC CCG GCG GCC GGT TGG
o 1395
1 Ser Phe Val Pro Phe Asn Gly Pro Gly Ile Pro Ala Ala Gly Trp
o

5' GTG GGC TTT GGT GCC ATT TGG AAC AGC AAT AGC GGT GCG CCG AAC
o 1440
1 Val Gly Phe Gly Ala Ile Trp Asn Ser Asn Ser Gly Ala Pro Asn
o

5' GTG ACC ACC GTT CAG GCG TAT GAA CTG GGC TTT GCG ACC GGT GCG
o 1485
1 Val Thr Thr Val Gln Ala Tyr Glu Leu Gly Phe Ala Thr Gly Ala
o

5' CCG GGC AAT CTG CAG CCG ACC ACC AAC ACC AGC GGC AGC CAG ACC
o 1530
1 Pro Gly Asn Leu Gln Pro Thr Thr Asn Thr Ser Gly Ser Gln Thr
o

5' GTG GCG AAA AGC ATT TAT GCC GTT GTT ACC GGT ACC GCG CAG AAT
o 1575
1 Val Ala Lys Ser Ile Tyr Ala Val Val Thr Gly Thr Ala Gln Asn
o

5' CCG GCG GGC CTG TTT GTT ATG GCG AGC GGC GTG ATT AGC ACC CCG
o 1620
1 Pro Ala Gly Leu Phe Val Met Ala Ser Gly Val Ile Ser Thr Pro
o

5' AGC GCC AAT GCC ATC ACC TAT ACC CCG CAG CCG GAT AGG ATC GTG
o 1665
1 Ser Ala Asn Ala Ile Thr Tyr Thr Pro Gln Pro Asp Arg Ile Val
o

5' ACC ACC CCG GGC ACC CCG GCG GCG GCG CCG GTT GGC AAA AAT ACC
o 1710
1 Thr Thr Pro Gly Thr Pro Ala Ala Ala Pro Val Gly Lys Asn Thr
o

5' CCG ATT ATG TTC GCC AGC GTT GTT AGG AGG ACC GGT GAT GTT AAC
o 1755
1 Pro Ile Met Phe Ala Ser Val Val Arg Arg Thr Gly Asp Val Asn
o

5' GCG ACC GCG GGC AGC GCG AAT GGC ACC CAG TAT GGC ACC GGC AGC
o 1800
1 Ala Thr Ala Gly Ser Ala Asn Gly Thr Gln Tyr Gly Thr Gly Ser
o

5' CAG CCG CTG CCG GTG ACC ATC GGT CTG AGC CTG AAC AAT TAT AGC
o 1845
1 Gln Pro Leu Pro Val Thr Ile Gly Leu Ser Leu Asn Asn Tyr Ser
o

5' AGC GCG CTG ATG CCG GGT CAG TTC TTC GTG TGG CAG CTG ACC TTT
o 1890
1 Ser Ala Leu Met Pro Gly Gln Phe Phe Val Trp Gln Leu Thr Phe
o

5' GCC AGC GGT TTT ATG GAA ATT GGC CTG AGC GTG GAT GGC TAT TTT
o 1935
1 Ala Ser Gly Phe Met Glu Ile Gly Leu Ser Val Asp Gly Tyr Phe
o

5' TAT GCG GGC ACC GGT GCG AGC ACC ACC CTG ATT GAT CTG ACC GAA
o 1980
1 Tyr Ala Gly Thr Gly Ala Ser Thr Thr Leu Ile Asp Leu Thr Glu
o

5' CTG ATT GAT GTT AGG CCG GTT GGT CCG AGG CCG AGC AAA AGC ACC
o 2025
1 Leu Ile Asp Val Arg Pro Val Gly Pro Arg Pro Ser Lys Ser Thr
o

5' CTG GTT TTT AAC CTG GGC GGT ACC GCG AAC GGT TTT AGC TAT GTG
o 2070
1 Leu Val Phe Asn Leu Gly Gly Thr Ala Asn Gly Phe Ser Tyr Val
o

5' AGC GAA AAA GAT GAA CTG TAA
o 2091
1 Ser Glu Lys Asp Glu Leu .
o
