# Supplementary material for: Reduced evolvability of Escherichia coli MDS42, an IS-less cellular chassis for molecular and synthetic biology applications
Source: Microb Cell Fact. 2010 May 21;9:38. doi: 10.1186/1475-2859-9-38 (PMC2891674; doi:10.1186/1475-2859-9-38)
Supplement: Additional file 4 — Structure predicitions for ORF238. [file 1475-2859-9-38-S4.PDF]

Peptide sequence used in queries:

```
>238aa Leu-rich pCTXVP60
MLLLLFLWLMDQDPWKERLELLHKLELLELLLLLLFQELLLMEWTQELLLLLLL
LLLKILLLLLLLLELEDHHNKLINKKLGELIFIIMMFLLGLLLMLQDLFFILFNILHK
IIHSLLFFLKCMLDGLEECNSDSLLLDLEFLEEDLLLLLFHQELKLDQVLKLDNFH
MLLLMLGLLNQLLLLCQTLDQICIIQLEIQDLFQLLFFLFIIILLIHLEDLLLLFKLLL
KLDHLKILNLL
```

DAS-TMfilter prediction results 2/8/2010

```
Calculating prediction for the following proteins
with reference library 08:

>238aa Leu-rich pCTXVP60

... Done.

*** List of predicted non-TM-protein codes ***

none

*** List of predicted TM-protein codes ***

>238aa Leu-rich pCTXVP60


=== Result of the prediction ===

>238aa Leu-rich pCTXVP60
# TMH:  6 Q: trusted
@   33   4.915 core:   26 ..   41 1.003e-05
@   57   9.401 core:   46 ..   68 1.332e-12
@   92   6.834 core:   83 ..  121 1.145e-08 Twin peaks - two TMH with a
short linker
@  109   3.572 core:   83 ..  121 1.148e-03
@  173   4.243 core:  168 ..  183 1.073e-04
@  210   7.788 core:  200 ..  227 3.957e-10

<-------- end of list -------->
```

HMMTOP version 2.0 prediction results

```
Protein: 238aa Leu-rich pCTXVP60
Length:  238
N-terminus:  IN
Number of transmembrane helices: 4
Transmembrane helices: 48-67 84-107 167-184 203-222

Total entropy of the model:  17.0012
Entropy of the best path:  17.0026

The best path:

     seq  MLLLLFLWLM DQDPWKERLE LLHKLELLEL LLLLLFQELL LMEWTQELLL    50
     pred IIIIIIIIII IIIIIIIIII IIIIIIIIII IIiiiiiiii iiiiiiiHHH

     seq  LLLLLLLKIL LLLLLLLELE DHHNKLINKK LGELIFIIMM FLLGLLLMLQ   100
     pred HHHHHHHHHH HHHHHHHooo oooooooooo oooHHHHHHH HHHHHHHHHH

     seq  DLFFILFNIL HKIIHSLLFF LKCMLDGLEE CNSDSLLLDL EFLEEDLLLL   150
     pred HHHHHHHiii iiiiiiiiii iiIIIIIIII IIIIIIIIII IIIIIIIIII

     seq  LFHQELKLDQ VLKLDNFHML LLMLGLLNQL LLLCQTLDQI CIIQLEIQDL   200
     pred Iiiiiiiiii iiiiiiHHHH HHHHHHHHHH HHHHoooooo oooooooooo

     seq  FQLLFFLFII ILLIHLEDLL LLFKLLLKLD HLKILNLL  238
     pred ooHHHHHHHH HHHHHHHHHH HHiiiiiiii iiiiiiiI
```
