# Supplementary material for: Reduced evolvability of Escherichia coli MDS42, an IS-less cellular chassis for molecular and synthetic biology applications
Source: Microb Cell Fact. 2010 May 21;9:38. doi: 10.1186/1475-2859-9-38 (PMC2891674; doi:10.1186/1475-2859-9-38)
Supplement: Additional file 5 — Relationship of reads, IS elements, genome segments and gene annotations. [file 1475-2859-9-38-S5.PDF]

TX1352 Enterococcus faecium

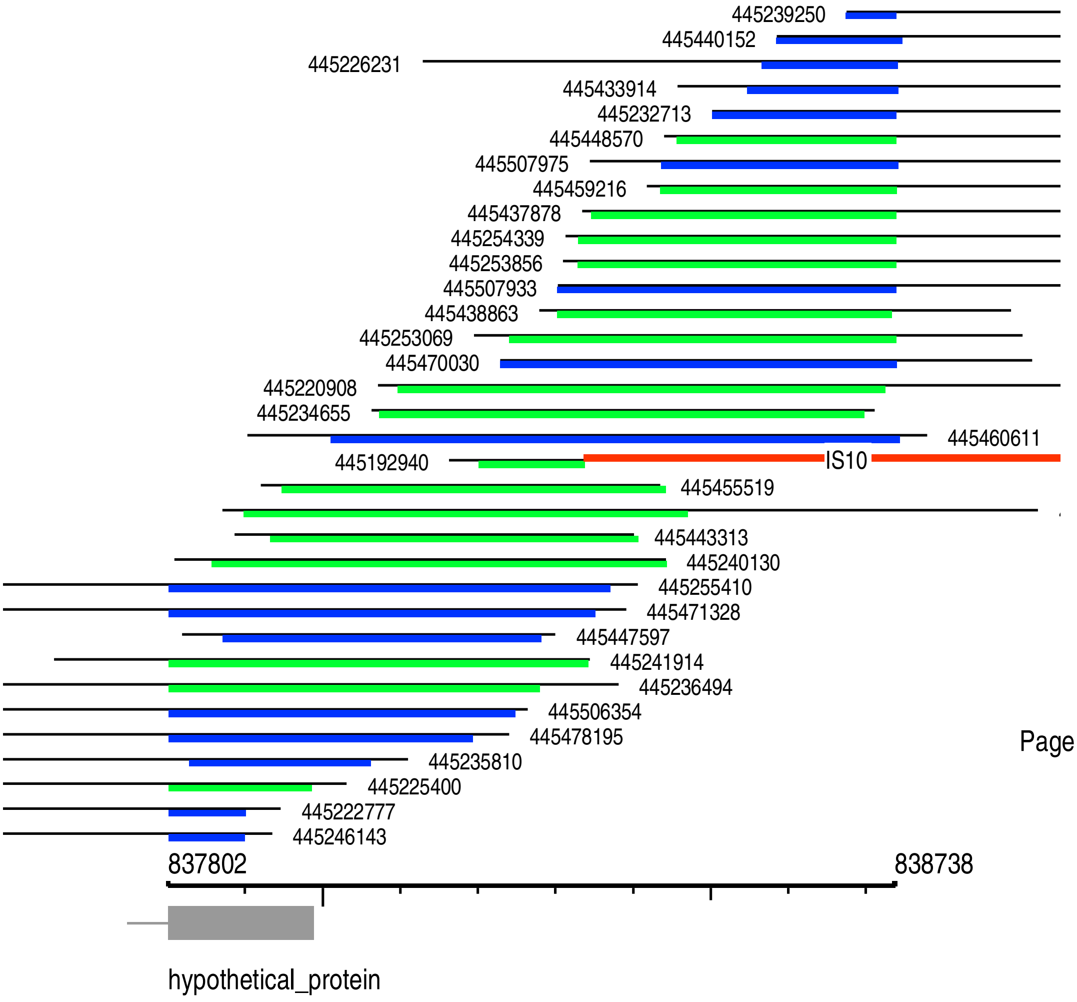

Page: 2

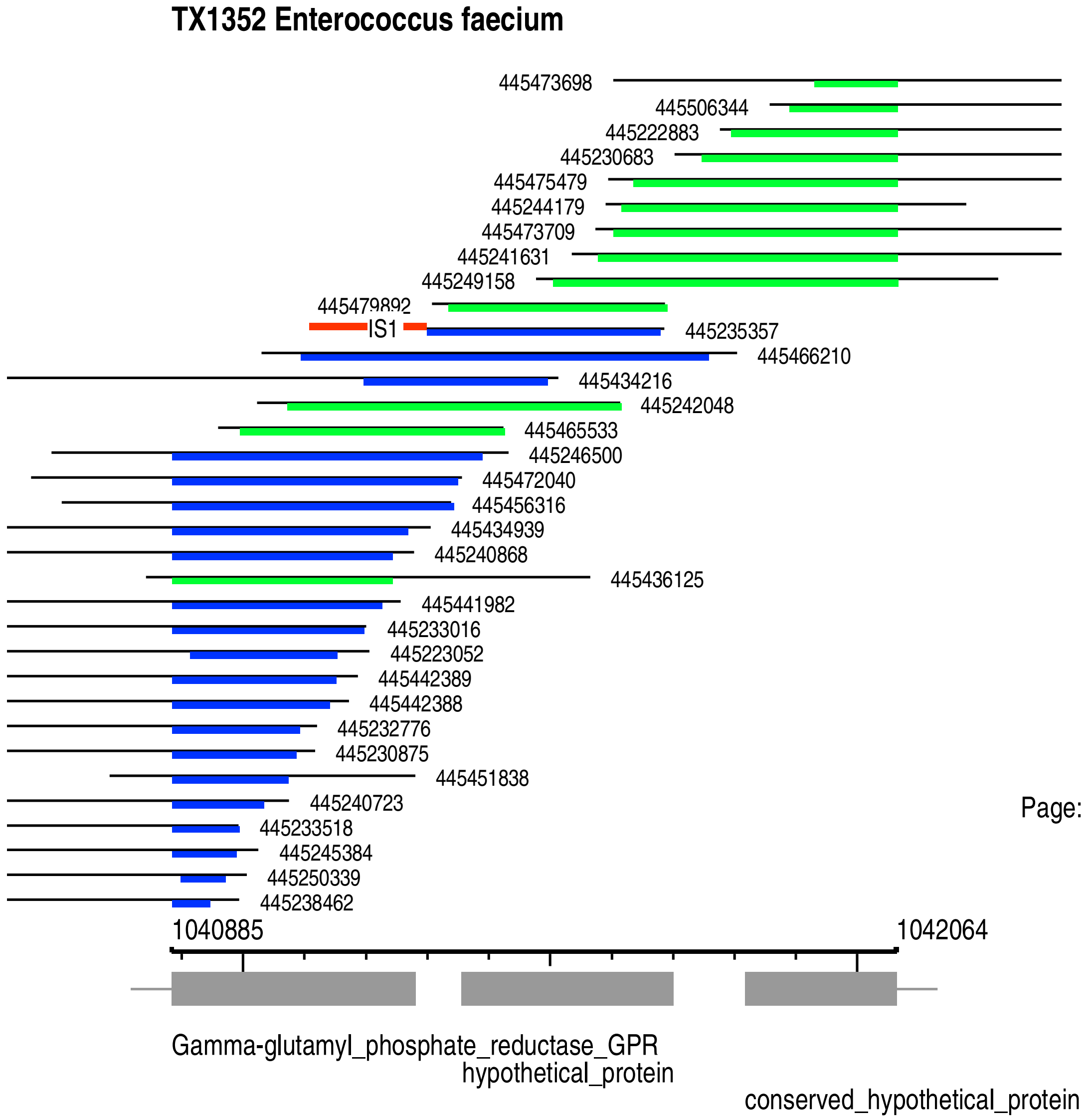
TX1352 Enterococcus faecium
445473698
445506344
445222883
445230683
445475479
445244179
445473709
445241631
445249158
445479892
IS1
445235357
445466210
445434216
445242048
445465533
445246500
445472040
445456316
445434939
445240868
445436125
445441982
445233016
445223052
445442389
445442388
445232776
445230875
445451838
445240723
445233518
445245384
445250339
445238462
1040885
1042064
Gamma-glutamyl_phosphate_reductase_GPR
hypothetical_protein
conserved_hypothetical_protein

TX1352 Enterococcus faecium

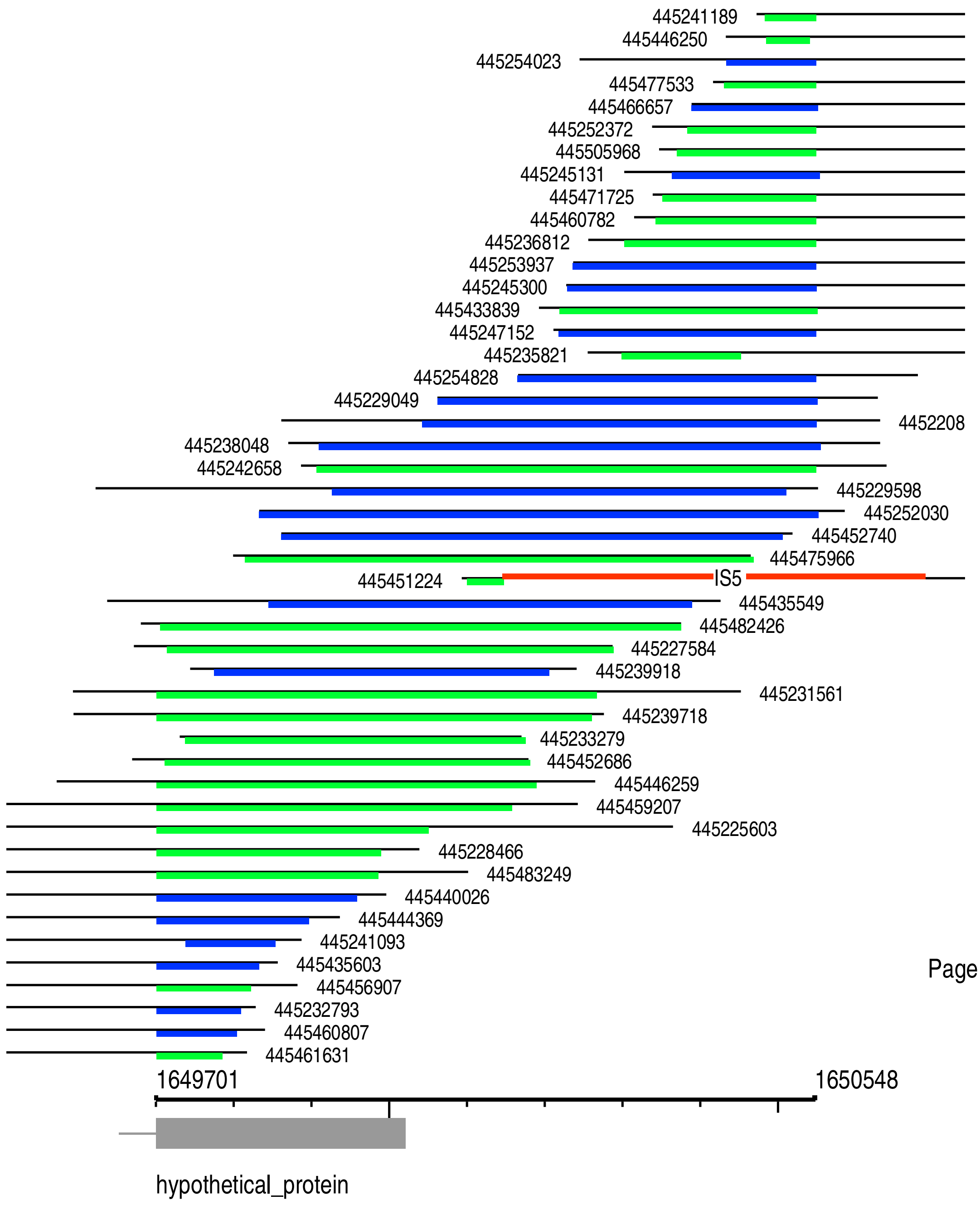

Page: 4

TX1352 Enterococcus faecium

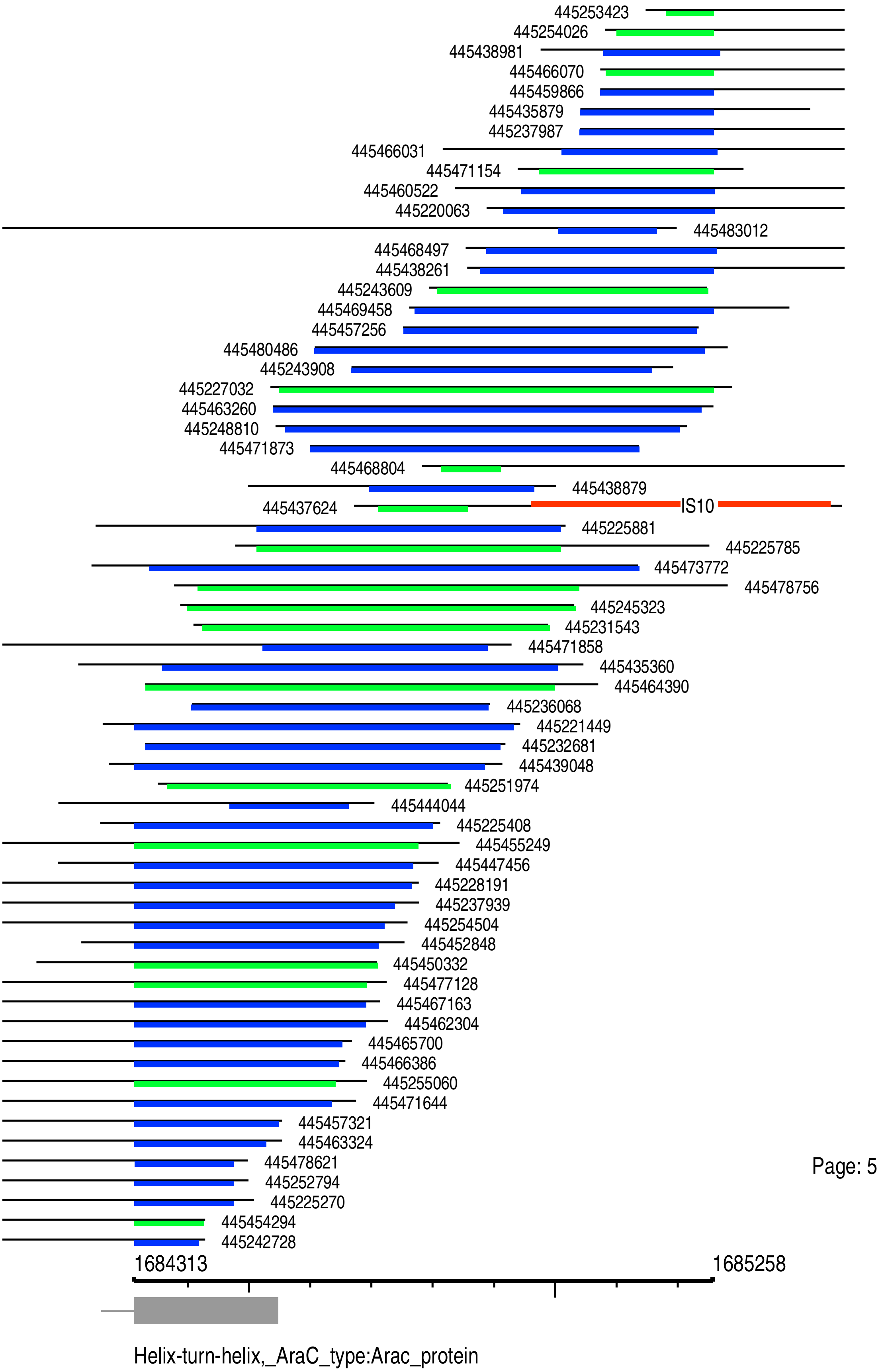

TX1352 Enterococcus faecium

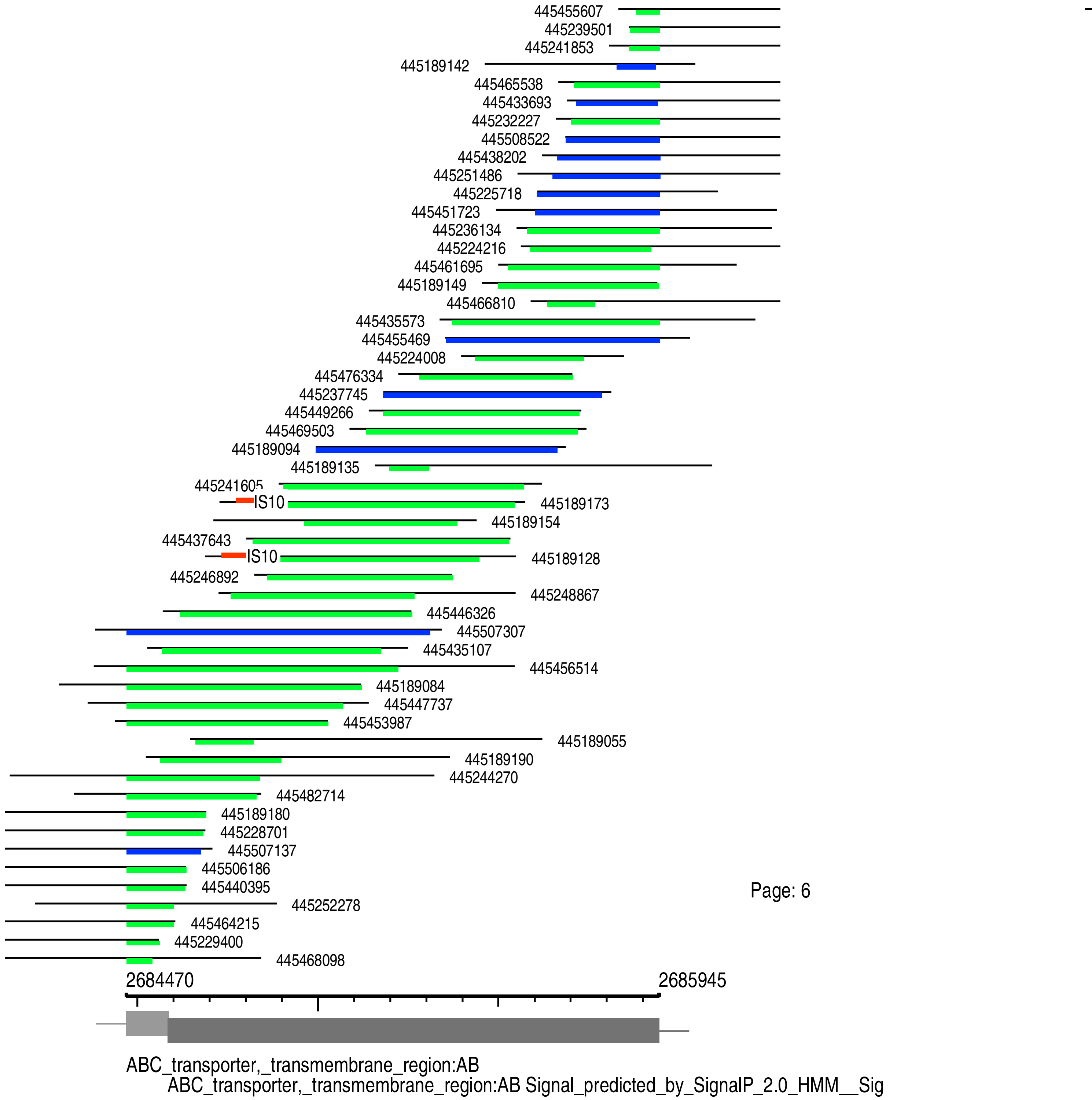

TX1352 Enterococcus faecium

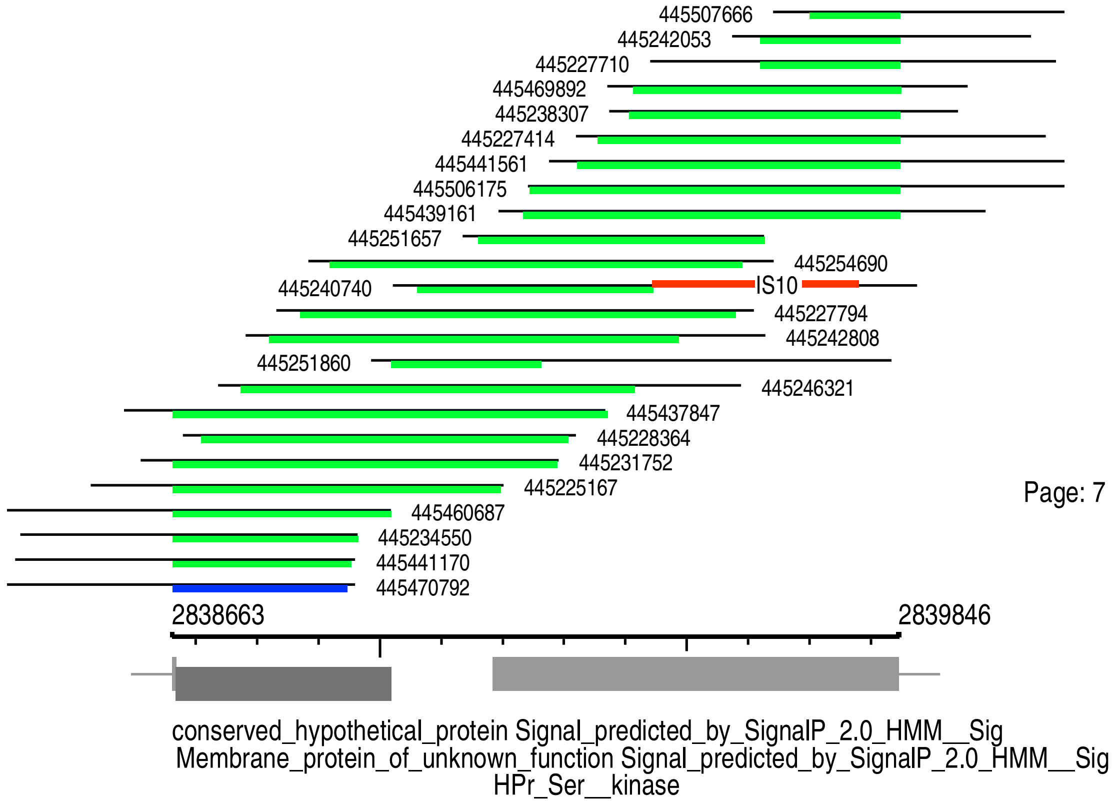

TX160488 Pseudomonas putida kt2440

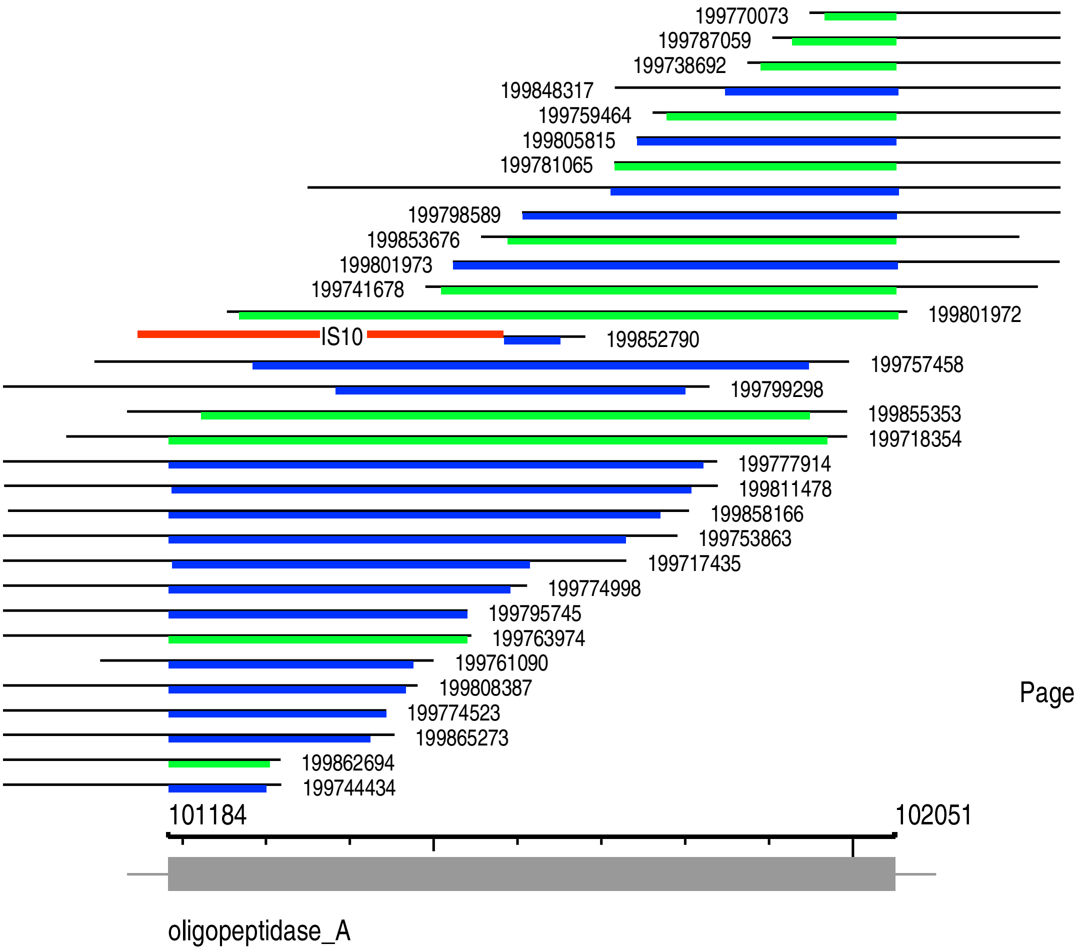

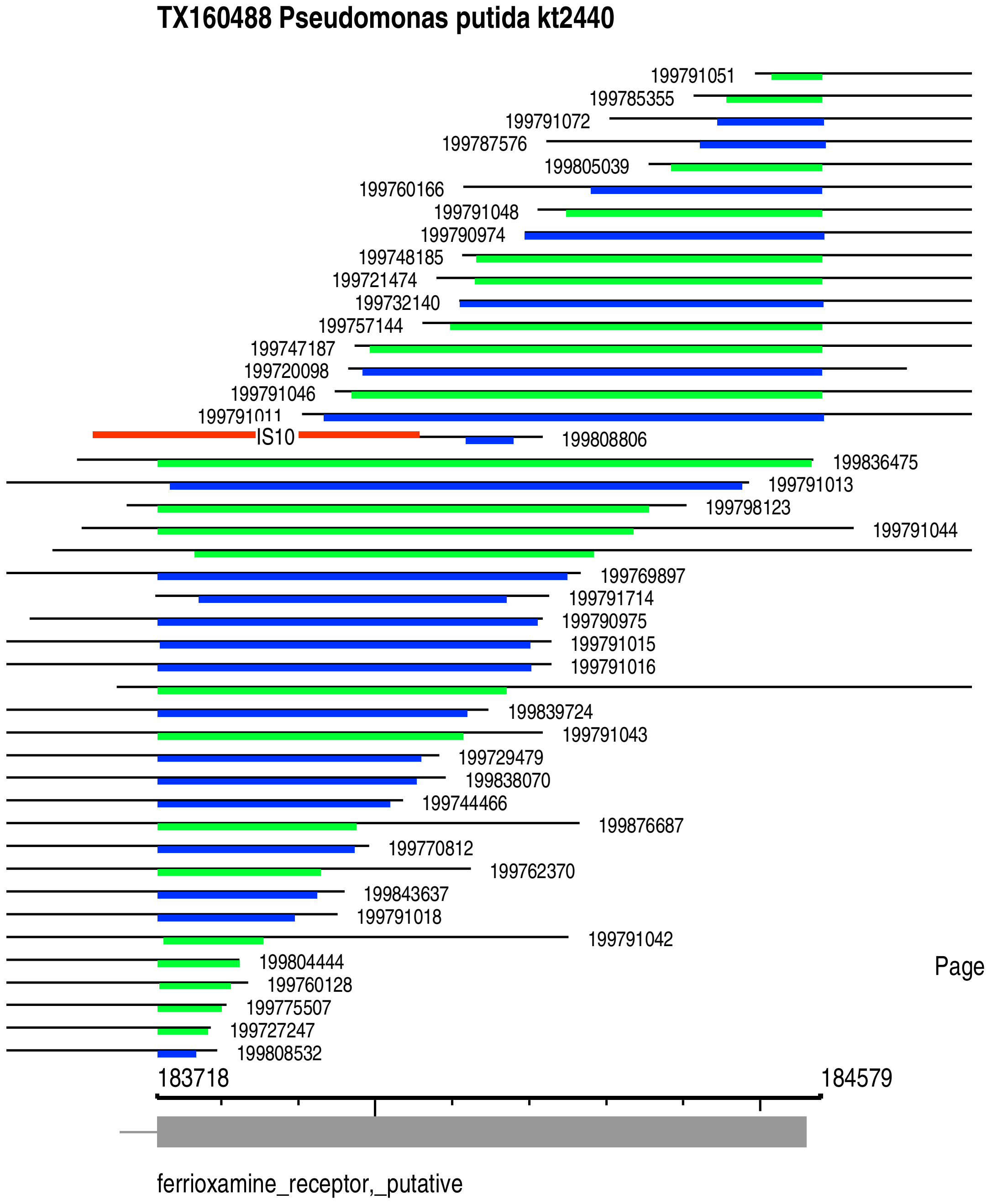
TX160488 Pseudomonas putida kt2440
199791051
199785355
199791072
199787576
199805039
199760166
199791048
199790974
199748185
199721474
199732140
199757144
199747187
199720098
199791046
199791011
IS10
199808806
199836475
199791013
199798123
199791044
199769897
199791714
199790975
199791015
199791016
199839724
199791043
199729479
199838070
199744466
199876687
199770812
199762370
199843637
199791018
199791042
199804444
199760128
199775507
199727247
199808532
183718
184579
ferrioxamine_receptor,_putative

Page: 9

TX160488 Pseudomonas putida kt2440

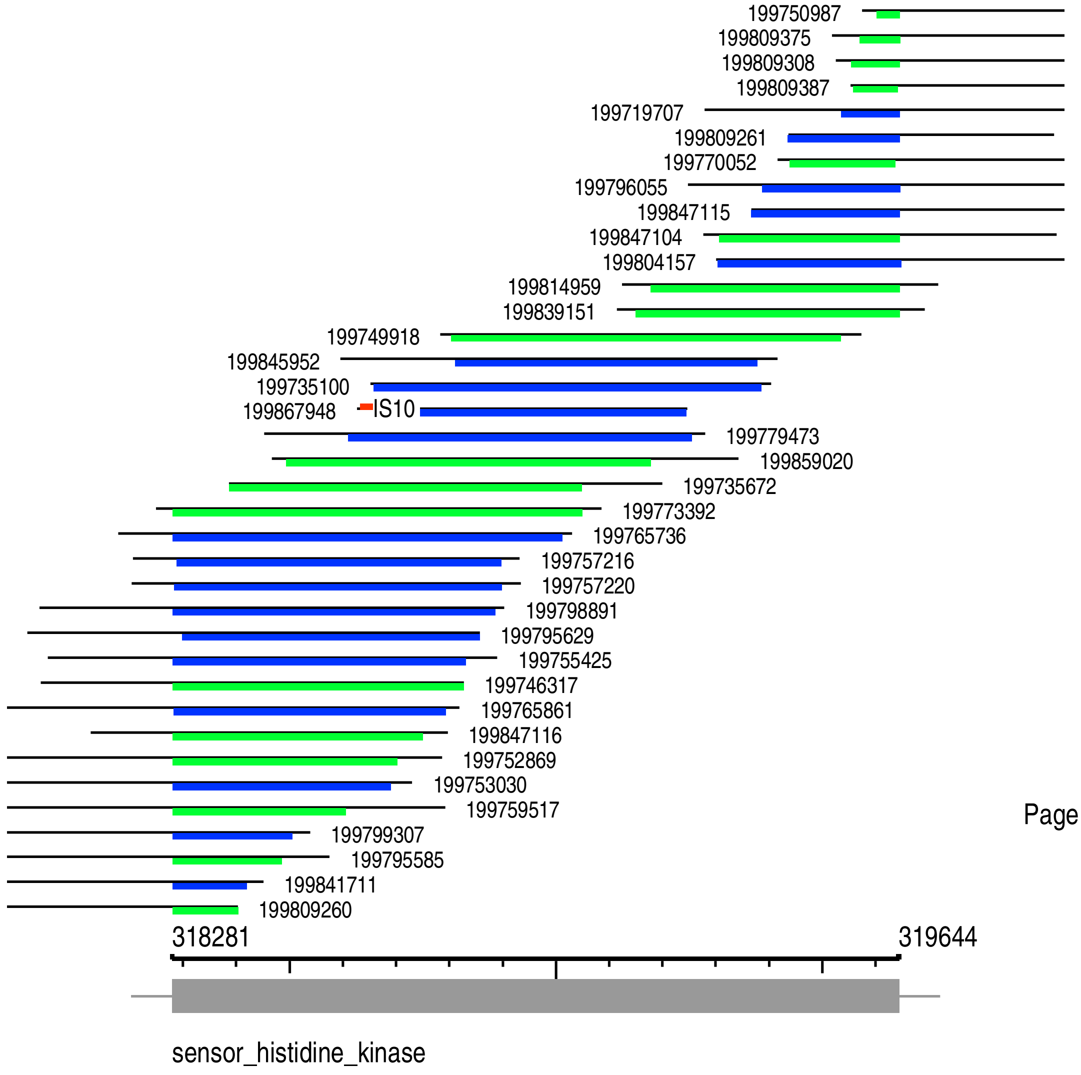

TX160488 Pseudomonas putida kt2440

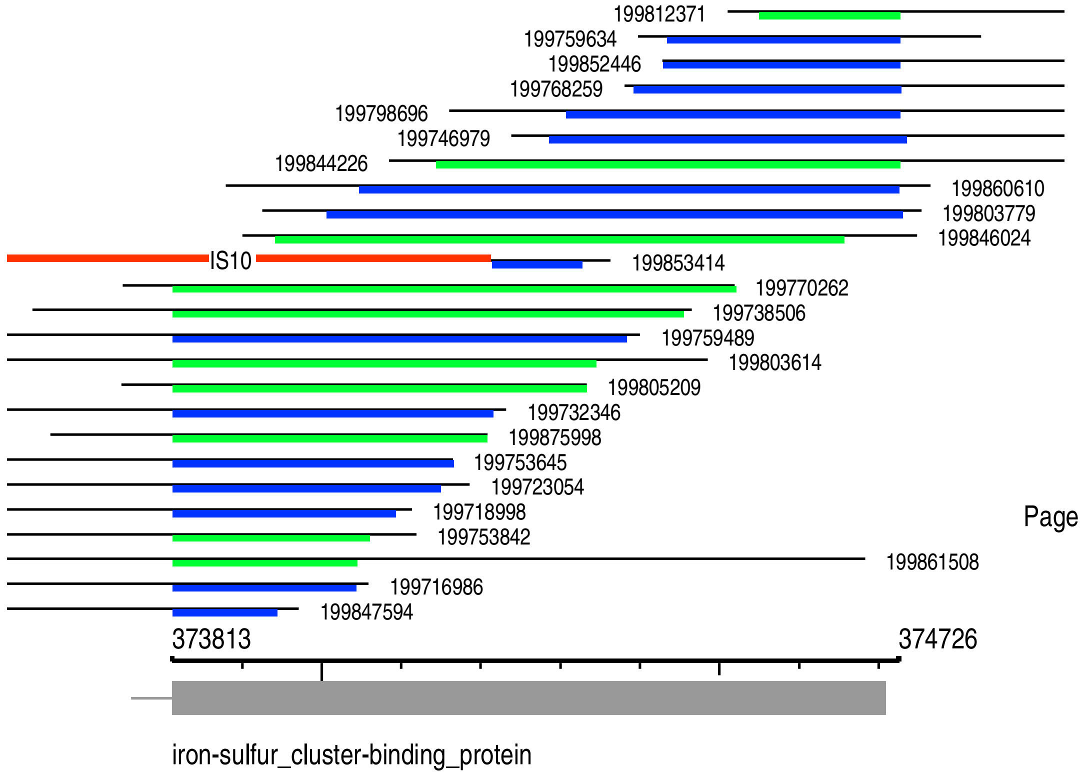

TX160488 Pseudomonas putida kt2440

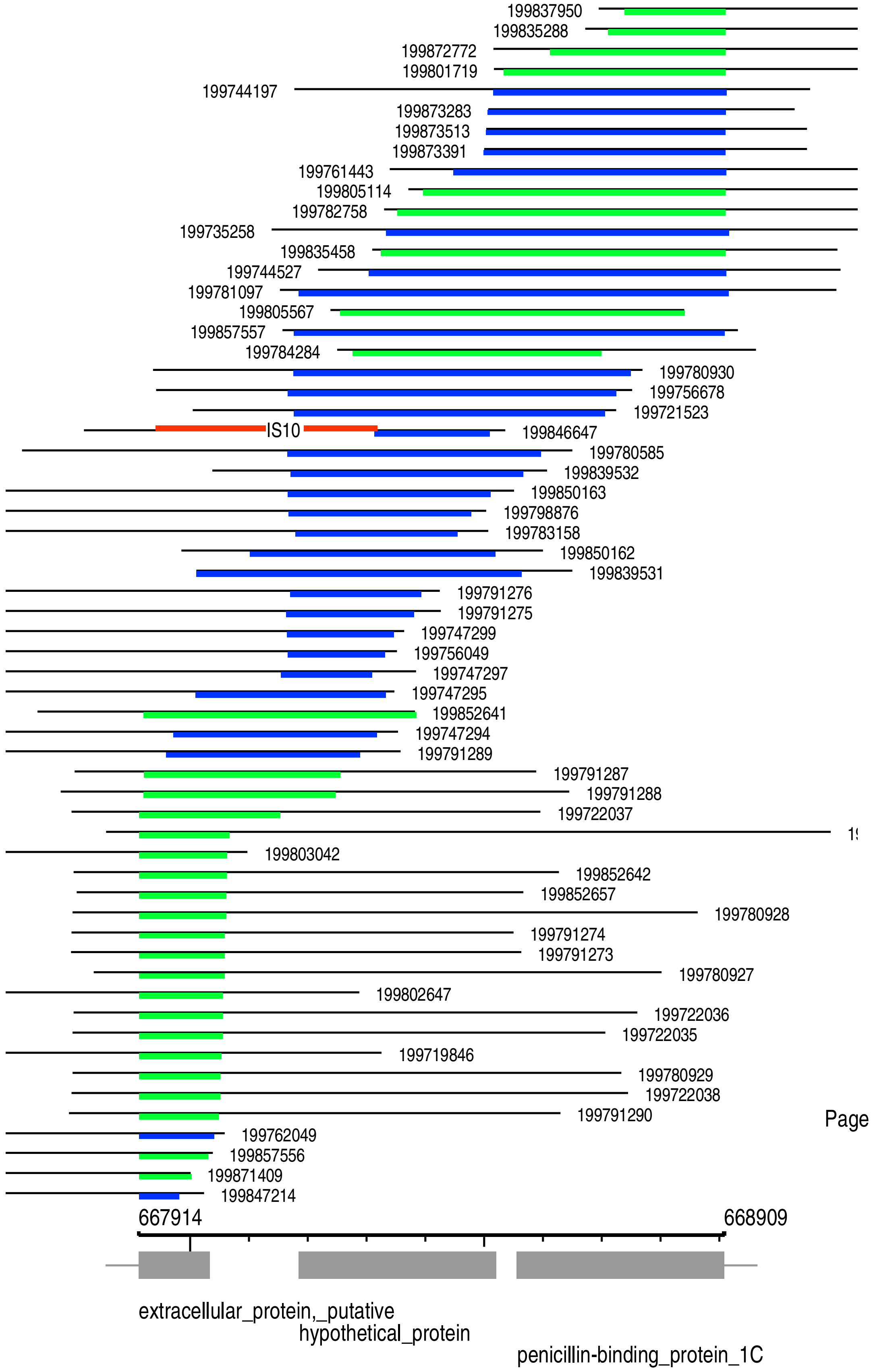

TX160488 Pseudomonas putida kt2440

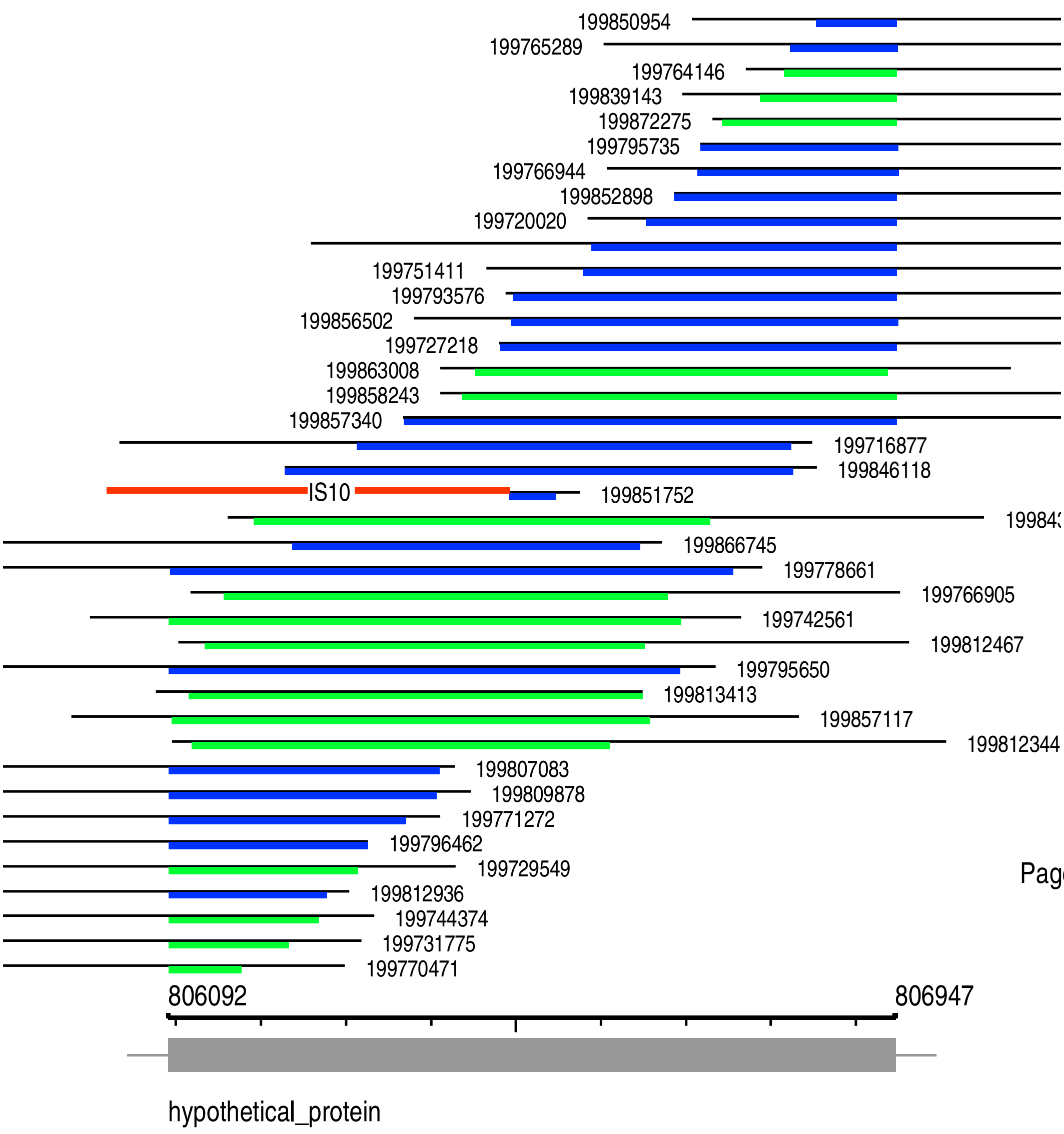

TX160488 Pseudomonas putida kt2440

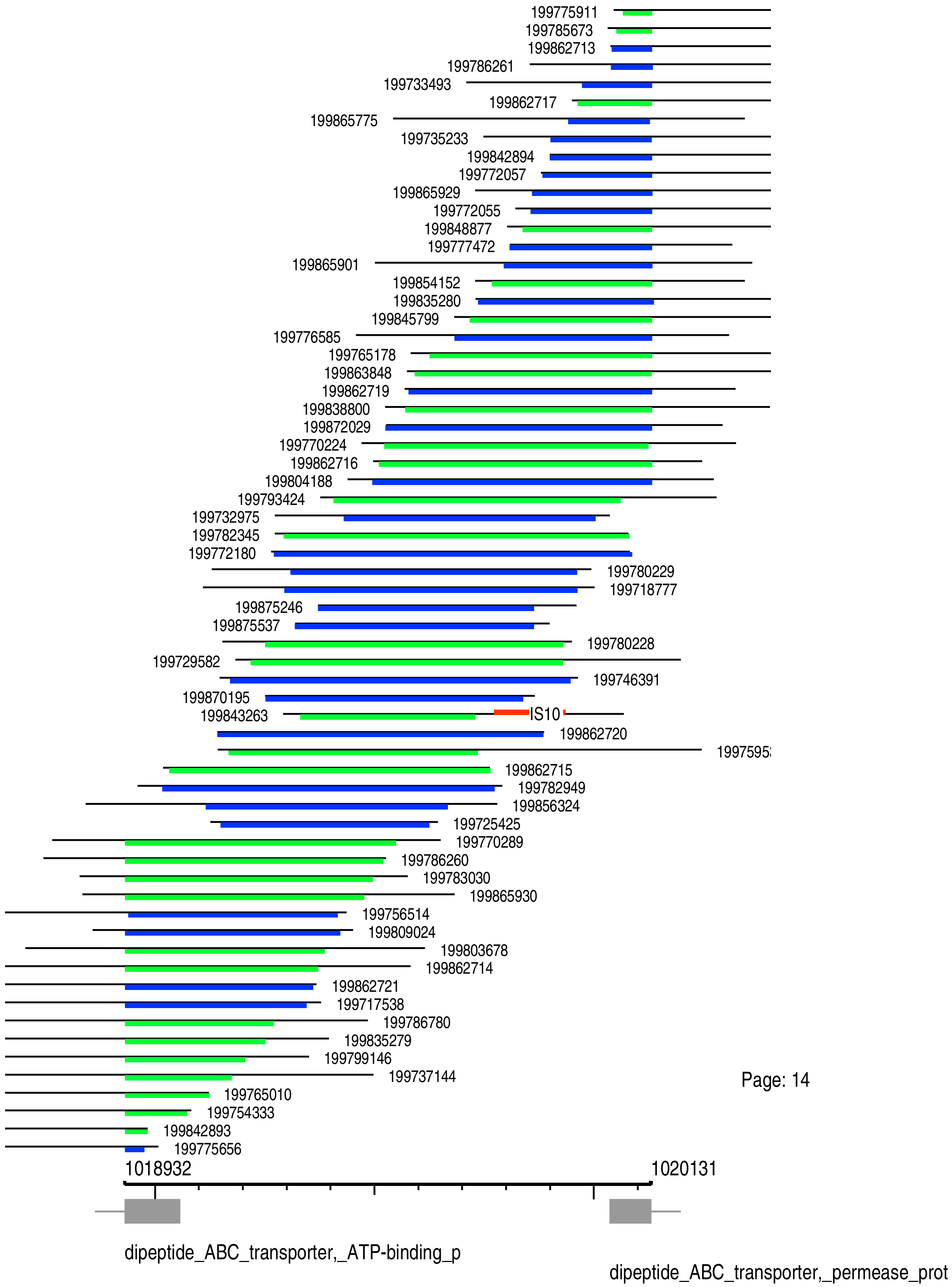

TX160488 Pseudomonas putida kt2440

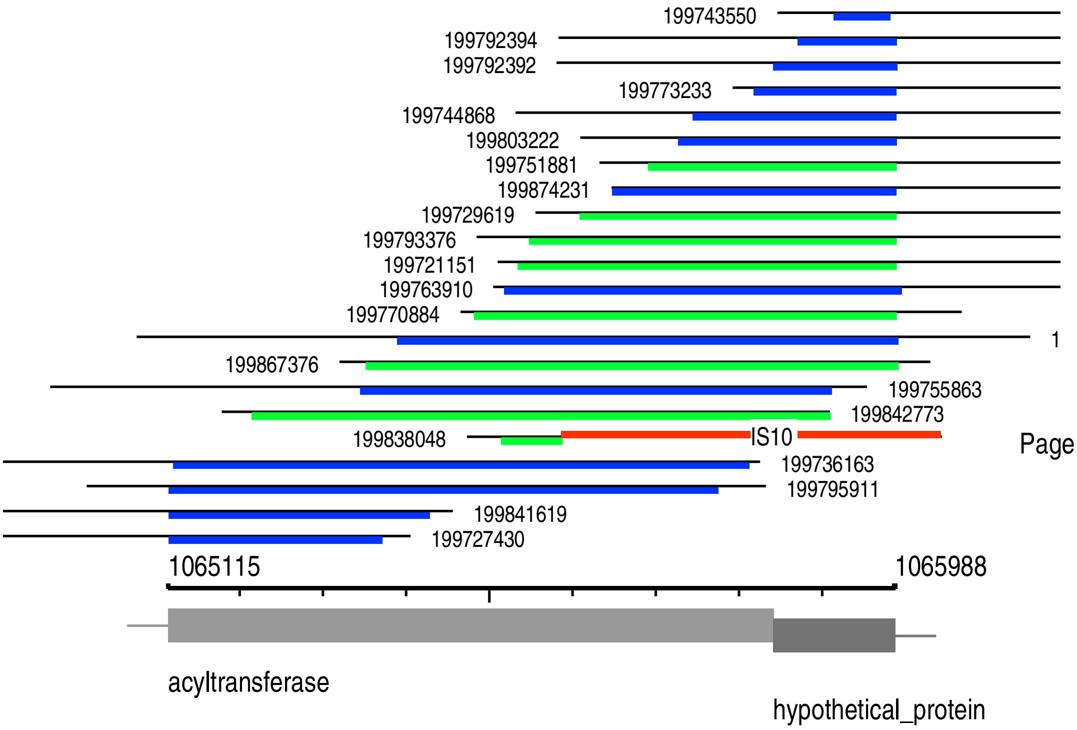

Page: 15

TX160488 Pseudomonas putida kt2440

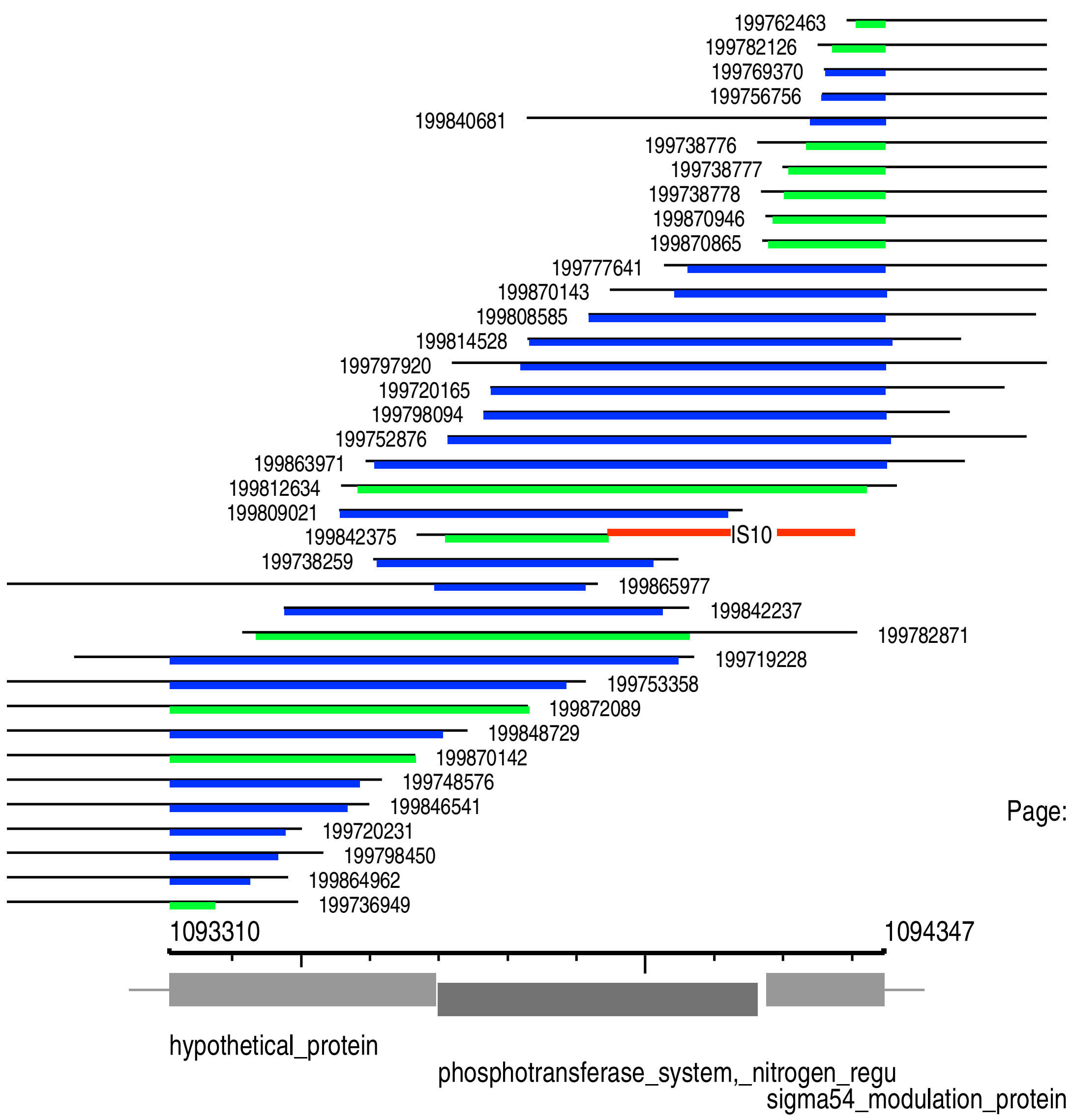

Page: 16

TX160488 Pseudomonas putida kt2440

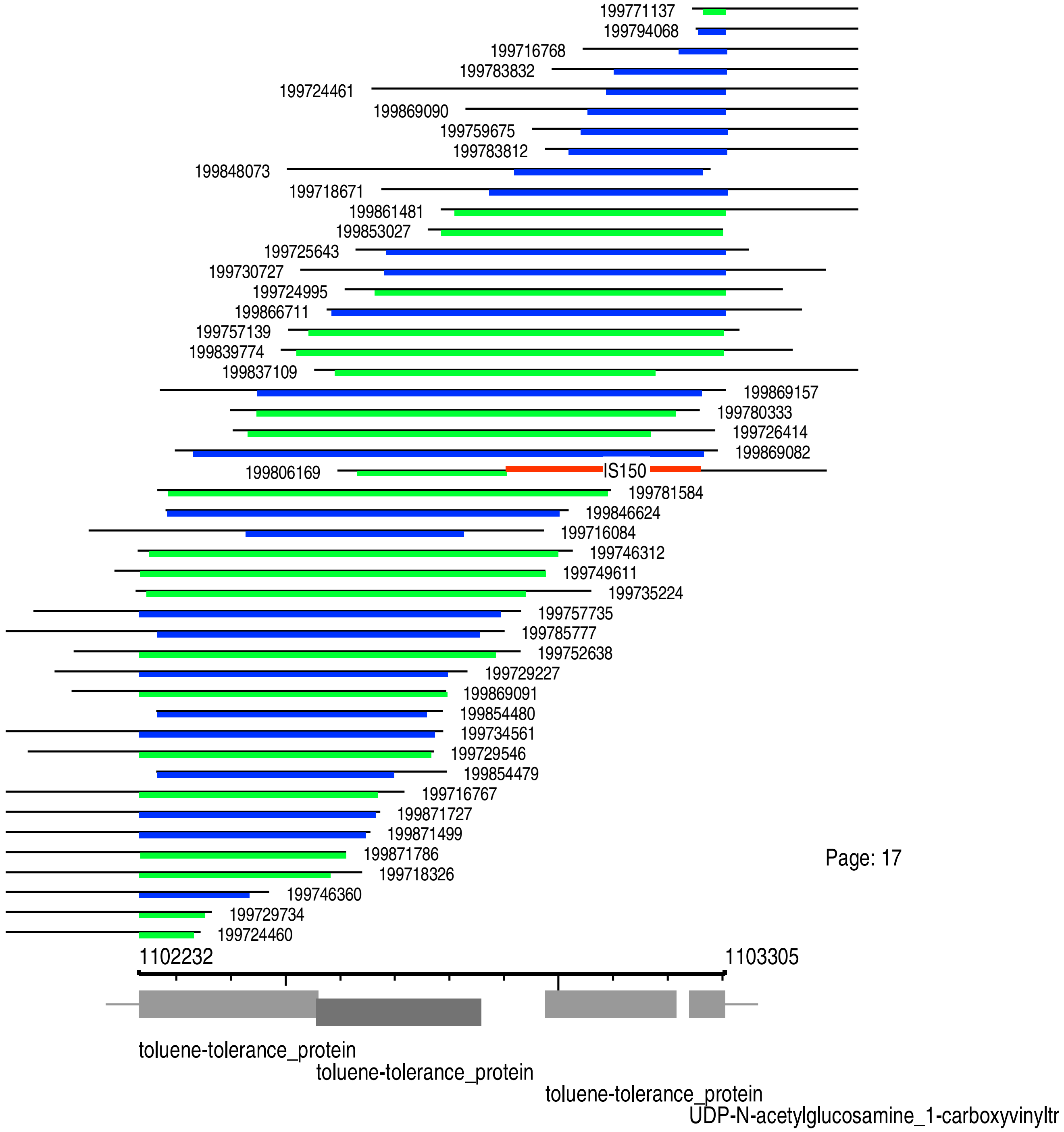

TX160488 Pseudomonas putida kt2440

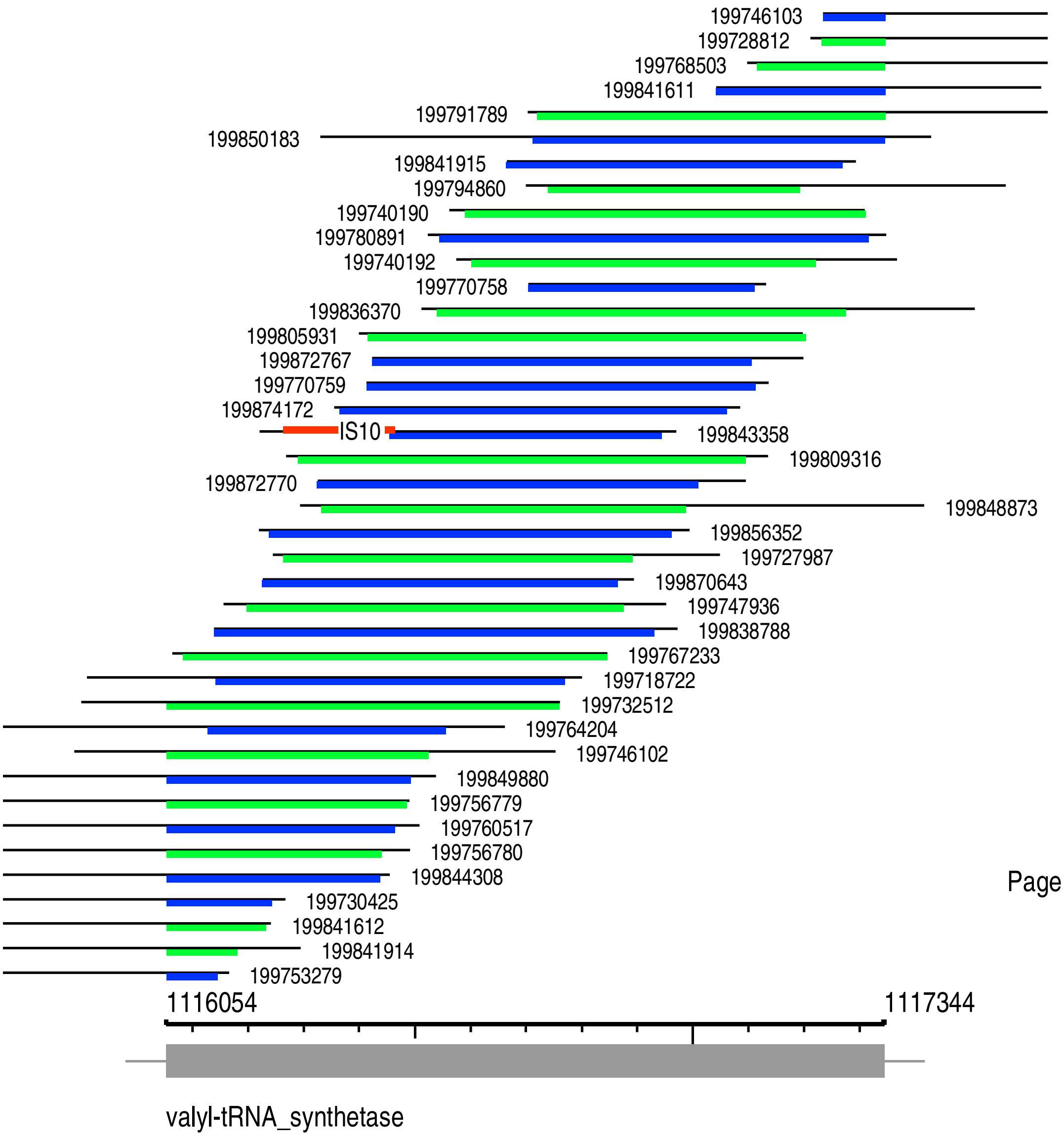

Page: 18

TX160488 Pseudomonas putida kt2440

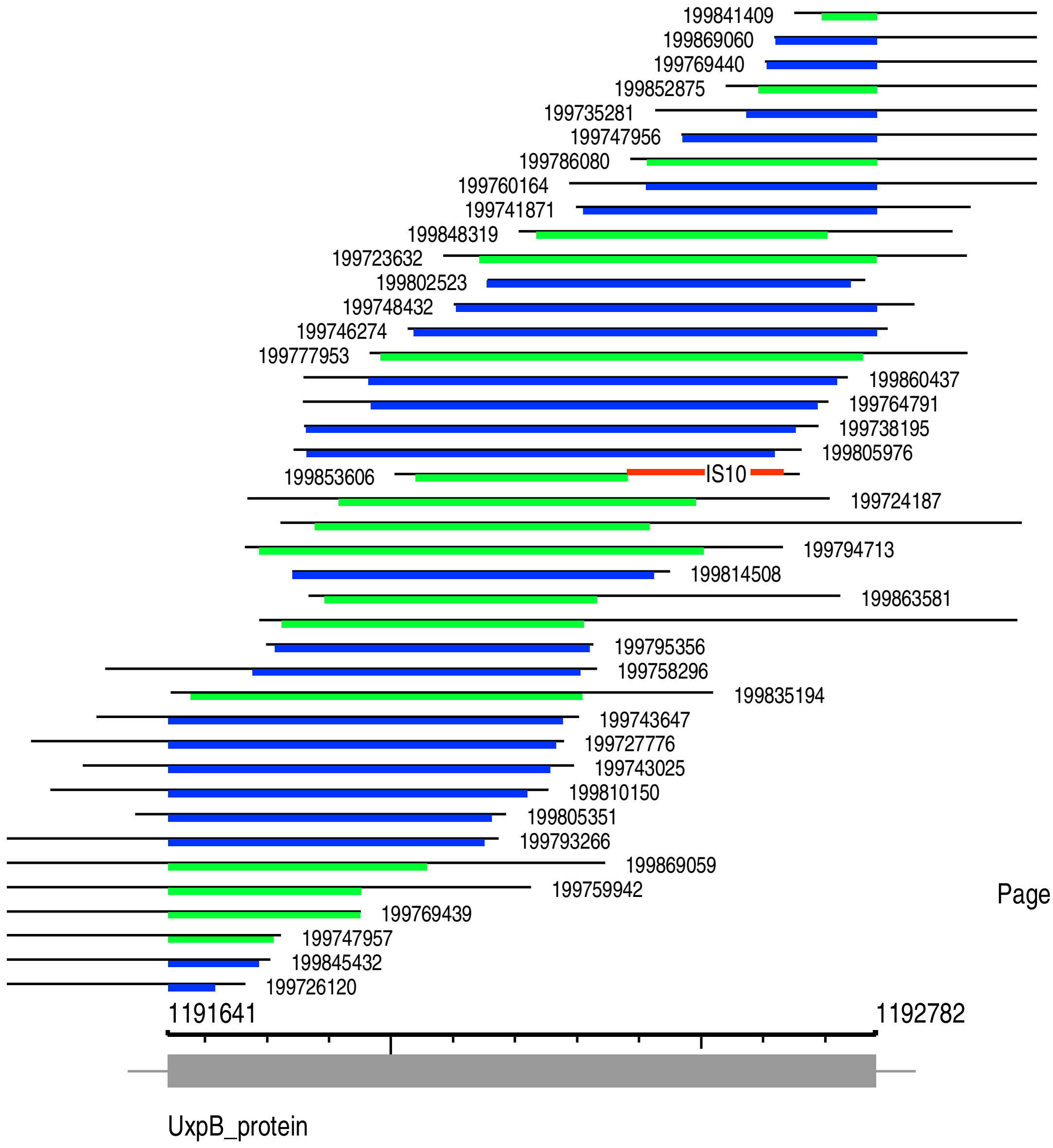

TX160488 Pseudomonas putida kt2440

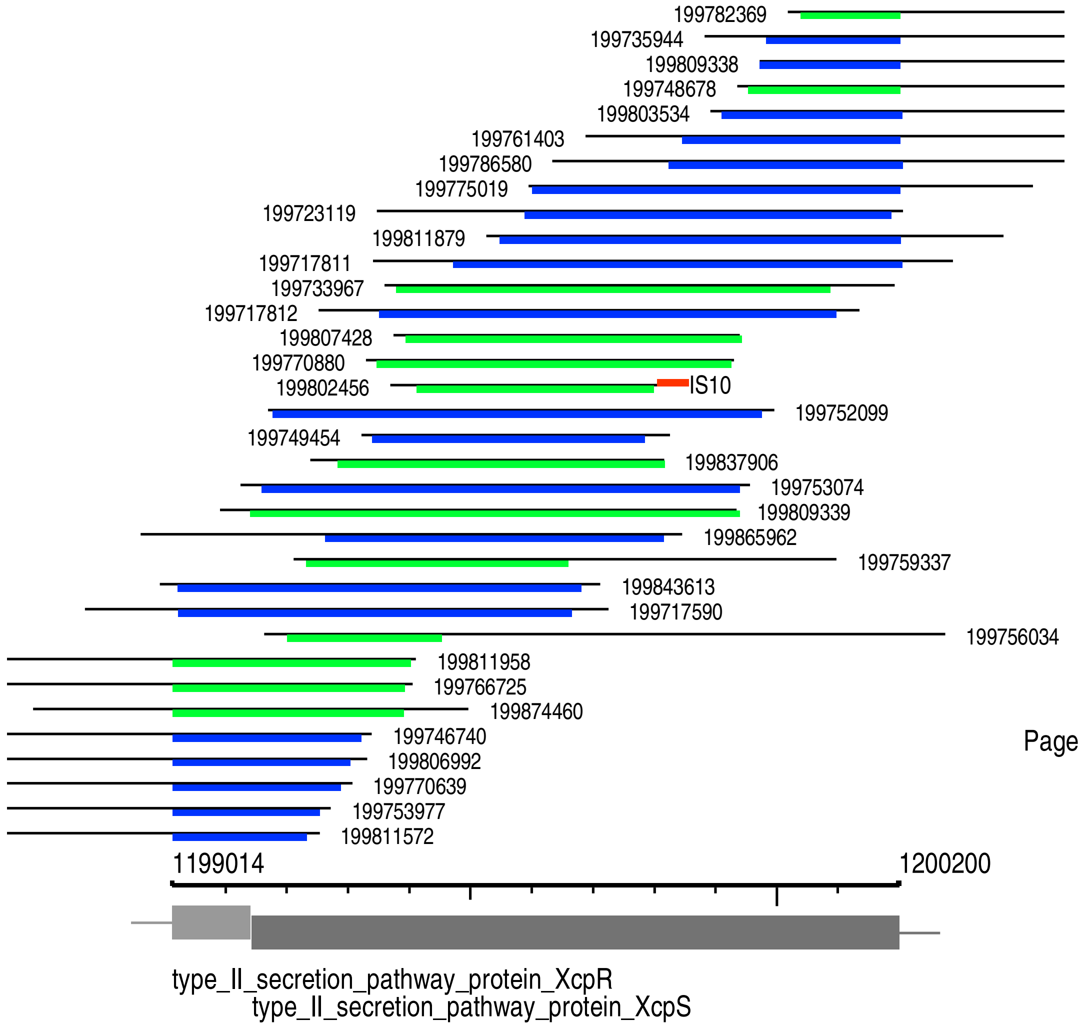

Page: 20

TX160488 Pseudomonas putida kt2440

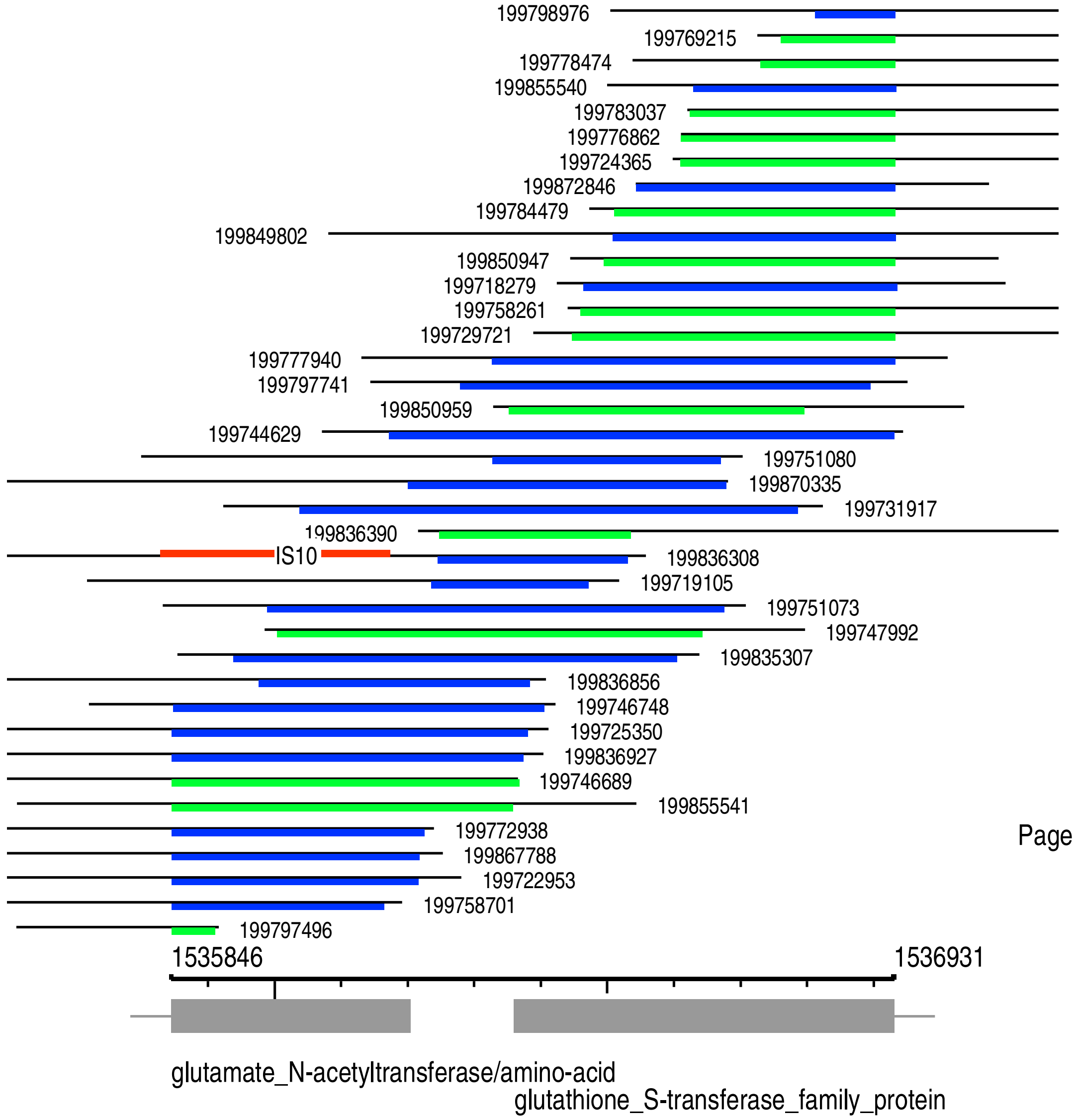

Page: 21

TX160488 Pseudomonas putida kt2440

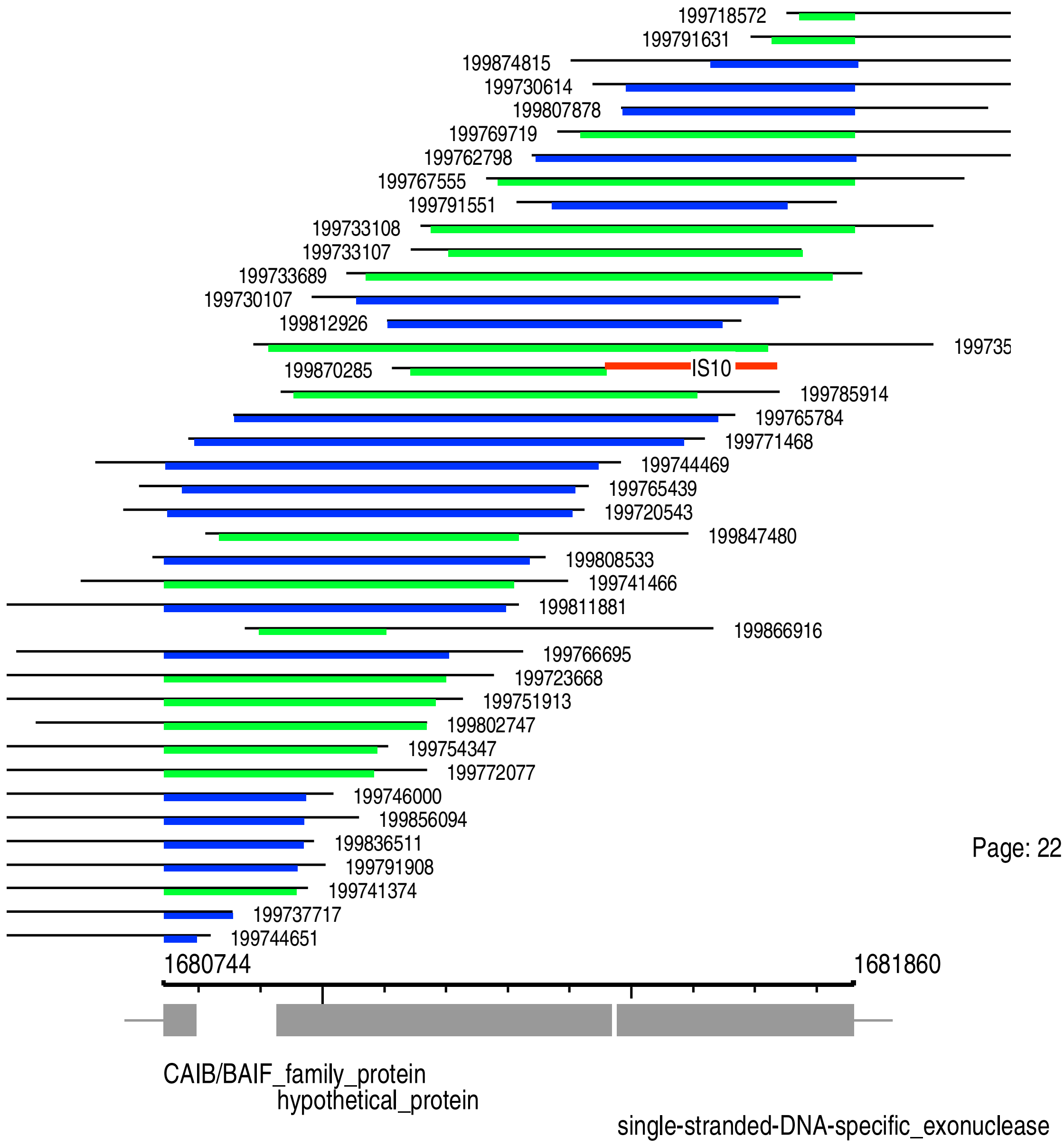

TX160488 Pseudomonas putida kt2440

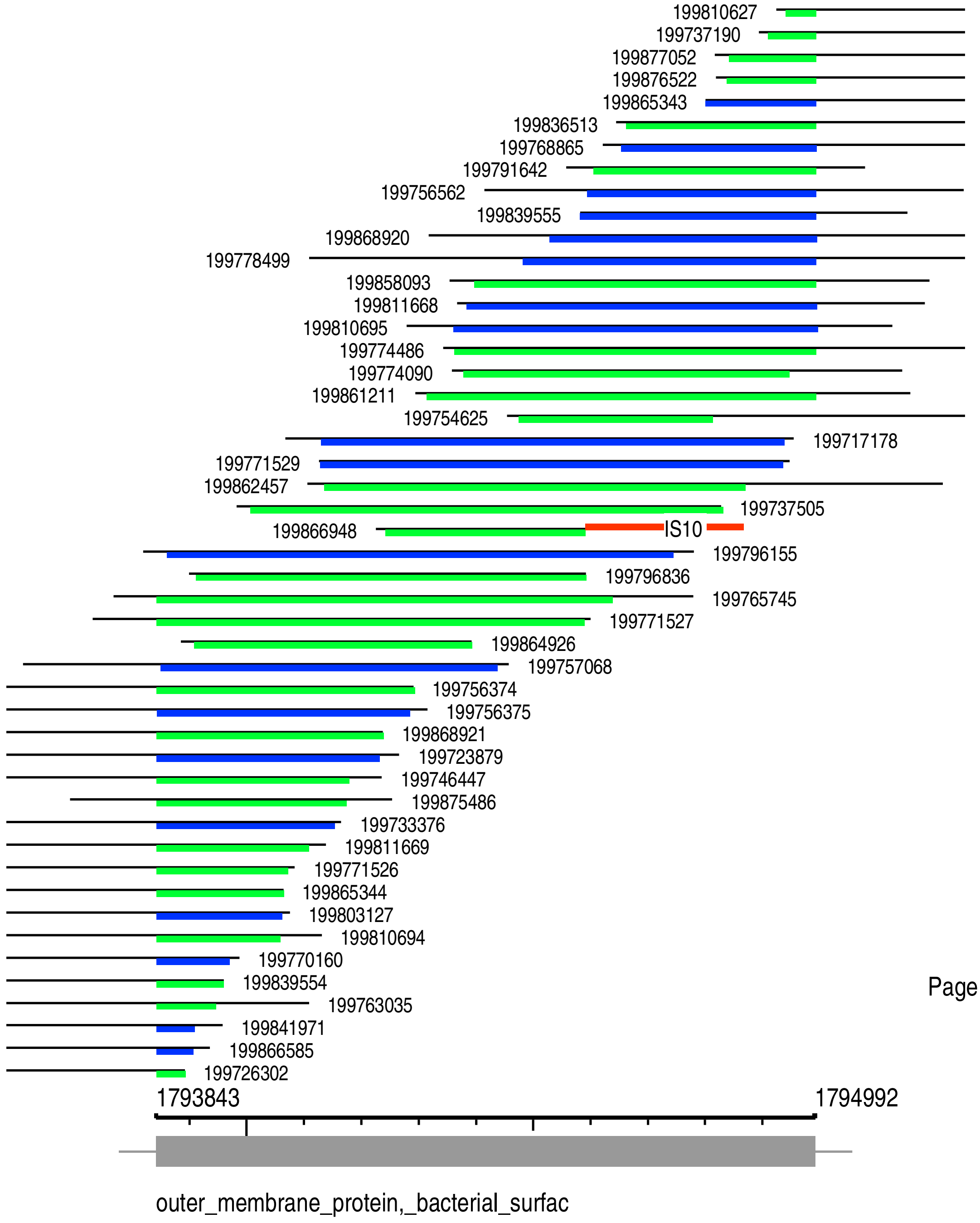

TX160488 Pseudomonas putida kt2440

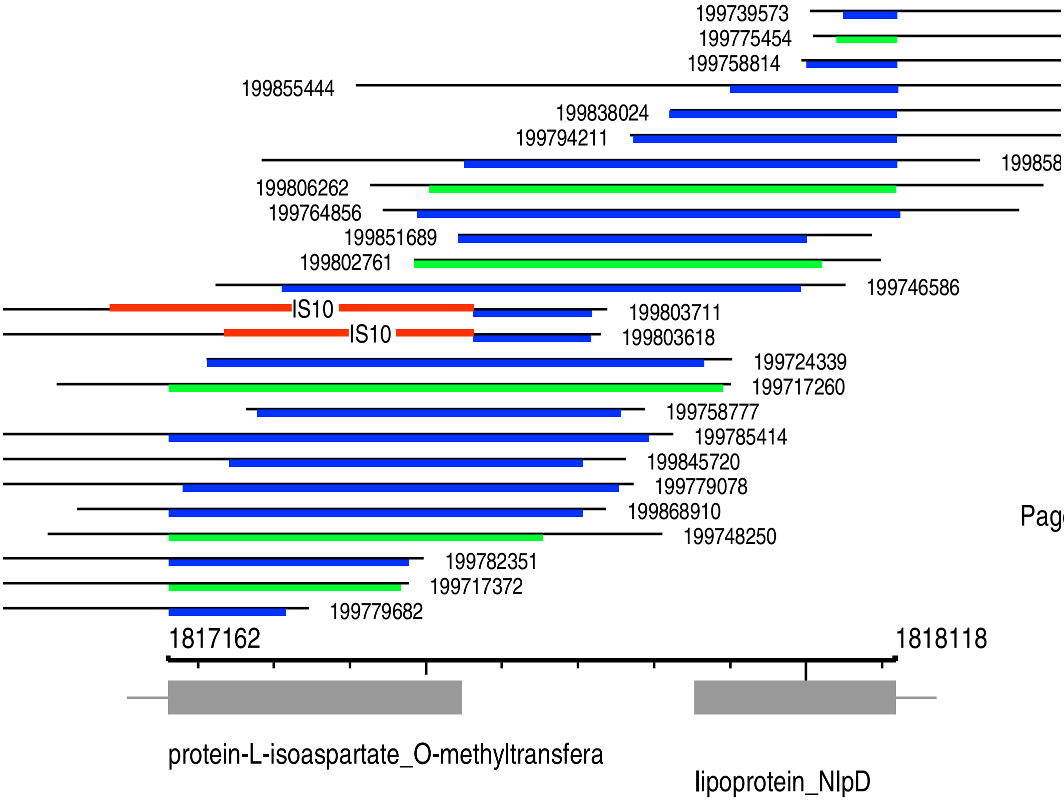

TX160488 Pseudomonas putida kt2440

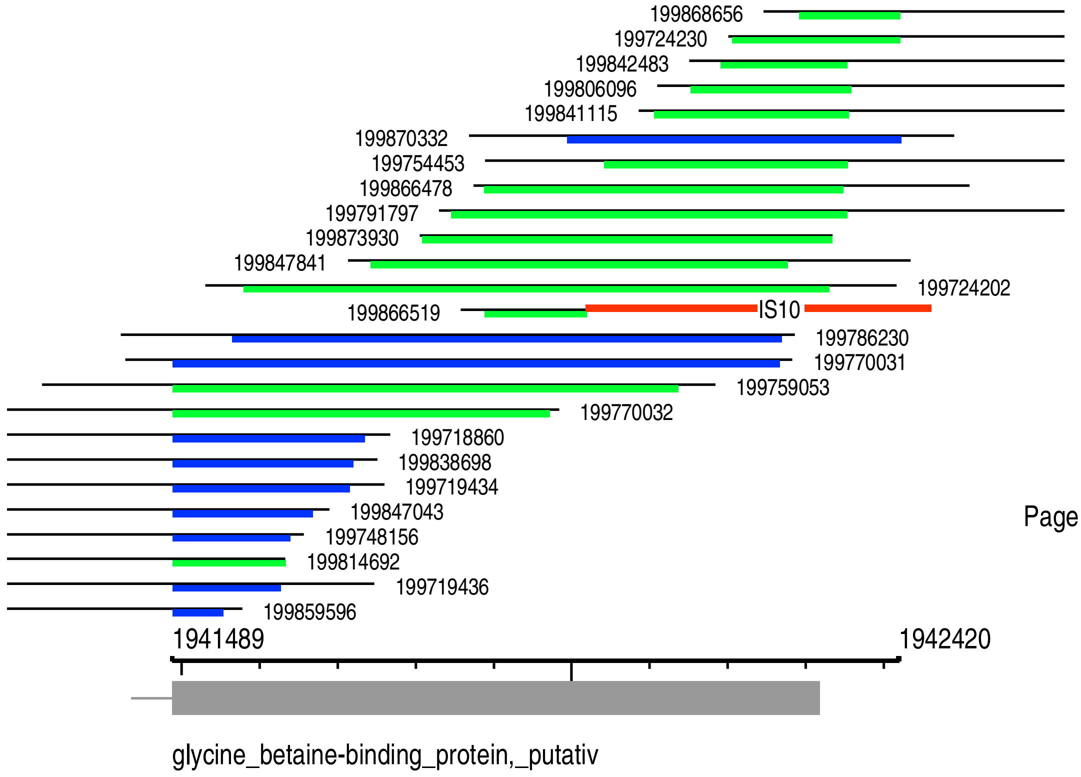

TX160488 Pseudomonas putida kt2440

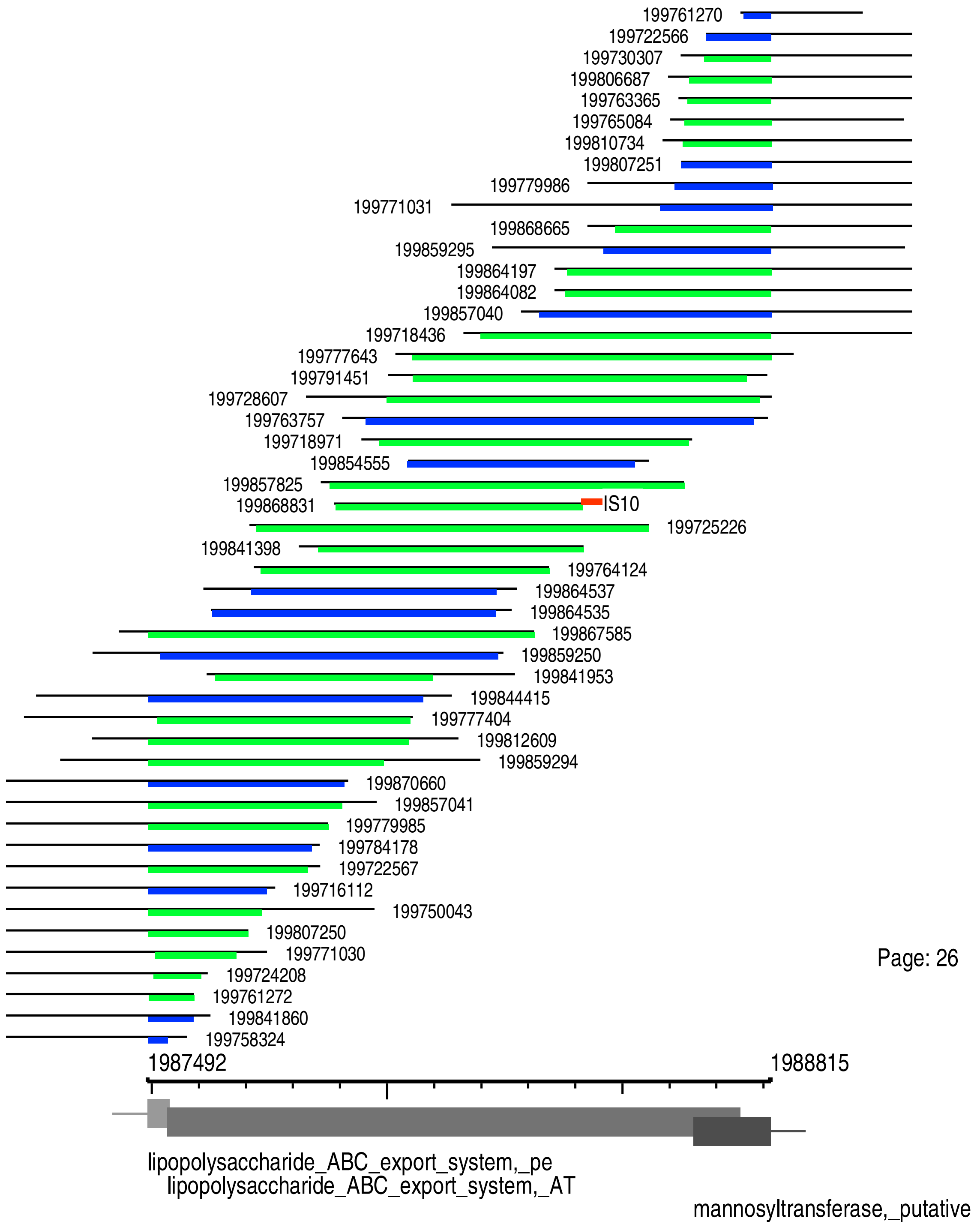

Page: 26

TX160488 Pseudomonas putida kt2440

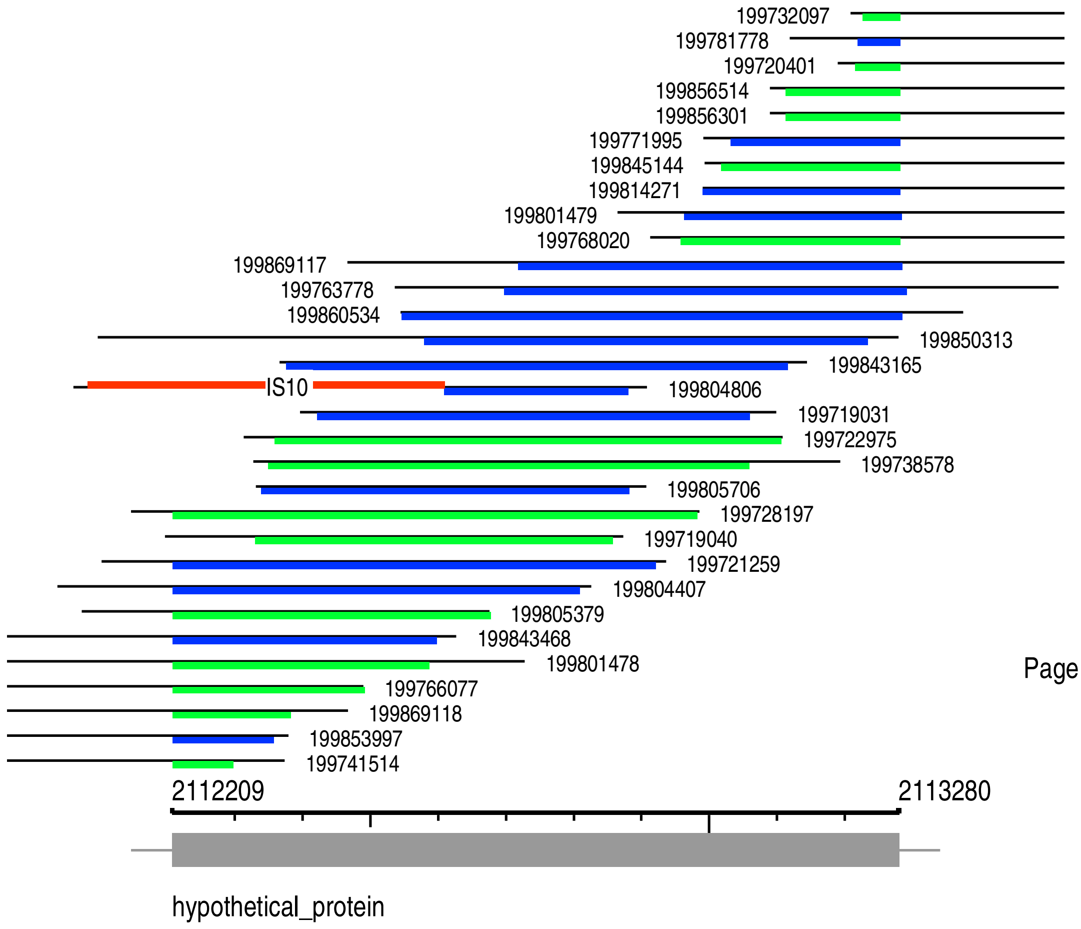

TX160488 Pseudomonas putida kt2440

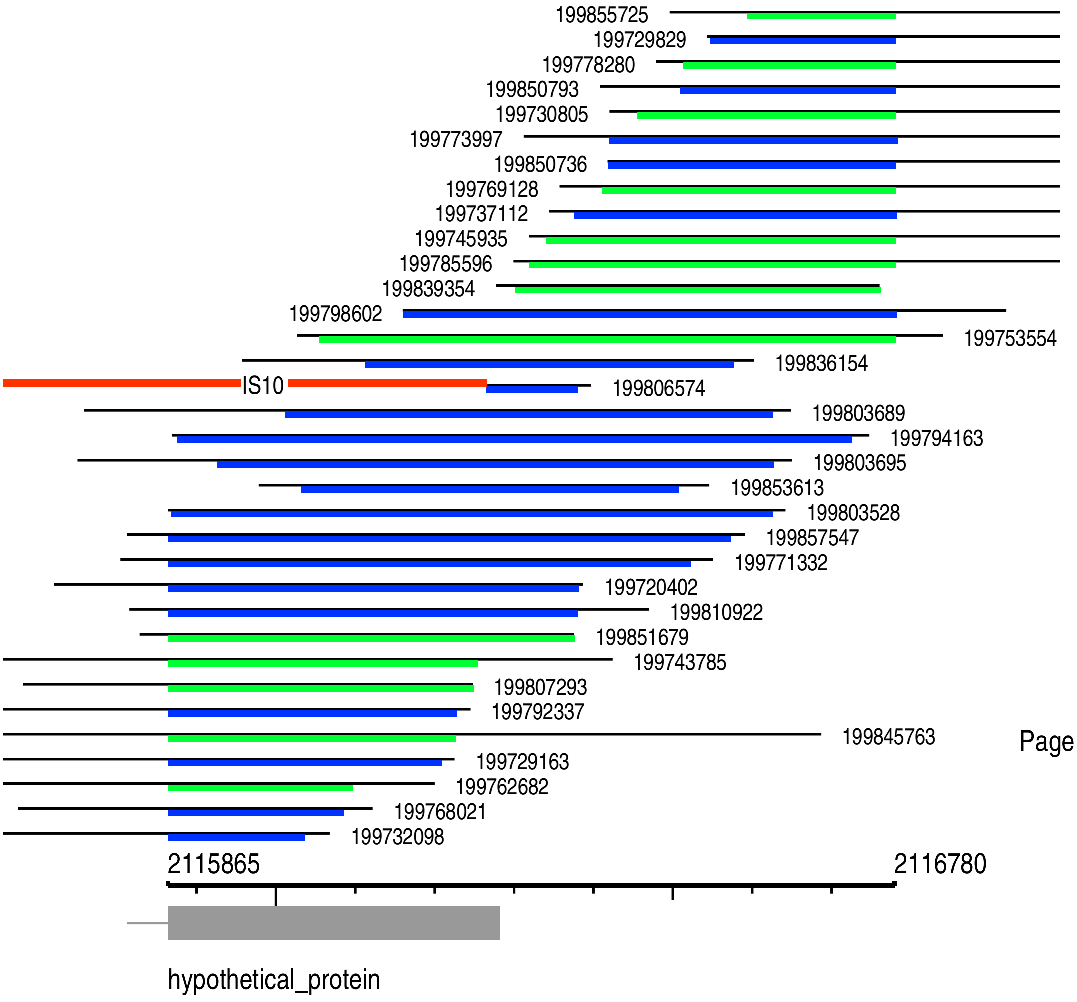

Page: 28

TX160488 Pseudomonas putida kt2440

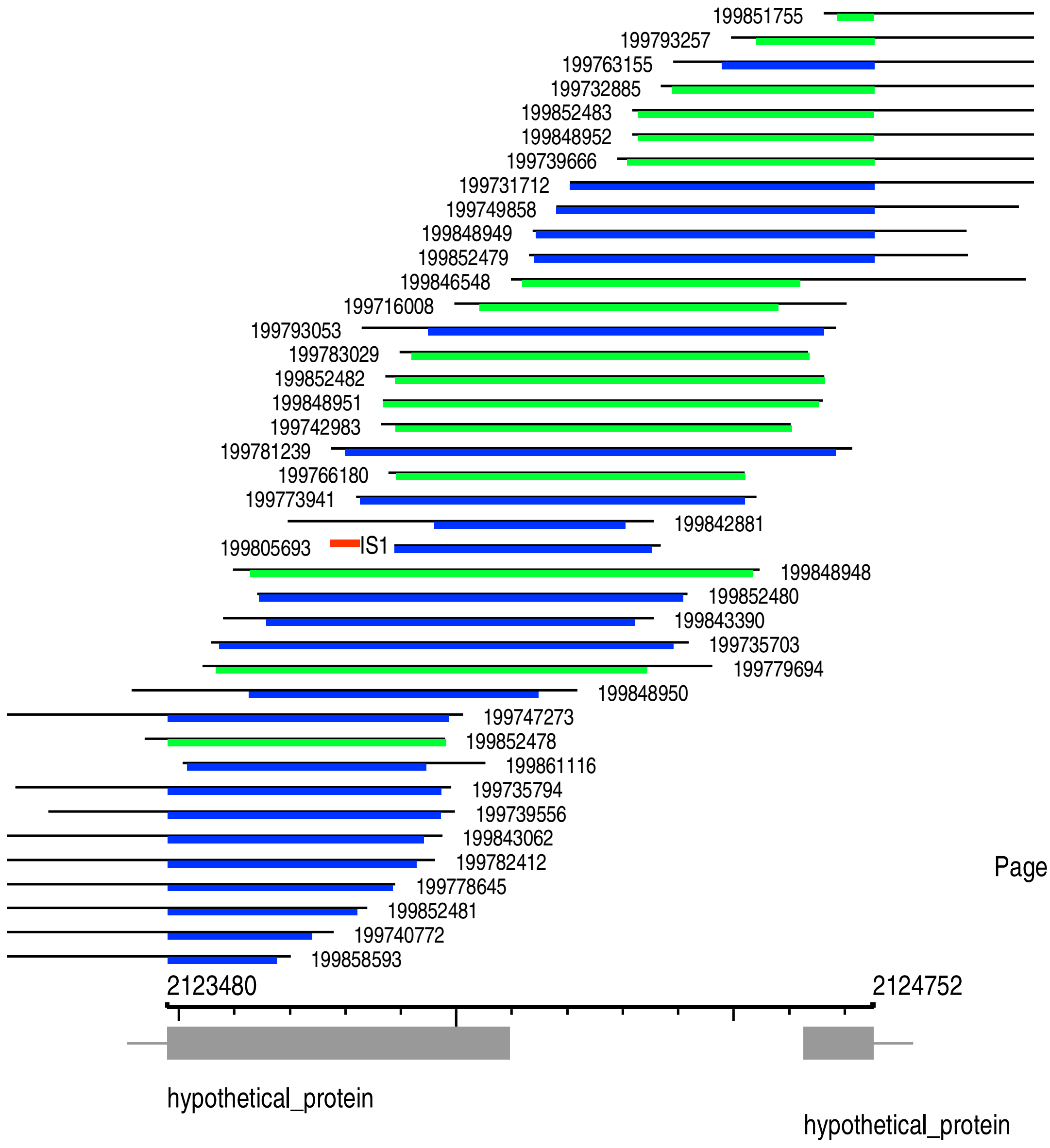

TX160488 Pseudomonas putida kt2440

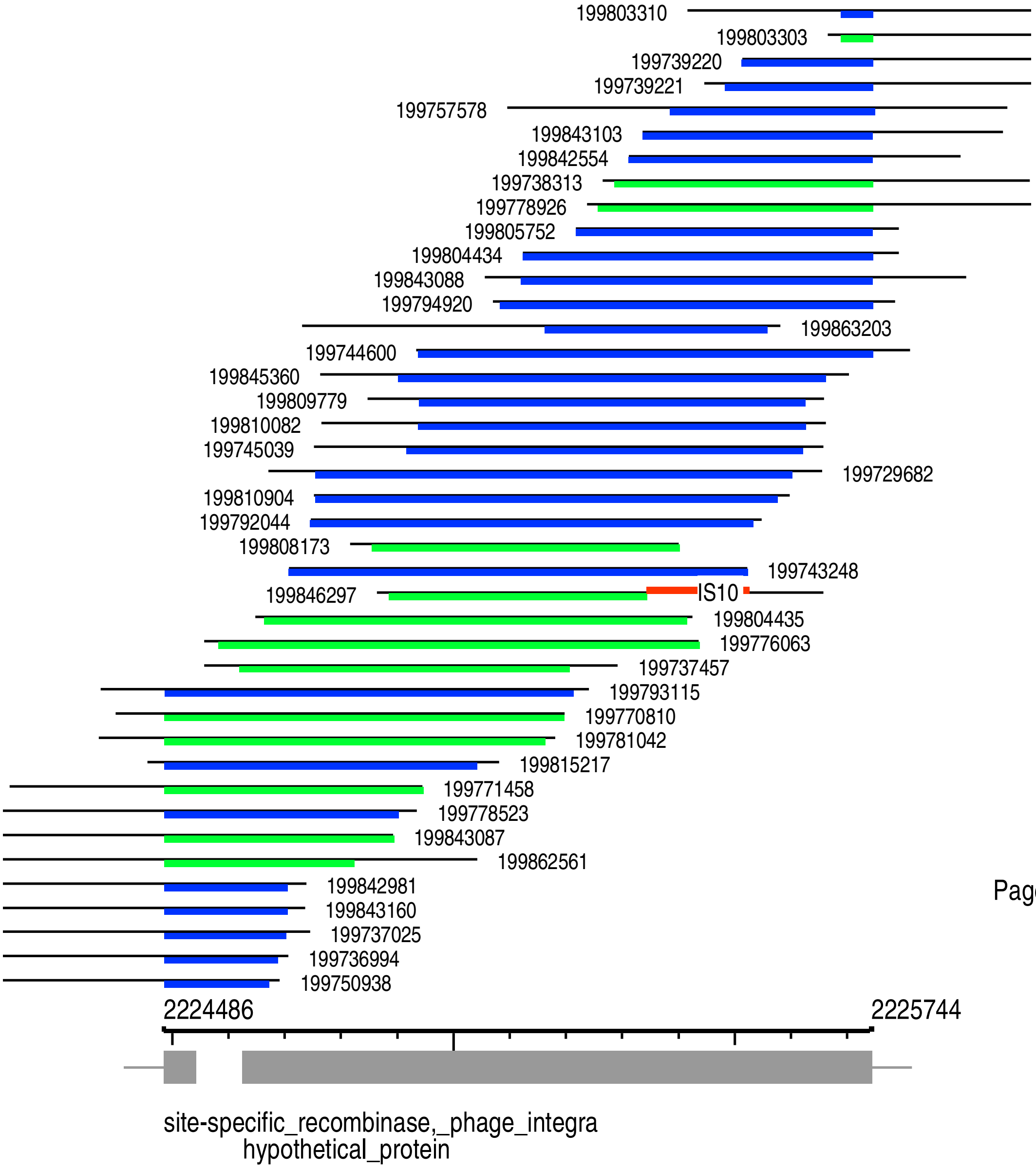

Page: 30

TX160488 Pseudomonas putida kt2440

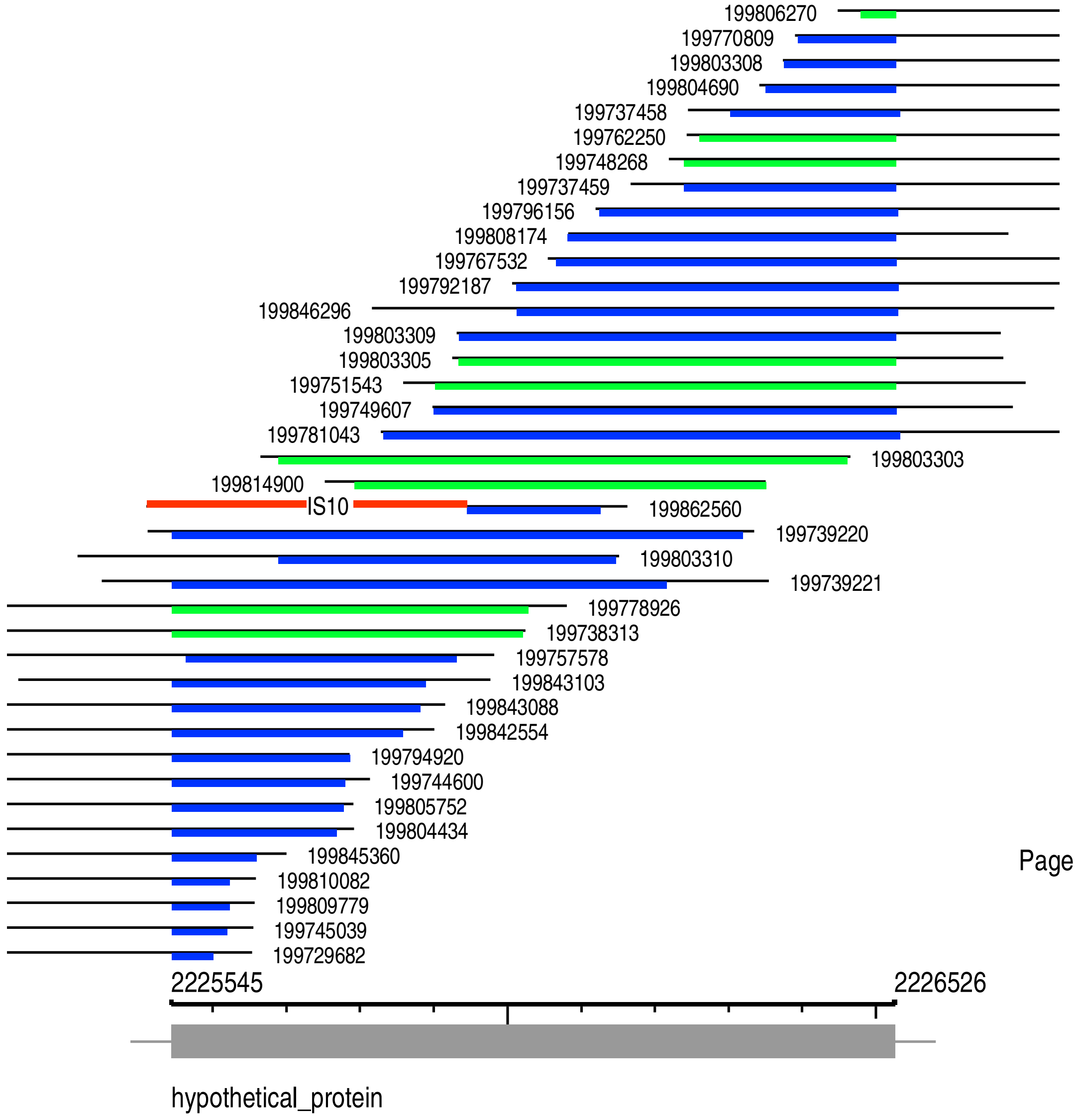

TX160488 Pseudomonas putida kt2440

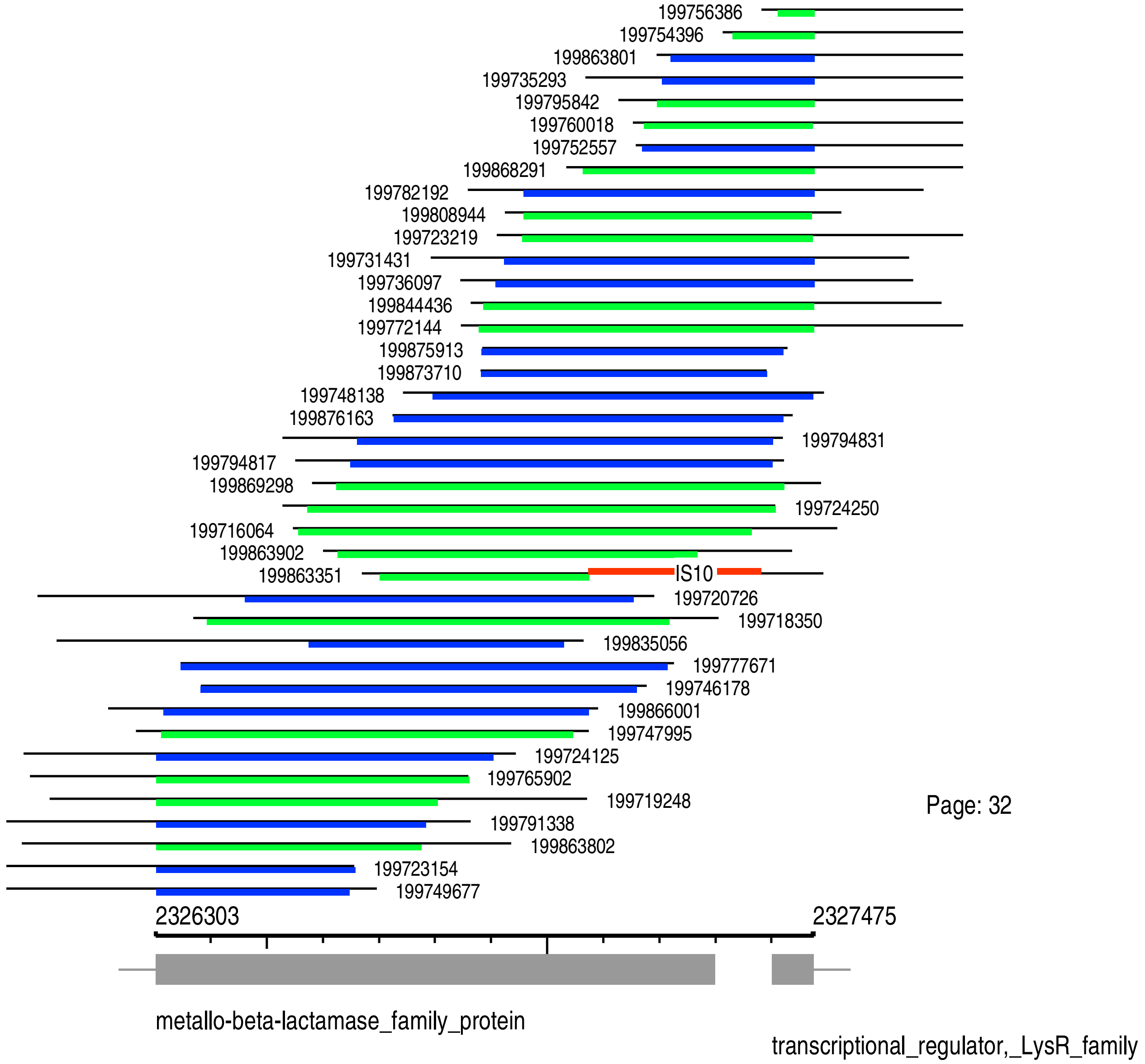

Page: 32

TX160488 Pseudomonas putida kt2440

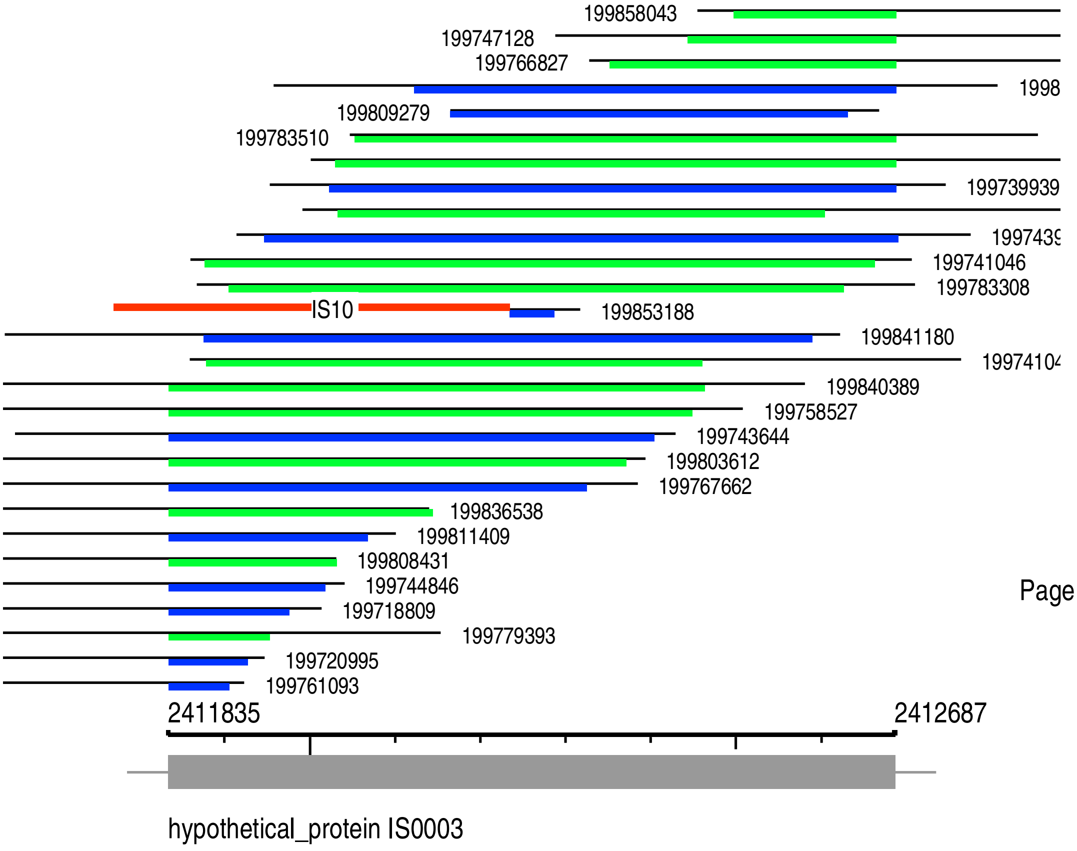

Page: 33

TX160488 Pseudomonas putida kt2440

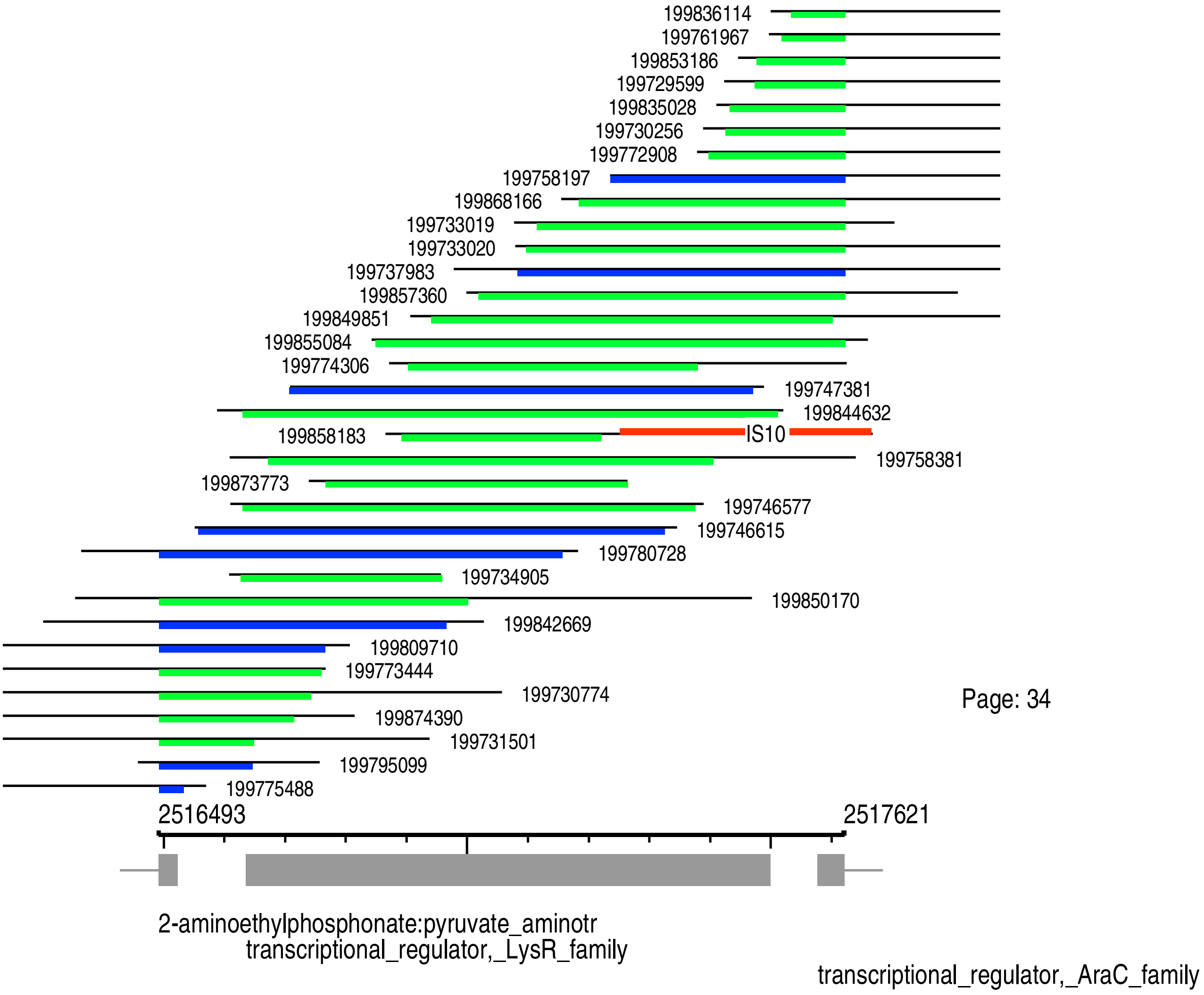

TX160488 Pseudomonas putida kt2440

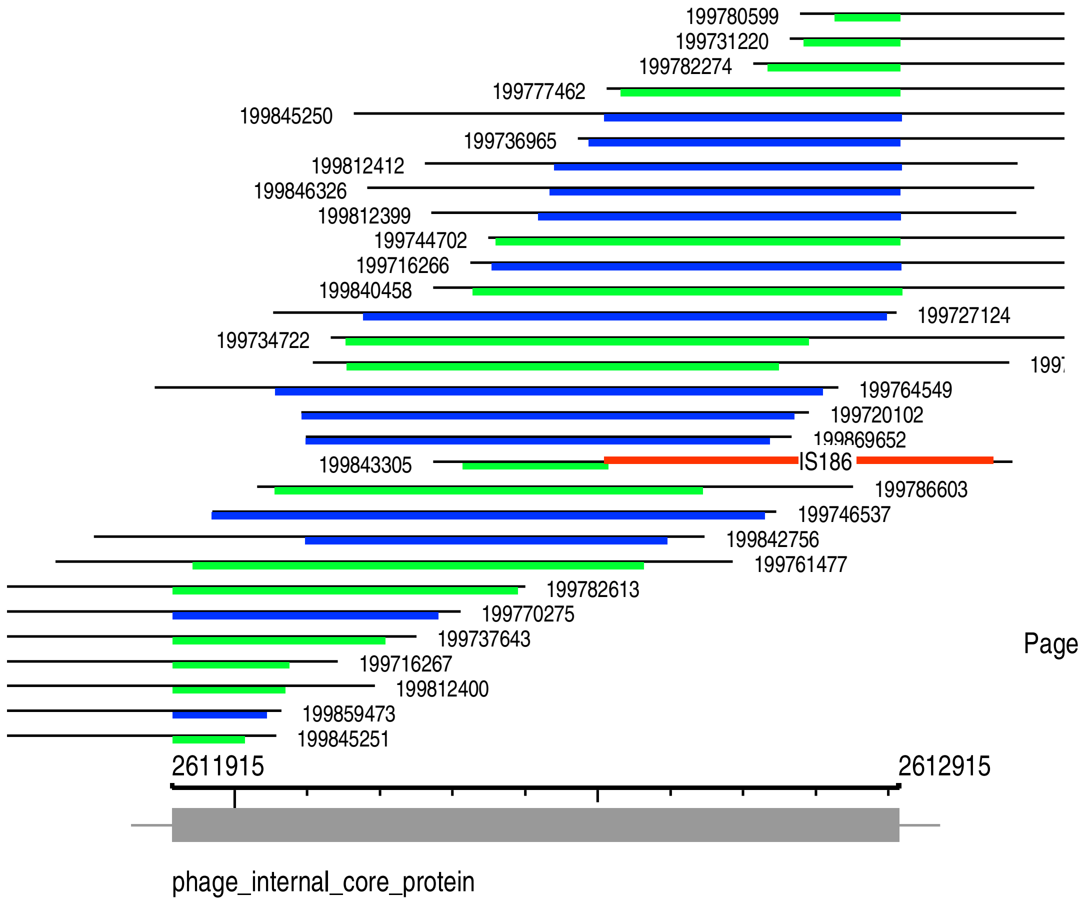

TX160488 Pseudomonas putida kt2440

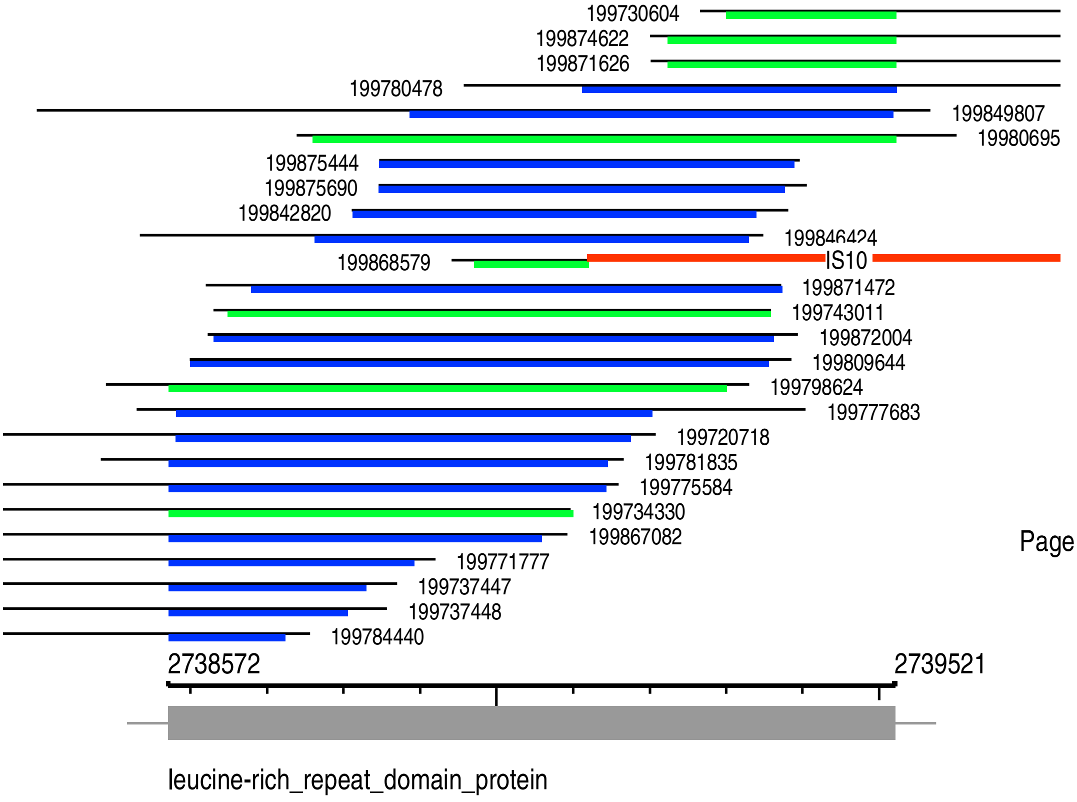

TX160488 Pseudomonas putida kt2440

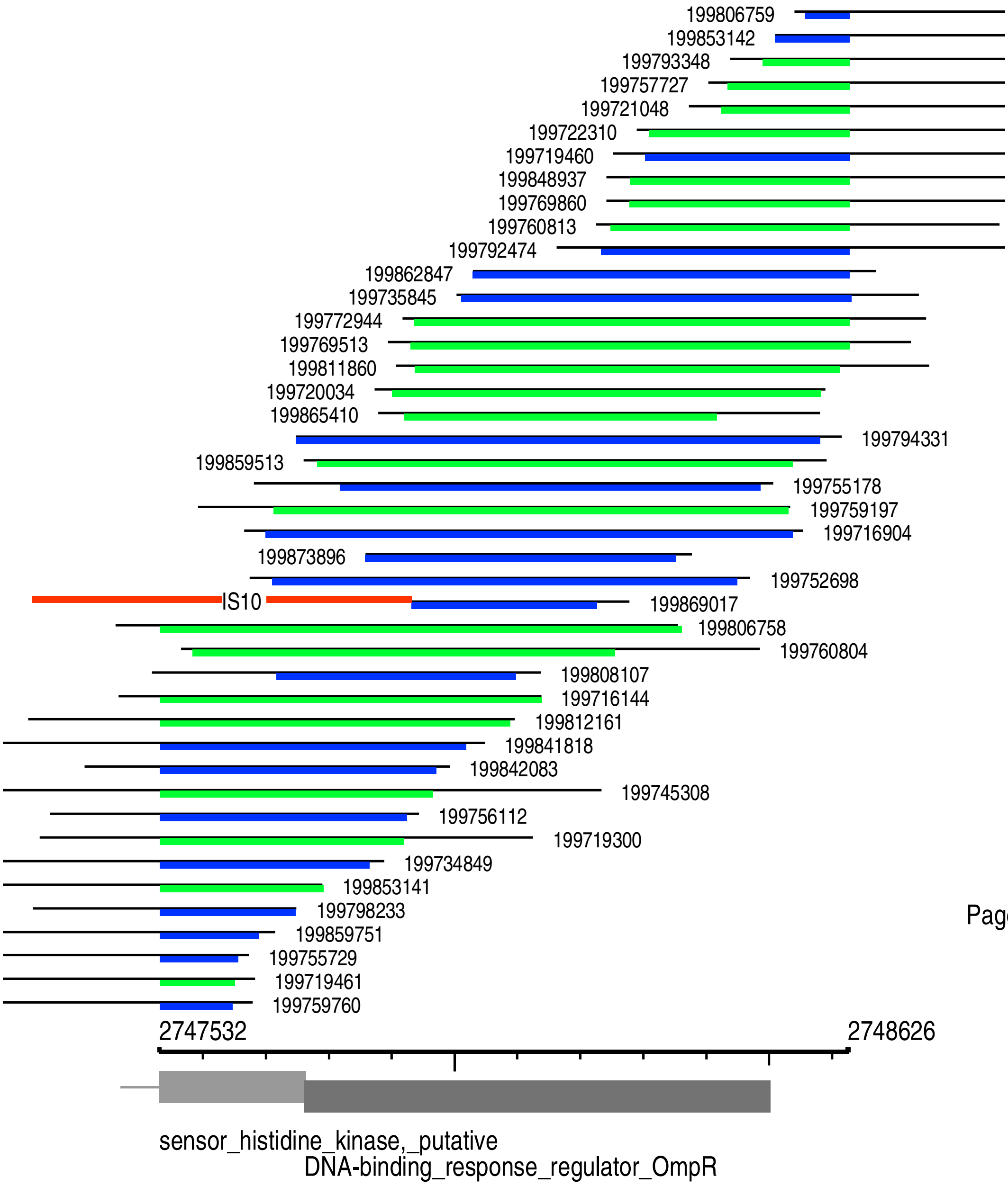

TX160488 Pseudomonas putida kt2440

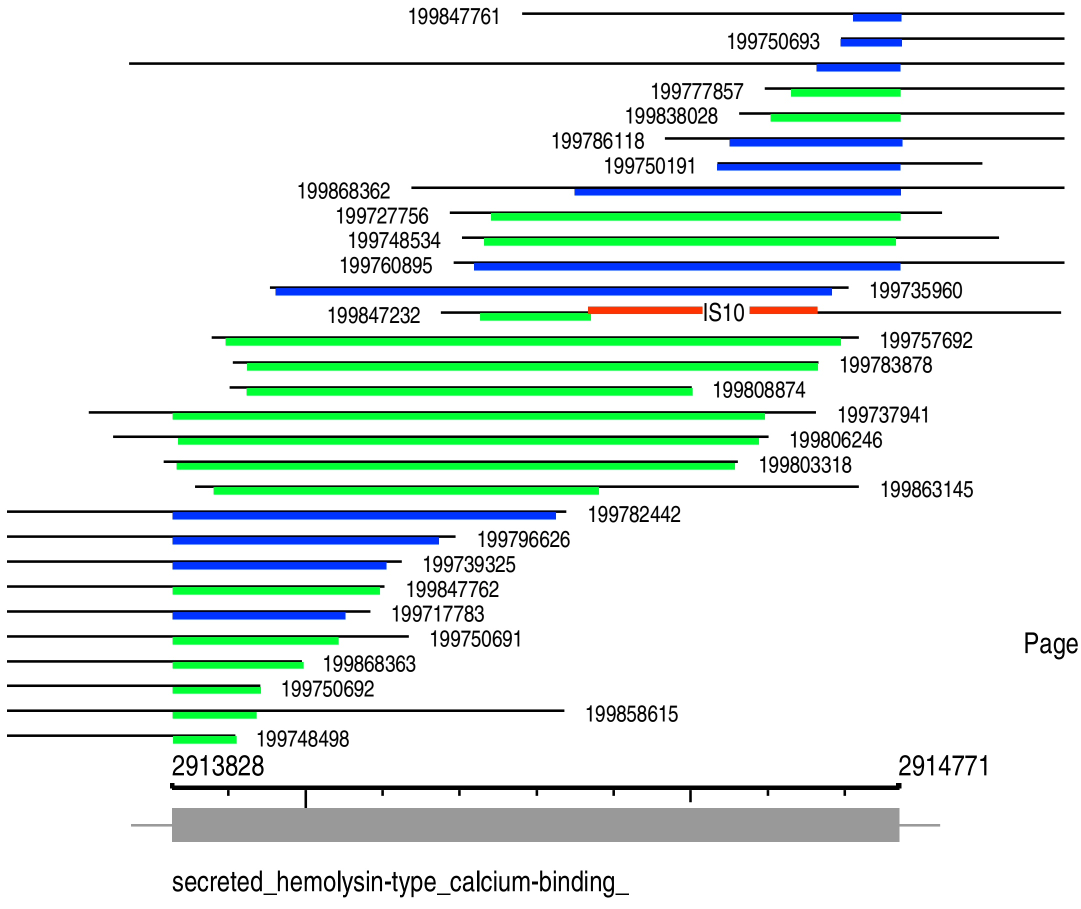

Page: 38

TX160488 Pseudomonas putida kt2440

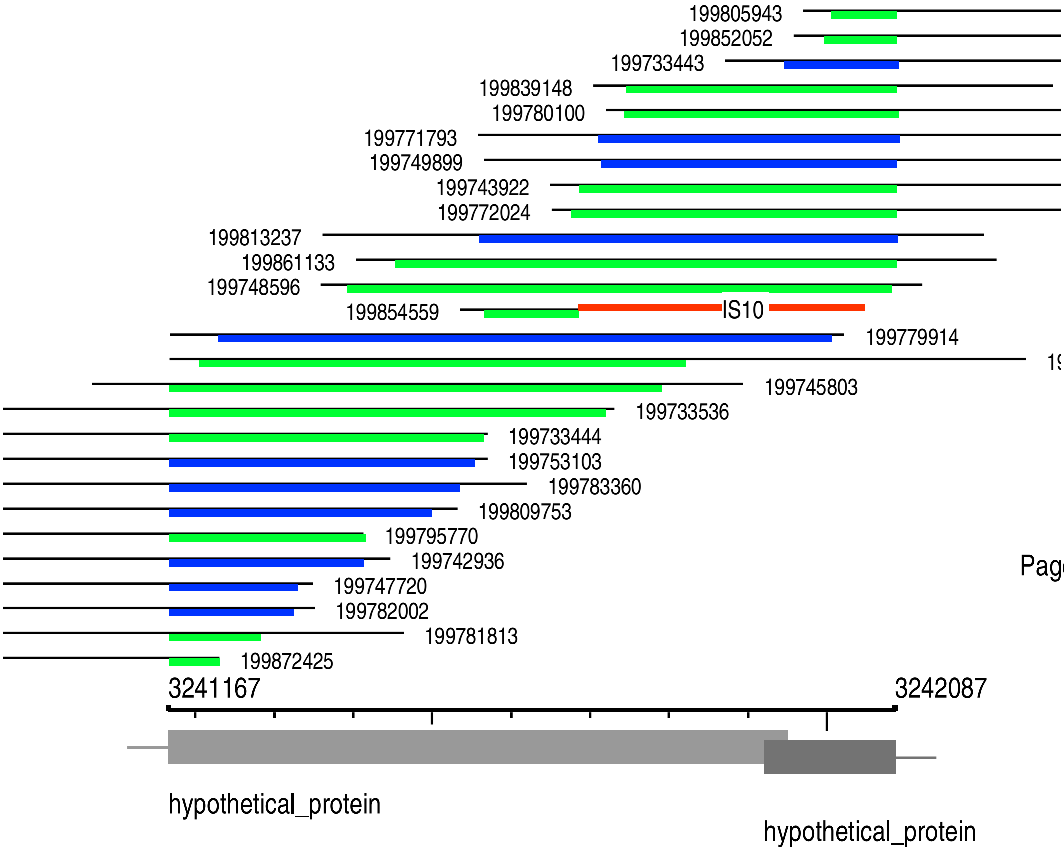

Page: 39

TX160488 Pseudomonas putida kt2440

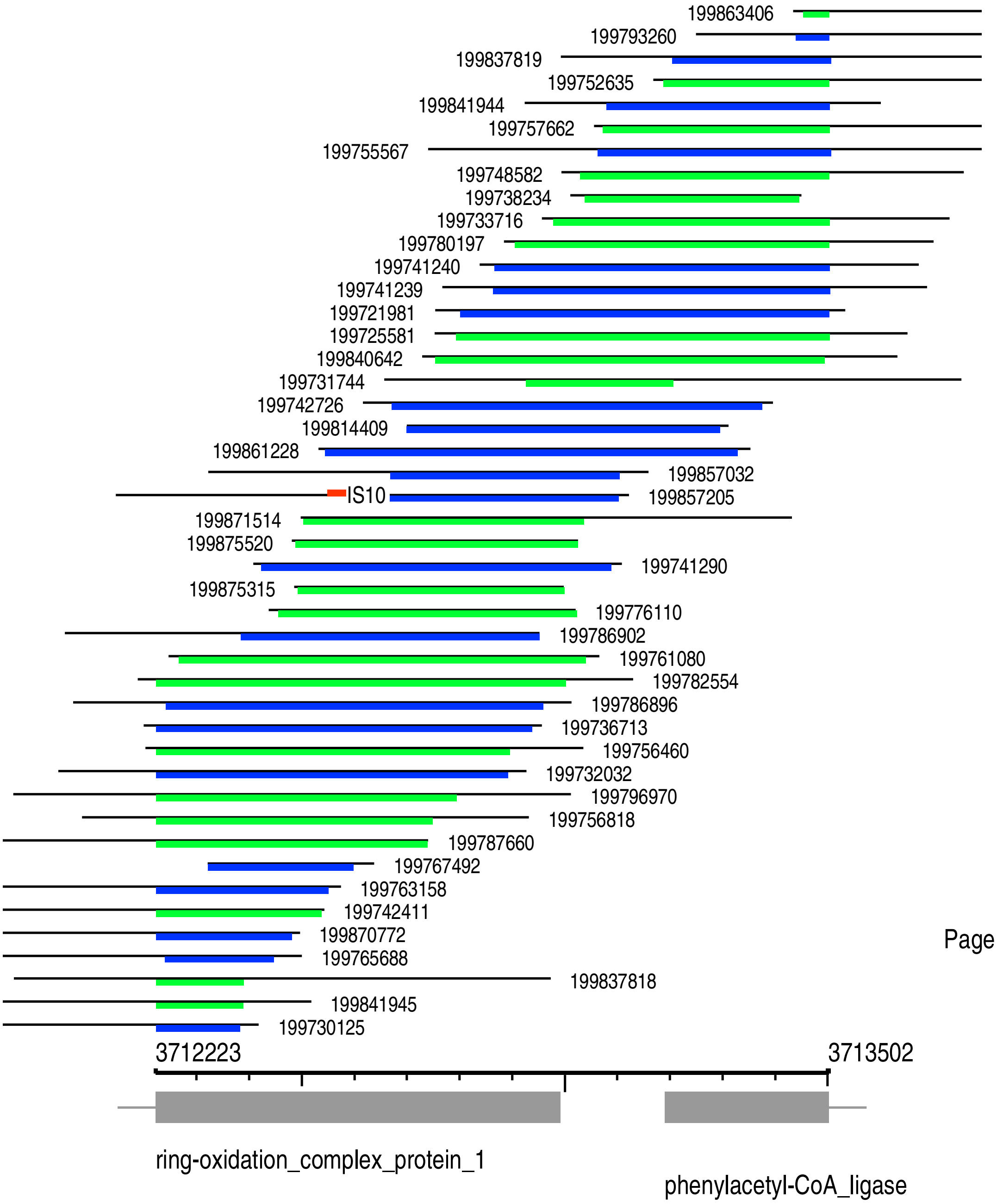

Page: 40

TX160488 Pseudomonas putida kt2440

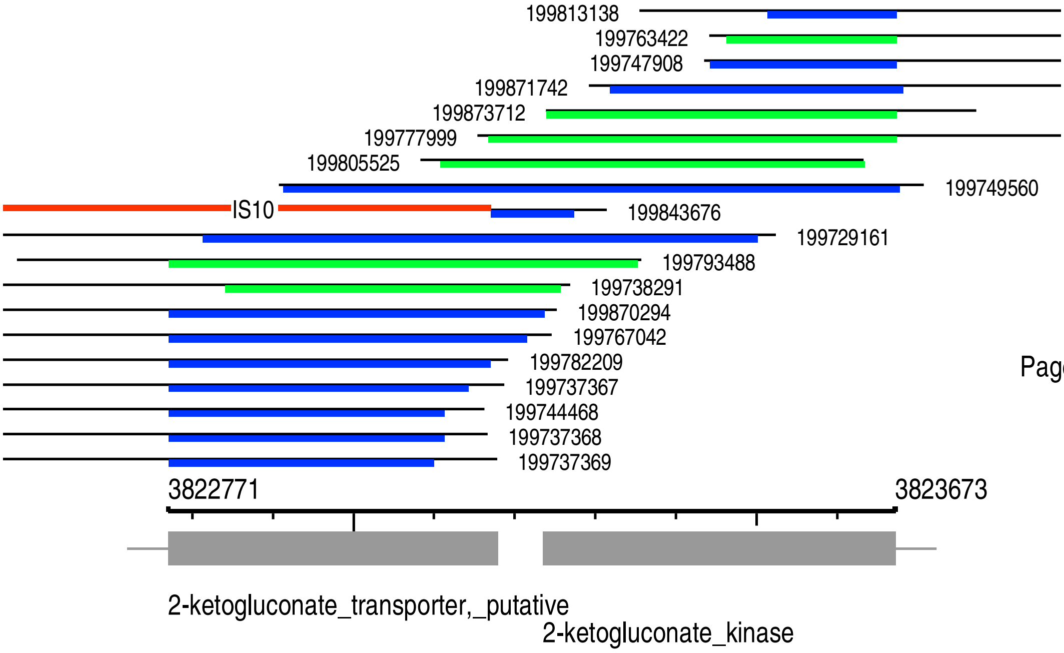

TX160488 Pseudomonas putida kt2440

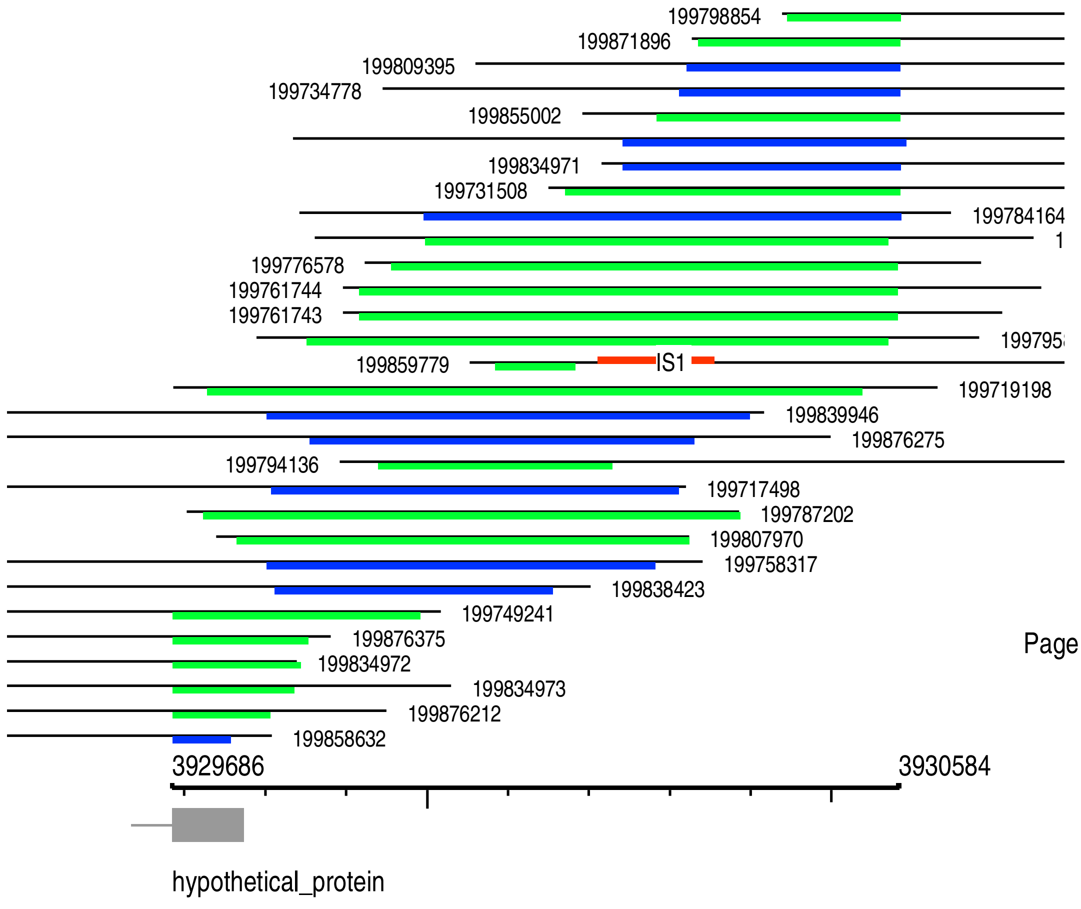

Page: 42

TX160488 Pseudomonas putida kt2440

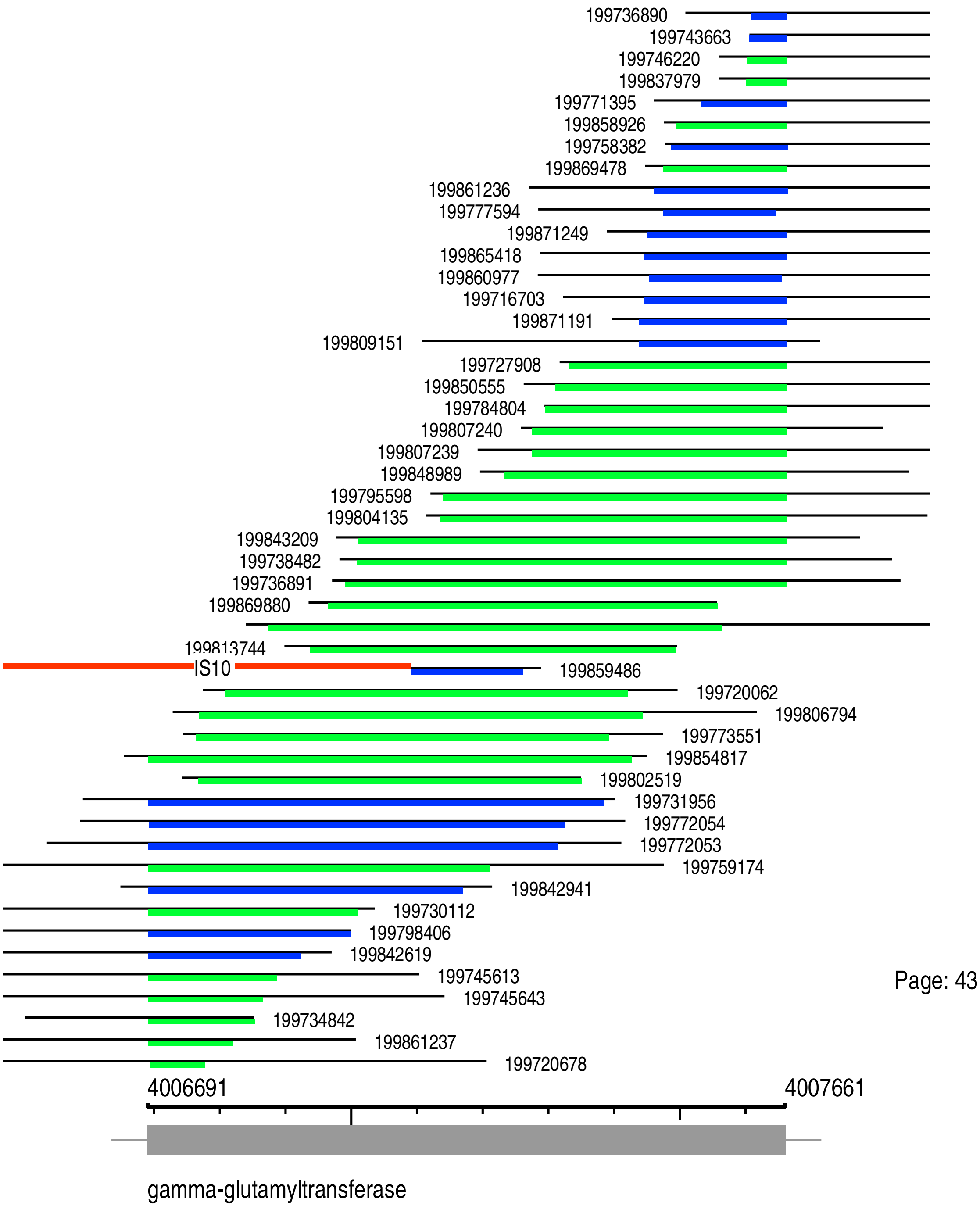

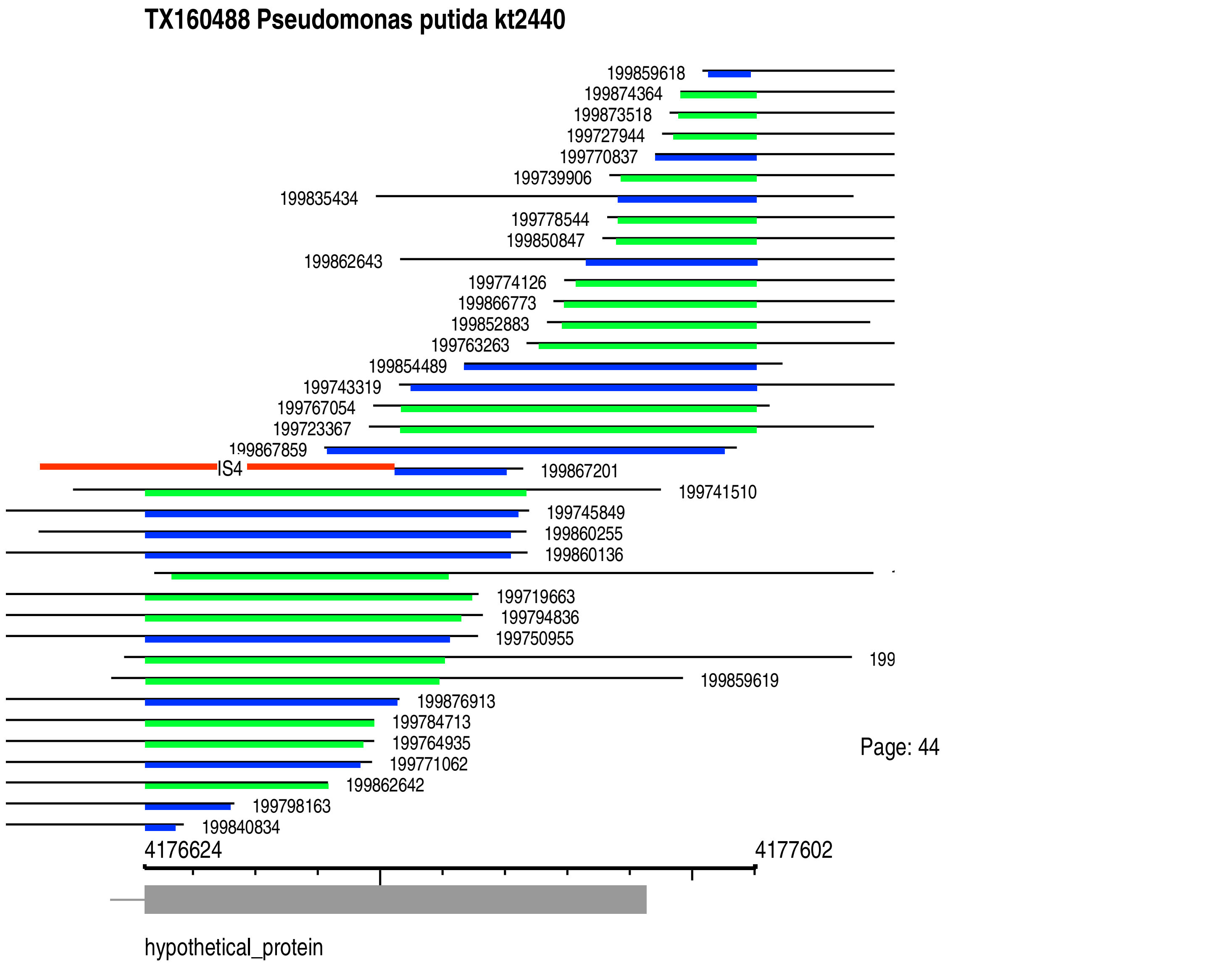
TX160488 Pseudomonas putida kt2440
199859618
199874364
199873518
199727944
199770837
199739906
199835434
199778544
199850847
199862643
199774126
199866773
199852883
199763263
199854489
199743319
199767054
199723367
199867859
IS4
199867201
199741510
199745849
199860255
199860136
199719663
199794836
199750955
199
199859619
199876913
199784713
199764935
199771062
199862642
199798163
199840834
4176624
4177602
hypothetical_protein

TX160488 Pseudomonas putida kt2440

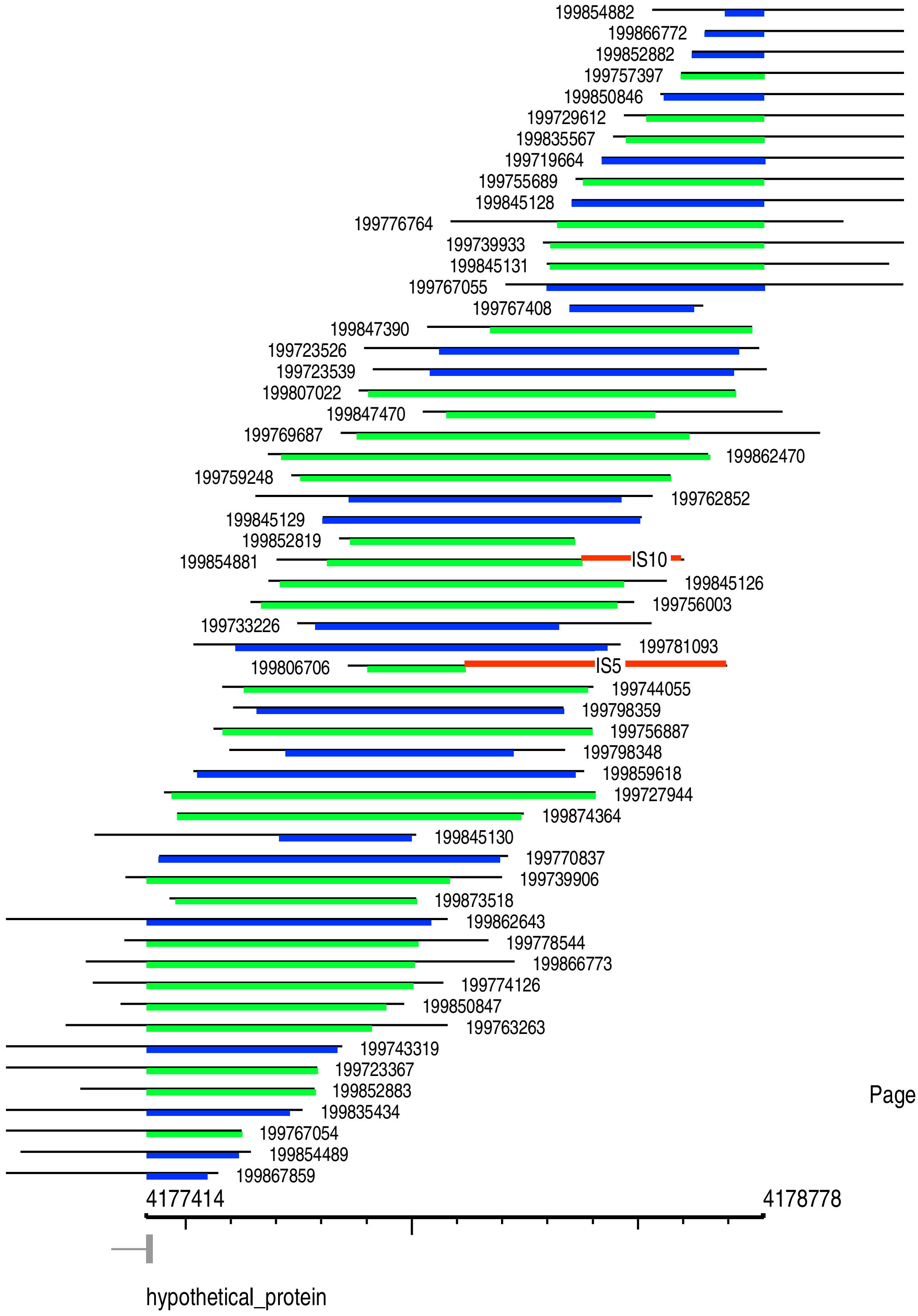

Page: 45

TX160488 Pseudomonas putida kt2440

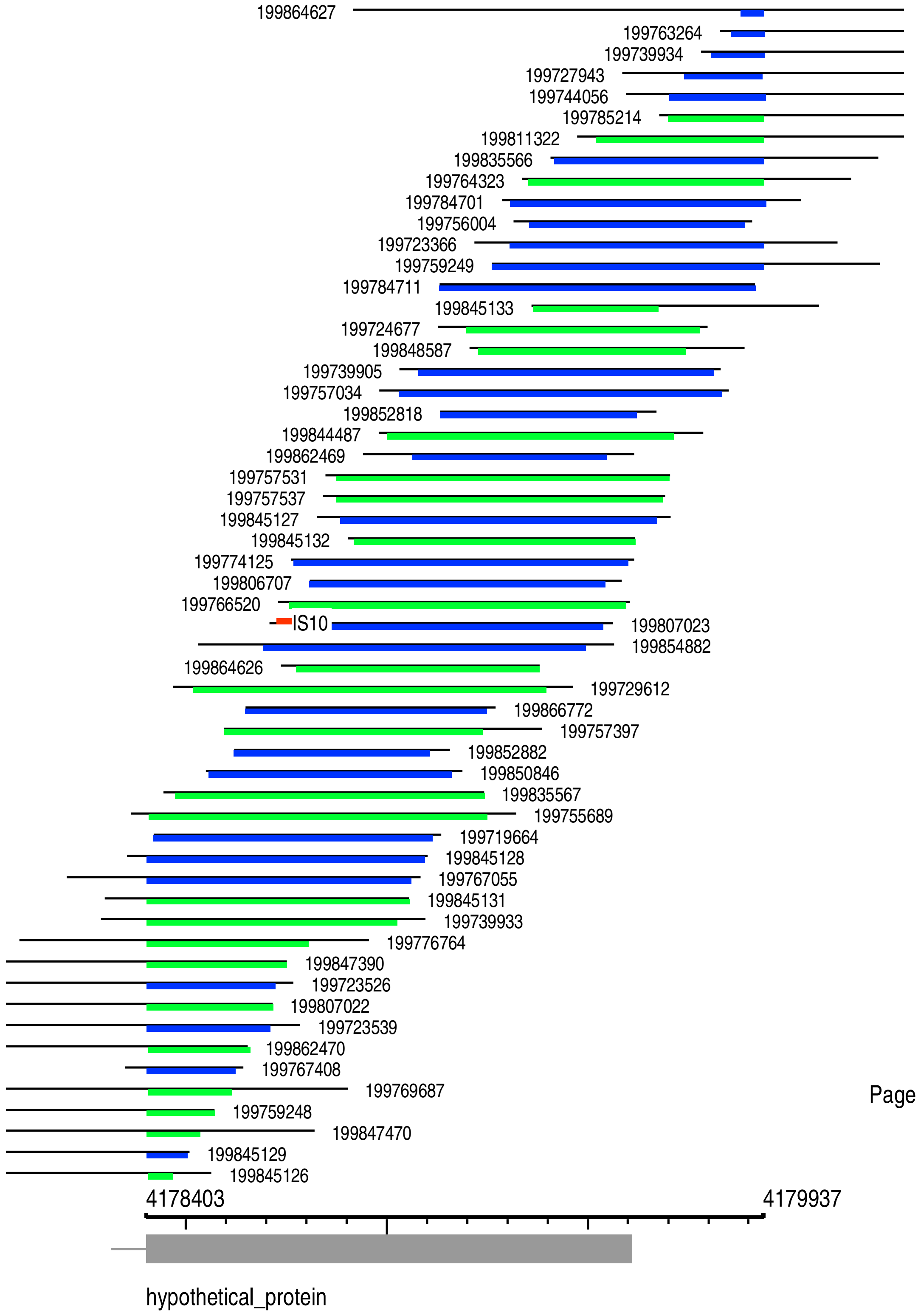

TX160488 Pseudomonas putida kt2440

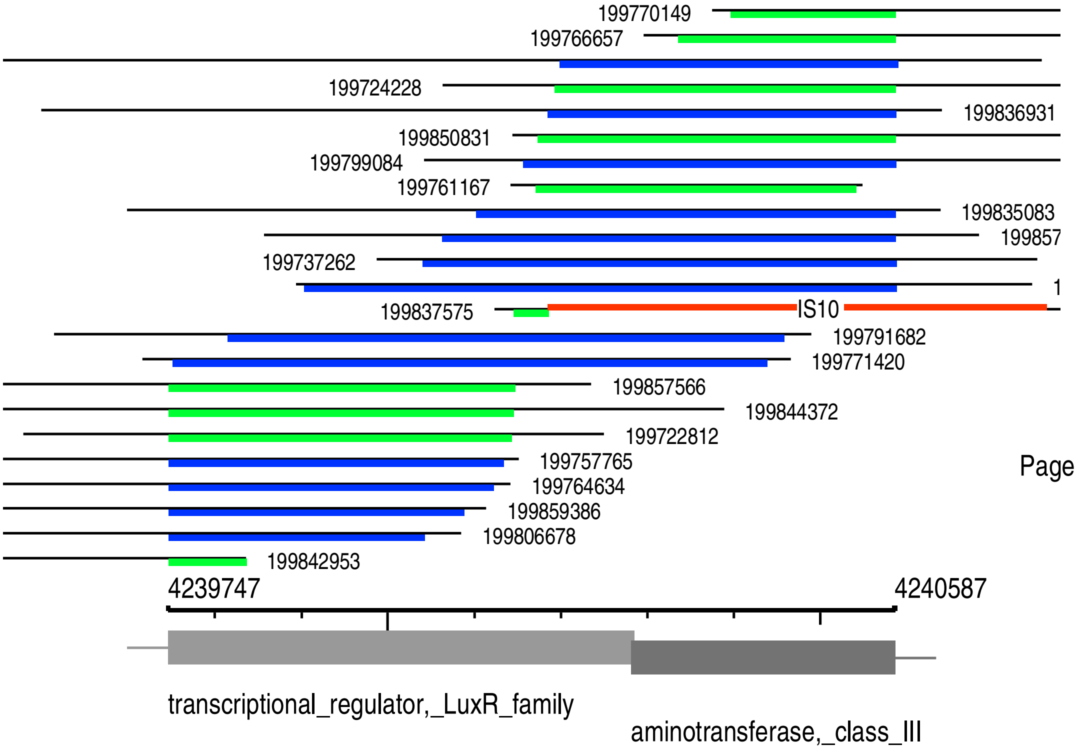

Page: 47

TX160488 Pseudomonas putida kt2440

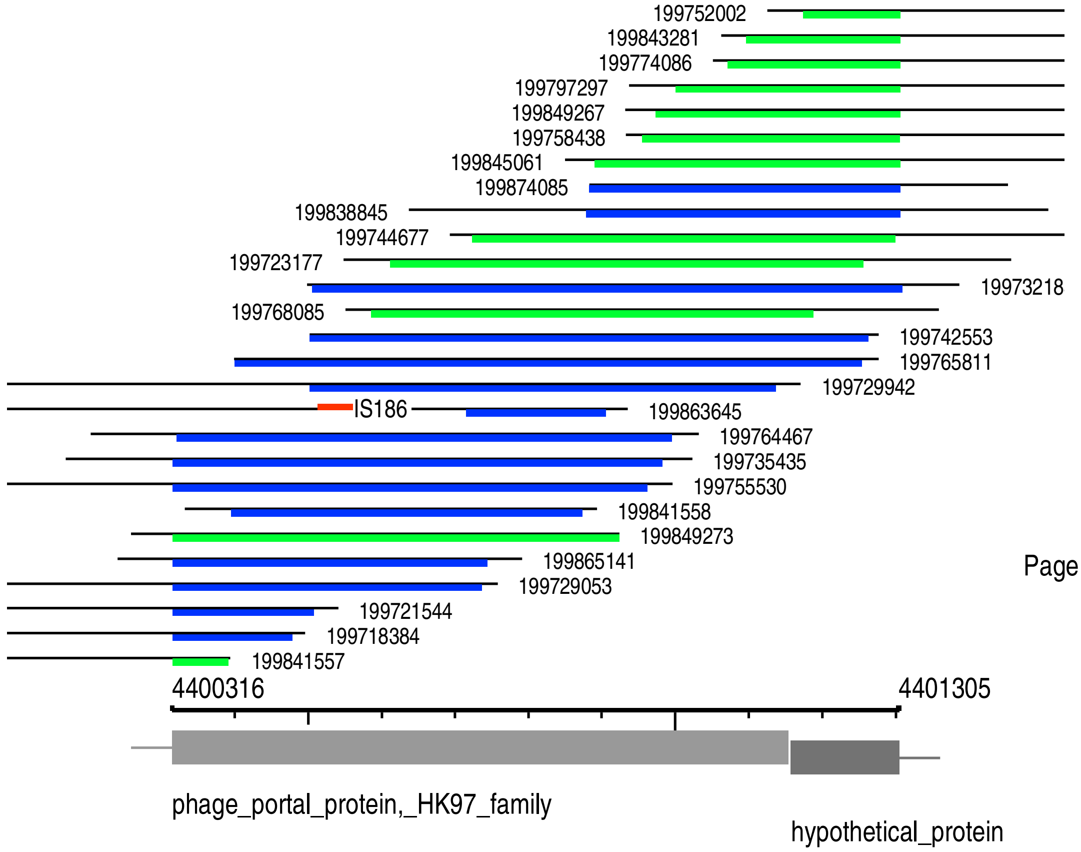

TX160488 Pseudomonas putida kt2440

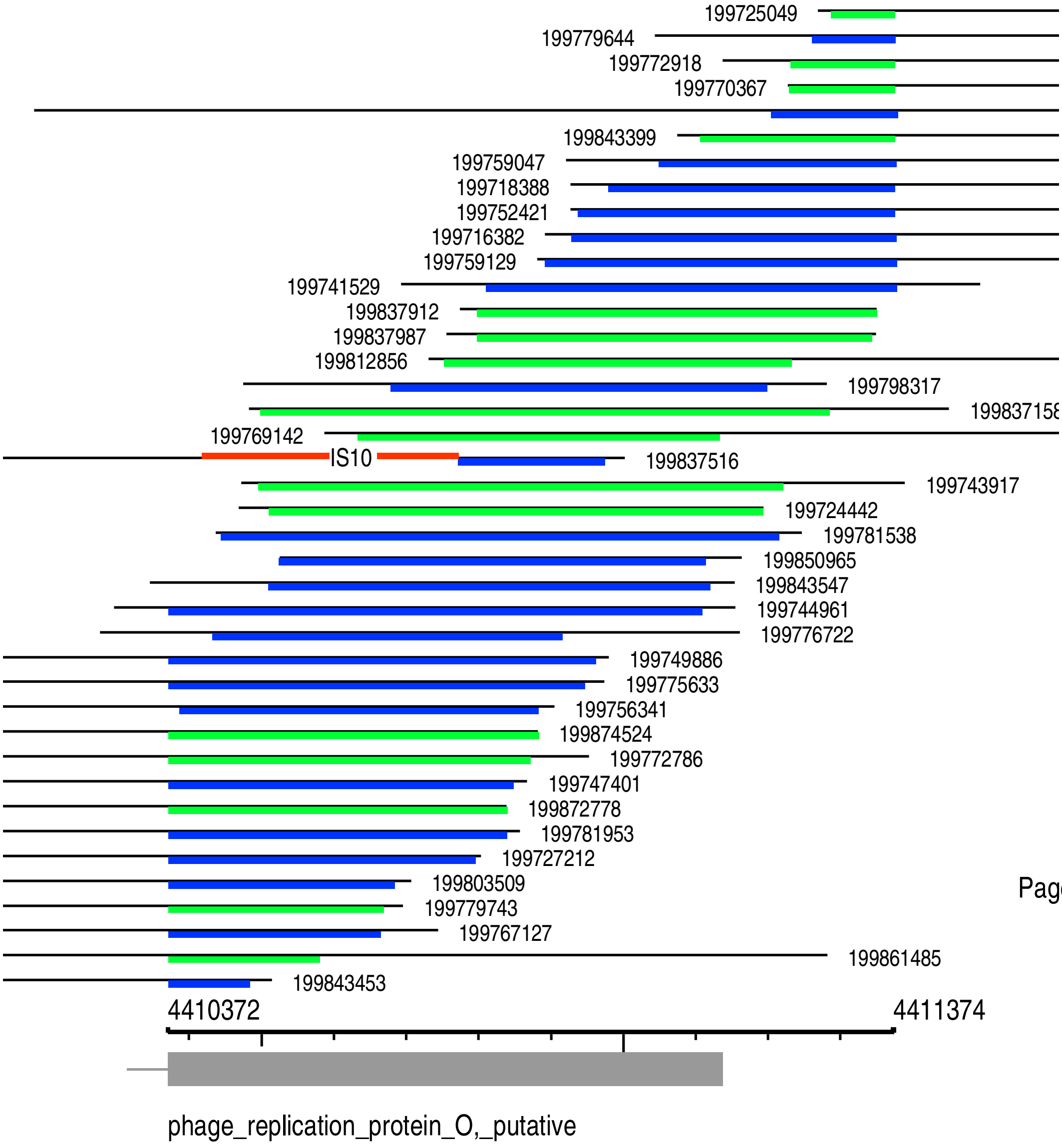

TX160488 Pseudomonas putida kt2440

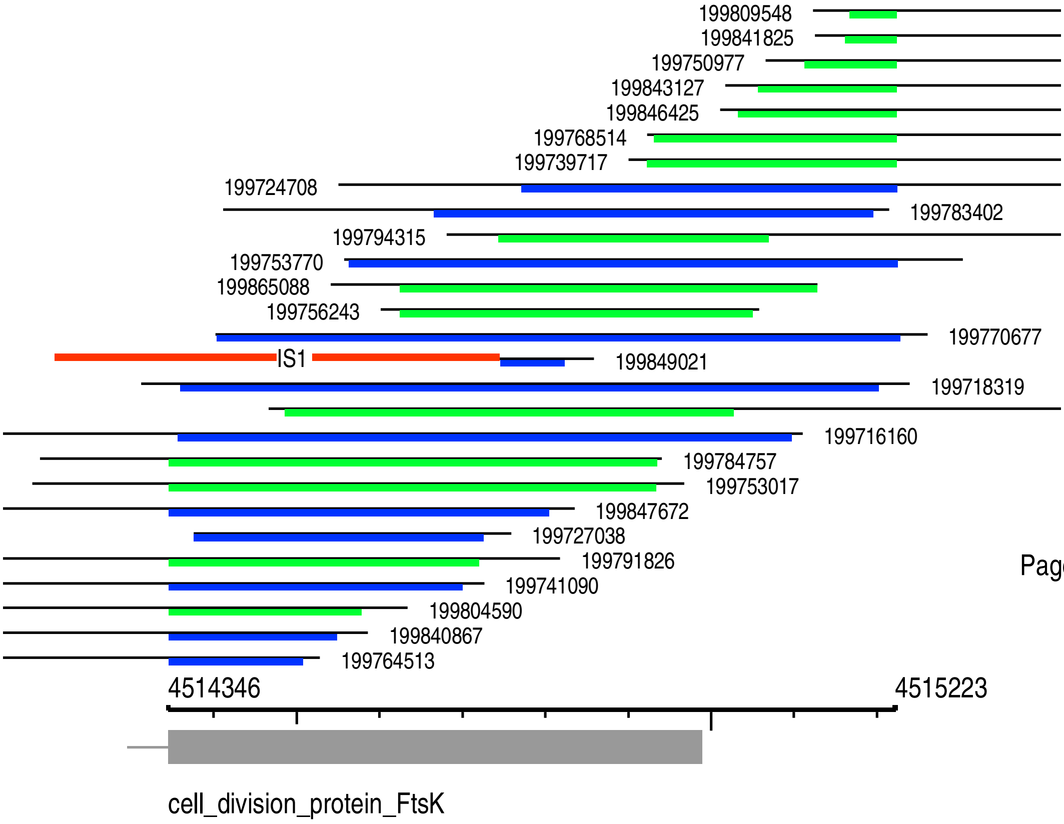

TX160488 Pseudomonas putida kt2440

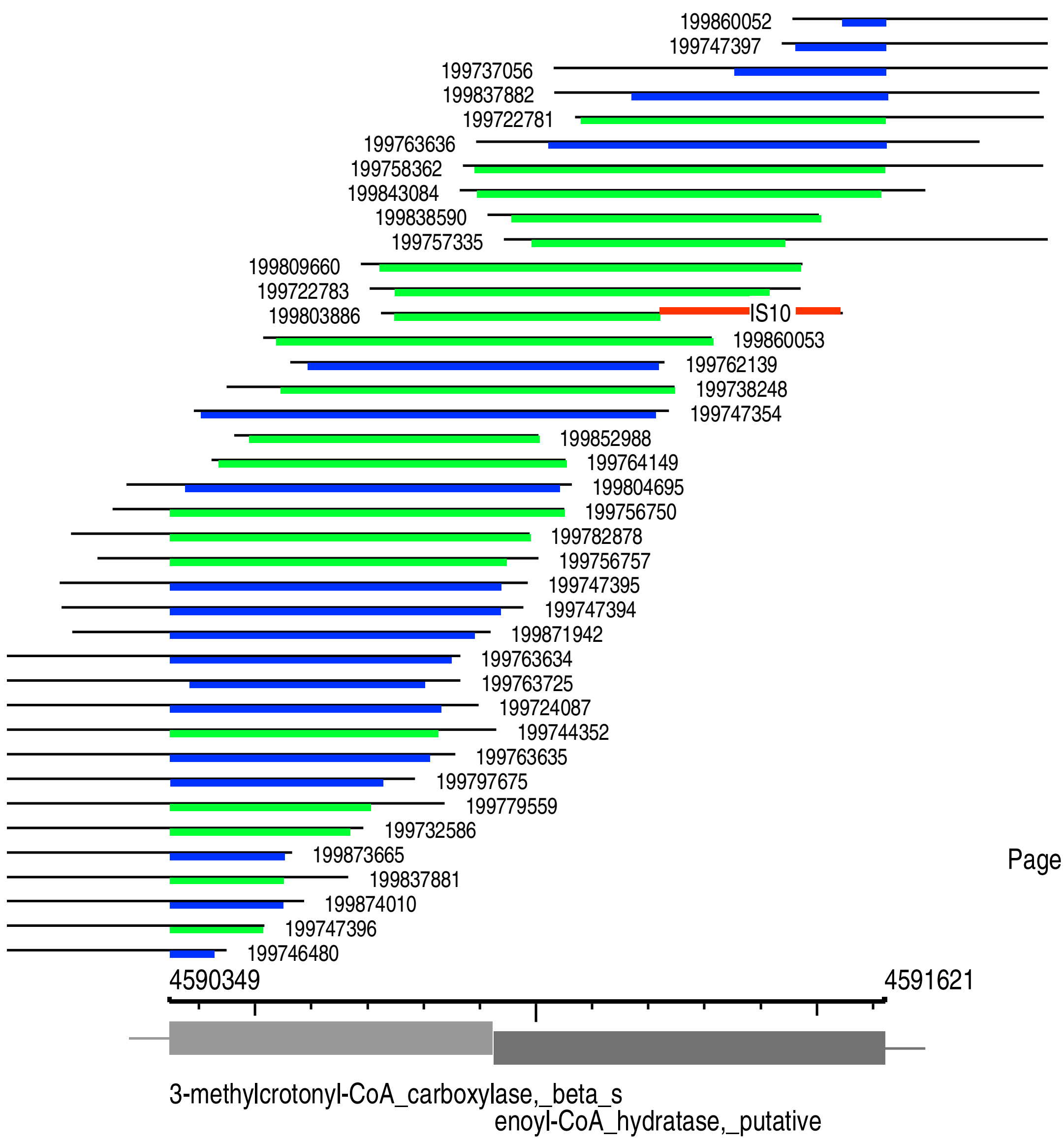

TX160488 Pseudomonas putida kt2440

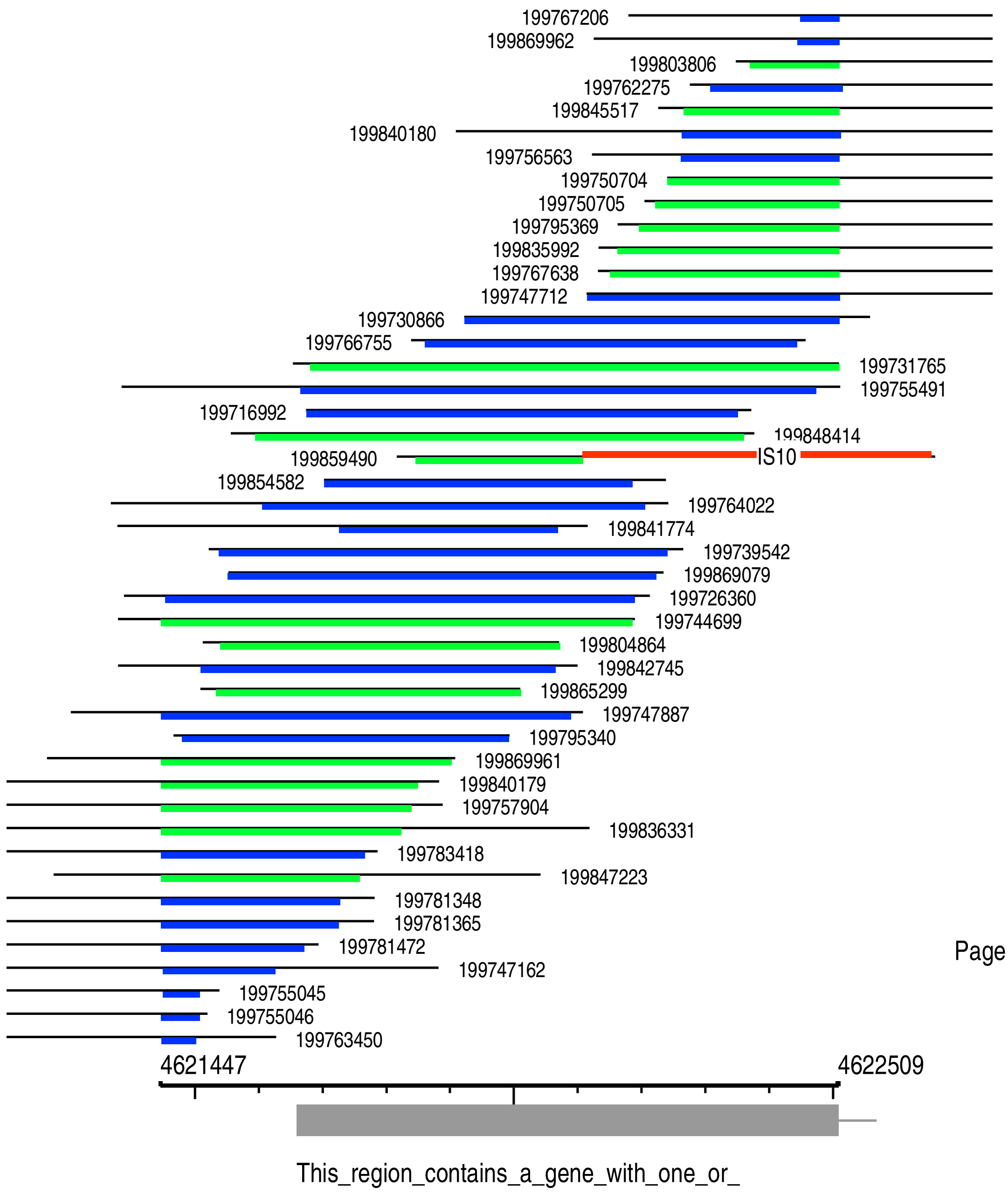

TX160488 Pseudomonas putida kt2440

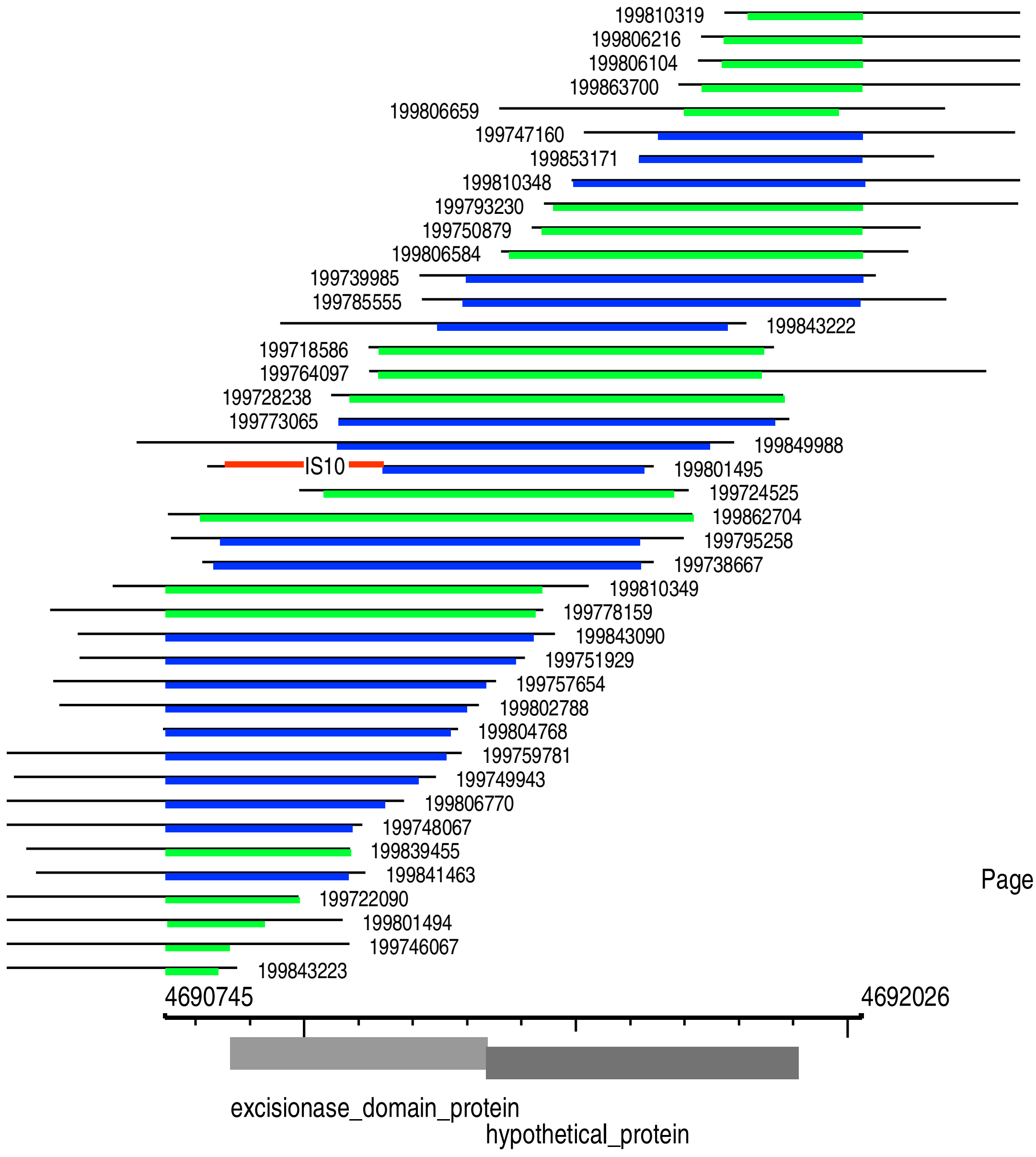

TX160488 Pseudomonas putida kt2440

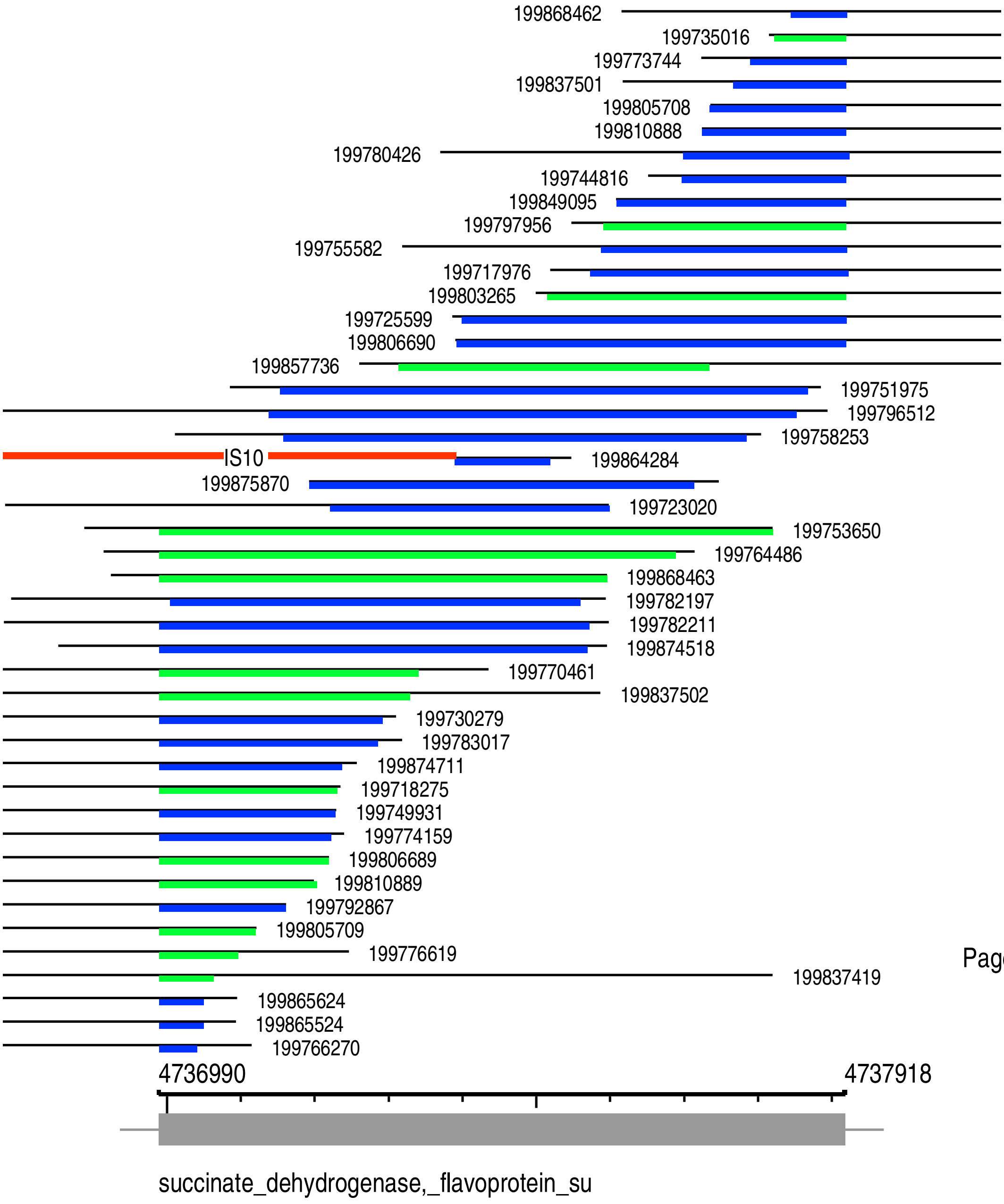

Page: 54

TX160488 Pseudomonas putida kt2440

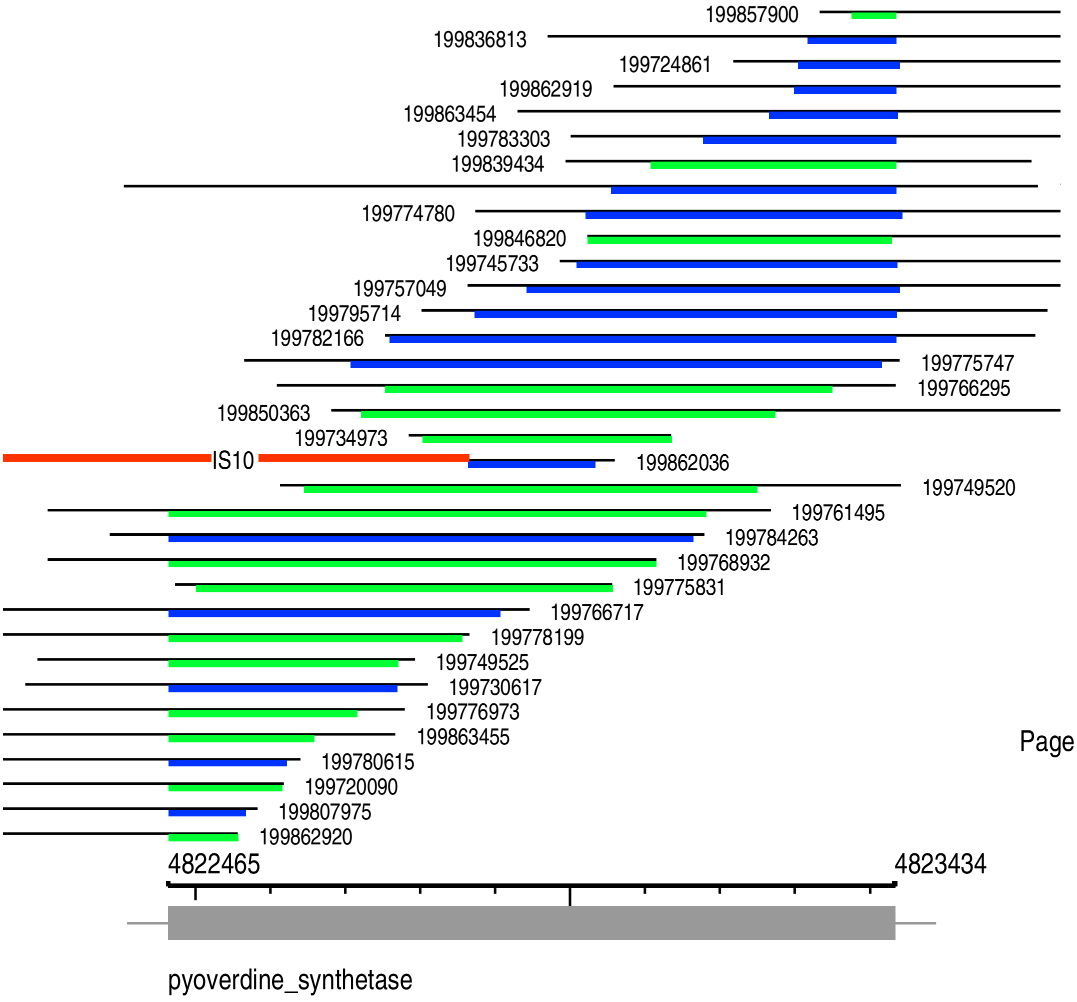

Page: 55

TX160488 Pseudomonas putida kt2440

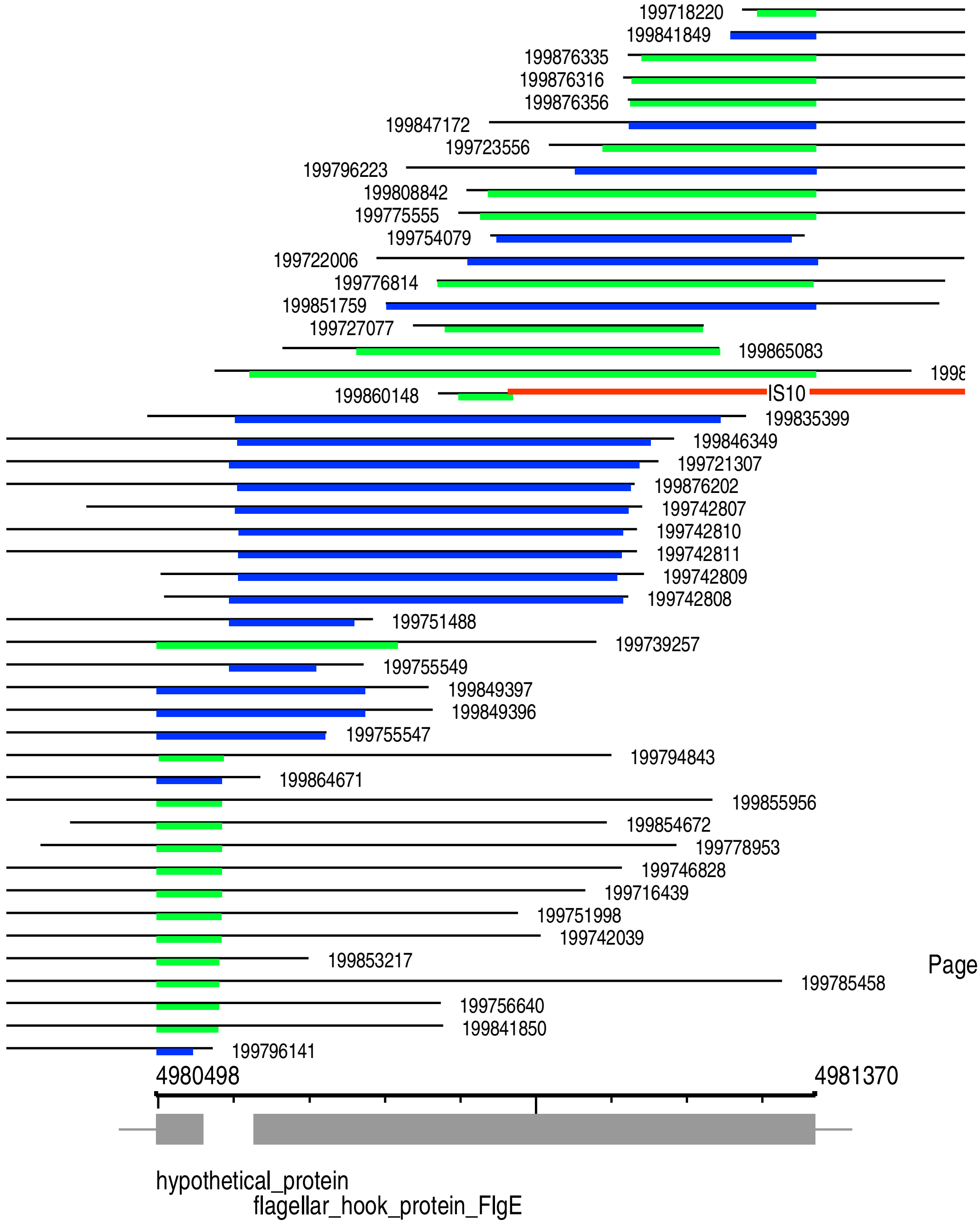

TX160488 Pseudomonas putida kt2440

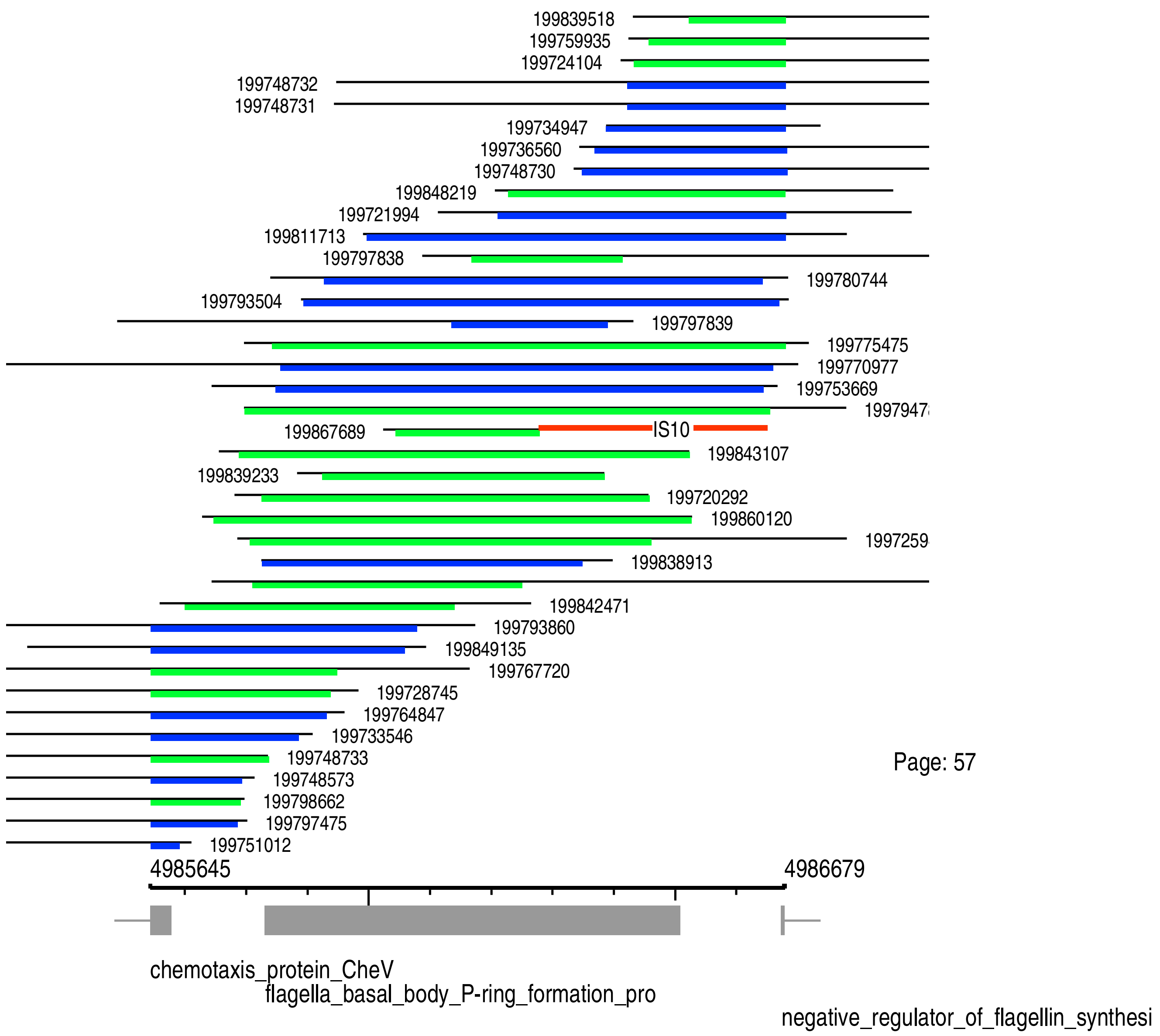

Page: 57

TX160488 Pseudomonas putida kt2440

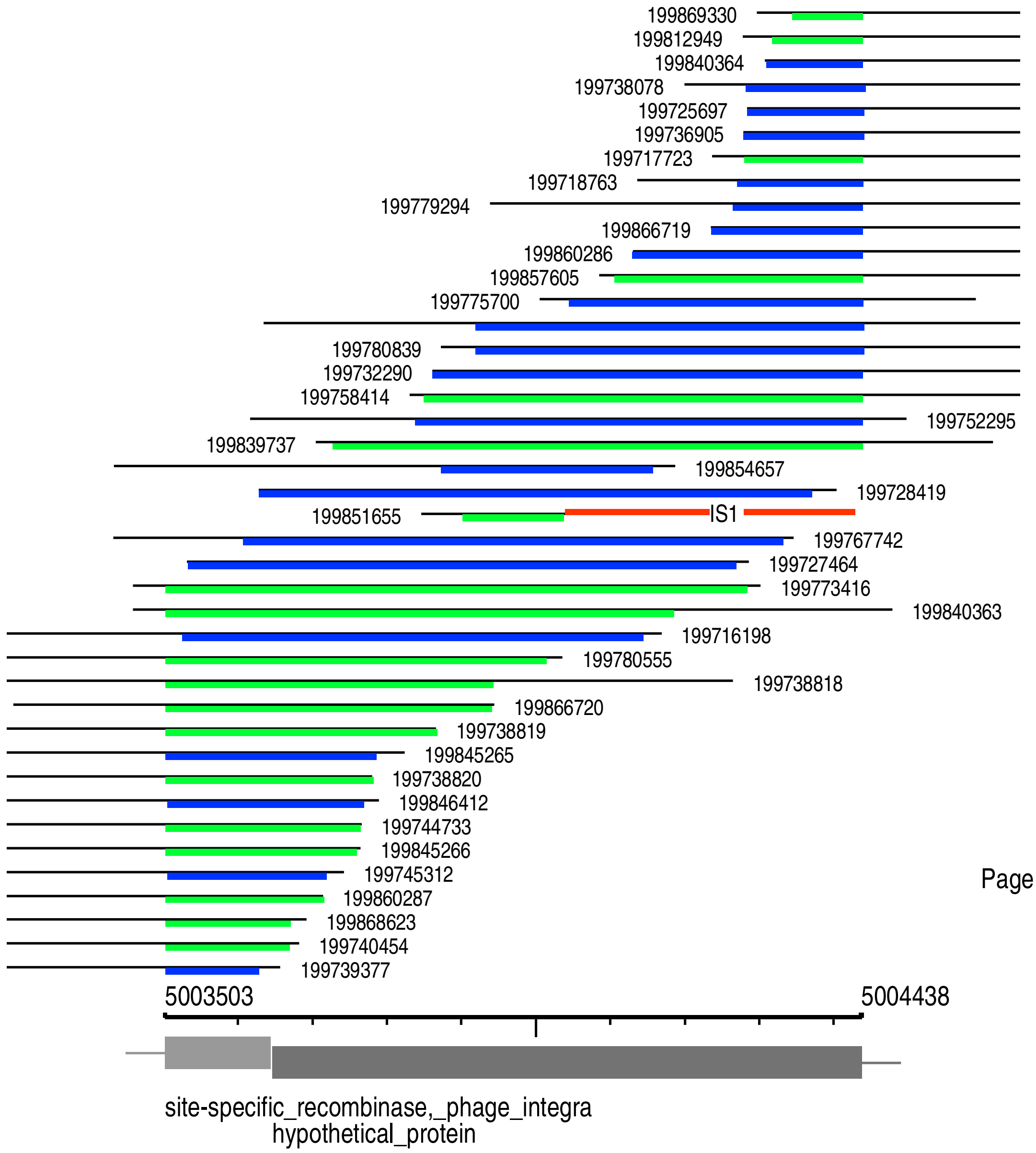

TX160488 Pseudomonas putida kt2440

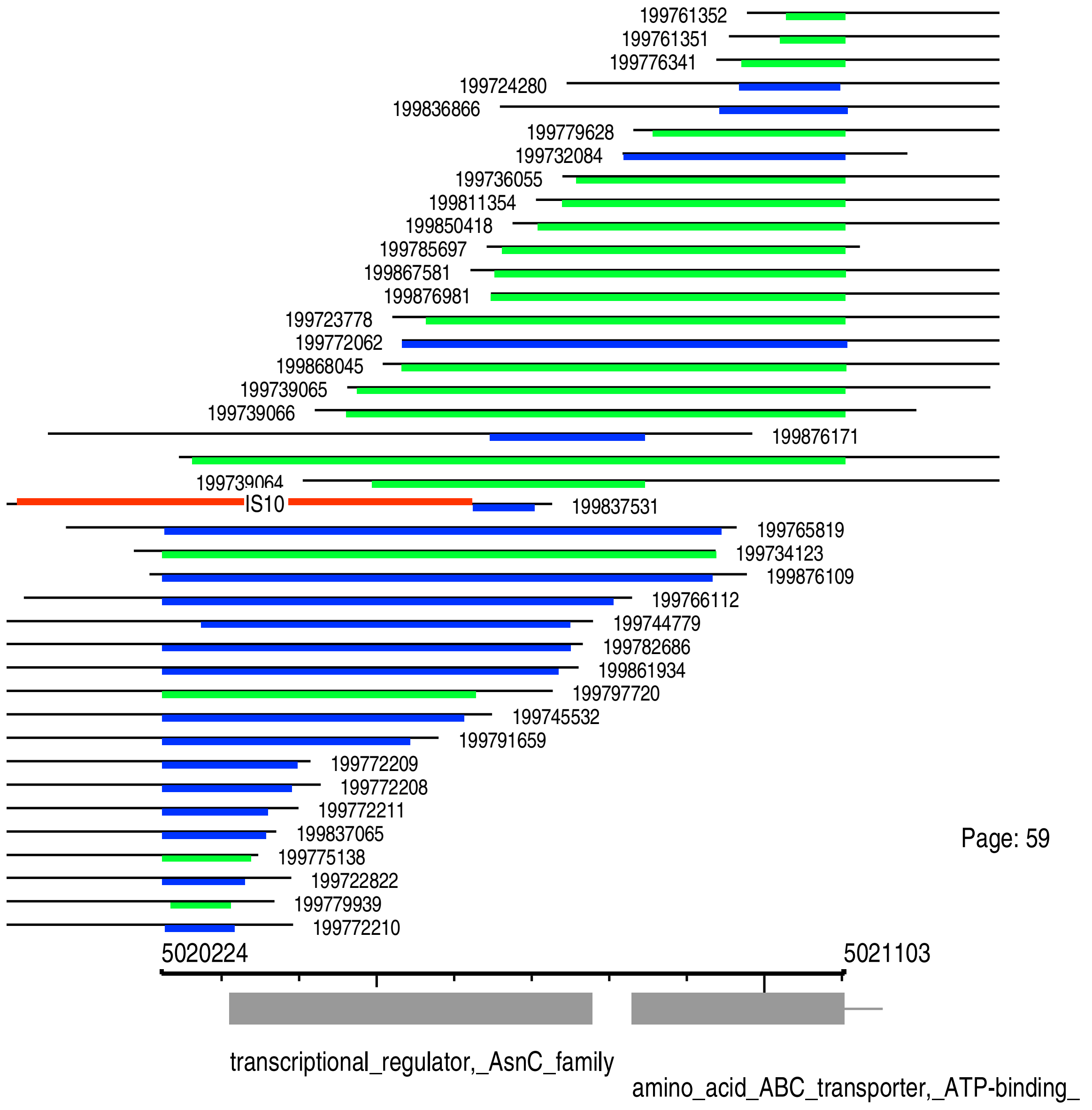

Page: 59

TX160488 Pseudomonas putida kt2440

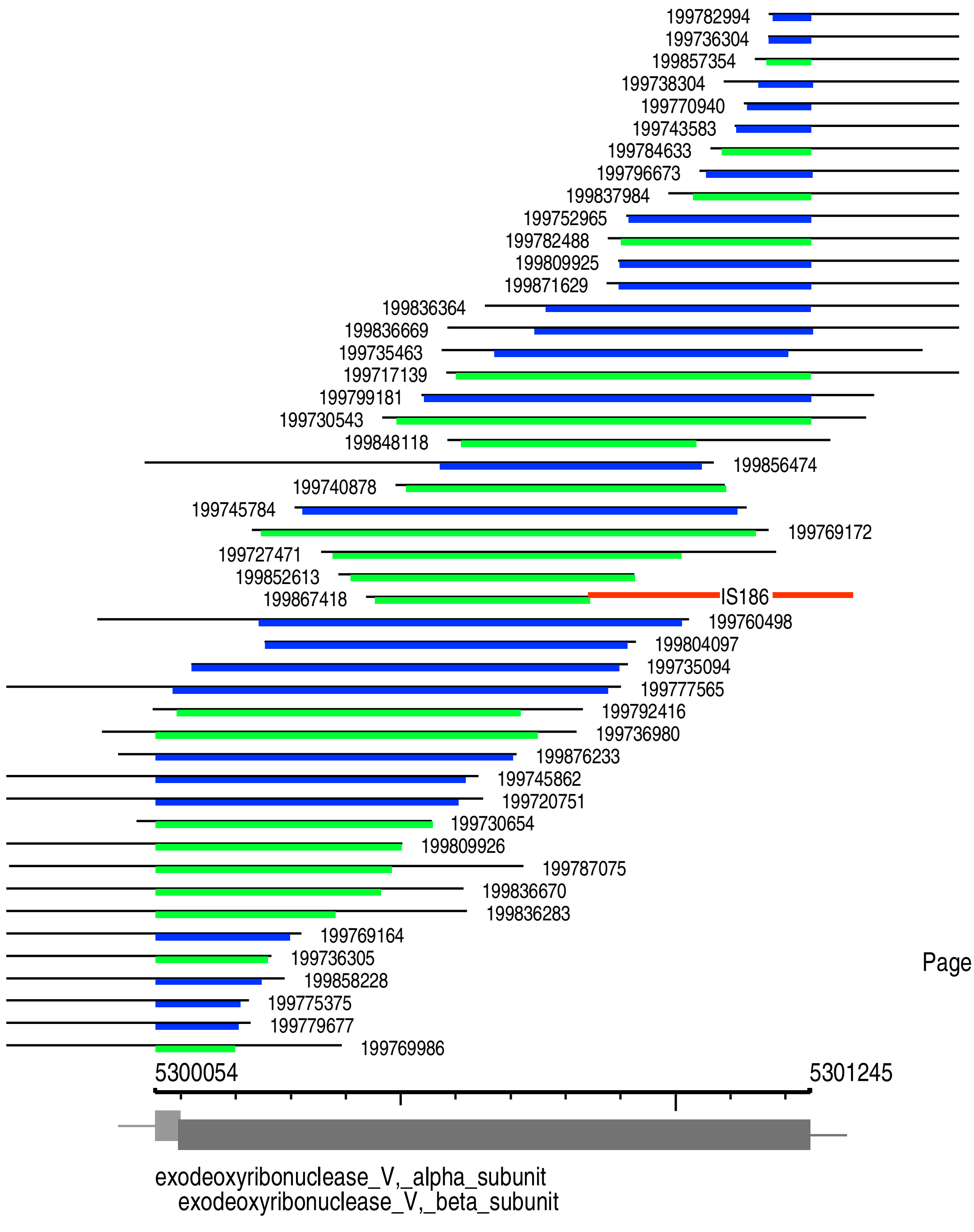

Page: 60

TX160488 Pseudomonas putida kt2440

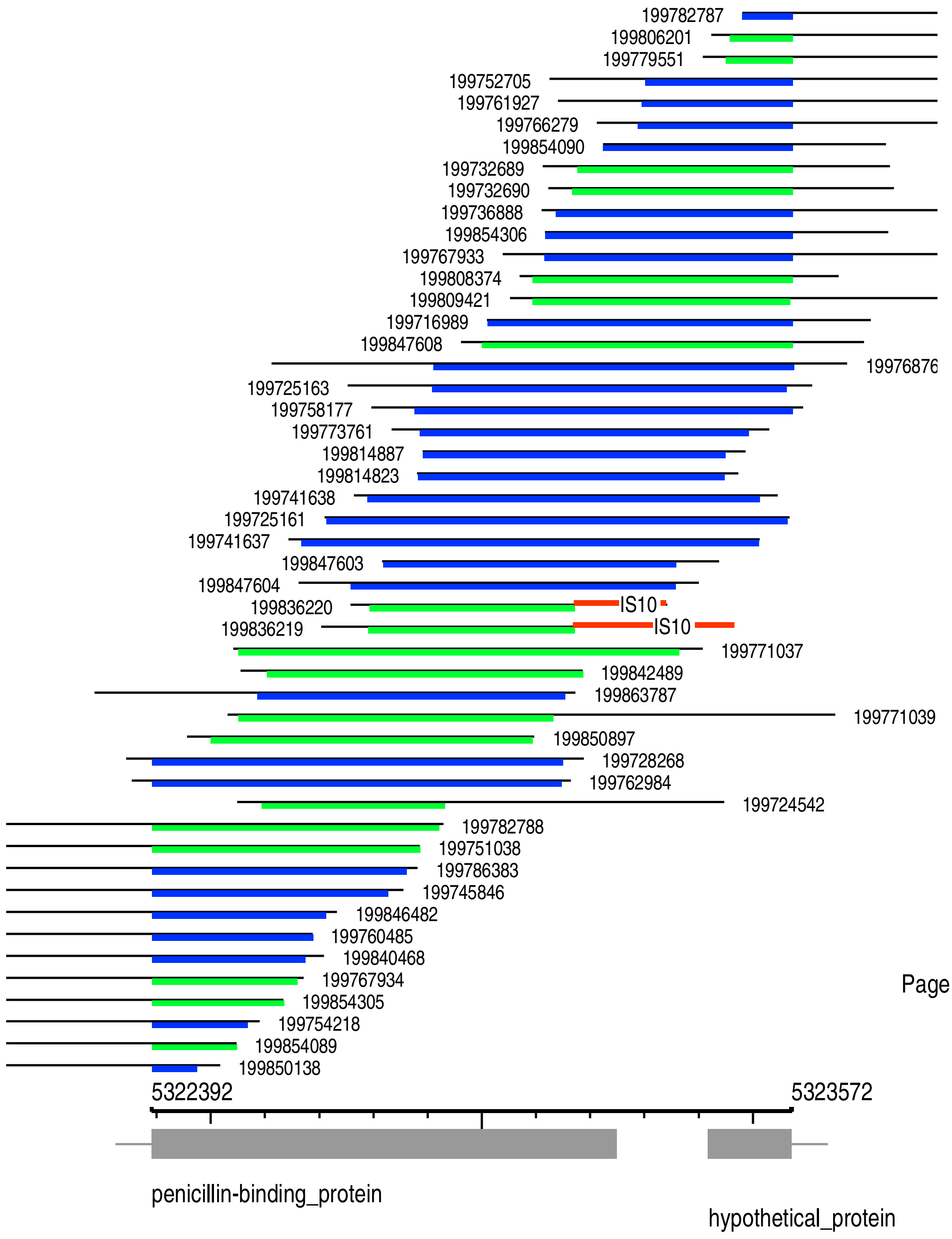

TX160488 Pseudomonas putida kt2440

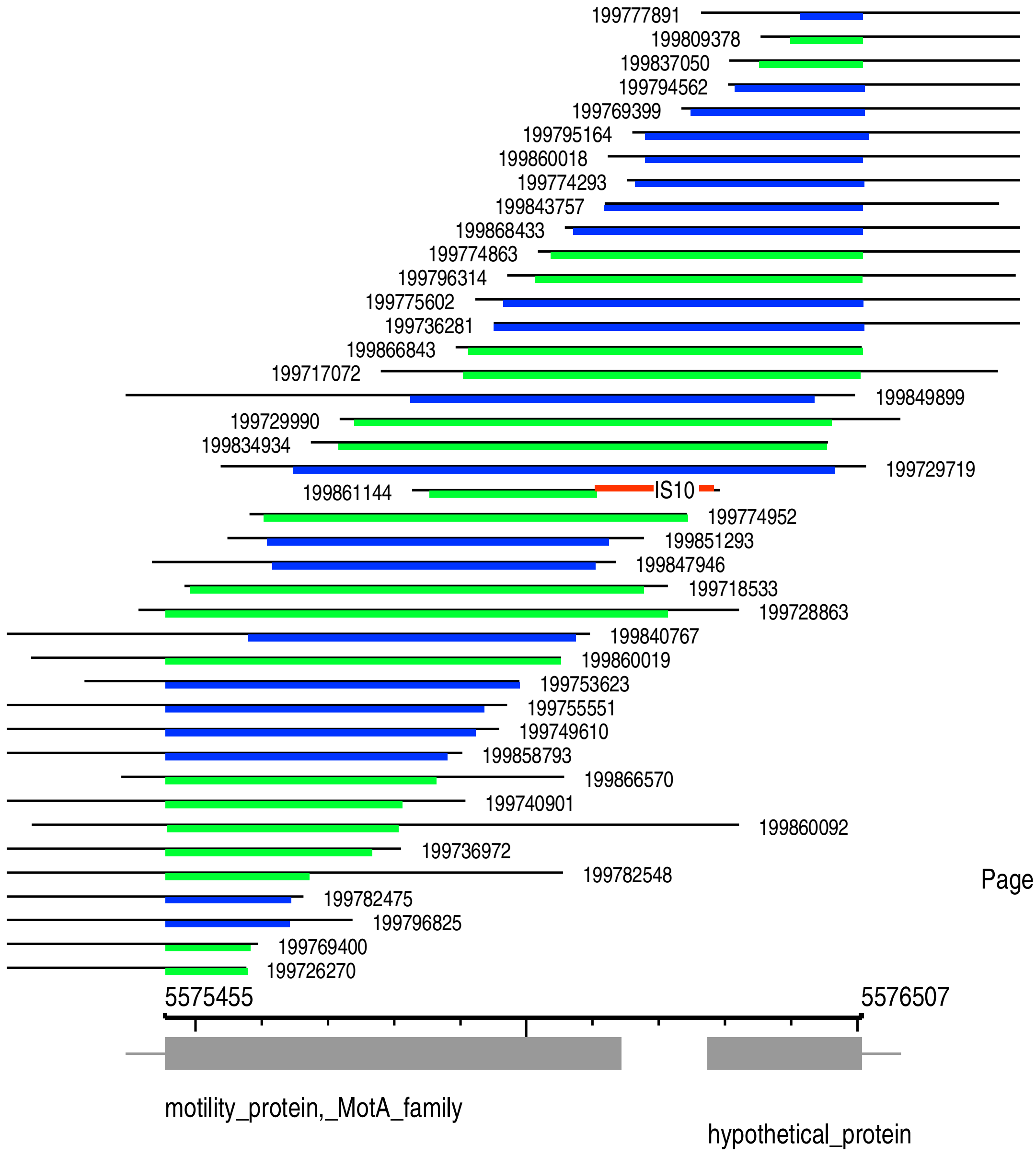

Page: 62

TX160488 Pseudomonas putida kt2440

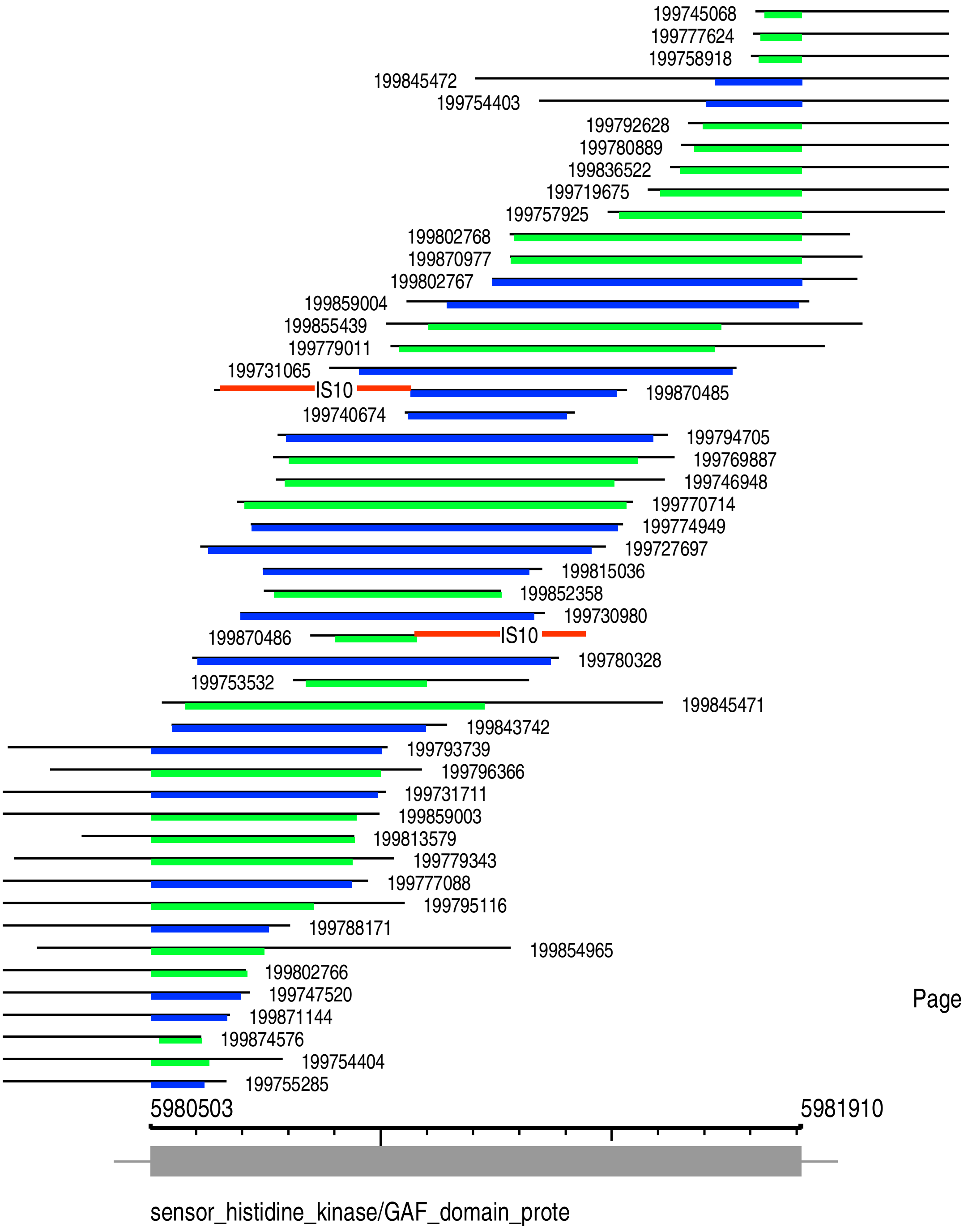

TX160488 Pseudomonas putida kt2440

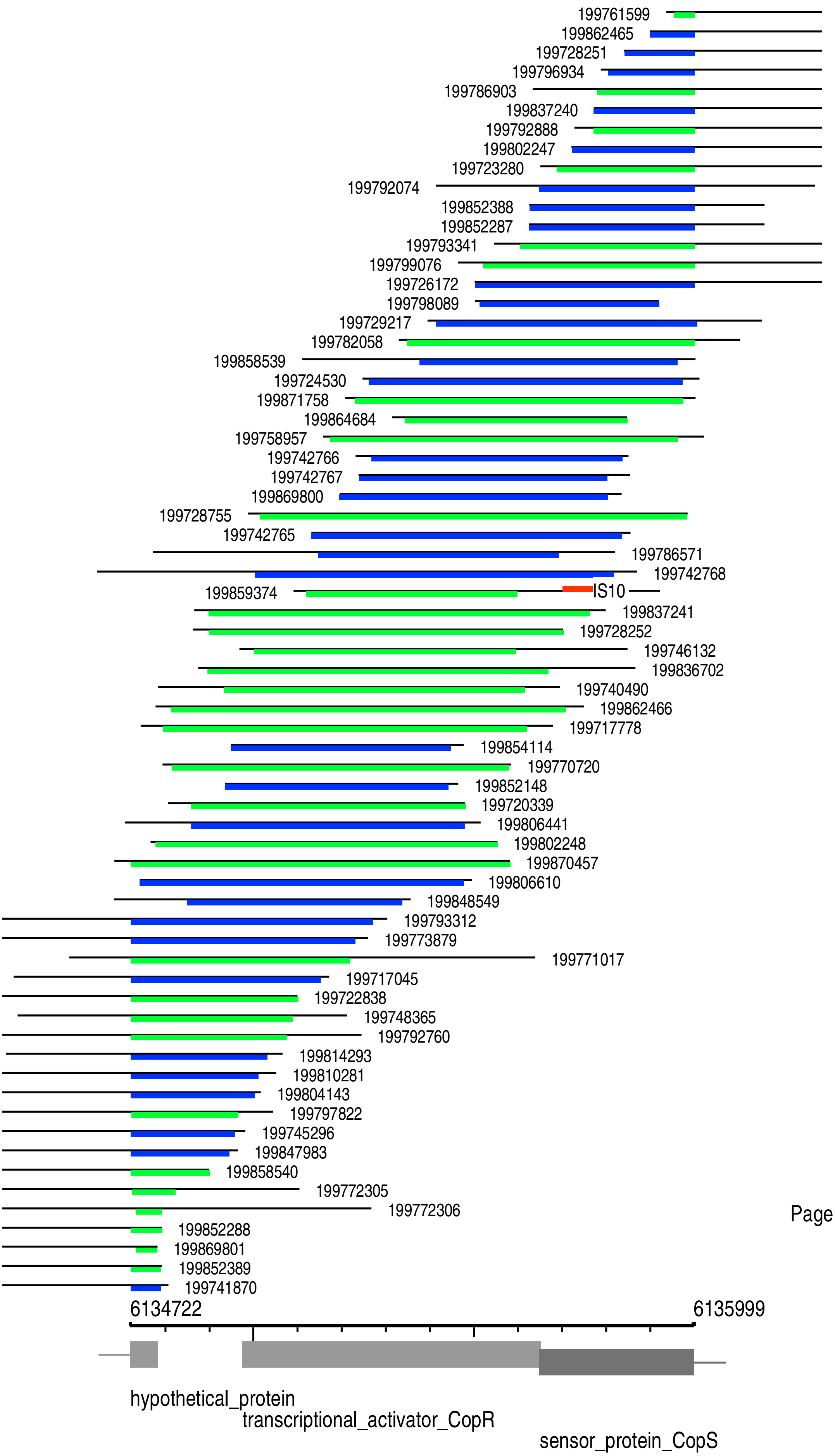

Page: 64

TX160488 Pseudomonas putida kt2440

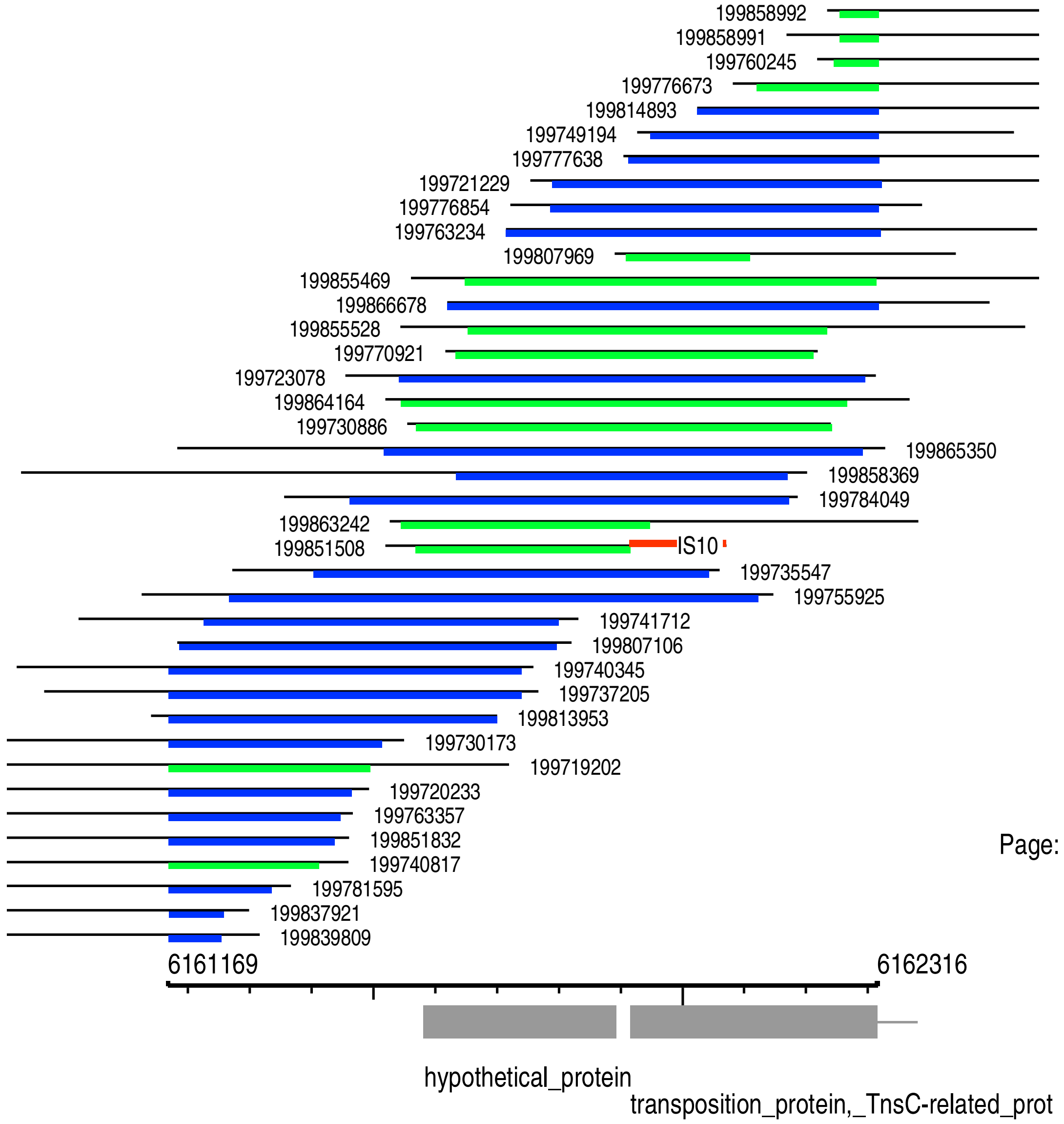

TX160490 Streptococcus pyogenes m1 gas

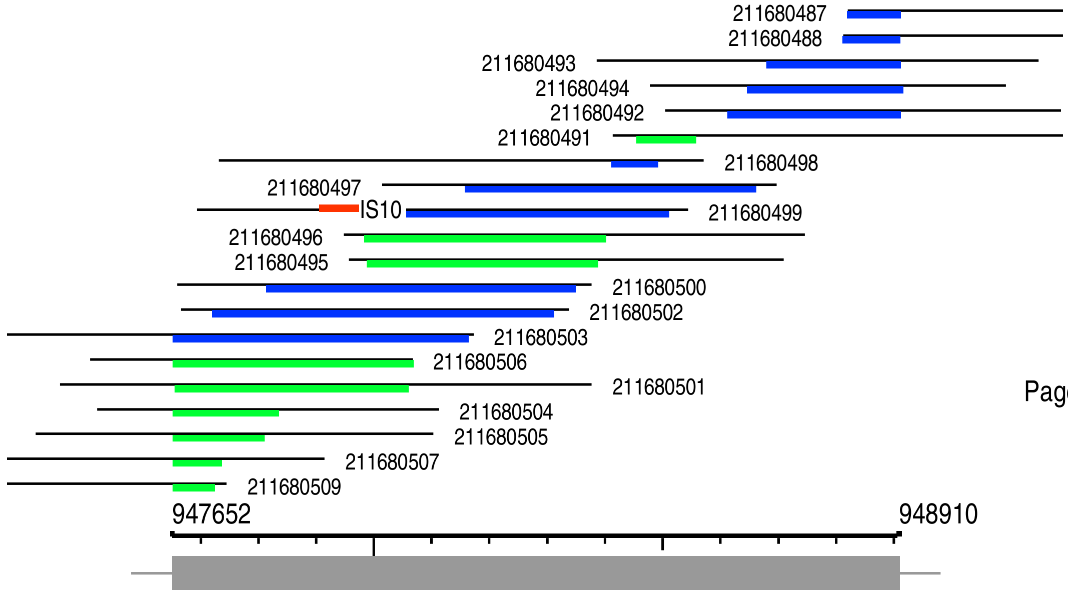

Page: 66

DNA_gyrase_A_subunit Best_Blastp_hit_=_gb|AAF63266.1|__AF2209

TX160490 Streptococcus pyogenes m1 gas

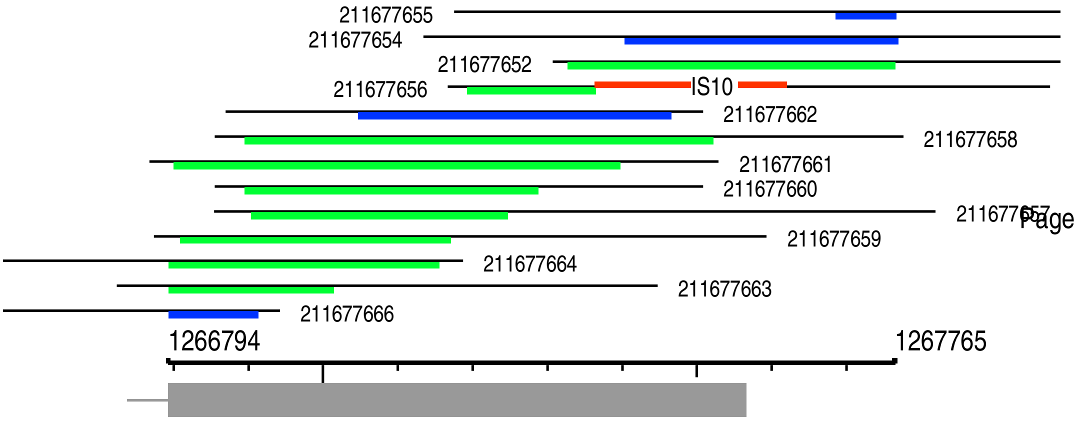

TX170187 Streptococcus pneumoniae tigr4

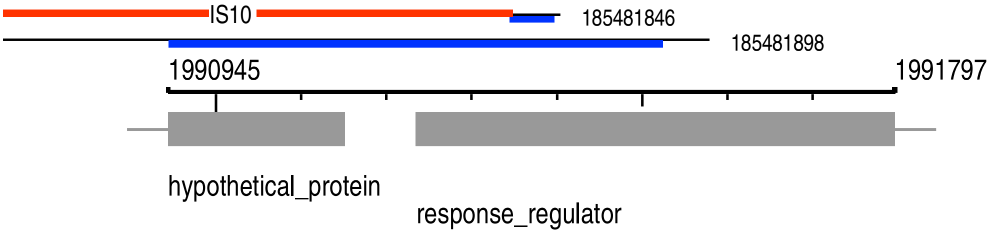

TX170187 Streptococcus pneumoniae tigr4

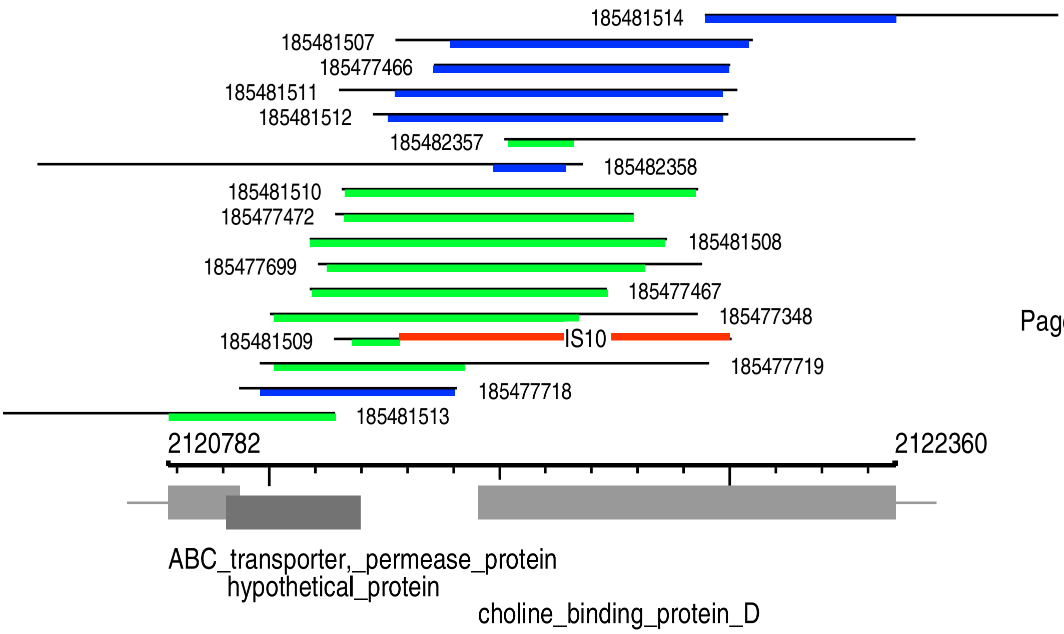

Page: 69

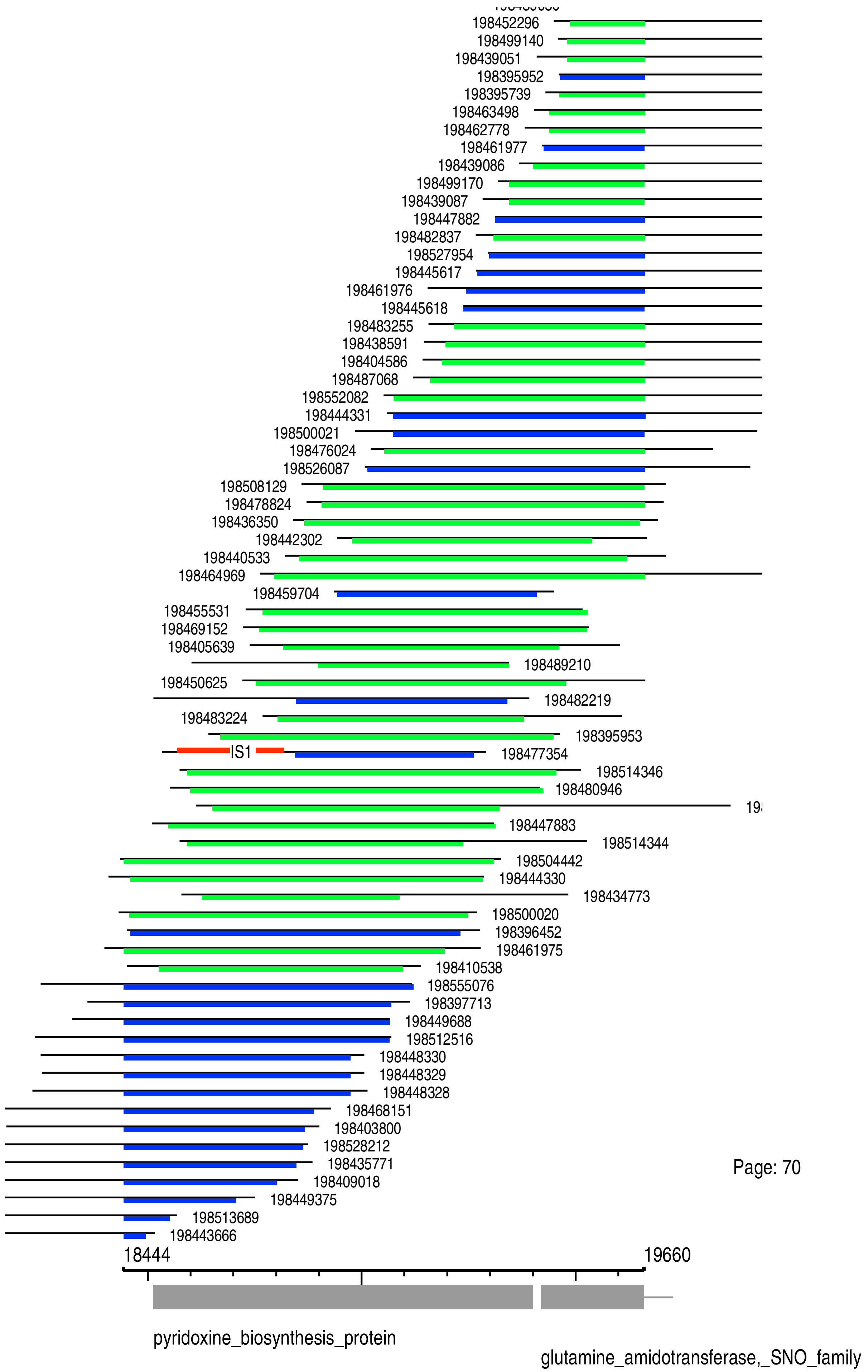
198452296
198499140
198439051
198395952
198395739
198463498
198462778
198461977
198439086
198499170
198439087
198447882
198482837
198527954
198445617
198461976
198445618
198483255
198438591
198404586
198487068
198552082
198444331
198500021
198476024
198526087
198508129
198478824
198436350
198442302
198440533
198464969
198459704
198455531
198469152
198405639
198489210
198450625
198482219
198483224
198395953
IS1
198477354
198514346
198480946
198447883
198514344
198504442
198444330
198434773
198500020
198396452
198461975
198410538
198555076
198397713
198449688
198512516
198448330
198448329
198448328
198468151
198403800
198528212
198435771
198409018
198449375
198513689
198443666
Page: 70
18444
19660
pyridoxine_biosynthesis_protein
glutamine_amidotransferase,_SNO_family

TX198094 Bacillus anthracis str ames

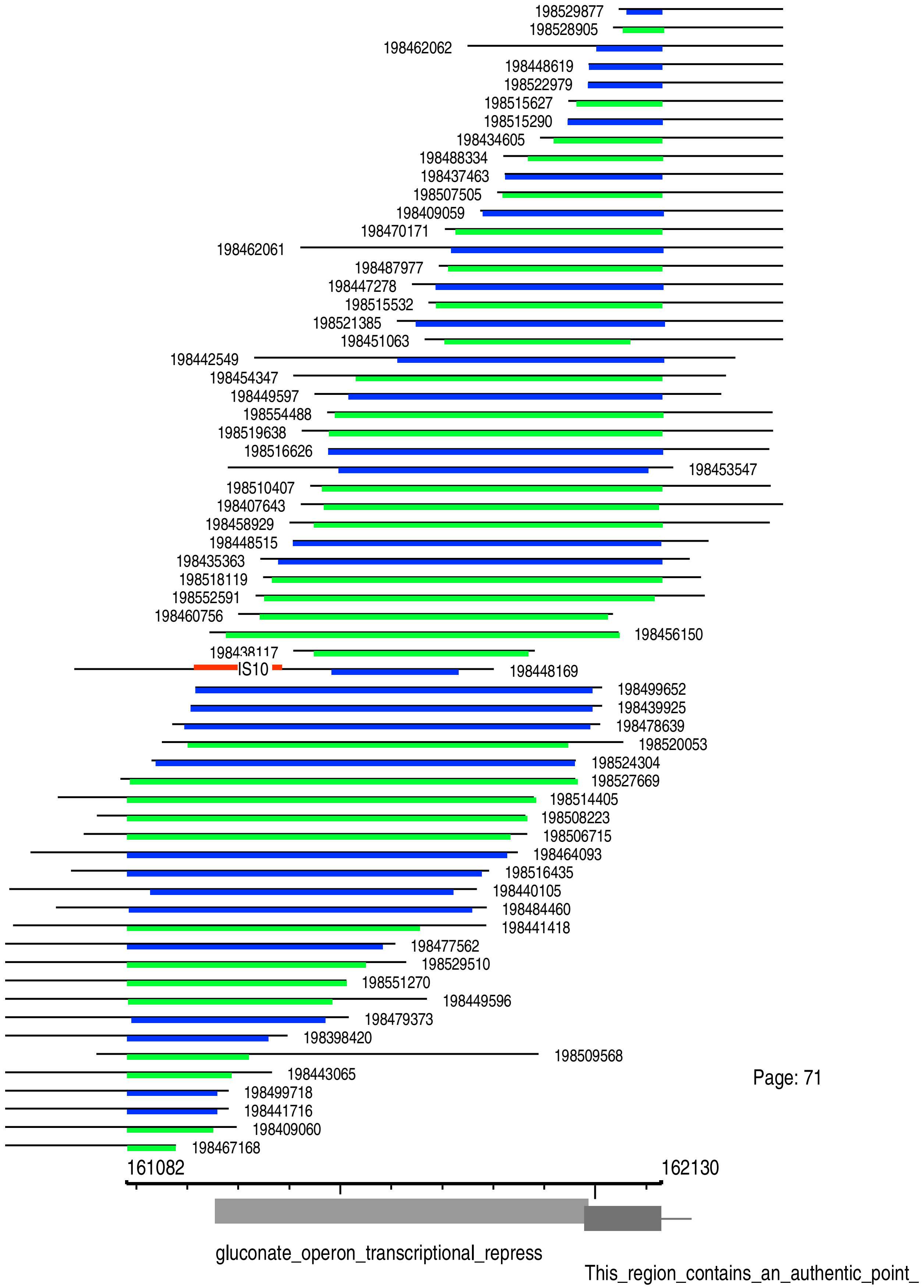

TX198094 Bacillus anthracis str ames

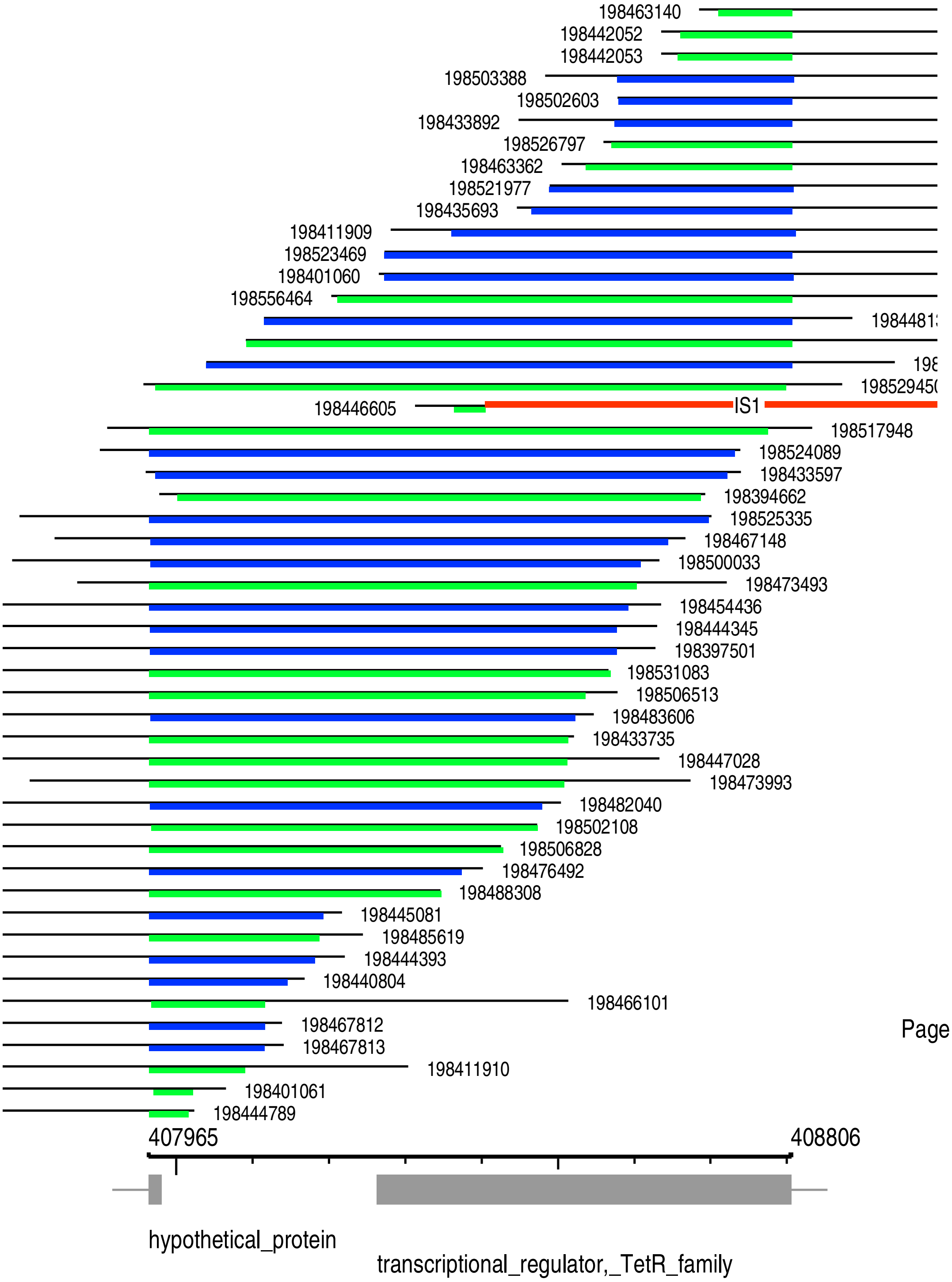

Page: 72

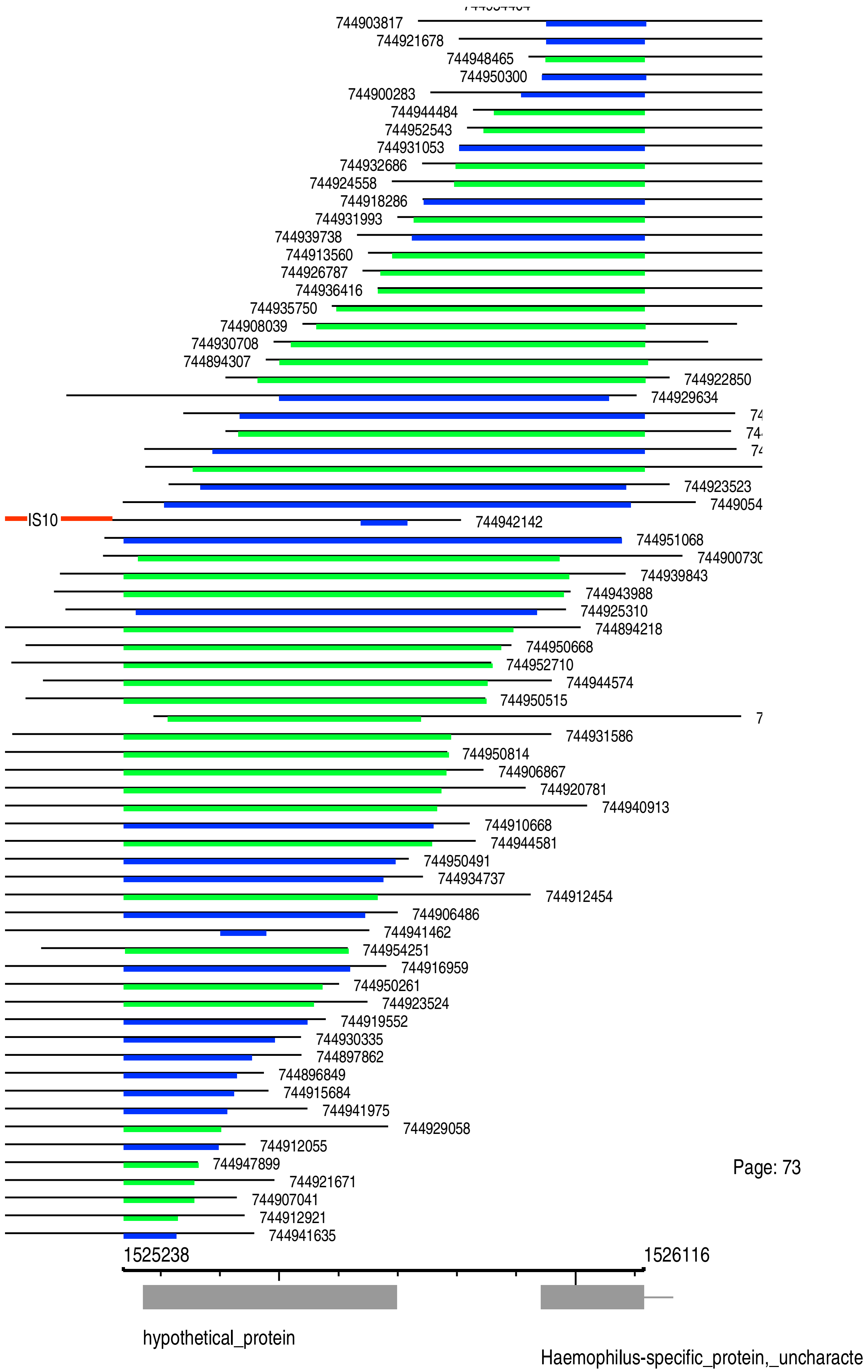
744903817
744921678
744948465
744950300
744900283
744944484
744952543
744931053
744932686
744924558
744918286
744931993
744939738
744913560
744926787
744936416
744935750
744908039
744930708
744894307
744922850
744929634
744923523
IS10
744942142
744951068
744939843
744943988
744925310
744894218
744950668
744952710
744944574
744950515
744931586
744950814
744906867
744920781
744940913
744910668
744944581
744950491
744934737
744912454
744906486
744941462
744954251
744916959
744950261
744923524
744919552
744930335
744897862
744896849
744915684
744941975
744929058
744912055
744947899
744921671
744907041
744912921
744941635
1525238
1526116
hypothetical_protein
Haemophilus-specific_protein,_uncharacte

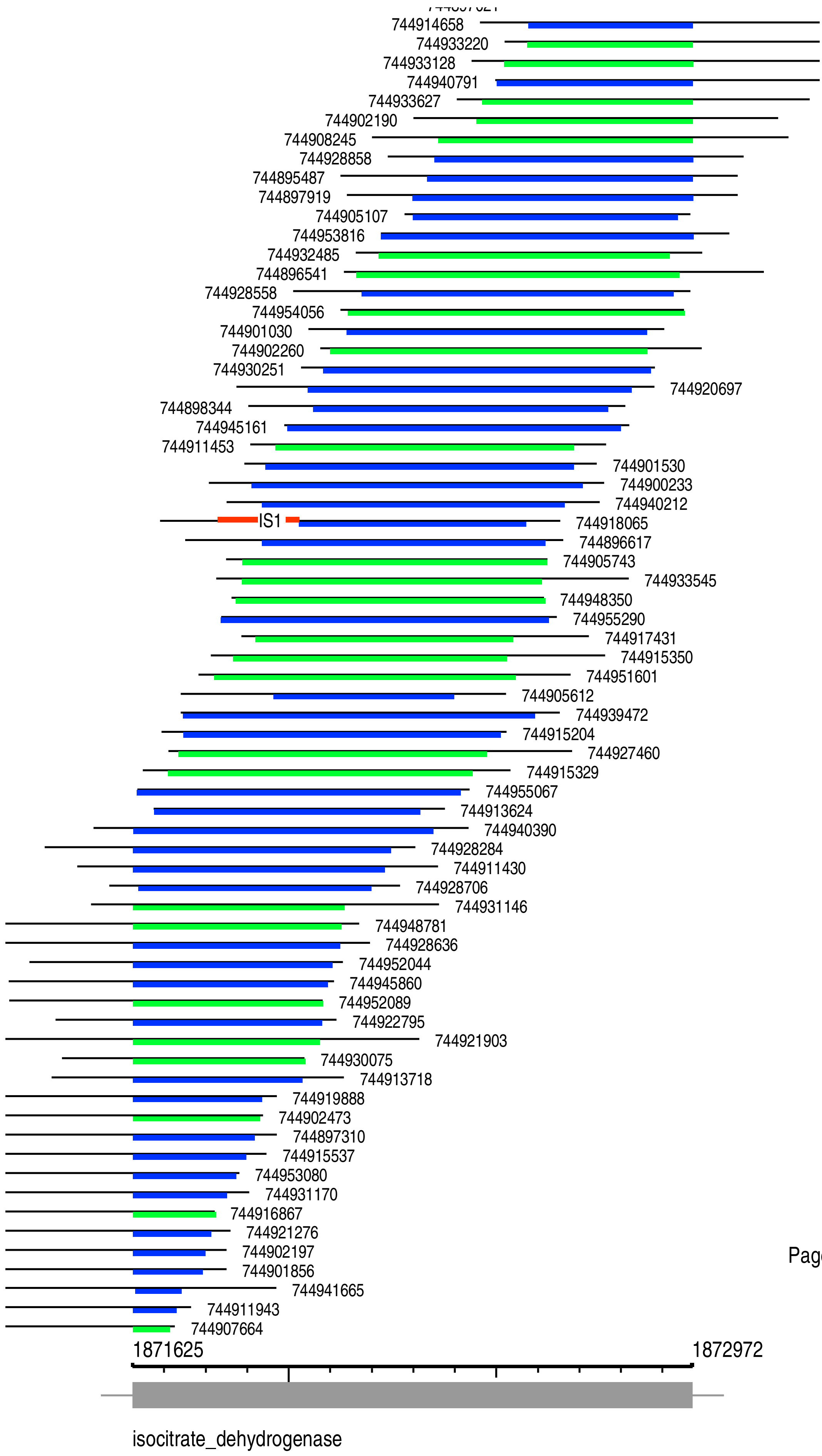
744914658
744933220
744933128
744940791
744933627
744902190
744908245
744928858
744895487
744897919
744905107
744953816
744932485
744896541
744928558
744954056
744901030
744902260
744930251
744920697
744898344
744945161
744911453
744901530
744900233
744940212
IS1
744918065
744896617
744905743
744933545
744948350
744955290
744917431
744915350
744951601
744905612
744939472
744915204
744927460
744915329
744955067
744913624
744940390
744928284
744911430
744928706
744931146
744948781
744928636
744952044
744945860
744952089
744922795
744921903
744930075
744913718
744919888
744902473
744897310
744915537
744953080
744931170
744916867
744921276
744902197
744901856
744941665
744911943
744907664
1871625
1872972
isocitrate_dehydrogenase

Page: 74

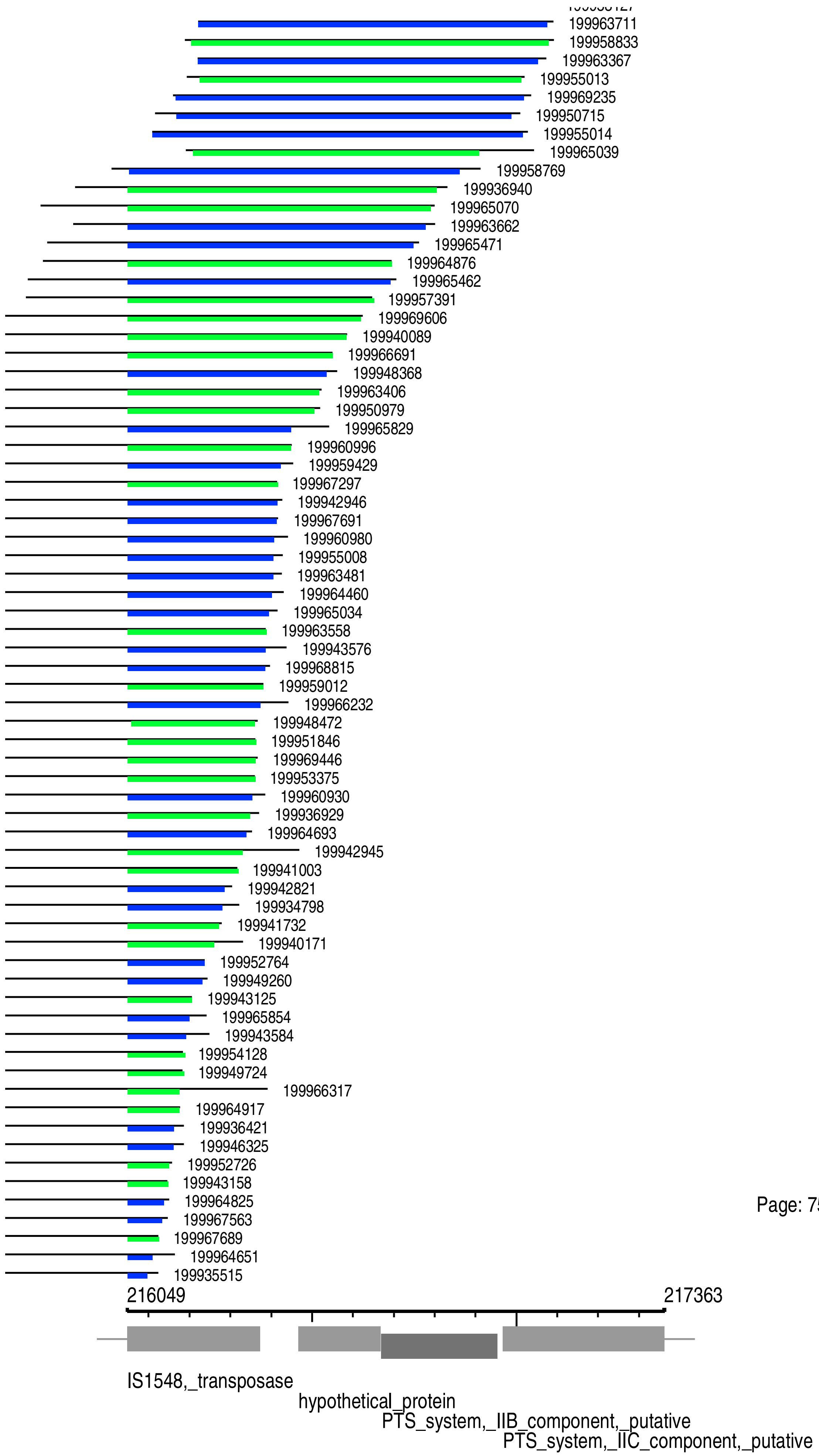
199963711
199958833
199963367
199955013
199969235
199950715
199955014
199965039
199958769
199936940
199965070
199963662
199965471
199964876
199965462
199957391
199969606
199940089
199966691
199948368
199963406
199950979
199965829
199960996
199959429
199967297
199942946
199967691
199960980
199955008
199963481
199964460
199965034
199963558
199943576
199968815
199959012
199966232
199948472
199951846
199969446
199953375
199960930
199936929
199964693
199942945
199941003
199942821
199934798
199941732
199940171
199952764
199949260
199943125
199965854
199943584
199954128
199949724
199966317
199964917
199936421
199946325
199952726
199943158
199964825
199967563
199967689
199964651
199935515
216049
217363
IS1548,_transposase
hypothetical_protein
PTS_system,_IIB_component,_putative
PTS_system,_IIC_component,_putative

TX208435 Streptococcus agalactiae 2603v r

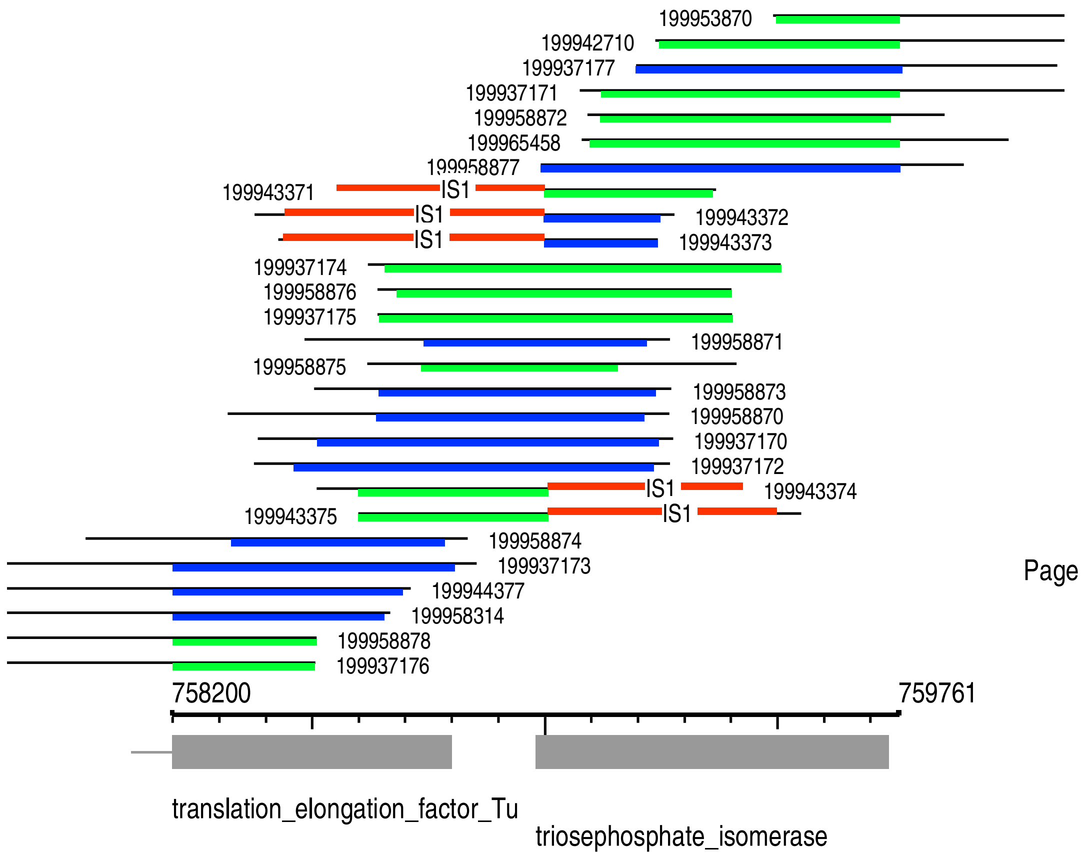

TX208435 Streptococcus agalactiae 2603v r

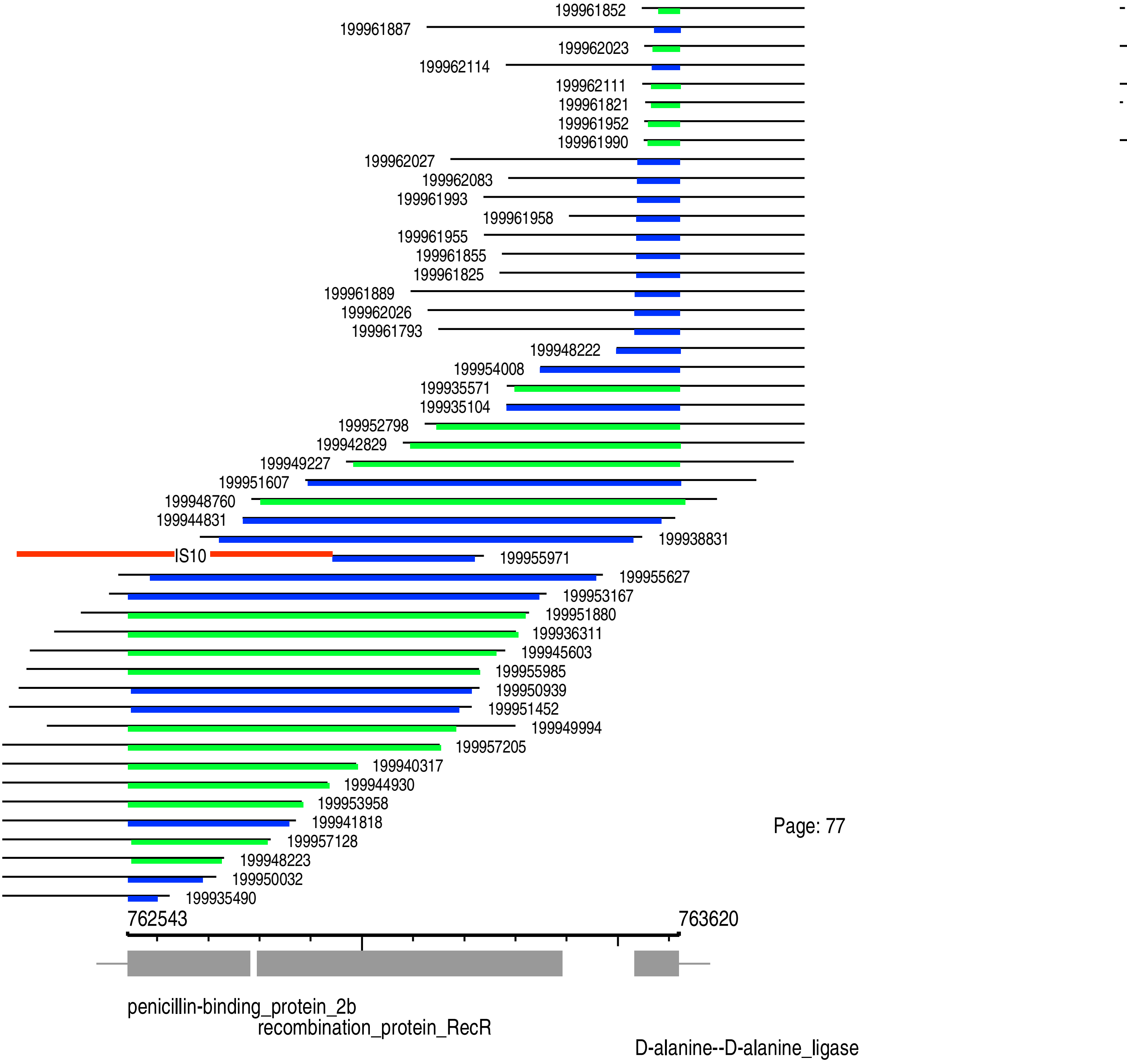

Page: 77

TX208435 Streptococcus agalactiae 2603v r

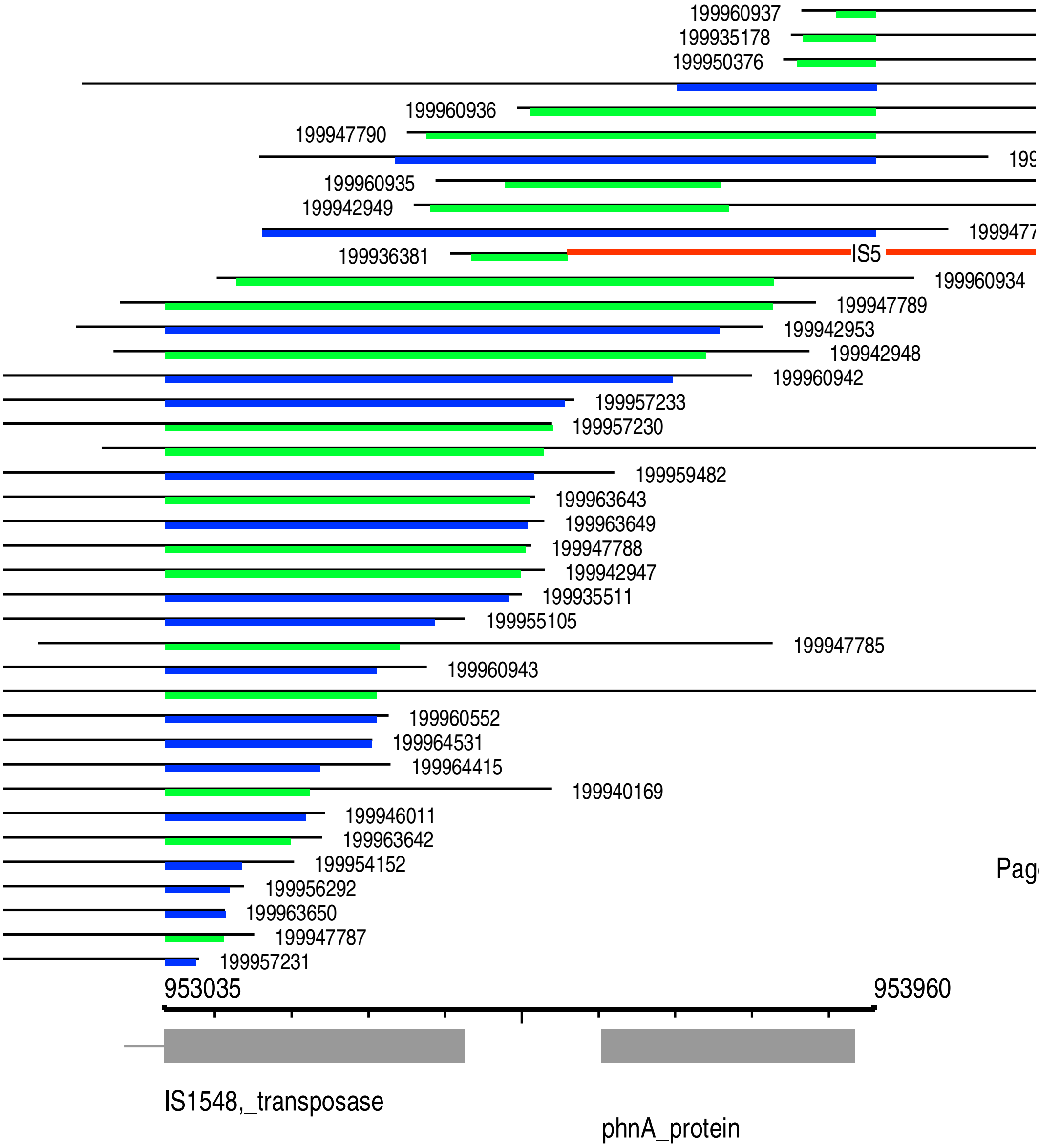

Page: 78

TX208435 Streptococcus agalactiae 2603v r

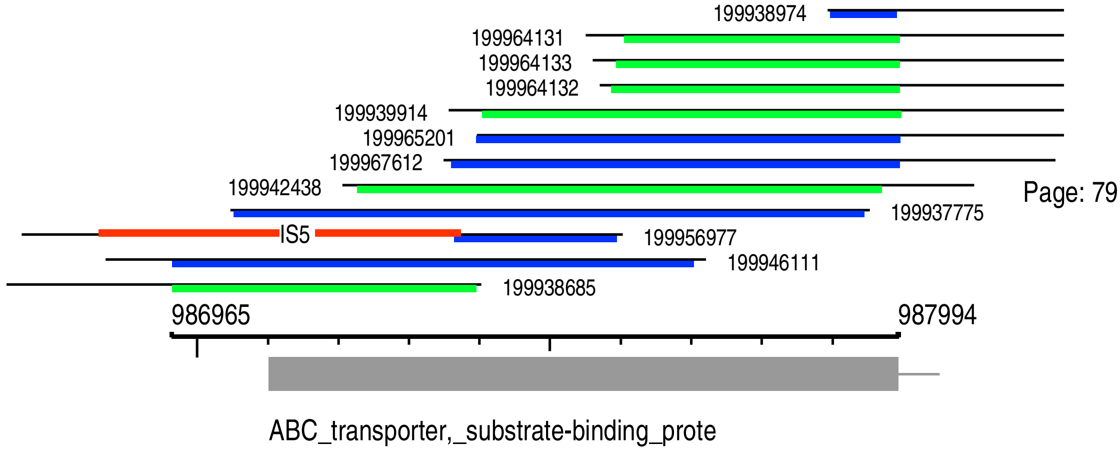

Page: 79

TX208435 Streptococcus agalactiae 2603v r

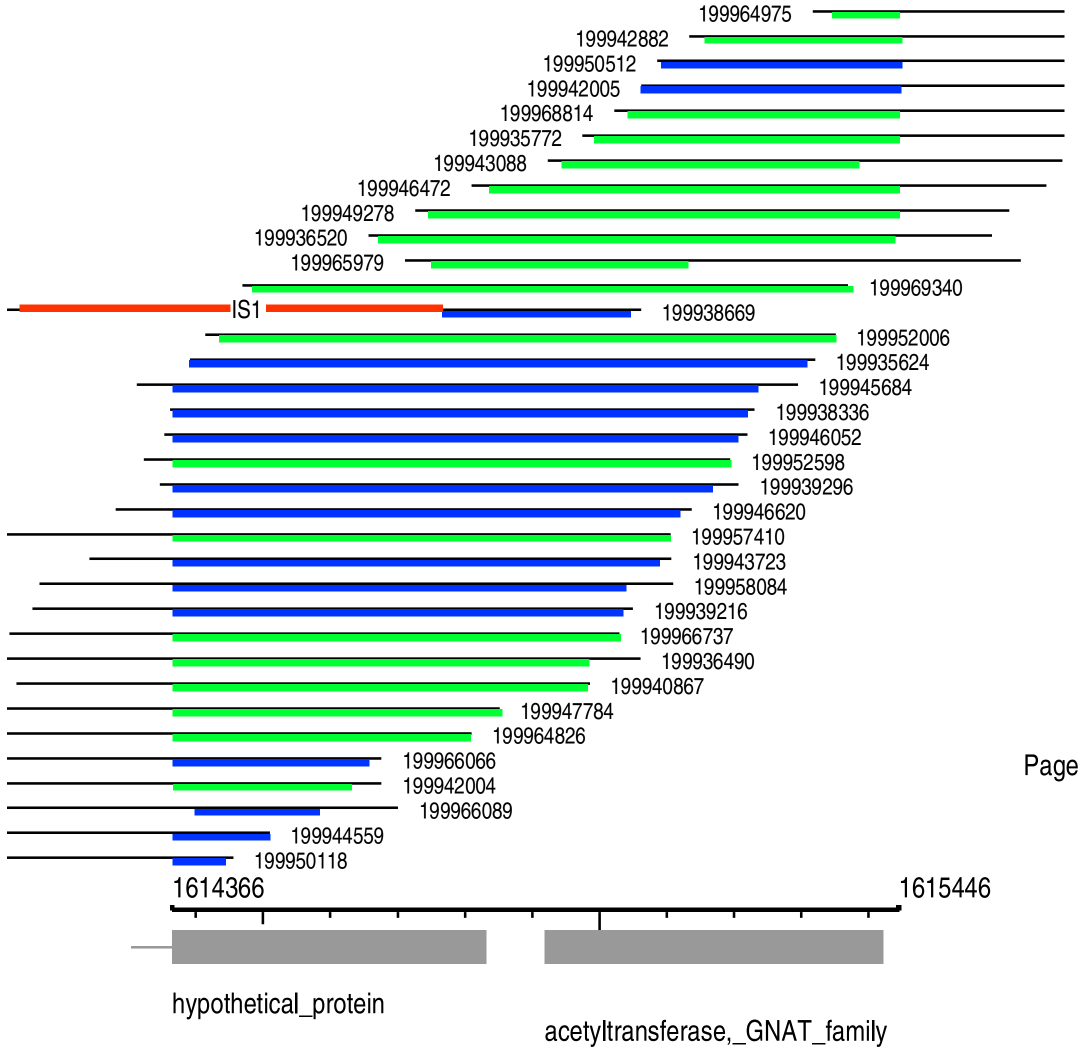

TX208435 Streptococcus agalactiae 2603v r

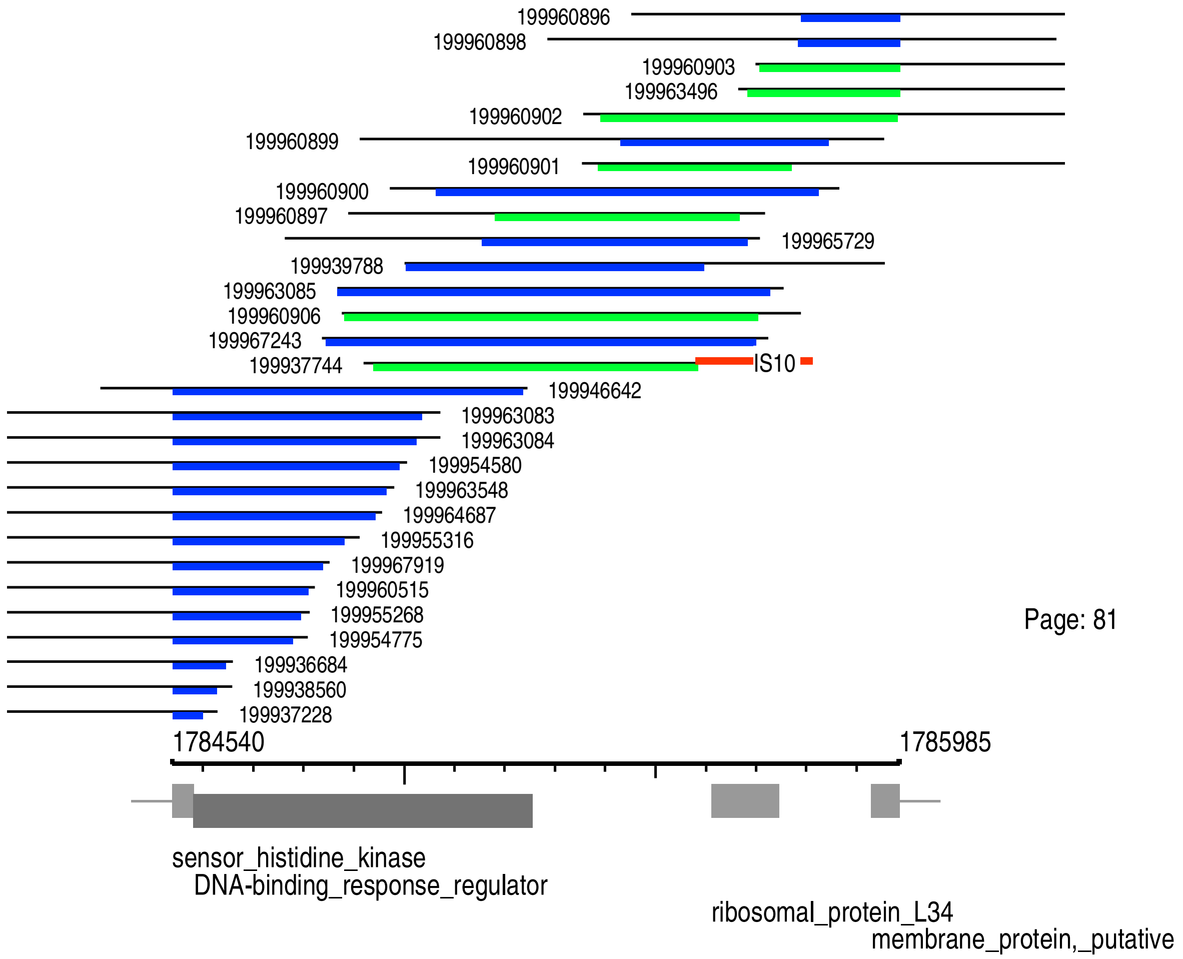

Page: 81

TX208435 Streptococcus agalactiae 2603v r

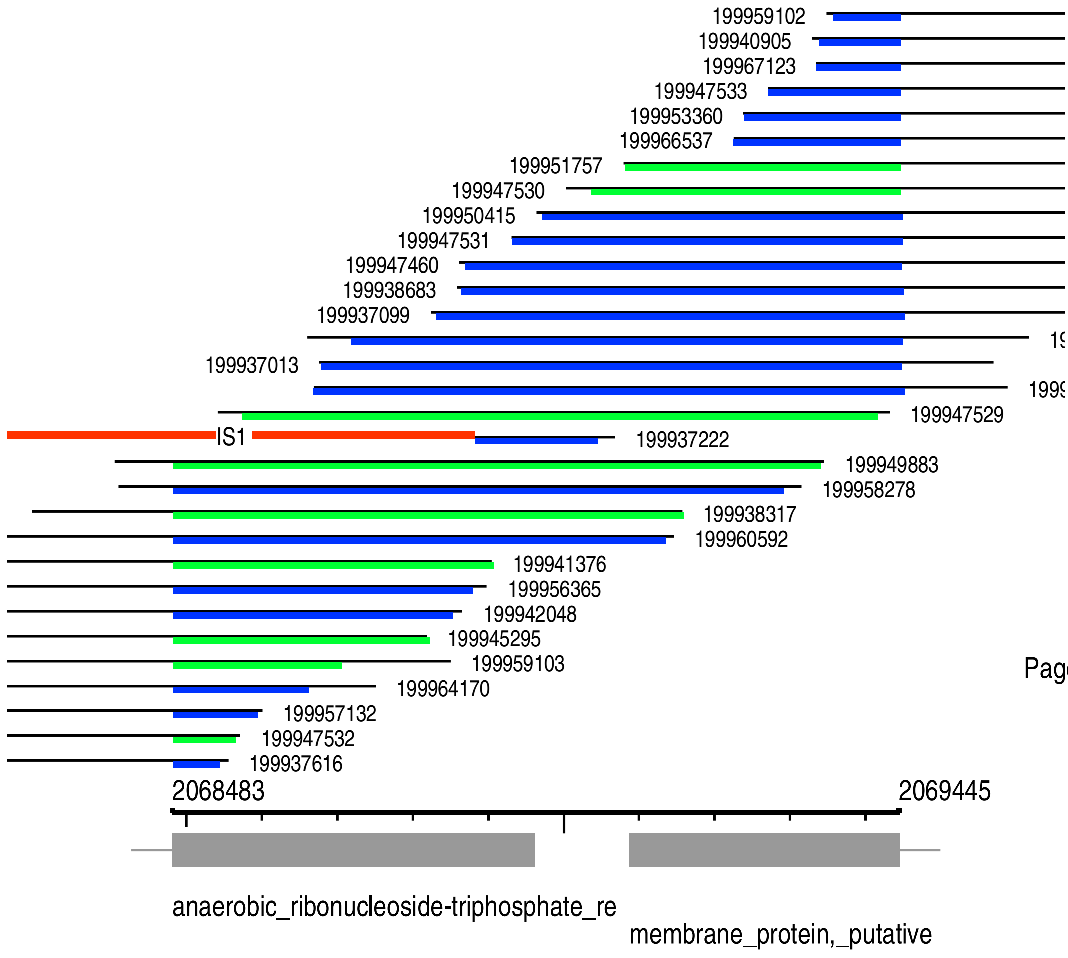

TX208435 Streptococcus agalactiae 2603v r

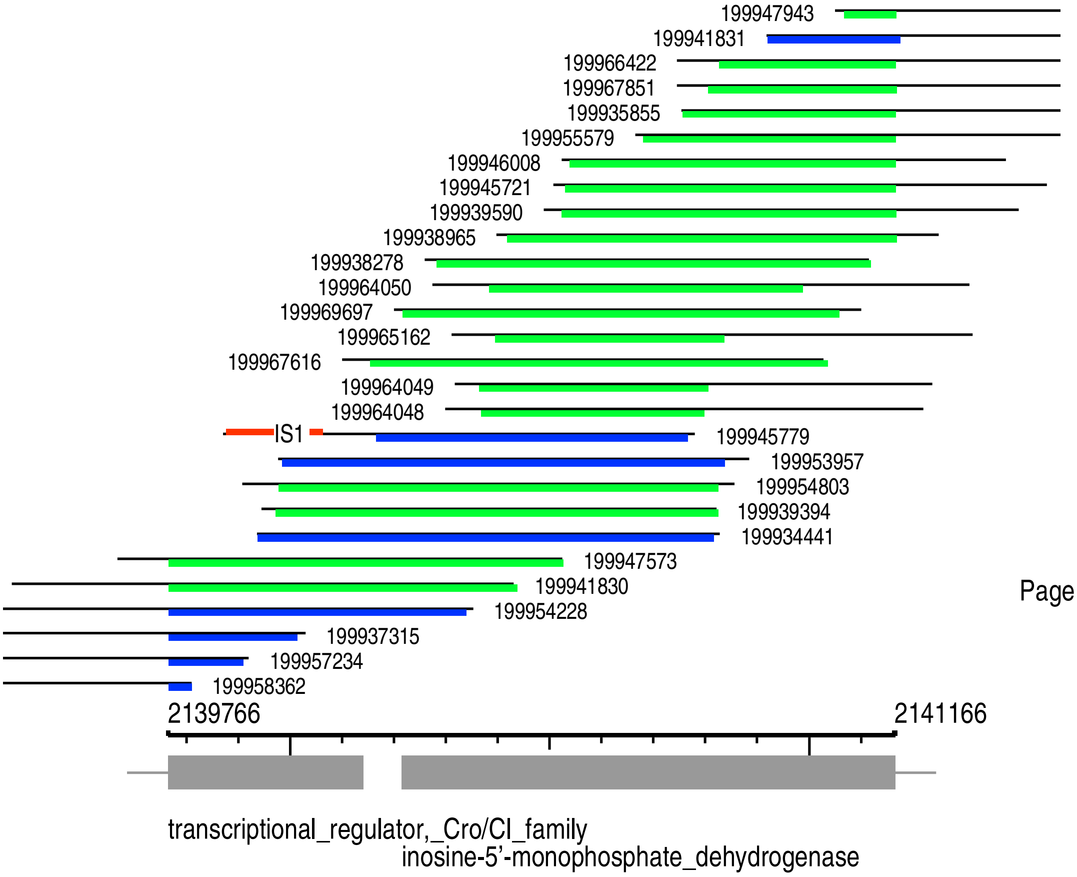

TX226185 Enterococcus faecalis v583

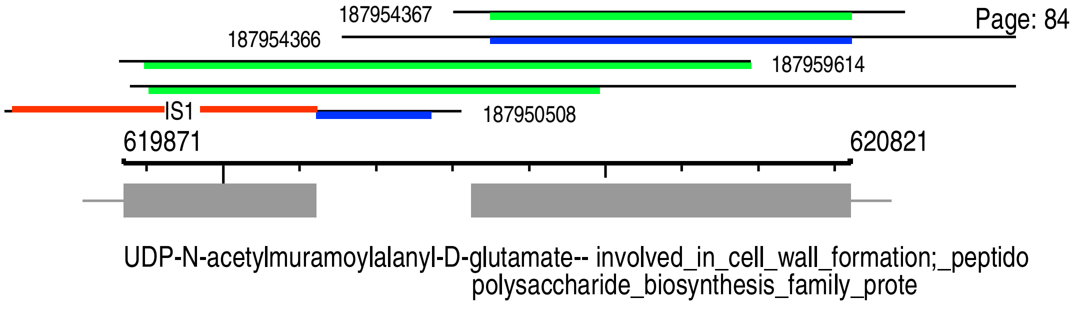

TX226185 Enterococcus faecalis v583

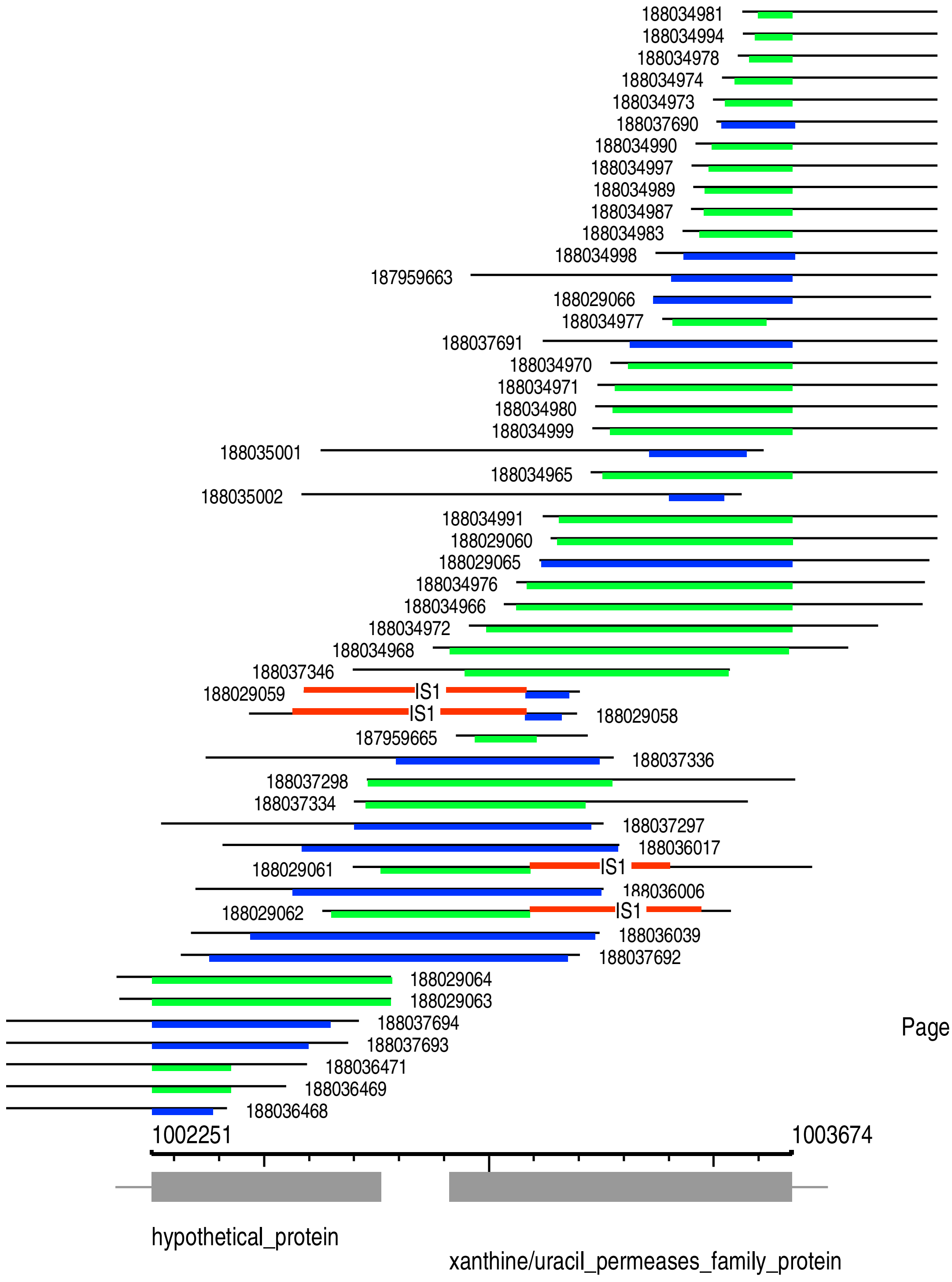

TX226185 Enterococcus faecalis v583

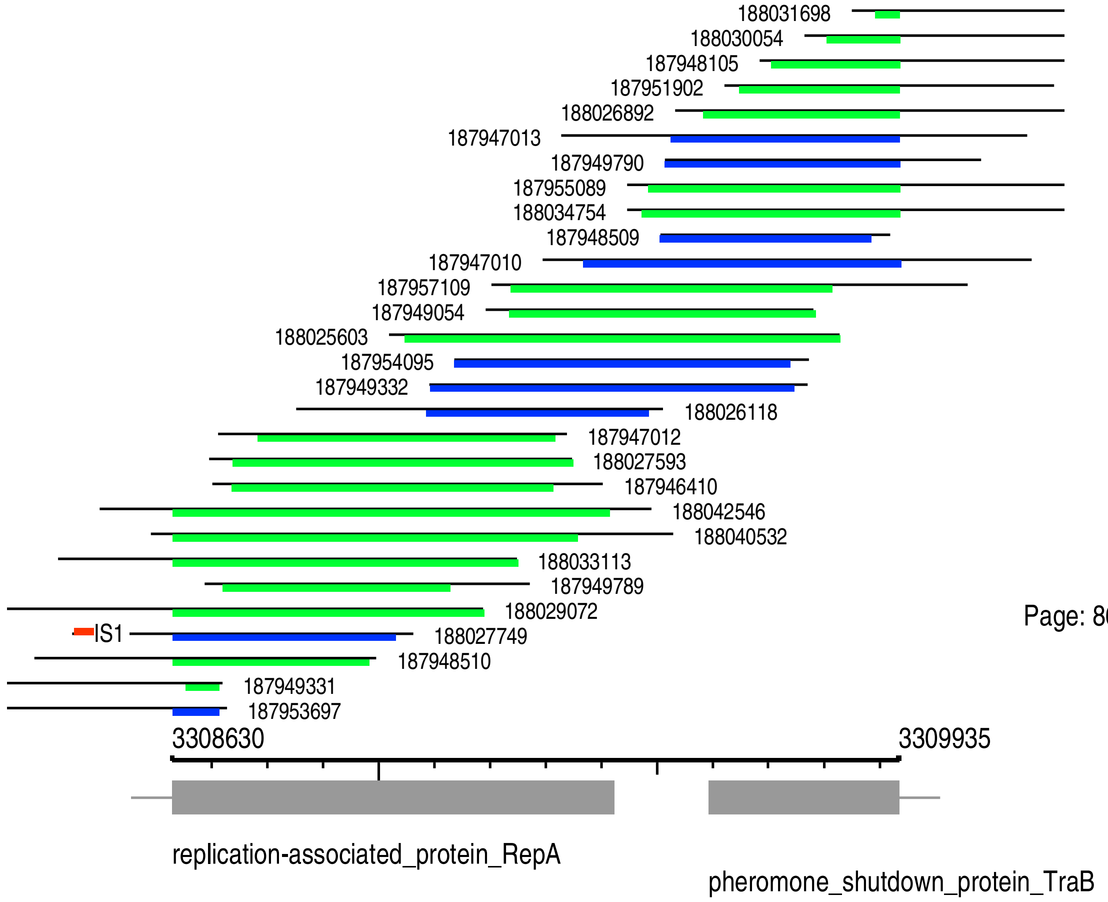

Page: 86

TX227377 Coxiella burnetii rsa 493

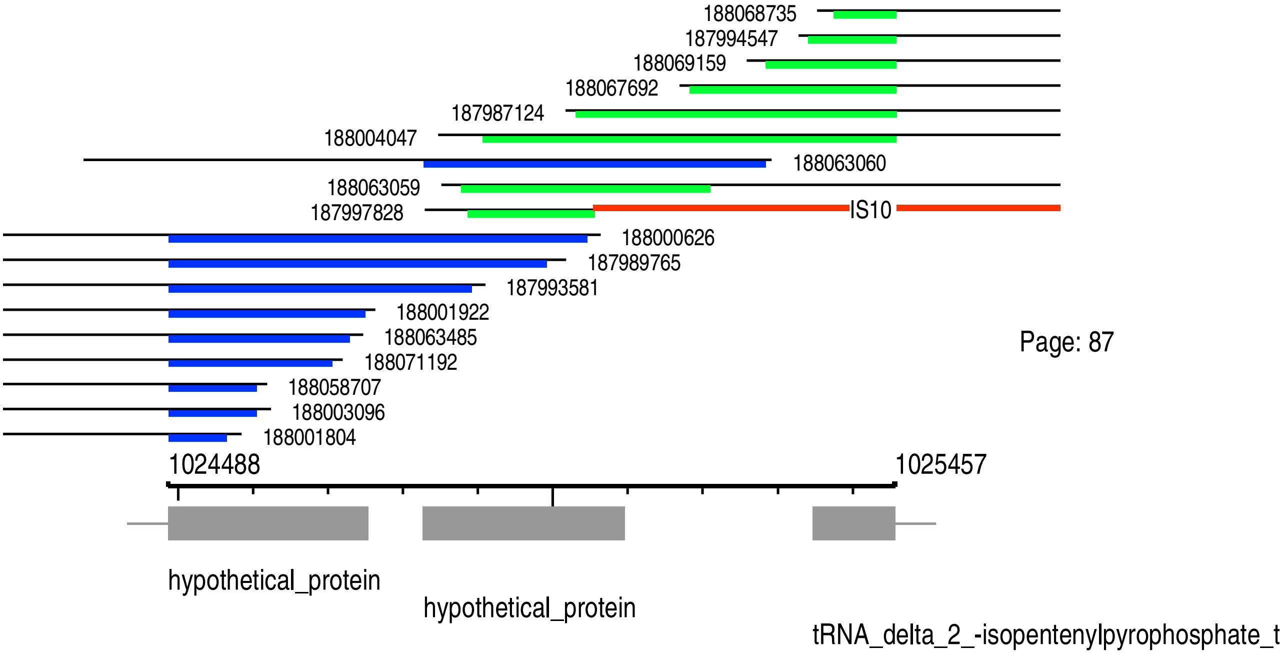

TX243275 Treponema denticola atcc 35405

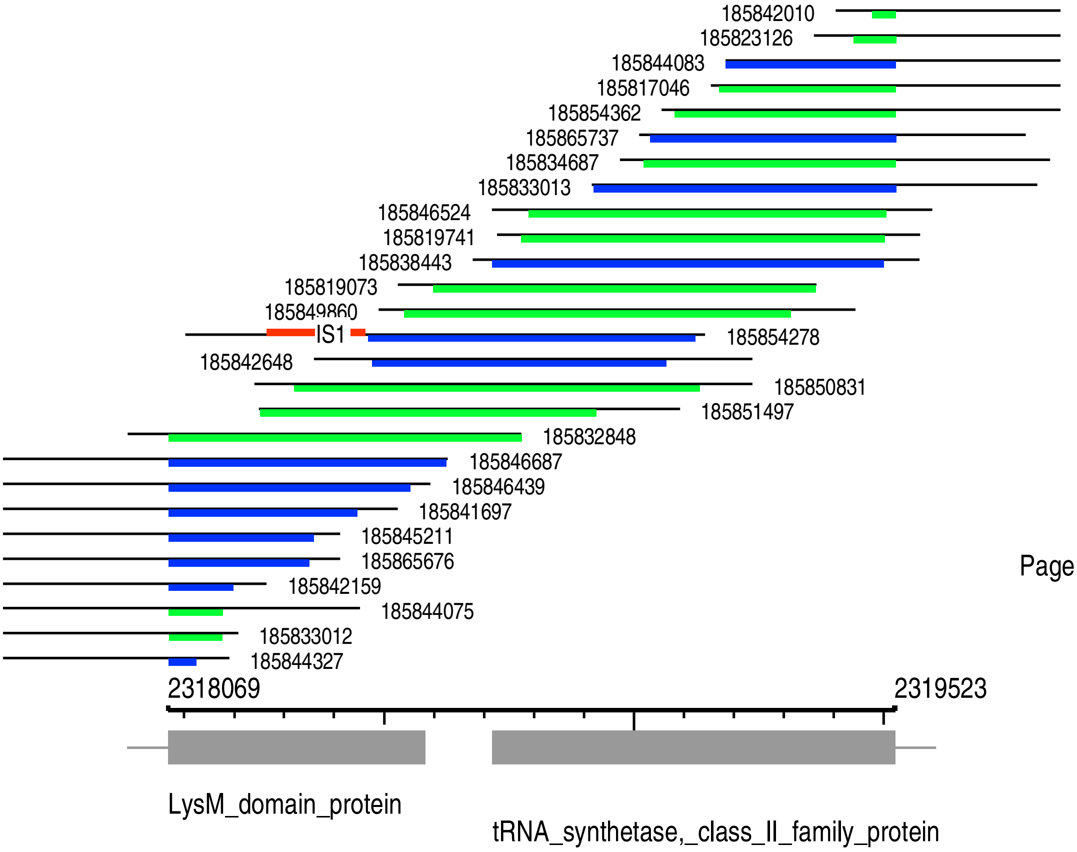

TX261594 Bacillus anthracis str ames 0581

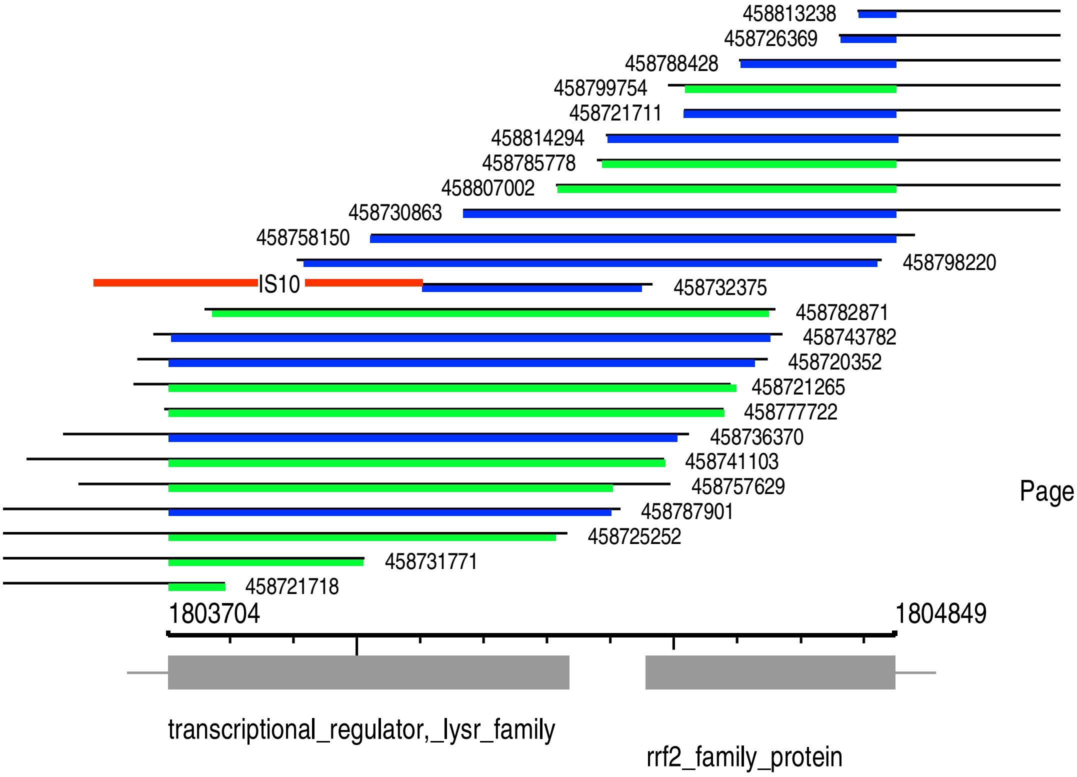

Page: 89

TX261594 Bacillus anthracis str ames 0581

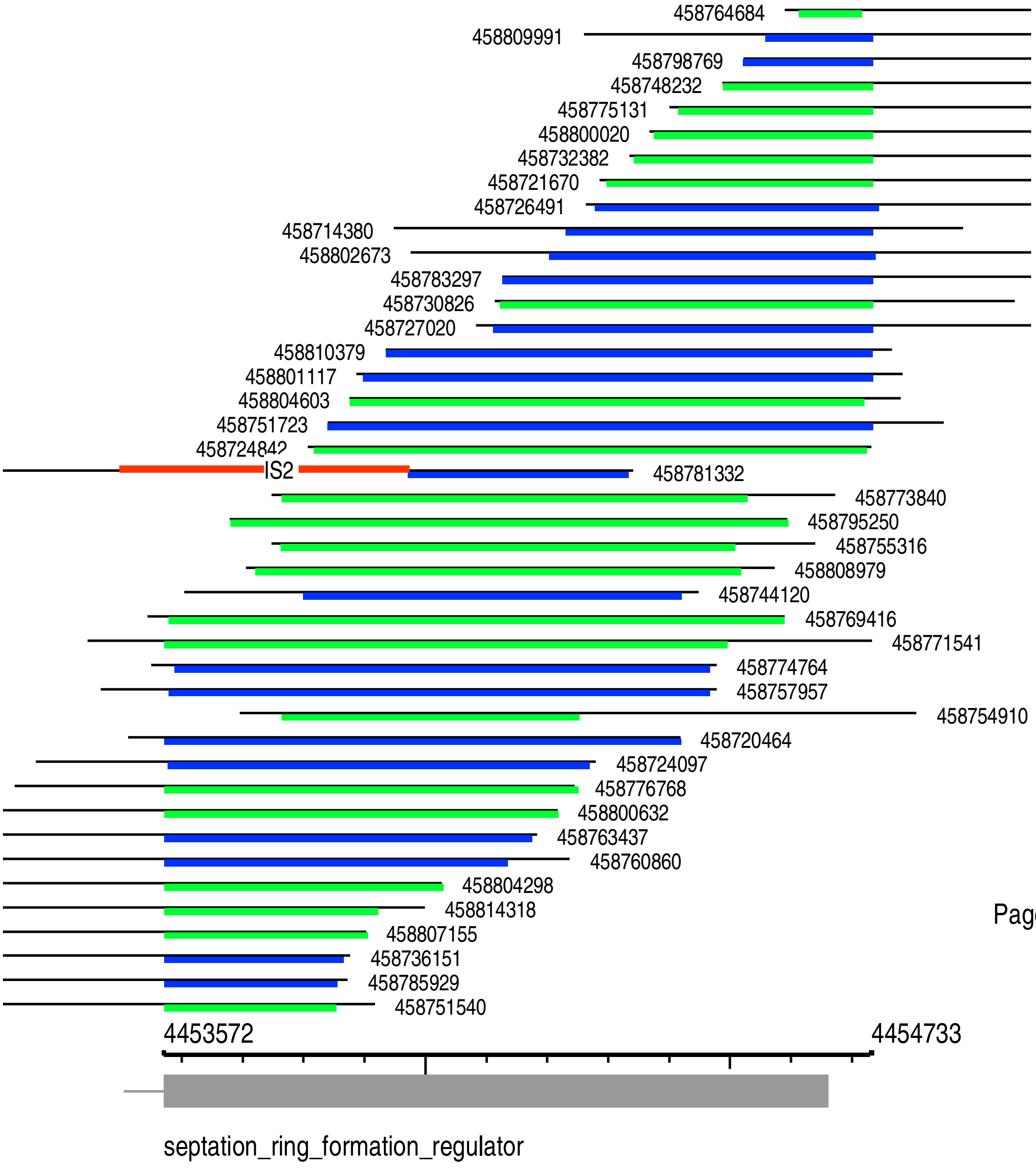

Page: 90

TX269483 Burkholderia cepacia r18194

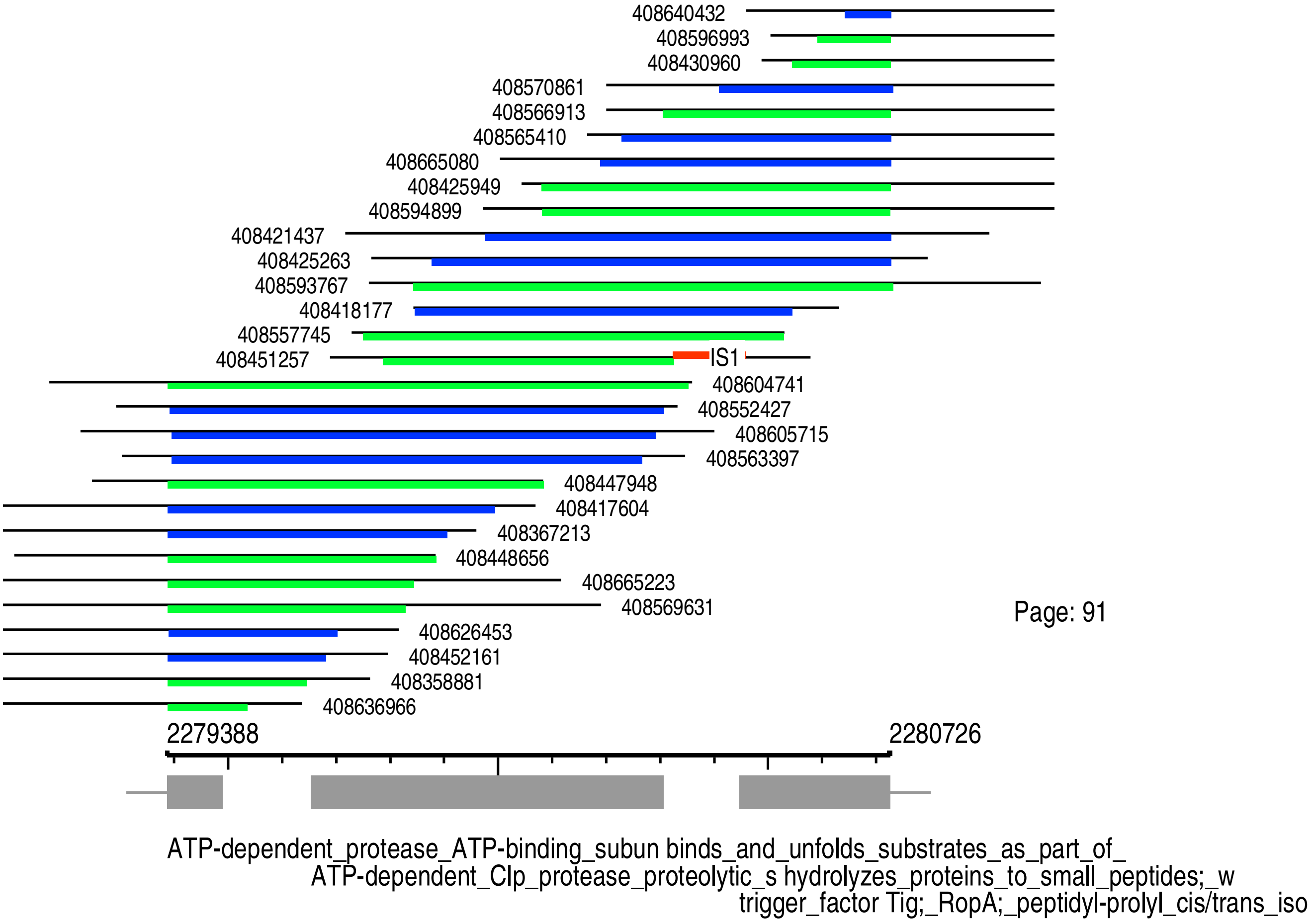

Page: 91

TX269483 Burkholderia cepacia r18194

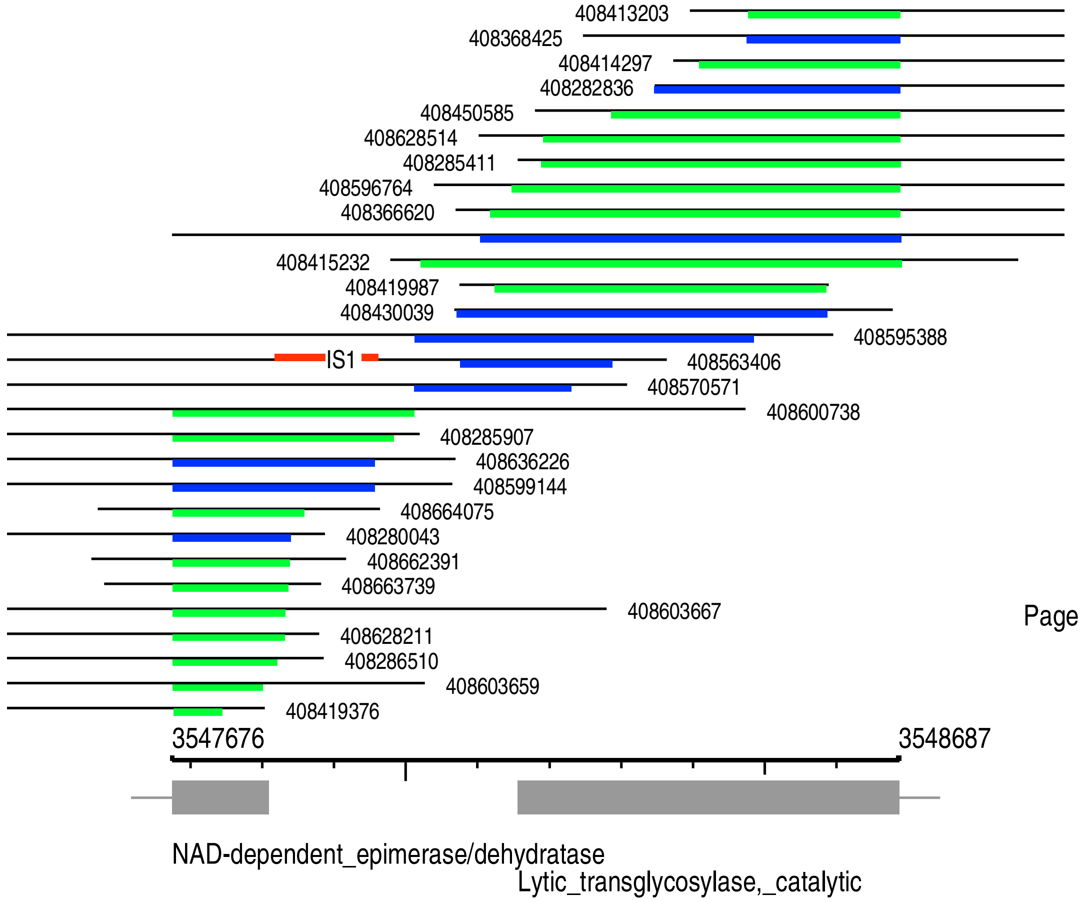

Page: 92

TX290397 Anaeromyxobacter dehalogenans 2cp-c

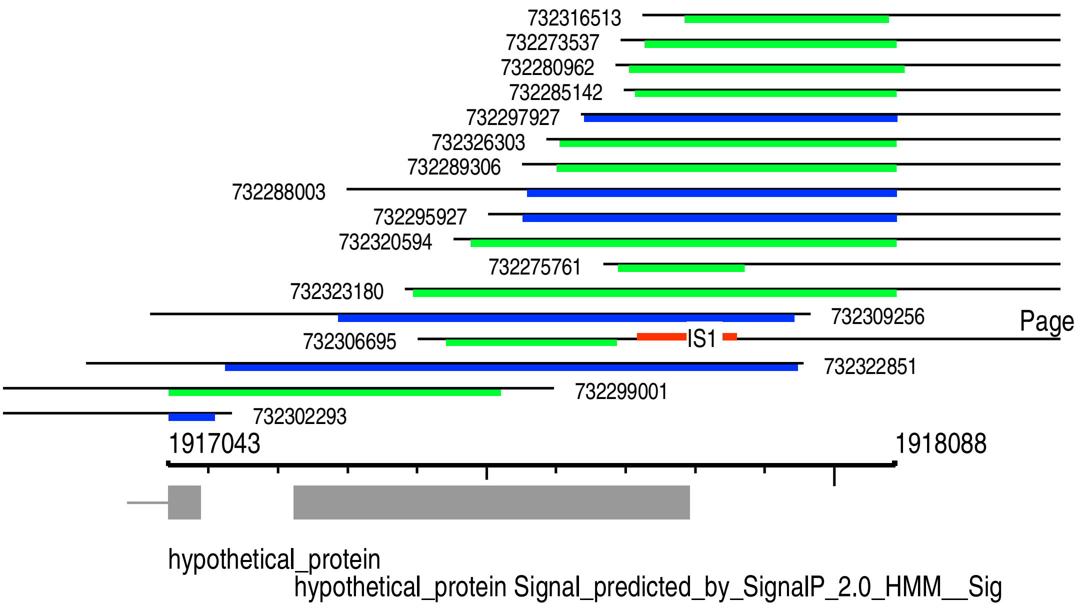

TX290397 Anaeromyxobacter dehalogenans 2cp-c

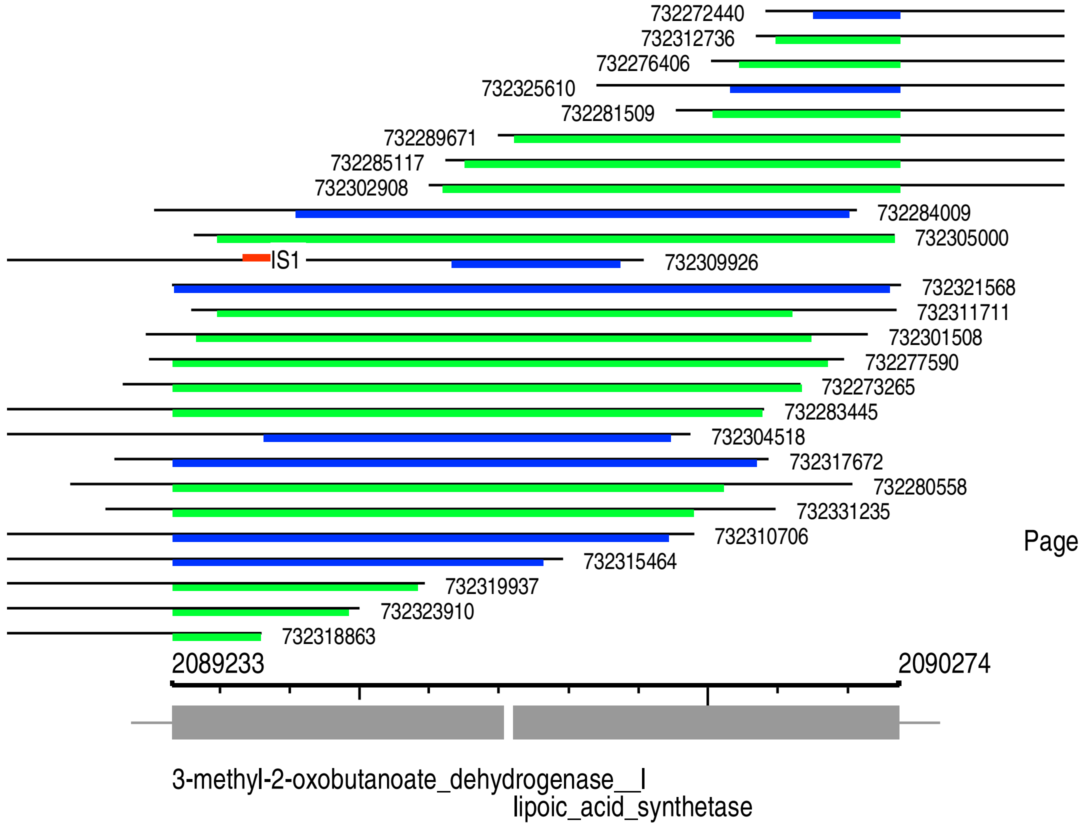

TX290397 Anaeromyxobacter dehalogenans 2cp-c

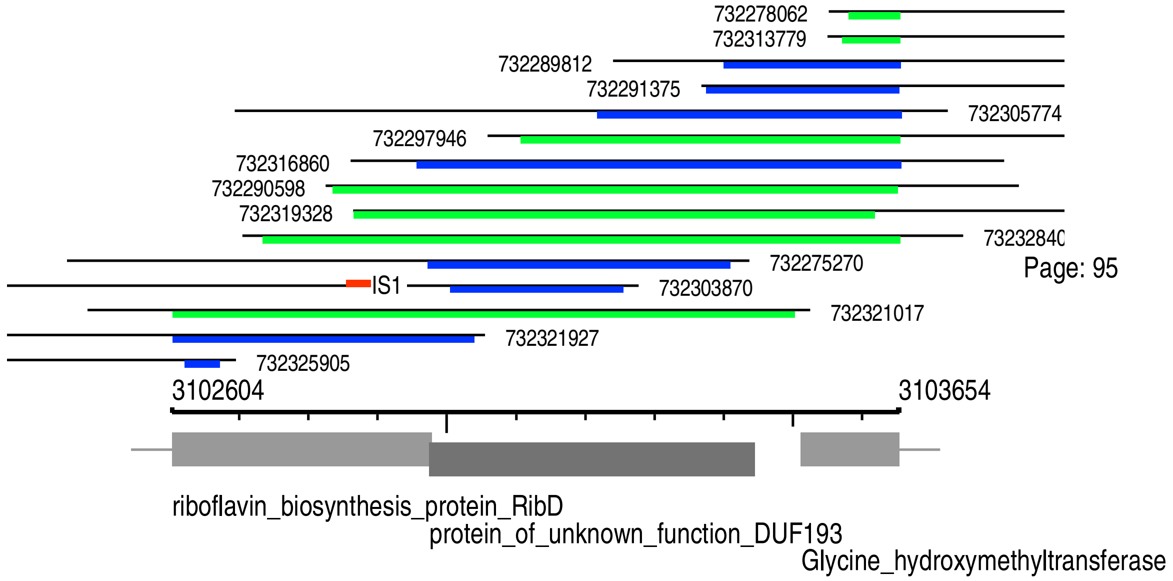

TX324925 Pelodictyon phaeoclathratiforme bu-1

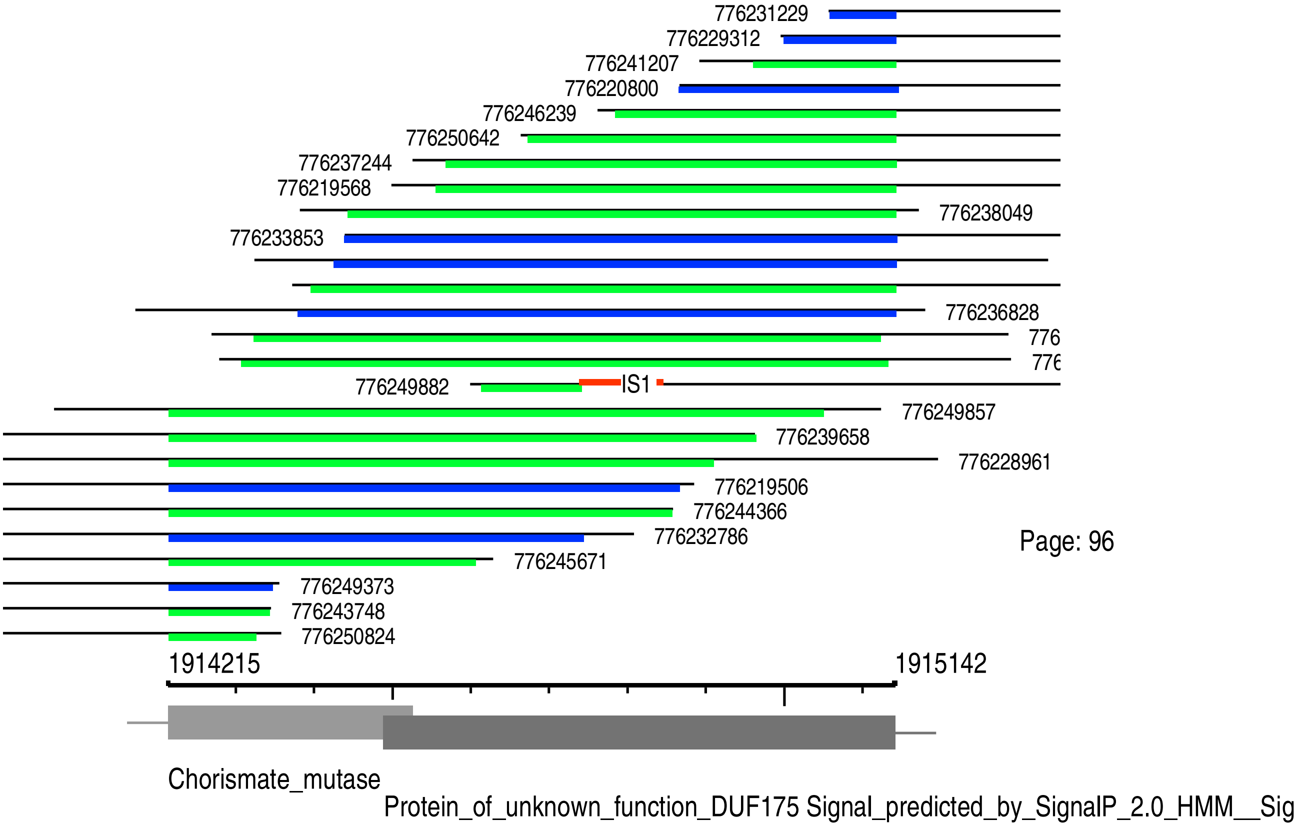

Page: 96

TX324925 Pelodictyon phaeoclathratiforme bu-1

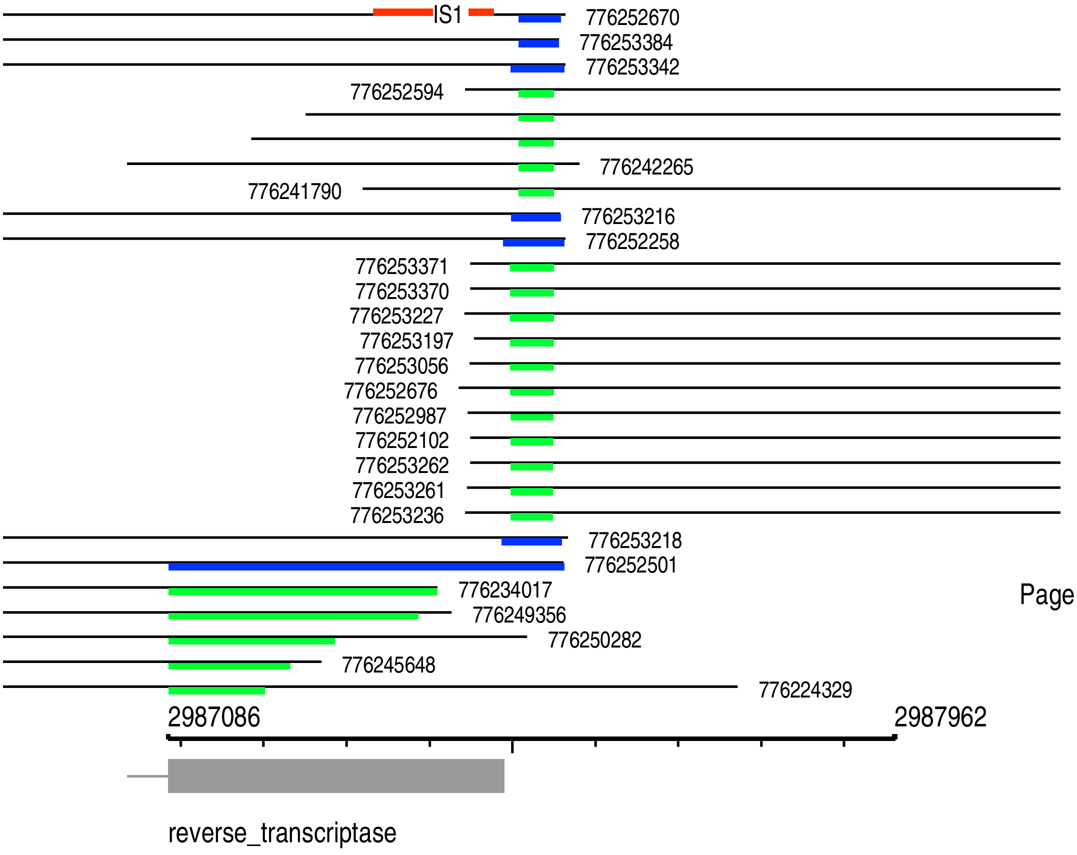

Page: 97
